# Improved reference assembly and core collection resequencing to facilitate exploration of important agronomical traits for the improvement of oilseed crop, *Carthamus tinctorius* L

**DOI:** 10.1093/gigascience/giaf151

**Published:** 2025-12-11

**Authors:** Megha Sharma, Varun Bhardwaj, Praveen Kumar Oraon, Shivani Choudhary, Heena Ambreen, Rohit Nandan Shukla, Harsha Rayudu Jamedar, Ajitha Vijjeswarapu, Vandana Jaiswal, Palchamy Kadirvel, Arun Jagannath, Shailendra Goel

**Affiliations:** Department of Botany, North campus, University of Delhi, Delhi 110007, India; Department of Botany, North campus, University of Delhi, Delhi 110007, India; Department of Botany, North campus, University of Delhi, Delhi 110007, India; Department of Botany, North campus, University of Delhi, Delhi 110007, India; Department of Biosciences, University of Exeter, Exeter EX4 4QD, United Kingdom; Bionivid Technology Pvt. Limited, Bengaluru 560064, India; ICAR—Indian Institute of Oilseeds Research, Hyderabad 500030, India; ICAR—Indian Institute of Oilseeds Research, Hyderabad 500030, India; CSIR—Institute of Himalayan Bioresource Technology, Palampur, Himachal Pradesh 176061, India; ICAR—Indian Institute of Oilseeds Research, Hyderabad 500030, India; Department of Botany, North campus, University of Delhi, Delhi 110007, India; Department of Botany, North campus, University of Delhi, Delhi 110007, India

**Keywords:** safflower, genome assembly, core collection, optical mapping, resistance genes, genome-wide association study, candidate gene analysis, haplotypes, pan-genome, KASP

## Abstract

**Background:**

Safflower (*Carthamus tinctorius* L.) is a drought-resilient oilseed crop. Besides producing edible oil rich in oleic and linoleic acids, it is also used in biofuels, cosmetics, coloring dyes, pharmaceuticals, and nutraceuticals. Despite its significant economic uses, the availability of genetic and genomic resources in safflower is limited.

**Results:**

We report an improved *de novo* genome assembly of safflower (Safflower_A2). A chromosome-level assembly of 1.15 Gb with telomeres and centromeric repeats was constructed using PacBio HiFi reads, optical maps, Illumina short reads, and Hi-C sequencing. Safflower_A2 shows better contiguity, completeness, and high-quality annotation than previous assemblies. The assembly was further validated with the help of a single-nucleotide polymorphism (SNP)–based linkage map. A genome-wide survey identified genes for comprehensive exploration of disease resistance in the safflower. Employing the *de novo* genome assembly as a reference, we used resequencing data of a global core collection of 123 accessions to carry out an SNP-based genome-wide association study, which identified significant associations for several traits and their haplotypes of agronomic value, including seed oil content. Resequencing data were also applied for a pan-genome analysis, which provided critical insights into genome diversity, identifying an additional ~11,000 genes and their functional enrichment that will be useful for region-specific breeding lines.

**Conclusion:**

Our study provides insights into the genomic architecture of safflower by leveraging an improved genome assembly and annotation. Additionally, resources, including a high-density linkage map, marker–trait associations, and pan-genome development in this study, provide valuable resources for use in breeding and crop improvement programs by the global research community.

## Introduction

Safflower *(Carthamus tinctorius L*. [NCBI:txid4222, 2n = 24]), a member of the family Asteraceae, is a drought-resilient diploid oilseed crop. The crop produces edible oil with a unique profile consisting of nutritionally desirable unsaturated fatty acids [1]. The seed oil is also a rich source of phospholipids, phytosterols, phenols, and tocopherols, which makes it highly valuable for diverse pharmaceutical and nutraceutical applications [2–5]. Safflower is currently cultivated across ~23 countries in a total area of ~1.23 million hectares, producing 1.10 million tonnes of seeds [6]. The largest global producers of the crop are Kazakhstan, Russian Federation, United States of America, Mexico, and India, accounting for more than 85% of the global seed production [6]. Currently, safflower has a market value of $232.1 million, but due to its ability to grow under drought conditions, its market is expected to increase to ~$355 million in a decade as drought conditions become more prevalent [7]. Despite its economic scope, safflower has observed a decline in acreage due to multiple factors, such as the spiny nature of the plant, susceptibility to various biotic and abiotic stresses, and scarcity of cultivars with high yield and oil content [8].

Several studies have established the diverse genetic pool of safflower harboring significant morphological, geographical, and molecular diversity [9–12]. Further, primary and secondary diversification of safflower has also led to the development of varieties with distinct traits that have emerged over time [13]. Owing to the vast variability observed in the crop, a single genome sequence would not sufficiently reflect the full repertoire of genes available in the crop. Resequencing multiple diverse genotypes encompassing the global diversity would enable the generation of crucial genomic resources that would be valuable for expediting safflower breeding programs. Currently, reference genomes are available for two safflower varieties, “Anhui 1” [14] and “Chuanhonghua 1” [15], which are region-specific and do not represent all desirable traits of global relevance. Moreover, while resequencing of 220 accessions reported by [15] provides valuable insights, ~70% accessions of the sequenced panel are from the Chinese gene pool, which does not adequately encapsulate the global diversity of the crop. This necessitates a comprehensive exploration of global germplasm for study and deployment of broader genomic diversity.

Here, we report an improved *de novo* chromosome-level genome assembly of an elite safflower accession (hereafter designated as “Safflower_A2”), characterized by several desirable traits of high agronomic value including high oil content (~47%), high oleic acid (~80%), higher seed yield, large head diameter (~24 mm), and high head number (~60 per plant) (Supplementary Fig. S1). The genome assembly was derived through integration of multiple sequencing technologies, including PacBio HiFi, Bionano optical maps, Hi-C, and Illumina paired-end short reads. Additional support for anchoring the generated genome assembly was provided by a single-nucleotide polymorphism (SNP)–based high-density genetic map generated by genotyping by sequencing (GBS) of a recombinant inbred line (RIL) mapping population (F_8_), developed using A2 as one of the parents. The genome assembly generated in this study has been utilized to generate a repertoire of resistance gene analogues (RGAs) ready to implement in crop improvement studies. Further, we performed resequencing of 116 accessions of a core collection reported earlier by our group [16] and 7 additional accessions of agronomical importance and employed it for genome-wide association studies (GWASs), which revealed crucial loci for several agricultural traits of interest, including seed oil content. Haplotype analysis was carried out to identify associated agronomically important traits. The SNPs identified were validated using the Kompetitive Allele-Specific PCR (KASP) analysis. Subsequently, we have constructed a pan-genome for safflower revealing distinct functional enrichments among pan-genes. To ensure direct availability of safflower resources and datasets to the scientific community, we present the “Safflower Genome Resource,” a comprehensive database housing generated genomic resources, including the reference genome, protein-coding genes, simple sequence repeats (SSRs), and SNPs. The database will provide essential support to the global plant breeder community in advancing trait improvement efforts in safflower.

## Results

### Development and evaluation of safflower genome assembly

The genome size of Safflower_A2 was estimated using flow cytometry and *k*-mer analysis. Flow cytometric analysis indicated an approximate size of 1.37 picograms (2C), which corresponds to ~1.34 Gb (Supplementary Fig. S2A–C) and is in line with the previously predicted size for safflower [15, 16]. Genome size estimation using *k*-mer (*k* = 17) distribution of HiFi long reads (Supplementary Table S1) gave an estimate of ~1.17 Gb (Supplementary Fig. S2D).

**Figure 1:**
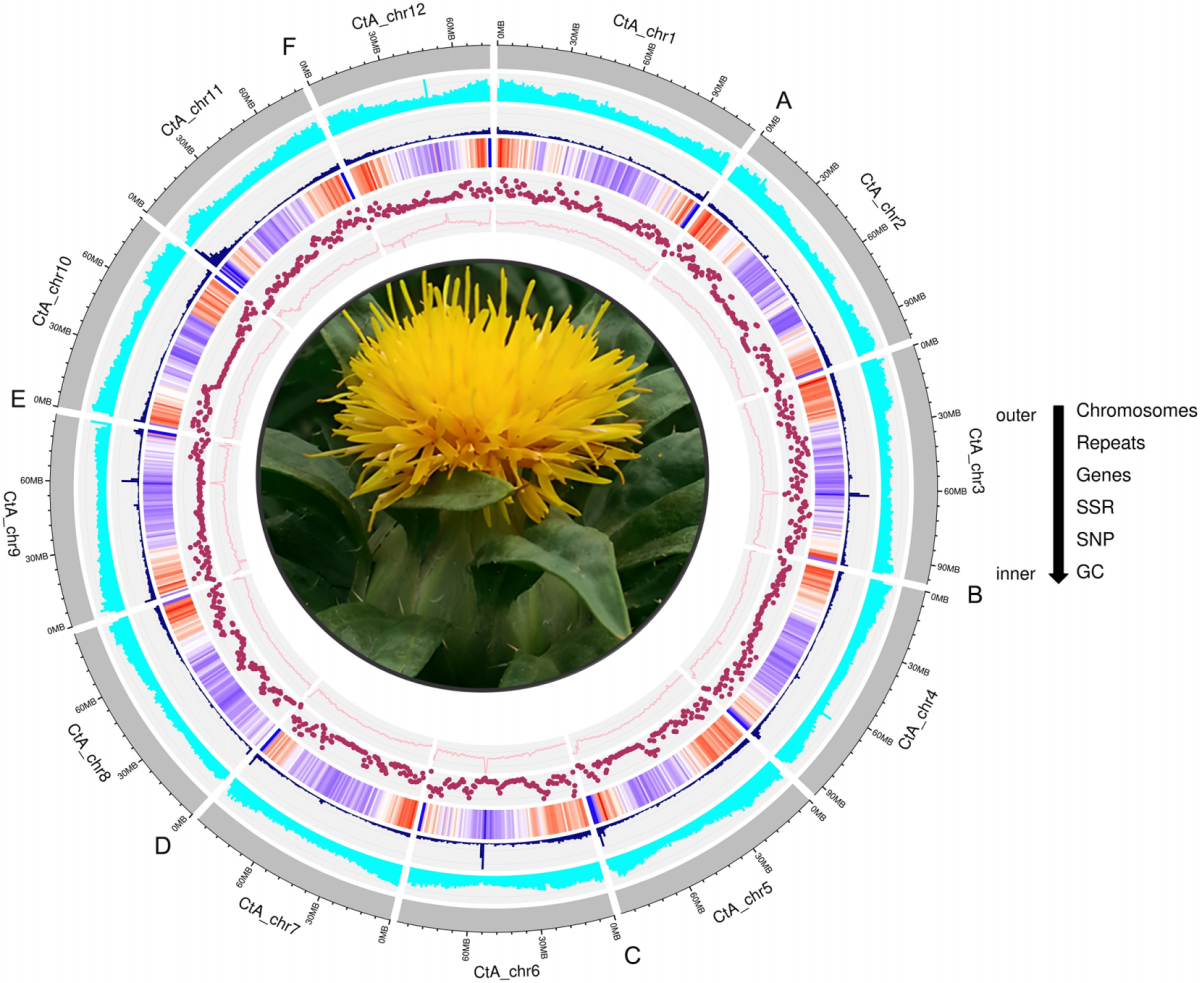
Overview of the Safflower_A2 genome. (A) The outermost layer of the circos represents the 12 assembled chromosomes. (B) Repetitive elements density. (C) Gene density. (D) Distribution of simple sequence repeats within the genome. (E) Distribution of SNPs across the chromosomes. (F) GC content of the safflower genome.

A combination of 4 different sequencing technologies, including PacBio HiFi reads, optical mapping, Hi-C, and Illumina paired-end short-read sequencing, was used for generation of the *de novo* reference genome assembly of safflower (Safflower_A2) (Supplementary Fig. S3). A total of 38.5 Gb of HiFi reads (~30× coverage), with a mean length of 13 kb and an accuracy of >99%, were generated for the construction of the contig-level genome assembly (Supplementary Table S2). First, 3,444,538 HiFi long reads were constituted into a contig-level assembly of 1.15 Gb comprising 2,427 contigs. Thereafter, the contigs were scaffolded using long optical maps, resulting in a scaffold-level assembly of 1.09 Gb comprising 31 scaffolds. Using paired-end linked reads produced by Hi-C sequencing, the 31 scaffolds generated above were integrated into 21 super-scaffolds and an additional scaffold that corresponded to the chloroplast genome. Finally, through careful manual curation, the scaffolds were anchored into 12 pseudochromosomes (2n = 24) (Fig. 1A, Supplementary Fig. S4). The total length of the final anchored assembly was ~1.09 Gb, with an N50 of 88.40 Mb and N90 of 81.10 Mb (Table 1). Our assembly further consisted of 1,680 unplaced small contigs that were <0.5 Mb in length with a total size of 68.5 Mb, making total length of the assembly 1.15 Gb. The contiguity, completeness, and accuracy of the genome were evaluated by mapping the short Illumina (99.29%) and long PacBio reads (95.91%) on to the generated assembly (Supplementary Table S3), complete BUSCO score (97.90%) (Supplementary Table S4), *k*-mer completeness score (97.73%), consensus quality value (QV) (68.94%) (Supplementary Table S5), and LTR assembly index (LAI) (22.49). The contiguous nature of the assembly enabled delineation of telomeres and centromeres in the safflower genome for the first time. The most frequent telomeric repeat was AACCCTG, with counts ranging from 7 to 1,429. We identified telomeres at one end of 9 chromosomes and at both ends of 3 chromosomes (Supplementary Fig. S5). We detected centromeric repeats on all chromosomes. These were of 4 different lengths (342 bp, 348 bp, 349 bp, and 350 bp), with counts ranging from 5 to 1,272 in the genome. The 349 bp repeat was the most abundant and present on all chromosomes except chromosomes 3 and 7 (Supplementary Fig. S5). Further, a reference based [17] chloroplast (cp) genome assembly was performed, resulting in a single circular contig of 153,026 bp (Supplementary Fig. S6). The annotation of our chloroplast assembly identified 117 cp genes, out of which 80 are protein-coding genes, 33 transfer RNA (tRNA) genes, and 4 ribosomal RNA (rRNA) genes.

**Table 1:** Comparison of Safflower_A2 genome with earlier published genome assemblies of safflower

Genome feature	Safflower_A2	Anhui_1	Chuanhonghua 1
**Assembly statistics**			
*k*-mer–based genome size estimation (in Gb)	1.17	1.17	1.17
Contigs	2427	368	3941
Length of primary assembly (Mb)	1154	1070	1171
N50 (Mb)	8.9	21.3	1.071
Pseudochromosomes	12	12	12
Length of final assembly (Gb)	1.09	1.05	1.174
N50 (Mb)	88.4	88.2	96.3
Longest scaffold (Mb)	111	106.7	185
Unplaced contigs	1,684	240	509
Size of remaining contigs (Mb)	66.3	Not available	Not available
**Quality assessment**			
Complete BUSCO	97.90%	90.70%	89.79%
Mapping proportion (long reads)	99.29%	98.10%	93.36%
**Annotation**			
Repetitive elements	71.30%	60.13%	71.41%
Number of transcripts	59,995	45,331	Not available
Complete BUSCO	91.5%	86.20%	71.70%
Average exons per gene	4.024	6.54	5.92
Mean exon length (bp)	265.84	269.59	235.66
Mean CDS length (bp)	1,215	1,265.89	Not available

Safflower_A2 exhibits better quality as compared to the other 2 published genome assemblies (Table 1). Whole-genome alignment of the Safflower_A2 assembly with the Anhui 1 [14] genome revealed high consonance between the 2 genomes (Supplementary Fig. S7A). However, the one-to-one alignment suggests that the genome assembly of Chuanhonghua 1 [15] is fragmented in nature (Supplementary Fig. S7B). The structural variant (SV) analysis detected large translocations in the Chuanhonghua 1 genome (Supplementary Fig. S7C). However, such translocations were not detected when aligned with the Anhui 1 genome (Supplementary Fig. S7D). Deletions (DEL) were the most abundant SV type across both alignments, predominantly in the 1 to 10 kb range (Supplementary Fig. S7E). Tandem duplications (DUP:TANDEM) were more frequent than interspersed duplications (DUP:INT), while inversions (INV) were less common but distributed across all size categories. Although total SV counts were higher when mapped to the Chaunhongua 1 genome, the overall size-based distribution of SVs remained consistent among the genomes (Supplementary Fig. S7F, G).

### Construction of a high-density genetic linkage map and assignment of chromosomes

A total of 151 Gb paired-end GBS data were generated for 121 lines of a RIL population (A2 × A1; designated “Population A”) with average coverage of ~1.15× (0.33×–1.82×) per individual. Variant calling yielded 1.49 million SNPs, which were filtered using stringent criteria (Supplementary Table S6), and a final set of 15,732 high-quality SNPs were used to construct the first SNP-based high-density linkage map in cultivated safflower comprising 12 linkage groups (LG1–LG12; Supplementary Fig. S8, Supplementary Table S7). The map spanned 1,581.05 cM, with linkage groups ranging in length from 65.70 cM (LG9) to 209.08 cM (LG1). The average number of markers per linkage group was 1,311 and ranged from 3,587 in LG8 to 217 in LG10. Average marker distance was 9.28 per cM, ranging from 5.72 per cM (LG3) to 24.76 cM (LG8). This genetic linkage map was anchored to the genome assembly, which showed concordance with the genetic maps, confirming the accuracy of assembled genome (Supplementary Fig. S9).

### Full-length transcriptome sequencing and detection of alternative splicing events

Transcriptomic libraries were generated from 8 different tissues of safflower: shoot, leaf, root, flower, bud and various seed developmental stages (5 days after pollination [DAP], 10 DAP, 20 DAP, and 30 DAP) (Supplementary Table S8). Long-read PacBio sequencing yielded 3,772,953 circular consensus sequence (CCS) reads, which resulted in 222,133 full-length high-quality transcripts. The obtained transcripts were aligned to the repeat-masked reference Safflower_A2 genome. The alignment data were used as evidence for gene prediction and detection of alternative splicing events. SUPPA2 detected 7 types of alternative splicing (AS) events (totaling 3,826), namely, retained intron (RI), skipping exon (SE), alternative 5′/3′ splice site events (A5S/A3S), alternative first/last exons (AF/AL), and mutually exclusive exons (MX). A5S were the most abundant and MX were the rarest type of splicing events, accounting for 57.05% and 0.26% of total local events, respectively (Supplementary Table S9).

### Annotation of the repeatome and detection of long terminal repeats for the genome expansion

We identified 787.75 Mb of repetitive elements constituting ~71.3% of the total length in the Safflower_A2 genome (Fig. 1B, Supplementary Table S10, Supplementary Fig. S10). Retrotransposons (class I transposable elements [TEs]) were the most dominant repetitive category, with its subclass long terminal repeats (LTRs) representing the major component (43.6%) of repetitive elements comprising 22.32% Ty3/*Gypsy* and 21.3% Ty1/*Copia* elements, whereas non-LTR retroelements (long interspersed nuclear elements [LINEs] and short interspersed nuclear elements [SINEs]) constituted 0.87% of the repetitive elements. Using Domain-based Annotation of Transposable Elements (DANTE), we assigned 70% of the Ty1/*Copia* elements and 65% of the Ty3/*Gypsy* elements detected by the EDTA to distinct LTR lineages (Supplementary Table S11). Among these, Ty1/*Copia*/SIRE and Ty3/*Gypsy*/Tekay were identified as the most abundant lineages in the safflower genome, with copy numbers of 65,067 and 71,143, respectively. Our results are concordant with the other members of Asteraceae [18–21]. Based on the presence of complete functional domains of LTR TEs, 3,497 Ty1/*Copia* elements and 4,716 Ty3/*Gypsy* elements were classified as intact (Supplementary Fig. S11A, B). Further, 2,195 Ty1/*Copia* and 2,879 Ty3/*Gypsy* elements were defined as autonomous, owing to the presence of target site duplication (TSD) and primer binding sites (PBSs). At the hierarchical level of lineages, Ty1/*Copia*/SIRE (1,742) and Ty3/*Gypsy*/Retand (2,193) exhibited the highest number of complete members. Phylogenetic tree of Ty3/*Gypsy* (Supplementary Fig. S11C) and Ty1/*Copia* (Supplementary Fig. S11D) grouped different subfamilies into distinct clades. However, Ty1*/Copia*/SIRE was divided into 3 clades, underlining the existing variation in this subfamily. An assessment of insertion time revealed that 87.19% of the complete LTR-TEs were inserted within the past 1 million years, indicating that transposon activity might be one of the major drivers of genome expansion in safflower (Supplementary Fig. S11E). In-depth analysis highlighted Ty3/*Gypsy*/Tekay, Ty3/*Gypsy*/Retand, Ty3/*Gypsy*/Athila, Ty1/*Copia*/SIRE, Ty1/*Copia*/TAR, and Ty1/*Copia*/Angela as the dominant contributors to this recent transposon burst. Notably, the most recent transposon burst aligns with the recent whole-genome duplication (*γ*) event in safflower [15].

DNA transposons (class II elements) constituted around 17.63% of the total repetitive elements (Supplementary Table S10, Supplementary Fig. S10). DNA transposons were further classified into tandem inverted repeats (TIRs; 7.13%), miniature inverted-repeat transposable elements (MITEs; 7.61%), and helitrons (2.88%) (Supplementary Table S10, Supplementary Fig. S10). Simple sequence repeats (SSRs) constituted 0.38% of the total repeats (Fig. 1D).

Noncoding RNA (tRNA and rRNA) genes were also surveyed in the Safflower_A2 genome assembly. We identified 4,763 rRNA genes comprising 3,619 5S type, 398 5.8S type, 377 18S type, and 370 28S type. Additionally, 1,110 tRNA genes were predicted, of which 766 coded for 20 amino acids (Supplementary Table S12).

### Annotation of protein-coding genes

**Figure 2:**
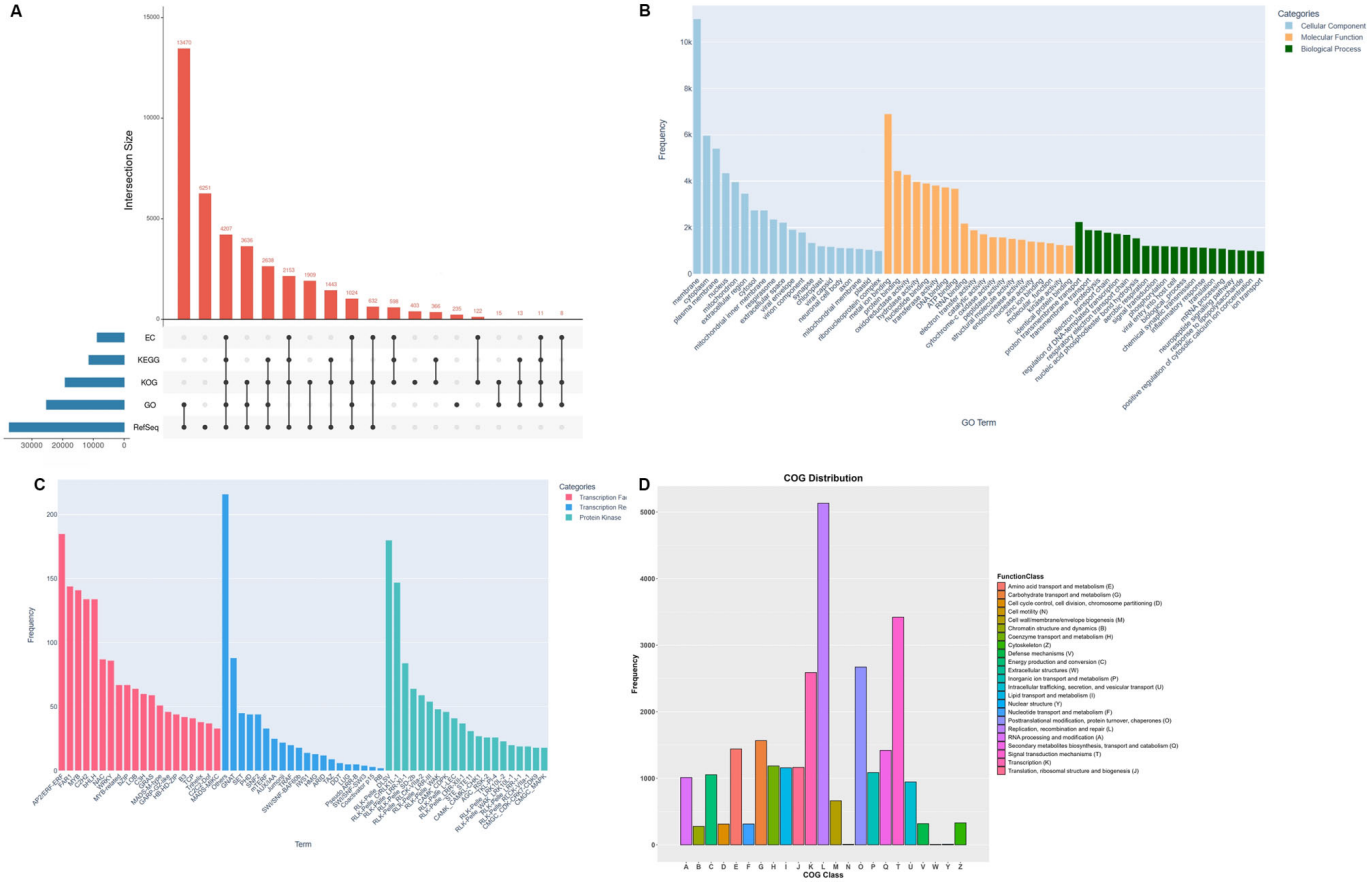
Overview of annotation of protein-coding genes for Safflower_A2 genome assembly. (A) Functional annotation of protein-coding genes using RefSeq, Cluster of Orthologous Genes (KOG), Gene Ontology (GO), Kyoto Encyclopedia of Genes and Genomes (KEGG), and Enzyme Code (EC) databases. (B) Frequency histogram showing the distribution of top 20 GO terms across 3 categories: Biological Process, Cellular Component, and Molecular Function. (C) Top 20 members of transcription factors (TFs), transcription regulators (TRs), and protein kinases (PKs) identified in the Safflower_A2 genome. (D) Distribution of KOG categories across protein-coding genes.

The safflower genome harbored a total number of 59,995 transcripts with an average of ~4.4 exons per transcript and at an average intergenic distance of ~14 kb, identified by evidence from homology and RNA sequencing (RNA-seq) methods (Fig. 1C, Table 1). After clustering through cd-hit, these 59,995 transcripts corresponded to 39,945 unigene models (at 80% similarity). The derived transcript set yielded a combined BUSCO score of 91.5%, indicating completeness of the safflower gene repertoire (Supplementary Table S13). Collectively, ~80% (47,704) of the predicted protein coding transcripts were annotated, with at least 1 functional term using publicly available databases RefSeq, Gene Ontology (GO), Enzyme Code (EC), Cluster of Orthologous Groups (KOG), Kyoto Encyclopedia of Genes and Genomes (KEGG), and Interproscan (Fig. 2, Supplementary Table S14). In addition, we could delineate a total of 2,893 transcription factors and regulators. We also identified 1,587 protein kinases.

### Identification of RGAs

**Figure 3:**
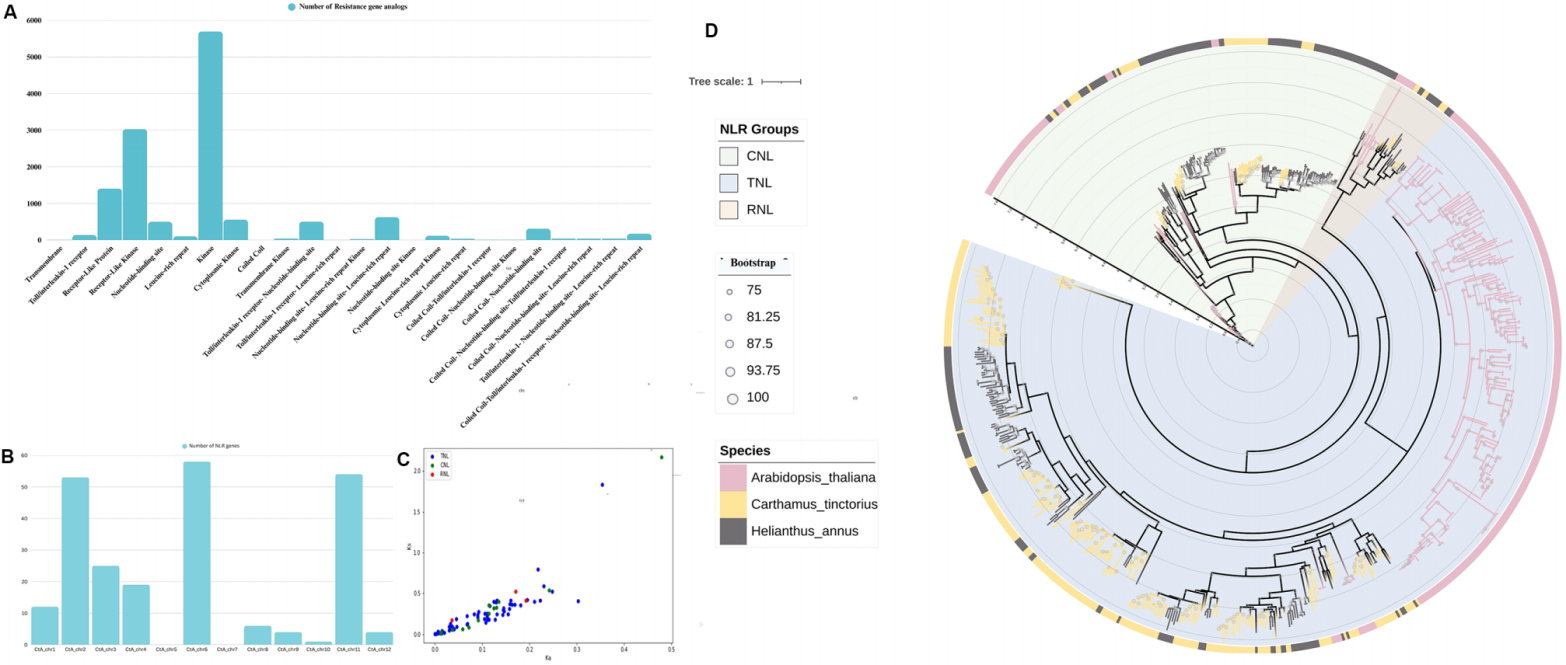
Overview of R genes in the Safflower_A2 genome. (A) Different domains present in R genes of safflower. (B) Distribution of the NLR genes on safflower chromosomes . (C) Ka/Ks analysis of NLR genes. (D) Phylogenetic analysis of NLR genes of safflower, sunflower, and *Arabidopsis thaliana*.

To facilitate genetic dissection of disease resistance in safflower, we determined a total of 2,461 putative genes encoding for RGAs, which were categorized into 24 major classes based on their constituent domains (Fig. 3A, Supplementary Table S15). Among the RGAs, the most characterized and well-known gene family for disease resistance in plants is the nucleotide-binding-site leucine-rich repeat Receptor (NLR) gene family [22], which includes Toll/interleukin 1 receptor-nucleotide binding site leucine-rich repeat (TNL), coiled-coil nucleotide-binding site leucine-rich repeat (CNL), and resistance to the powdery mildew RPW8-NBS-LRR (RNL) genes. We identified 228 non-redundant, high-confidence NLR genes encoding for 236 NLR transcripts in the safflower genome, of which 191, 38, and 7 members encoded TNL, CNL, and RNL genes, respectively. The localization of the NLRs on different safflower chromosomes exhibited biased distribution, with chromosomes 2, 6, and 11 encompassing high proportions of NLRs, while no NLRs were found on chromosomes 5 and 7 (Fig. 3B, Supplementary Fig. S12). Physically, the NLRs within each chromosome were present in multigene clusters near the telomeric region (Supplementary Fig. S12). Among the NLR genes, 75 closely related homologous gene pairs were identified, with an average Ka/Ks value of 0.49, 0.52, and 0.33 for TNL, CNL, and RNL respectively (Fig. 3B). All the NLR genes showed negative selection (Ka/Ks < 1), but 2 gene pairs also indicated positive selection (Ka/Ks > 1). We delineated several types of duplication events, including tandem (163), proximal (50), dispersed (11), and segmental (12), which accounted for ~70%, 21%, 4.6%, and 5% of all NLR genes, respectively.

We also predicted NLR genes in genomes of *Arabidopsis thaliana* [23] and *Helianthus annuus* (sunflower) [24], identifying 284 and 202 NLR genes, respectively. Phylogenetic analysis was performed incorporating the NLR genes from all 3 species. We detected 3 distinct clades: TNL, CNL, and RNL, supported by high bootstrap values (>75%) (Fig. 3D). Among these clades, NLR genes from safflower and sunflower clustered together, whereas *A. thaliana* genes were placed in a divergent clade. The collinearity analysis between safflower and *Arabidopsis* identified 144 collinear genes distributed across 30 syntenic blocks (Supplementary Fig. S13A). In contrast, the analysis between safflower and sunflower revealed 66 collinear genes within 11 syntenic blocks (Supplementary Fig. S13B).

Out of the 236 NLRs, we were able to assign the function to the 214 NLRs based on its functional annotation. Annotation of these NLRs identified proteins such as *TMV-N-like*, ROQ1, RPP13, RRS1, RUN1, and DSC1. These proteins have been shown to play significant roles in conferring resistance against a wide range of pathogens, including bacterial, viral, and fungal pathogens in different plant species (Supplementary Table S16). These NLRs might provide resistance/tolerance against the major threats such as wilt, root rots, and leaf blight. The incorporation of these resistance genes into safflower breeding programs could facilitate the development of robust, disease-resistant cultivars.

### Exploring genetic basis of various agronomically important traits in safflower

A total of 2.05 Tb of resequencing data were generated for 123 accessions with an average coverage of 15.3× per accession (Supplementary Table S17). Variant calling of the safflower core collection identified ~13 million raw SNPs and ~2.3 million small indels. We obtained a final set of ~1.8 million SNPs after robust filtering (Supplementary Table S18; Supplementary Table S19).

**Figure 4:**
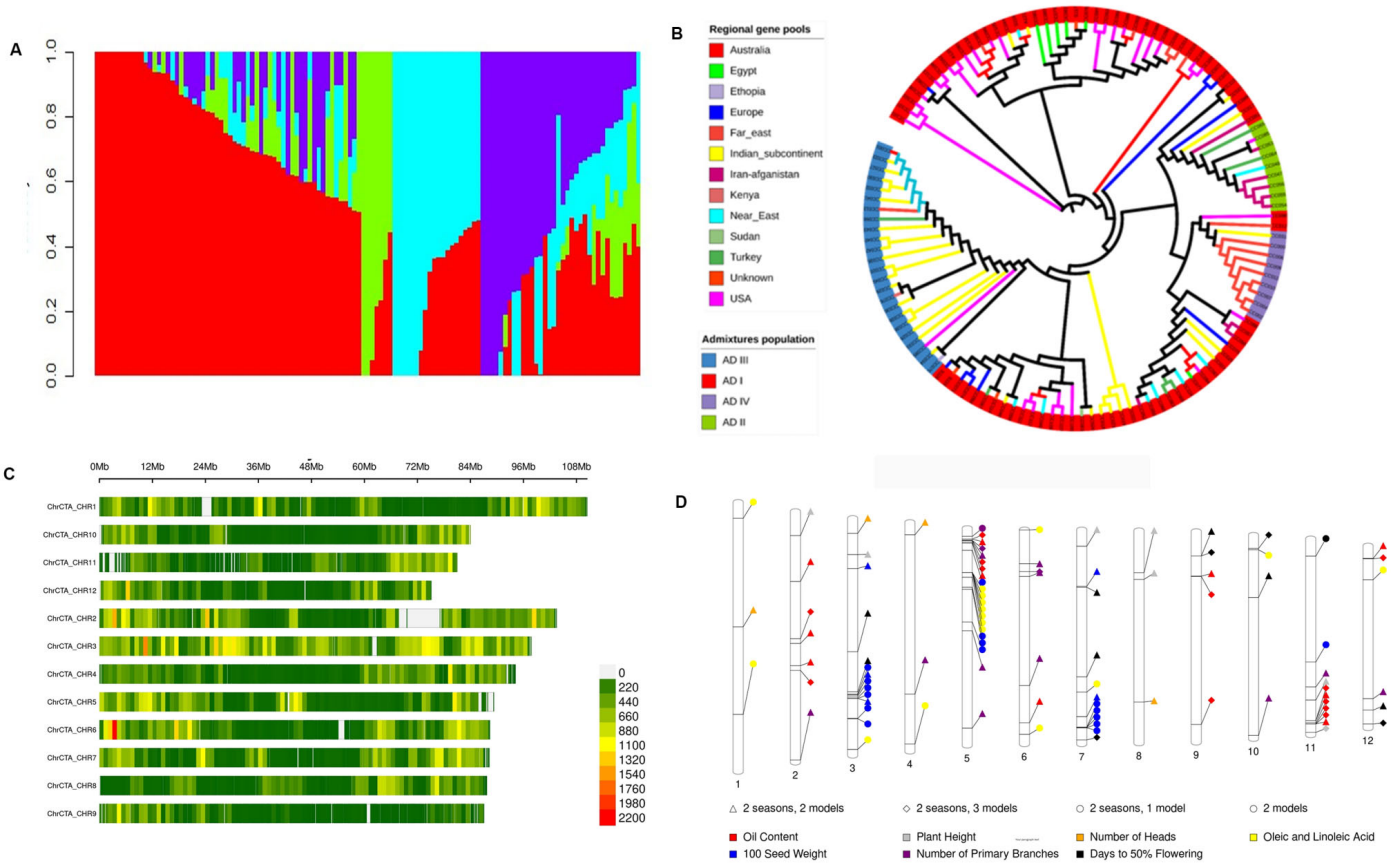
Diversity analysis and genome-wide association study of safflower core collection. (A) Admixture plot showing 4 subpopulations: ADI (red), ADII (green), ADIII (cyan), and ADIV (purple). (B) Phylogenetic tree showing evolutionary analysis. (C) Marker density plot for 320,399 SNPs used in GWAS analysis for chromosomes 1 to 12; colors indicate marker density per Mb. (D) Ideogram of QTNs detected from GWAS. QTNs for oil content, oleic and linoleic acid, 100-seed weight, plant height, head number, primary branches, and days to 50% flowering detected across 2 growing seasons (2011–12 and 2012–13) are shown according to their respective chromosomal (labeled 1–12) positions.

The population structure analysis of the core collection identified 4 major clusters (*K* = 4; membership coefficient [qi] ≥0.5), designated as ADI, ADII, ADIII, and ADIV and comprising 62, 6, 19, and 16 accessions, respectively (Supplementary Table S20). ADI comprised a large number of accessions from different continents, while ADIII delimited primarily Indian accessions (Fig. 4A). Principal component analysis (PCA) was performed, and the first 2 principal axes, PC1 (18%) and PC2 (13%), were plotted (Supplementary Fig. S14A). All accessions from ADI clustered together in quadrants 2 and 3 of the PCA. Quadrant 3 comprised accessions from the United States, while quadrant 2 consisted of accessions from other regional gene pools. All accessions from ADIII clustered in quadrant 4, whereas ADII and ADIV accessions were clustered in quadrant 1. Based on the phylogenetic tree, 4 major clusters (NJI–NJIV) were observed (Fig. 4B). Most ADI accessions clustered together in NJI. ADII accessions, along with some ADI accessions, were in NJII. NJIII comprised accessions from ADIII, while NJIV had accessions from ADIV and ADI. Our population genetic structure analysis, based on ADMIXTURE, PCA, and phylogenetic analysis, was able to infer consistent phylogenetic relationships between the accessions. F_st_ divergence was estimated between the populations. ADI and ADIII showed high genetic divergence (F_st_ = 0.45), whereas ADII and ADIV showed minimum genetic divergence (F_st_ = 0.239). Linkage decay (LD) of the resequenced accessions indicated that the LD decreased to half (*r*^2^ = 0.15) from its maximum at ~6 kb (Supplementary Fig. S14B). The overall LD was similar for all chromosomes ranging from 0.15 to 0.10, which is in consonance with earlier reports on sunflower [25].

Phenotypic data for all traits represented in the core collection are sourced from our earlier study [16], showing a broad spectrum of variability with a normal distribution. Pearson’s correlation analysis demonstrated strong concordance (>90%) in phenotypic data across 2 growing seasons for traits, including oil content (OC), plant height (PH), and days to 50% flowering (DTF). However, for traits like 100-seed weight (100SW), the number of primary branches (PB), and the number of heads (HN), the correlation was moderate (0.61 to 0.81). Phenotypic data for oleic acid and linoleic acid (OA-LA) content was available for only 1 growing season, and therefore, seasonal concordance could not be assessed for these traits. SNPs (~1.8 million) generated were further filtered to a final set of 320,399 (filtering criteria summarized in Supplementary Table S21 and SNP distribution in Supplementary Table S22 and Fig. 4C). We identified 3,159 significant marker–trait associations (MTAs) collectively for all 8 agronomic traits over 2 growing seasons at *P* < 0.0001. The population structure analysis indicated 4 subpopulations in the core collection; hence, the analysis was carried out keeping the number of principal component (PCs) as 4. The quantile–quantile (Q-Q) plots were analyzed to identify the best-fitting models for each trait, and the analysis revealed that multilocus models were best suited for detecting significant associations. Only the MTAs that were following specific criteria were retained (outlined below) and called quantitative trait nucleotides (QTNs). For traits OC, PH, and DTF, the QTNs were consistently identified across all 3 multilocus models in both growing seasons. In contrast, traits HN, PB, and 100SW showed more seasonal variability; hence, QTNs were defined as those present in at least 1 multilocus model and both growing seasons. OA and LA data were available for only 1 growing season; thus, QTNs for these traits were identified using the best-fitting model, multilocus mixed model (MLMM). A total of 96 QTNs were identified for 8 traits on all chromosomes (Fig. 4D, Supplementary Table S23).

LD block analysis revealed that the average size of the LD block in the QTN region was ~6.7 kb (Supplementary Table S24). Consequently, candidate genes were searched within 7 kb upstream and downstream of the QTN positions. A total of 32 candidate genes corresponding to 33 QTNs were identified across all traits based on their putative functions reported in the literature. Haplotypes were identified in the LD block for QTNs with putative functions, and their associations with the phenotypes were further analyzed (Table 2, Supplementary Fig. S15). Selected QTNs were subsequently validated using KASP assays (Supplementary Table S25, Supplementary Table S26, Supplementary Fig. S16), providing additional support for the genetic associations identified.

**Table 2:** Table representing QTNs marking candidate genes and associated haplotypes for various important agronomical traits in safflower

Trait	QTN	Chromosome	Position	Alleles	Candidate gene and its annotation	Number of haplotypes	Number of SNPs in haploblock	Number of accessions exhibiting the haplotypes	Favorable haplotype(s)	Haplotype location with respect to gene	Distance from gene (bp)	Superior haplotypes
**100-seed weight**	SW10	3	74251721	G,A	g35324; RNA-binding protein 2-like isoform X2	60	40	112	H001 (53)	Downstream	67	H019, H021
	SW23	3	74075377	C,T	g35305; Peptidyl-prolyl *cis-trans* isomerase CYP57 isoform X1	50	17	118	H001 (57), H002 (4), H003 (3), H004(3)	Downstream	10	H001, H031, H002
	SW3	3	72485086	A,G	g35324; Xyloglucan galactosyltransferase XLT2	48	32	115	H001 (61), H002 (6)	Gene within haplotype	0	H024, H008, H030
	SW31	5	9066250	G,A	g42789; Oleosin-B6-like	28	13	122	H001 (94)	Gene within haplotype	0	H021, H002
	SW37	7	81516758	T,A	g57921; Protein FRIGIDA-ESSENTIAL 1-like isoform	30	16	122	H001 (93)	Downstream	199	
**Days to 50% flowering**	DTF7	3	54974331	C,T	g34050; Exocyst complex component SEC5A-like isoform X2	74	26	113	H001 (39), H002 (2)	Gene within haplotype	0	H031, H029, H030
	DTF10	7	86666550	G,T	g58272; E3 ubiquitin-protein ligase COP1-like	80	13	115	H001 (29), H002 (3), H003 (3)	Upstream	605	H058, H053, H080
	DTF2	11	7169035	C,T	g12806; E3 ubiquitin-protein ligase UPL1-like	13	6	123	H001 (89), H002 (7), H003 (6), H004 (5), H005 (4), H006 (3)	Downstream	4,954	H001, H005, H006
	DTF2	11	7169035	C,T	g12807; 40S ribosomal protein S8	13	6	123	H001 (89), H002 (7), H003 (6), H004 (5), H005 (4), H006 (3)	Gene within haplotype	0	H001, H005, H006
**Number of heads**	HN5	4	5068529	G,A	g37215; Alpha-xylosidase 1-like	40	23	115	H001 (76)	Upstream	530	H01, H024
**Oleic and linoleic acid**	OA-LA15	5	18156971	C,G	g43426; Cytochrome P450 71A4-like	21	26	123	H001 (53), H002 (36), H003 (11), H004 (6)	Upstream	14,331	H001, H003
	OA-LA17	5	18157188	A,G	g43427; Cytochrome P450 71A4-like	21	24	123	H001 (53), H002 (36), H003 (11), H004 (6)	Upstream	26	H001, H003
	OA-LA20	5	18141916	A,C	g43426; Cytochrome P450 71A4-like	14	23	117	H001 (73), H002 (25), H003 (8)	Upstream	43	H001
	OA-LA33	5	18179456	T,A	g43429 and g43430.t1; Cytochrome P450 71A2-like	18	18	122	H001 (85), H002 (21)	Downstream	5,875	
	OA-LA35	6	84242096	T,A	g53664; Probable calcium-binding protein CML25	52	20	120	H001 (69)	Upstream	2,183	H001
	OA-LA5	7	66993411	G,A	g57141; Cytochrome P450 710A11-like	26	16	121	H001 (70), H002 (27)	Upstream	608	
**Oil content**	OC2	2	65505220	T,C	g28700; Pentatricopeptide repeat-containing protein At4g20740	83	16	122	H001, H002 (3), H003 (3), H004 (3)	Upstream	71	H001, H096
	OC4	5	5164369	C,T	g42440; Purple acid phosphatase 27	11	6	123	H001 (82), H002 (14), H003 (9), H004 (7), H005 (3), H006 (2)	Gene within haplotype	0	H001, H002
	OC6	9	18518779	A,G	g64666; Probable UDP-N-acetylglucosamine–peptide N-acetylglucosaminyltransferase SPINDLY isoform X1	16	15	122	H001 (107)	Gene within haplotype	0	H006, H007, H009, H001
	OC9	9	79566941	G,A	g67871; Retrovirus-related Pol polyprotein from transposon TNT 1–94	3	2	122	H01 (108), H02 (8), H03 (7)	Upstream	2,428	H001, H003
	OC1	11	76393484	G,A	g16920; Protein BIG GRAIN 1-like E	71	40	116	H001 (30), H002 (7), H003 (6), H004 (3), H005 (3), H006	Gene within haplotype	0	
	OC8	11	76395157	C,T	g16920; Protein BIG GRAIN 1-like E	67	39	116	H001 (33), H02 (7), H003 (6), H004 (3), H005 (3), H006 (3)	Gene within haplotype	0	H001, H040, H058
**Number of primary branches**	PB4	5	5018372	A,C	g42435; Acetolactate synthase 1, chloroplastic	29	14	122	H001 (94)	Upstream	3,704	H001, H028
	PB9	5	3333942	T,C	g42292; F-box/LRR-repeat protein 14-like isoform X1	26	15	122	H001 (96)	Upstream	688	H001, H008
	PB1	12	63076322	C,T	g20942. and g20943;Putative pentatricopeptide repeat-containing protein At1g12700, mitochondrial	31	17	119	H001 (86), H002 (3)	Gene within haplotype	0	
**Plant height**	PH3	2	10029119	A,G	g25891;Vignain-like	63	13	122	H001 (54), H002 (6)	Downstream	1,031	H001, H048
	PH4	3	15050693	G,A	g32062;Trafficking protein particle complex subunit 6B (TRAPPC6B)	20	19	122	H001 (103)	Downstream	872	

**Figure 5:**
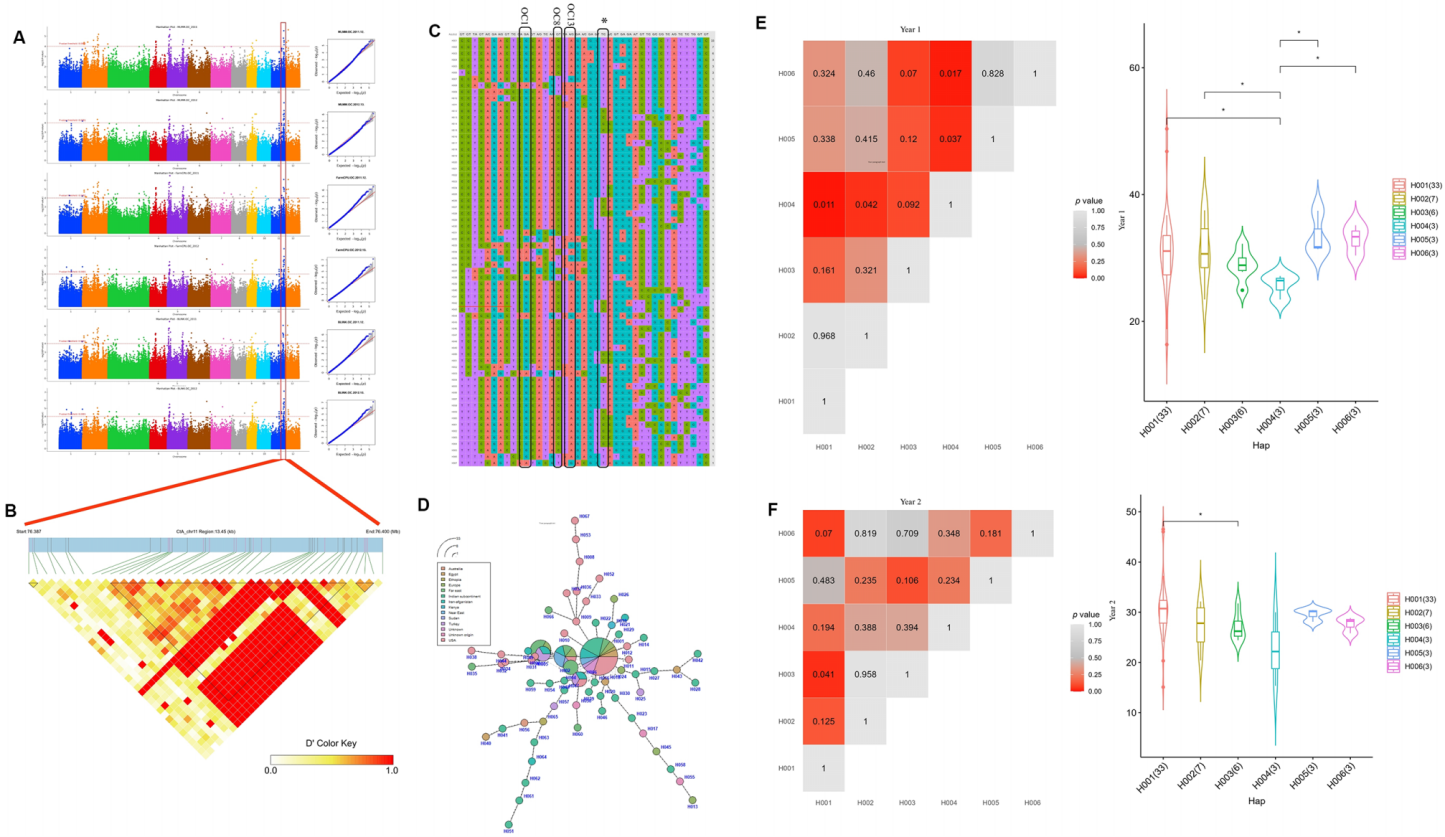
Exploration of the genetic basis of oil content in the safflower. (A) Q-Q and Manhattan plots for oil content representing all the multilocus models. (B) LD-Block of 7 kb depicting the correlation between the SNPs for the oil content. (C) Haploblocks comprising the QTN OC1 (CtA_chr11_76393484), OC8 (CtA_chr11_76395157), OC13 (CtA_chr11_76395310), and SNPs of gene BIG GRAIN (represented by *). (D) Haplo-network representing the network of the haplotypes. Correlation of the favorable haplotypes with phenotypic data for (E) season 1 (OC_1: 2011–12) and (F) season 2 (OC_2: 2012–13).

Being an oilseed crop, OC is the most important trait for safflower. Three QTNs, OC1, OC8, and OC13 (Fig. 5A, B), marked the gene BIG GRAIN 1-like protein, a positive regulator of auxin transport and signaling, reported to control grain size in rice by modulating cell division [26]. Interestingly, we found OC1, OC8, OC13, and BIG GRAIN within the same haploblock (Fig. 5C). OC8_H01 is the most geographically distributed, whereas OC8_H04 is the least represented haplotype (Fig. 5D). OC8_H05 and OC8_H06 encode for moderate to high oil (>25%) (Fig. 5E, F, Supplementary Fig. S15A, B). KASP assay validated QTNs OC1 and OC8, along with 4 SNP sites (76395211, 7639615, 76395777, 76397432), which were 1.5 kb upstream to the BIG GRAIN 1-like gene. Another QTN, OC12, lies in the vicinity of a gene encoding myosin-binding protein (MYOB2). MYOB2 is known to be a lipid droplet associated protein in *Arabidopsis* leaves, which may be involved in enhancing lipid transport and storage [27]. OC12_H02 is the haplotype associated with high oil (30% to 55%) (Supplementary Fig. S15C) and, henceforth, validated using the KASP assay.

For trait SW, QTN SW23 is downstream of a CYP57 isoform, known to regulate cell division and elongation processes that influence seed size and weight in *Arabidopsis* [28]. Another QTN, SW3, is in the regulatory region of the xyloglucan galactosyltransferase gene, involved in hemicellulose modification of primary cell walls of most dicotyledonous plants [29]. Haplotype analysis detected that SW23_H02 is associated with high seed weight, whereas SW3_H02 is associated with low to moderate seed weight (2.5–5 g) (Supplementary Fig. S15D, E). A KASP panel of 8 accessions validated the presence of QTN SW23 and the 2 associated SNPs with the gene. QTN SW31 lies in the upstream region of the oleosin-B6-like protein, which is associated with lipid droplet stability and oil body formation in seeds, potentially playing a role in seed weight and energy storage [30]. QTN SW10 is located downstream of the gene encoding RNA-binding protein 2 (RBP), implicated in post transcriptional regulation. RBPs, such as APUM24, are known regulators of seed development [31] (Supplementary Fig. S15F, G). Another QTN, SW39, is associated with the OFP9 transcriptional repressor protein, known to regulate grain size in rice through hormonal modulation [32]. QTN SW10 and SW39 were verified by KASP analysis.

For oleic and linoleic acid, we detected 6 QTNs (OA_LA15, OA_LA17, OA_LA20, OA_LA33, OA_LA45, and OA_LA44) on chromosome 5 associated with cytochrome P450 71A4-like, which is involved in fatty acid catabolism via epoxidation [33]. Haplo-pheno analysis (Supplementary Fig. S15H–K) suggests that QTNs associated with cytochrome P450 71A4-like resulted in low to moderate OA, but no correlation was detected with LA. It is possible that FAD and cytochrome P450 71A4 are competing for the same substrate (oleic acid), but further studies are required to explore this hypothesis [34]. KASP analysis validated the presence of OA_LA17 in the panel comprising both high and low linoleic acid lines. Haplo-pheno analysis of QTN OA_LA20 identified a haplotype (OA_LA20_H002) that was associated with low oleic acid content (Supplementary Fig. S15J). Two SNPs were identified, which might play a significant role in regulating fatty acid content in safflower. Both SNPs were validated by KASP analysis.

For DTF, we identified QTN DTF2, associated with the gene encoding E3 ubiquitin-protein ligase UPL1-like, known to regulate various developmental processes, including flowering [35]. QTN DTF10 was found in the proximity of E3 ubiquitin-protein ligase COP1-like, which accelerates the degradation of GIGANTEA (GI) via the 26S proteasome, thereby delaying flowering under low-temperature conditions [36]. Haplotype analysis identified DTF2_H05 and DTF2_H06, associated with a reduction in the number of days to flowering, whereas DTF10_H02 and DTF10_H03 were responsible for increasing DTF (Supplementary Fig. S15L, M). Validation of DTF2 and 1 corresponding SNP was done using KASP, lending further support to our analysis.

For pre-harvest traits such as PH, QTN PH3 (on chromosome 2) is downstream of the gene encoding a vignain-like protein, a cysteine protease, involved in resource allocation or tissue remodeling, which is critical during active growth [37]. For PH3, we detected 2 haplotypes PH3_H02, which led to the increased height in season 1 but not in season 2 (Supplementary Fig. S15N, O). QTN PH4 (validated using the KASP) lies in the vicinity of the gene coding for trafficking protein particle complex subunit 6B, a component of the transport protein particle (TRAPP) complex, which is involved in vesicle transport, important for plant growth and development [38].

For PB, QTN PB14 is located near TF Teosinte branched 1/Cycloidea/proliferating cell factor T (TCP20), which modulates plant development by influencing hormonal pathways, including brassinosteroid biosynthesis, which is closely linked to branching and shoot architecture [39, 40]. Another QTN, PB9, lies upstream of a gene encoding F-box/LRR-repeat protein 14, involved in the auxin signaling pathway and programmed cell death. Haplotype analysis revealed that PB14_H02 shows an association with a low number of primary branches (Supplementary Fig. S15P). QTN PB14 and PB9 were validated using the KASP analysis.

For HN, QTN HN5 lies upstream of the gene coding for alpha-xylosidase 1, critical for maintaining cell wall integrity and enhanced cell wall loosening in the elongating floral stem, but only 1 major haplotype was detected [41].

### Quantitative reverse transcription PCR validation of candidate genes for OC, OA-LA, and SW

As an oilseed crop, post harvest traits are crucial for safflower. For expression profiling of genes associated with seed-related traits (OC, OA–LA, and SW), 6 representative candidate genes were shortlisted based on the *P* values (*P* < 10^−4^) of their respective QTNs (Supplementary Table S23). Gene expression was checked in transcriptome data of a published study [14]. Gene expression was assayed by quantitative reverse transcription PCR (qRT-PCR) to measure transcript levels at 4 seed developmental stages (5, 10, 20, and 30 DAP) in 3 selected genotypes (CC113, CC106, and S116) showing contrasting phenotypes for these traits (Supplementary Table S27).

For OC, 4 genes were selected (*g16890:* BIG GRAIN 1-like E, *g16872*: myosin-binding protein 2, *g64666:* SPINDLY isoform X1, *g42440:* purple acid phosphatase 27), which showed consistently low *P* values across different models and growing seasons (Supplementary Table S28). The primers were designed for all 4 genes, and unambiguous amplification was obtained in g16872 and g64666 (Supplementary Table S28). The gene *g16872* (myosin-binding protein 2) was strongly upregulated at 10 DAP in CC106 but remained low in CC113 and S116, a pattern consistent with CC106’s high oil content (Supplementary Fig. S17A, Supplementary Table S29). However, gene *g64666* (SPINDLY isoform X1) did not show a consistent differential expression pattern between the 4 selected developmental stages and need to be investigated further (Supplementary Fig. S17B, Supplementary Table S29). For OA-LA, gene *g43426* (cytochrome P450) was located in the vicinity of the 3 strong QTNs exhibiting low *P* values (Supplementary Table S28) and was therefore selected for validation. RT-PCR analysis showed a strong upregulation at 10 DAP in 2 LA-rich genotypes, CC113 and S116, as compared to OA-rich CC106 (Supplementary Fig. S17C, Supplementary Table S29). For SW, gene *g57921* (FRIGIDA-ESSENTIAL 1-like isoform) was selected since QTN SW37 showed strong *P* values (*P* < 10^−4^) across 2 growing seasons (Supplementary Table S23). The gene exhibited strong upregulation at 20 DAP, in S116, followed by CC113 and then CC106. This pattern was consistent with phenotypic values of the respective accessions (Supplementary Fig. S17D, Supplementary Table S27, Supplementary Table S29).

### Pan-genome assembly, annotation, and presence–absence variation analysis

**Figure 6:**
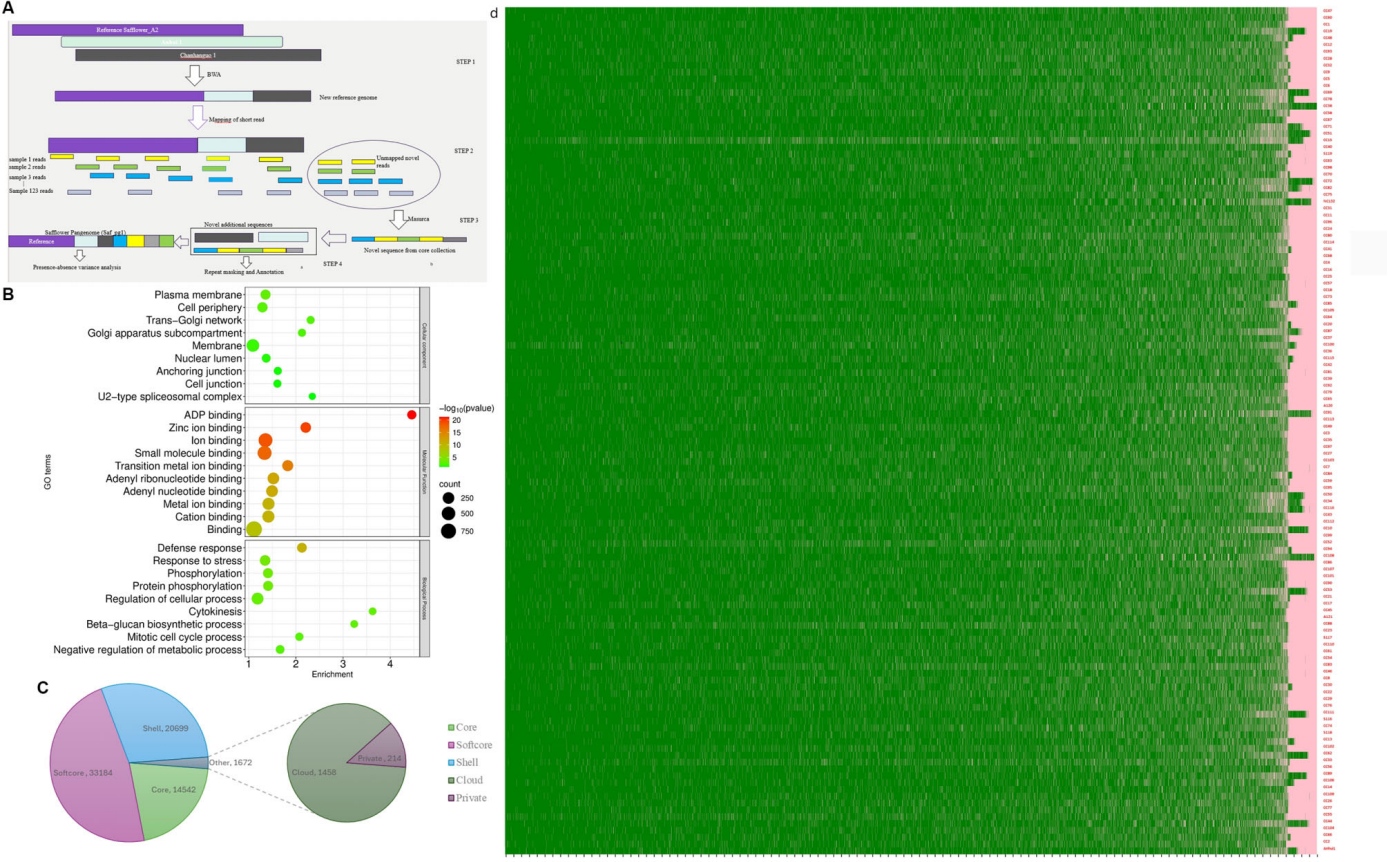
The pan-genome of safflower. (A) Schematic diagram for the construction of pan-genome. (B) Functional enrichment of the novel genes of the safflower pan-genome. (C) Classification of pan-genes based on PAV analysis. (D) PAV matrix showing the presence (green) and absence (pink) of pan-genes in the different accessions.

Using the 3 chromosomal-level safflower assemblies (this study, [14, 15]) and accessions from the core collection (Fig 6A), we assembled a pan-genome of safflower of 1.26 Gb (henceforth called Safpg_v1), including an additional 99.9 Mb, which represents an increase of 8.9% over the reference genome. This additional sequence comprised 63,814 contigs with a length range from 1 to 47.85 kb. We predicted 11,479 transcripts, which increased the total number of predicted transcripts of the safflower genome to 71,470 (size >150 bp). Functional annotation of the newly added transcripts against the RefSeq database assigned functions to ~5,383 transcripts. The enrichment of variable genes was mainly detected in the following categories: regulation of biological process, response to stimulus, catalytic and binding, and disease resistance. GO enrichment analysis of annotated novel transcripts revealed significant enrichment in biological processes related to stress response (Fig. 6B). We detected 136 and 103 enriched transcripts for biotic and abiotic stresses, respectively. Furthermore, domains were identified through InterProScan exhibiting domains related to protein kinases, zinc-finger, reverse transcriptase, disease resistance gene, HEAT repeat domains, and leucine-rich repeats. KEGG pathway analysis identified key pathways, including linoleic acid metabolism, galactose metabolism, and ABC transporter systems. These enrichment results indicated that these additional genes were largely involved in metabolic processes, helping the plant in combating biotic and abiotic stresses [42].

The breeding history and genetic changes of the crop can be revealed in the presence–absence variation (PAV) among different accessions. We identified 14,542 core, 33,184 soft core, 20,699 shell, 1,452 cloud, and 214 private genes (Fig. 6C) through the PAV matrix (Fig. 6D). All the core and soft core genes were assigned as conserved (47,726), whereas shell, cloud, and private genes were assigned as variable (22,371) genes. Modeling of the pan-genome showed that the number of core genes remained constant. However, the number of pan-genes continued to increase with the addition of the new genome, suggesting that saturation had not yet been achieved (Supplementary Fig. S18A). We detected larger gene length and more exons in the core genes as compared to variable genes, showing the conserved nature and long evolutionary history of core genes, as well as a comparatively new origin of the variable genes. The maximum number of the genes was contributed by NC132, an accession from the United States. The largest number of the variable genes was harbored by CC62, a Russian accession. The maximum number of shell genes was present in accession CC25 from India. Interestingly, an Indian accession, CC38, tends to be the most diverse accession of the core collection, with 1,326 cloud genes and 203 private genes. CC108 (United States), CC72 (Spain), CC51 (Iran), and NC132 (USA) are also diverse accessions based on cloud genes (Supplementary Table S30). The number of core and soft core genes is similar among the regional pools. The highest number of variable genes was seen in accessions of the Indian subcontinent and the United States, depicting their high genetic diversity (Supplementary Fig. S18B). Out of 103 new abiotic resistance genes, 55 were found across all 13 regional gene pools, and 43 were present in at least 11 of them. For biotic stress, 56 out of 135 enriched transcripts were seen in all 13 gene pools, while 65 were present in at least 10 gene pools (Supplementary Tables S30 and S31). These pan-genomic genes could be used for regional breeding programs.

## Discussion

In the present study, we generated an improved safflower genome assembly, which was validated with the help of a GBS-based linkage map. This reference assembly was used for calling SNPs from core collection resequencing data. GWAS, candidate gene analysis, and haplo-pheno analysis were conducted, leading to identification of genomic regions associated with important safflower agronomic traits. The resequencing data were also used to identify a pan-genome for the crop.

### Improved quality of the Safflower_A2 genome assembly

We report an improved, contiguous, and richly annotated genome sequence of a safflower accession (Safflower_A2) with high oil and nutritionally desirable high oleic acid content. The 2 earlier reported chromosomal-level genome assemblies were derived from Chinese safflower accessions, Anhui [14] and Chuanhonghua 1 [15], which were rich in the linoleic acid. *k*-mer analysis (*K* = 17) of our genome demonstrated a genome size of 1.17 Gb with low heterozygosity and high repeat content, which is in consonance with earlier studies [14, 15]. The estimated genome size through flow cytometry was 1.34 Gb, which was 8.7% higher than *k*-mer distribution analysis. A lower estimation by *k*-mer analysis could be attributed to the high amount of repeat sequences in the safflower genome [43]. The genome assembly of Safflower_A2 is 1.15 Gb, which is better than the Anhui 1 genome assembly (1.07 Gb) and similar to Chuanhonghua 1 (1.17 Gb). As mentioned earlier, the Safflower_A2 and Anhui 1 genomes revealed one-to-one alignment (Supplementary Fig. S7A). However, Safflower_A2 chromosome lengths were significantly longer than those of Anhui 1 (Supplementary Figs. S19A, S18B). The Safflower_A2 genome also showed higher completeness, with a significantly higher BUSCO score (97.9%) as compared to the Anhui 1 (90%) and Chuanhonghua 1 (89.25%) genomes. A higher rate of mapping back of raw long reads of the Safflower_A2 genome (99.29%) as compared to the Anhui 1 (98%) and Chuanhonghua 1 (93%) genomes further supports superior quality of the Safflower_A2 genome (Table 1). Although the primary genome assembly of Chuanhonghua 1 was reportedly higher at 1.17 Gb, our analysis shows the fragmented nature of this genome (explained by indices described below); hence, comparison of chromosomal lengths could not be accurately conducted as we could not correlate chromosomes between the 2 genomes (Supplementary Fig. S7B). During our structural variant analysis, we detected the presence of large translocations in the Chuanhonghua 1 genome (particularly on chromosome 1), which further suggests possible misassembly or unresolved scaffolding errors during genome construction (Supplementary Fig. S7C, D). Although such large-scale structural rearrangements could be due to true biological divergence, their absence in the Anhui 1 genome indicates that the translocations are likely assembly artifacts. We have validated our genome assembly using a high-density linkage map constructed using GBS data from a RIL population. A high concordance between the genome assembly and the linkage map confirms the accuracy and completeness of our genome assembly. As compared to earlier studies, we detected a higher number of LTRs in our genome, which is supported by a high LAI score (22.49) that is comparable to that of the Anhui 1 genome (23.08) and significantly higher than that of Chuanhonghua 1 genome (14.65; Table 1). Further, we detected telomeric repeats at both ends of 3 chromosomes and one end of 9 chromosomes, which indicates near completeness of the Safflower_A2 genome assembly. We also detected centromeric repeats of 4 different lengths (342 bp, 348 bp, 349 bp, and 350 bp) on all the chromosomes, representing resolution of the repetitive regions of the genome. Centromeric and telomeric regions are being reported for the first time in the Safflower_A2 genome. We detected 59,995 protein-coding transcripts, corresponding to 39,945 Unigene models (at 80% similarity) after clustering. A higher number of unigenes can be attributed to improved quality of the Safflower_A2 genome and use of the BRAKER3 pipeline in the current study, which outperforms MAKER2 (used in earlier studies) in the prediction of exons, genes, and transcripts [44]. Use of a comprehensive Iso-seq dataset as evidence for annotation improved the BUSCO score of predicted protein sequences (Table 1). Furthermore, our results are in consonance with the recent reports on other plant species, including *Eriobotrya japonica* [45] and *Lonicera caerulea* [39].

### Identification of genes associated with disease resistance

The generation of a repertoire of R genes and their analysis are important to facilitate breeding for resistance to biotic stresses. A comprehensive analysis of R genes in the safflower genome is lacking. We identified 228 non redundant genes, coding for 236 NLR transcripts in the safflower genome, which would expedite the cloning of resistance genes and enhance our understanding of their associated mechanisms [47]. The protein coded by an NLR gene consists of the NBS domain, which hydrolyzes energy, and the LRR domain for pathogen recognition [48]. The TIR domain is involved in the downstream signaling involving enhanced disease susceptibility 1 (EDS1) protein, providing immunity against biotic and hemi-biotic pathogens, exhibiting both local and systematic immunity. In contrast, non-TNL domains (CNL and RNL) are involved in NPR1-mediated immunity, contributing to broad-spectrum systemic immunity in plants [49]. In safflower, the ratio of TNL and non-TNL is ~4.25, suggesting the higher contribution of TNL in the disease resistance of safflower. The number of TNL-RGAs is higher than non–TNL-RGAs in safflower, which is in contrast with sunflower, wherein non-TNL genes were higher (~0.85) [48]. However, our observations are in consonance with those of *Arabidopsis* (~4.26), as observed in the earlier studies [15]. To understand the evolutionary history, we conducted a phylogenetic analysis by incorporating NLR genes from the model plant *A. thaliana* and sunflower (*H. annuus*). Phylogenetic analysis classified NLR genes into 3 distinct clades: TNL, CNL, and RNL, indicating ancient divergence and functional specialization within the NLR gene families (Fig. 3D). Among these clades, NLRs from safflower and sunflower clustered together, representing a lineage-specific expansion within the Asteraceae family. In contrast, *A. thaliana* genes were placed in a divergent clade, highlighting both conserved and divergent evolutionary patterns across species. Additionally, large branch lengths in the TNL and CNL clades across all species suggest dynamic evolution, likely driven by coevolution with pathogens. In contrast, the shorter branch lengths of the RNL genes suggest their role as helper components downstream to the immune signaling pathway [51]. Furthermore, TNL and CNL genes show early divergence, while RNL genes representing more recent divergence. The collinearity analysis revealed a higher number of colinear blocks between safflower and *Arabidopsis* as compared to safflower and sunflower, which could be attributed to the greater number of annotated R genes in the *A. thaliana* genome compared to the sunflower genome. Additionally, the TNL to non-TNL ratio in both safflower and *A. thaliana* is similar (approximately 4.2), while in sunflower, it is around 0.85. The variation in the TNL to non-TNL ratio between the safflower and sunflower genomes can be explained by a whole-genome duplication event that occurred after the divergence of the 2 species [15]. This lower ratio of TNLs in sunflower may contribute to the reduced number of colinear blocks observed.

The functional annotation of R genes in safflower has unveiled a repertoire of proteins integral to the plant’s defense mechanisms against a spectrum of pathogens (Supplementary Table S16). Notably, proteins such as TMV resistance protein-N-like, ROQ1, RPP13, RRS1, RUN1, and DSC1 have been identified to confer resistance against viral, bacterial, and fungal pathogens, including *Xanthomonas, Pseudomonas, Ralstonia, Puccinia*, and *Verticillium* species, respectively [52–60]. For instance, ROQ1 recognizes bacterial effectors like XopQ and HopQ1, activating defense responses that are crucial for resistance, as shown in crops like tomato and *Nicotiana benthamiana* [52–54]. Similarly, RPP13 has been shown to contribute to basal resistance in wheat by interacting with pathogen effectors, thereby restricting the development of diseases such as powdery mildew [55, 61]. The At4g11170 gene encodes a resistance methylated protein (RMP) that has been associated with resistance to *Fusarium oxysporum* [62], a significant pathogen in safflower cultivation. In addition to these, genes like NRG1.1 [63], RPP8 [64–66], At4g11170 [62, 67], and RGA3 [51, 68–71] have been implicated in defense against pathogens such as *Alternaria, Fusarium, Phytophthora, Potyvirus*, and *Golovinomyces*, respectively. Incorporating these R genes into safflower breeding strategies through marker-assisted selection can expedite the development of resistant cultivars. Furthermore, pyramiding multiple R genes conferring resistance to different pathogens can enhance the durability and breadth of disease resistance. This approach mitigates the risk of resistance breakdown due to pathogen evolution and provides a sustainable solution for disease management in safflower.

### Exploring the genetic basis of various agronomically important traits in safflower

To decipher the full repertoire of genes available in a crop, the availability of sequence data from diverse accessions is important. However, due to the significantly large diversity harbored by safflower, resequencing of a core collection is more practical, cost-effective, and time-saving. Thus, we resequenced a core collection comprising 123 accessions developed earlier by our group [16]. This core collection was developed through a maximization strategy from a germplasm collection of 531 accessions representing the global genetic, morphological, and geographical diversity available for safflower. In the earlier study by [15], a collection of 220 accessions was resequenced. However, this collection mainly consisted of accessions of Chinese origin (149), with a limited representation of global genetic diversity. Through ADMIXTURE analysis, we predicted 4 hypothetical subpopulations and identified 19 admixtures in our population. Our earlier study [72], based on SSR data, indicated 2 clusters (highest peak at *K* = 2 and a smaller peak at *K* = 4) and 16 admixtures. Use of a larger SNP dataset in the current study increased the resolution. We further inferred genetic relationships among accessions using distance-based methods, NJ and PCA, and observed its concordance with Bayesian-based ADMIXTURE methods. All accessions from ADI clustered together in quadrants 2 and 3 of PCA. Quadrant 3 comprised accessions from the United States, while quadrant 2 consisted of accessions from other regional gene pools. All accessions from ADIII clustered in quadrant 4, whereas ADII and ADIV accessions were clustered in quadrant 1. Most ADI accessions clustered together in NJI. ADII accessions, along with some ADI accessions, were in NJII. NJIII comprised accessions from ADIII, while NJIV had accessions from ADIV and ADI. We observed a lack of geographical structuring among accessions of the core collection, which could be attributed to the maximization strategy used for core collection development, emphasizing allelic diversity with minimum redundancy [16]. Further, a low F_st_ and kinship value between the subpopulations represents the low differentiation and low genetic similarity between the subpopulations. Thus, low molecular relatedness and weak population structure among the core collection accessions reduce the likelihood of false marker–trait associations [49, 50], affirming its appropriateness for association mapping.

GWAS is a powerful tool in crop genetics, providing comprehensive insights into the genetic basis of complex traits, accelerating breeding programs by eliminating the need for developing biparental populations [73]. In the current study, 5 different models were tested, and 3 multilocus models (MLMM, Fixed and random model Circulating Probability Unification [FarmCPU], and Bayesian-information and Linkage-disequilibrium Iteratively Nested Keyway [BLINK]) were utilized to detect significant MTAs. The multilocus models demonstrate robustness as they consider multiple loci, hence reducing false positives. In our data, multilocus models fared better than single-locus models, as indicated by Q-Q plots. MLMM is effective in identifying large effect loci, while FarmCPU can detect small effect loci [74, 75]. Although FarmCPU has a reduced rate of false positives compared with MLMM, it is still prone to errors. In comparison, BLINK includes LD information in the analysis [76] and hence performs better for environmentally sensitive traits, as we observed in HN, PB, and 100SW. Since all 3 models have their strengths and weaknesses, we analyzed Q-Q plots to ensure that the model used is not overfitting and an appropriate model has been used (explained in Results section). Further, we ensured the robustness of markers by comparing the analysis of 2 seasons of data and retaining markers that were identified in both seasons. We report a total of 96 QTNs, wherein 22 QTNs were identified for OC, 7 for PH, 16 for OA and LA, 21 for SW, 4 for HN, 14 for PB, and 12 for DTF (Supplementary Table S23). In a previous study [15], QTNs have been reported for oil content, flower color, ball (head) number, branch height, bract spine, first branch number, plant height, and stem diameter. While QTNs reported in the study show strong correlations with traits, an efficient mixed-model association (EMMA) model for the detection of MTAs is computationally less robust compared to MLMM, FarmCPU, and BLINK used in the current study, especially for large datasets. Thus, by leveraging a multimodel GWAS approach, phenotypic data from 2 consecutive growing seasons, and a globally diverse germplasm collection, our study provides more robust QTNs. The QTN-marked regions were subjected to candidate gene analysis, identifying genes invaluable for breeding programs that could lead to developing varieties with improved oil content and cultivars optimized for specific growing environments. We identified several candidate genes associated with key agronomic traits, including OC, PH, DTF, OA, LA, 100SW, PB, and HN, as detailed in Table 2. These candidate genes could also be used for genome editing approaches, although further validation is required. Similar approaches have been employed in apple [77], rice [78], and soybean [79]. To increase the confidence in the identified candidate genes, we performed haplotype analysis. A similar approach has been employed for the detection of haplotypes associated with various agronomically important traits [80, 81]. We detected robust haplotypes exhibiting significant variation in the core collection for OC, SW, DTF, PB, OA, and LA. However, only 1 or 2 major haplotypes were identified for PH and HN, highlighting their complexity due to strong interactions with environmental conditions. We observed many SNPs (ranging from 11–40) within each haplotype. The number of haplotypes ranged from 11 to 83, with OC, DTF, SW, OA, and LA having more than 50 haplotypes, indicating the presence of diverse SNP combinations in germplasm. Interestingly, we found that putative candidate genes were in proximity (within 2 Kb) of the haplotypes, suggesting that these SNP groups may play crucial roles in gene function. In this study, we identified favorable haplotypes, which are prevalent in large populations and encode a wide range of trait values and likely selected through evolution [82, 83]. However, we also detected superior rare haplotypes, contributing to exceptional agronomical trait values. For superior OC, we identified 3 accessions (CC106, CC101, and CC090) characterized by high oil content coupled with superior alleles for OA or LA. However, these accessions had favorable haplotypes for other traits. The information generated can be used to create varieties with multiple superior haplotypes using approaches like pyramid breeding [84].

In crop improvement programs, functional markers (FMs) are instrumental in enabling precise selection for desirable traits. The development of high-throughput and cost-effective genotyping platforms, such as KASP, has significantly enhanced the application of marker-assisted selection by allowing rapid screening of large breeding populations. In this study, we developed KASP assays targeting 20 SNP loci (Supplementary Tables S25, S26) associated with 7 key agronomic traits. Based on phenotypic trait values, panels were constructed, and marker functionality was assessed across diverse genetic backgrounds. Among the 20 SNPs developed and validated in the present study, 10 were QTNs, including traits such as OC (OC1, OC8, OC12), OA and LA (OA_LA17), 100SW (SW_23), DTF (DTF2), PH (PH_4), and PB (PB14, PB18). Furthermore, 10 SNPs were selected based on their proximity to candidate genes involved in trait regulation, including those associated with oil content (4), fatty acid content (2), seed weight (3), and flowering time (1). Our study developed a KASP assay for the SNPs closer to genes (Supplementary Table S26) known to play an important role in various agronomic traits. These KASP markers provide a rapid and reliable tool for marker-assisted selection, enabling efficient integration of favorable alleles into elite cultivars. The validation of representative QTNs and their associated SNPs with candidate genes reinforces the reliability and robustness of our bioinformatic dataset.

Further, expression profiling of candidate genes for post harvest seed-related traits (OC, OA-LA, and SW) revealed strong genotype and stage-specific patterns. Gene *g16872* (myosin-binding protein 2, OC) showed strong induction at 10 DAP in CC106, consistent with its high oil content, suggesting a role in lipid accumulation during the early seed-filling stage. *MYOB2* were localized to lipid droplets in *Arabidopsis* and are part of the cytoskeletal structure involved in lipid mobilization [27]. Gene *g43426* (cytochrome P450, OA-LA) exhibited stage-specific upregulation at 10 DAP in LA-rich genotypes (i.e., CC113 and S116), aligning with its putative function in fatty acid modification. Gene cytochrome P450 has been indicated to play a role in fatty acid modification [33, 34]. For SW, *g57921* (FRIGIDA-ESSENTIAL 1-like isoform) showed increasingly higher expression at 20 DAP among 3 genotypes tested, which is in agreement with increasing seed weight (S116 > CC113 > CC106). A FRIGIDA-like protein has been reported by [85] as a candidate for seed weight in chickpea. Our RT-PCR results validate the functional relevance of genes identified in the present study.

The present integrative bioinformatic analyses identified key candidate genes, laying the groundwork for future experimental studies. Future studies should aim to further characterize identified genes for their functional role through approaches like genome editing, gene knockout, overexpression, or allelic function analysis. Nonetheless, the information generated in current analysis is invaluable for safflower breeding programs.

### Pan-genome analysis reveals distinct functional enrichments among pan-genes

A single reference genome cannot encompass the entire genetic variability present in a species like *C. tinctorius*, which has gone through extensive diversification [86]. Pan-genomes developed from diverse individuals act as an important resource to capture the available genetic variability and mining of alleles. In recent years, pan-genomes have been constructed for various plant species [86–89]. We constructed the pan-genome of safflower using unmapped reads of 123 accessions of our core collection through an iterative mapping and assembly approach. The newly assembled sequence includes those regions absent in the reference genome and thus acts as an extended repertoire of available genes for the crop. GO enrichment analysis of annotated novel transcripts revealed significant enrichment in biological processes related to stress response. Abiotic stress–related gene (103 transcripts) included peroxidases, DELLA proteins, DNA repair proteins, E3 ubiquitin-protein ligases, heat shock proteins, and TIFY transcription factors, indicating their potential role in enhancing safflower’s tolerance to environmental stresses. In contrast, transcripts associated with biotic stress were found to encode a range of resistance proteins, including RML1A-like, RGA-3, RPP-13, RGA-4, R1A-10, R1B-14, RML1B-like, thaumatin-like proteins, BTB/POZ domain proteins, ankyrin repeat-containing proteins, and TMV-resistance genes. Identification of thaumatin-like proteins [90–92] is crucial, as it has been linked to resistance against Fusarium wilt, which is a major disease for safflower (Supplementary Table S16). In terms of molecular function, the novel transcripts were predominantly enriched in binding activities, especially ion binding, small-molecule binding, and nucleic acid binding. Cellular component analysis indicated their localization to membrane-associated structures such as the plasma membrane, nuclear lumen, cell junctions, and the U2-type spliceosome complex. Domain analysis further revealed features such as protein kinases, LRR domains, P-loop NTPase, zinc-finger motifs, and HEAT repeat domains, suggesting roles in signal transduction and stress response. Enriched domains like GIP-1 (G-protein interacting protein), heavy metal–associated motifs, transient receptor potential channels, and lipoxygenase PLAT domains highlight potential involvement in detoxification, ion transport, and lipid metabolism under stress [35].

We performed PAV analysis using the map-to-pan approach and identified core genes that are common across multiple genomes and define the species, as well as variable genes found in only a few genomes, which contribute to the unique characteristics of each genome. We further analyzed core and variable genes among regional gene pools. The regional gene pools of safflower (i.e., United States, India, Far East, and Europe) comprise accessions (Supplementary Tables S31 and S32) with large numbers of variable genes and thus exhibit high genetic diversity that might have arisen during diversification of safflower. In our study, the Indian accession, CC38, was found to be the most diverse accession of the core collection, consisting of a large number of cloud and private genes. PAV analysis of stress-related transcripts showed peroxidases and heat shock proteins common across accessions and gene pools. However, genes like TIFY-8 and WD repeat-containing protein 76 were present in only 30 and 83 accessions, respectively. Among all the accessions, distribution of stress-related genes showed 96 genes in CC30 (Indian subcontinent), 94 genes in CC47 (Iran–Afghanistan), and 91 genes in CC62 (Europe), suggesting that these gene pools are most diverse (Supplementary Table S32). The US accession CC86, with only 64 genes, indicated that this gene pool is the least diverse. For biotic stress, TMV resistance protein N-like, L-type lectin-domain containing receptor kinase S, and DSC were found in most accessions. The Indian subcontinent accession (CC56) showed the highest number of biotic resistance genes, while the US accession carried the fewest. Overall, the inclusion of novel gene content from the safflower pan-genome highlights substantial genetic diversity, which likely contributed to the species’ adaptive capacity, providing resources for regional breeding programs.

## Methods

### Plant material

For generating the genome assembly, a safflower accession from the United States (PI:560169; USDA; named “Safflower_A2”), attributed with substantially high seed oil content (~47%) and naturally enriched with high oleic acid (~87%), was selected. The safflower core collection reported earlier by our group [16], comprising 116 globally distributed accessions and 7 additional accessions (Supplementary Table S17) with agronomically important traits, was subjected to Illumina-based resequencing (RRID:SCR_010233) (~15× coverage) using the NovaSeq 6000 platform.

### Genome sequencing and assembly

HiFi long-read sequencing was performed on PacBio Sequel II platform (RRID:SCR_017990), following the manufacturer’s instructions (PacBio). A Bionano Saphyr chip (Bionano Genomics [RRID:SCR_017992]) was used for generating optical mapping data. The Proximo Hi-C (Plant) Kit Protocol (Phase Genomics) was used to construct the Hi-C library (Phase Genomics). The Hi-C libraries were sequenced on an Illumina NovaSeq 6000.

Genome size was estimated through 2 approaches: flow cytometry and *k*-mer frequency distribution. For flow cytometry, the CyStain PI Absolute P kit (Sysmex) was used for sample preparation following the manufacturer’s instructions. A minimum of 5,000 stained nuclei per sample were evaluated on CyFlow Cube 8 flow cytometer (Sysmex) using tomato “Stupicke” polní rané as a reference [93]. *k*-mer–based genome size estimation was performed using clean HiFi reads from PacBio SMRT sequencing, applying Kmerfreq [94] and GCE v1.02 [95].

Long PacBio HiFi reads were assembled into contigs using Hifiasm v.0.16 (RRID:SCR_021069) [96]. Due to the homozygous nature of the safflower genome, purging was disabled (-l0), and other parameters were applied at default settings. Using optical maps and contig-level assembly, hybrid scaffolding was performed using Bionano Solve v3.6 with default parameters. Scaffold-level assembly was polished using Illumina paired-end short reads through NextPolish v1.4.1 (RRID:SCR_025232) [97]. For construction of pseudochromosomes, hybrid scaffolds were linked using paired-end Hi-C reads with SALSA (RRID:SCR_022013) [98] at default settings. An additional 2 rounds of polishing were done using Pilon (RRID:SCR_014731) [99]. Finally, Hi-C raw reads were aligned to pseudochromosomes through BWA2 [100] to generate the Hi-C heatmap using PretextMap [101] and Juicer (RRID:SCR_017226) [102] for visualization, manual correction, and generation of the final chromosomal-level genome assembly. Additionally, we also assembled the Chloroplast genome using Illumina short reads by Navoplasty [103], where NC_030783.1 [75] was used as a reference.

The genome assembly was validated for its integrity and completeness. Sequencing reads from Illumina and PacBio HiFi were mapped back to the assembly using BWA-mem2 v2.2 (RRID:SCR_016662) and minimap2 v2.24 (RRID:SCR_018550) [104], respectively. For estimation of base-level accuracy, Merqury v1.3 [105] was applied. BUSCO v5.6.0 (RRID:SCR_015008) [106] analysis was implemented to assess completeness of the genome. LTR_retreiver v2.9.8 [107] was used for estimation of the LAI score. Telomeres were detected using the Telomere identification toolkit v0.2.41 (TIDK) [108]. Centromeric repeats were detected using TRASH [109].

The present genome assembly was aligned with earlier published safflower genomes [14, 15] using D-genies (RRID:SCR_018967) [110]. We further identified structural variants (deletion, insertion, translocations). First, high-quality PacBio HiFi reads from the the Safflower_A2 genotype were aligned to Anhui 1 and Chuanhongua 1 genome assemblies using minimap2 v2.24 [104] with parameters (-ax map-hifi). The resulting BAM files were sorted and indexed using SAMtools v1.15.1 (RRID:SCR_002105) [111]. SVIM v2.0.0 [112] was then run in default mode to call structural variants from each alignment. Only high-confidence variants greater than 1 Kb were used for downstream analysis. SyntenyplotR [113] and KaryoplotteR [114] were used for the visualization of the genome alignment maps.

### Construction of a high-density genetic linkage map and chromosomal assignment

A RIL population comprising 121 individuals (designated as “population A”; F_8_) was developed by crossing parents A1 (PI:537110) and A2 (PI:560169). The GBS library was prepared following [115] using a combination of *Mse*I and *Hae*II enzymes. Libraries were sequenced on the Illumina NovaSeq 6000 platform (RRID:SCR_016387). Filtered reads were aligned to the Safflower_A2 genome, and SNPs were called (Supplementary Fig. S3b). SNPs were filtered using criteria summarized in Supplementary Table S6. A high-density linkage map was constructed via JoinMap v4.1 using the Kosambi mapping function at logarithm of the odds (LOD) score 20, followed by marker order correction and calculation of genetic distances using R/ASMap [116] at LOD 4. The linkage map was utilized for evaluating and anchoring of chromosomes using ALLMAPS [117] (RRID:SCR_021171).

### Full-length transcriptome sequencing and detection of alternate splicing events

Iso-seq sequencing was performed on RNA from 8 samples (shoots, seedling-roots, leaves, flowers and buds, and seed developmental stages [at 5 DAP, 10 DAP, 20 DAP, and 30 DAP]). Size-selected SMRTbell libraries were sequenced on a PacBio Sequel II platform (RRID:SCR_017990). Raw data were processed via SMRTlink v9.0 to generate CCS using default parameters. IsoSeq v3 pipeline [118] (RRID:SCR_022749) was used to obtain full-length transcripts, which were collapsed into transcript clusters using pbcluster. pbmm2 [119] (RRID:SCR_025549) was used to map FL-transcripts to the repeat-masked Safflower_A2 genome using parameters –preset ISOSEQ –sort. Splicing patterns of FL transcripts were analyzed using SUPPA2 [120, 121].

### Annotation of repeatome, gene prediction, and functional annotation of protein-coding genes

Chromosomal-level genome assembly of the Safflower_A2 was subjected to an Extensive De novo TE Annotator tool (EDTA v2.0.0) (RRID:SCR_022063) [122] (using parameters –anno 1, –sensitive 1) for the identification of safflower-specific *de novo* TE libraries. The in-built tools within the EDTAv2 pipelines, including Repeat modeller, LTR_FINDER, LTRharvest, LTR_retreiver (RRID:SCR_017623), HelitronScanner, MITE hunters (RRID:SCR_020946), and TIR-Learner, were used for the annotation of the transposable elements in the safflower genomes using Repbase (v20181026) libraries (RRID:SCR_021169) [122]. MISA v1.0 (RRID:SCR_010765) was used for the identification of the SSRs in our genome. Identified repeats were masked using RepeatMasker v4.1.7 (RRID:SCR_012954) [123]. The classification of LTR retrotransposons (LTR-TEs) was carried out using the DANTE v0.2.5 pipeline [124]. This tool extracts information from the Viridiplantae data in the Rexdb database. Additionally, we employed DANTE_LTR [124] to identify and classify LTR-TEs as complete/autonomous (i.e., consisting of complete machinery for transposition). An LTR was classified as autonomous if it contained all necessary domains, including reverse transcriptase (RT), capsid-related domain (GAG), RNase H (RH), protease (PROT), and integrase (INT), whereas it was called complete if it contained target site duplications (TSDs) and the primer-binding site (PBS), along with the necessary domains. For phylogenetic analysis, amino acid sequences of the identified complete *Copia* and *Gypsy* elements were extracted to generate a multiple-sequence alignment using MAFFT (RRID:SCR_011811) [125]. A phylogenetic tree was subsequently constructed with iqtree2 v2.3.0. The insertion times of the complete LTRs were estimated using LTR_retriever v2.9.8 (RRID:SCR_017623) [126].

The identification of noncoding RNAs, including rRNA and tRNA, was performed in the safflower genome assembly. Predictions of rRNA genes were conducted using barrnap v0.9 (RRID:SCR_015995), applying specific parameters for eukaryotic genomes (–kingdom euk) [127]. For tRNA gene predictions, we utilized tRNAscan-SE v2.0 (RRID:SCR_010835) [128].

The masked genome was used for gene prediction using BRAKER3 v3.0.4 (RRID:SCR_018964) [44]. Mapping data generated by pbmm2 (as described above) was used as a training set for *ab initio* gene finders, AUGUSTUS (RRID:SCR_008417) [120] and GeneMark (RRID:SCR_011930) [129], for gene prediction. Transcripts <150 bases and protein sequences with <50 amino acids were removed. Subsequently, transcripts were filtered using BEDtools intersect v2.21.0 BEDTools (RRID:SCR_006646) to remove those that showed a continuous repeat coverage of ≥30%. To identify the number of unigenes, CD-HIT v4.8.1 (RRID:SCR_007105) [130] was employed using parameters -c 0.8 -n 5 -M 16000. BUSCO (RRID:SCR_015008) analysis was performed to assess completeness of filtered gene sets. Gene models were subjected to functional annotation using public nucleotide and protein databases in OmicsBox v3.1.2 (RRID:SCR_023676) [131]. Homology searches were conducted against the NCBI-RefSeq database using BLASTp (RRID:SCR_004870) with a threshold e-value of 1 × 10^−3^ against GO, EggNOG mapper v5 (RRID:SCR_021165), and the KEGG expression (RRID:SCR_012773) database [132]. The motifs and domain-based functional annotation and identification of conserved domains and families in protein-coding genes were implemented using all public databases in InterProScan v5.6 (RRID:SCR_005829) [133]. Transcription factors/regulators and protein kinases were identified using iTAK v1.6 [134] with default parameters.

### Identification of RGAs

RGAs were identified using the Disease Resistance Analysis and Gene Orthology (DRAGO2) pipeline [135]. DRAGO2 classifies RGAs into coiled-coil (CC), receptor-like kinases (RLKs), receptor-like proteins (RLPs), nucleotide binding-site leucine-rich repeats (NBS-LRR), and others. Based on domain structures, NBS-LRR were further classified into CNL and TNL. HMMER (RRID:SCR_005305) software was used to classify NBS-LRR genes using the NB-ARC profile (PF00931) for the NBS domain [136]. NBS-LRR sequences were retrieved from NCBI, and BLASTp was performed. Common candidate genes identified from 3 analyses (HMMER, DRAGO2, and BLASTp) were retained for downstream analysis. PFAM [137] and the Conserved Domains Database (CDD) [138] were used for functional domain annotation. Chromosome-wide distribution of NLRs was investigated using GFF files. To analyze NLRs under selection pressure, the nonsynonymous substitution to synonymous substitution (Ka/Ks) ratio was calculated using KaKs calculator 2.0 [134]. For inferring the evolutionary/phylogenetic history, NLR genes were also identified from the genome of *H. annuus* (OXS) [24] and *A. thaliana* [23]. Protein sequences were aligned using MAFFT (RRID:SCR_011811) [125] (–localpair –maxiterate 16 –reorder). A phylogenetic tree was constructed using IQ-TREE v2.0.6 (RRID:SCR_017254) [139] with maximum likelihood (ML) using 1,000 ultra bootstrap replicates. The visualization of the tree was done using Interactive Tree of Life (iTOL) v5 (RRID:SCR_018174). Colinear genes and syntenic blocks among the safflower and sunflower genomes, as well as the safflower and *Arabidopsis* genome, were identified using MCScanX (RRID:SCR_022067) (-s 3 -b 2 -w 2 -e 10e-3 -k 40) [77].

### Exploring the genetic basis of safflower for various agronomically important traits

Genomic DNA was sequenced on the Illumina NovaSeq 6000 platform (RRID:SCR_016387) to generate 150-bp paired-end reads. Reads were cleaned and mapped to the Safflower_A2 genome using BWA-mem v0.7.17 [83]. GATK v4.4.0 (RRID:SCR_001876) [84] was used for variant calling with the following parameters: –minimum-mapping-quality 20, –min-base-quality-score 20. Hard filtering was applied based on GATK best practices recommendations [140], followed by filtration using VCFtools v0.1.15 (RRID:SCR_001235) (Supplementary Table S18).

Fast Tree v2.1.10 [86] with the GTR model was used for construction of the phylogenetic tree using filtered SNPs and visualized using iTOL v5 [87]. The genetic structure of the core collection was assessed using ADMIXTURE v1.3.0 9 (RRID:SCR_001263) [88]. Number of clusters (*K*) was inferred based on lowest coefficient of variation error. PCA was performed using PLINK v1.90b4.6 (RRID:SCR_001757) [89] with default parameters. The first 2 eigenvectors showing maximum variability were plotted using R. Pairwise F_st_ between subpopulations inferred from ADMIXTURE (*K* = 4) were calculated using VCFtools [90] (RRID:SCR_001235). PopLD decay [91] with default settings was used for estimation of LD in safflower.

Phenotypic data described earlier [16] for 8 traits, including PH, HN, PB, DTF, OC, 100SW, OA, and LA content, from 2 independent growing seasons (2011–12 and 2012–13) were used. SNPs generated in the present study were filtered using TASSEL v5 (RRID:SCR_012837) [141]. GWAS analysis was conducted for data of 2 seasons independently using 2 single-locus models (general linear model [GLM] and mixed linear model [MLM]) [142] and 3 multilocus models (MLMM [74], FarmCPU [30], and BLINK [76]). These models were implemented using GAPIT v3 [143] in R programming software, assigning a PCA value of 4 (PCA.total = 4) based on admixture analysis. GAPITv3 uses the Benjamini–Hochberg method, which is well known to control the false discovery rate (FDR). MTAs were considered significant at *P* < 0.0001. MTAs that followed specific criteria were retained and classified as QTNs. For traits OC, PH, and DTF, the QTNs were consistently identified across all 3 multilocus models in both growing seasons. For traits HN, PB, and 100SW, which showed more seasonal variability, QTNs were defined as those present in at least 1 multilocus model and both growing seasons.

QTNs identified for 8 traits were used for candidate gene analysis (CGA). LD block analysis was conducted using LDBlockShow [144] to define the region for CGA. Candidate genes were subsequently searched within a 7-kb region upstream and downstream of the QTNs. To verify the association of candidate genes with traits, putative candidates were screened based on their annotated functions. Genes associated with metabolic pathways, stress responses, and traits such as oil biosynthesis, plant architecture, and flowering time were selected for haplo-pheno analysis. The GenhapR [82] package was utilized to detect haplotypes within the LD regions of candidate genes. Association analysis was conducted for haplotypes represented in 3 or more individuals in the core collection, while other haplotypes were classified as rare. Haplotypes with high average values and present in a large number of accessions were designated as favorable haplotypes. The haplotypes found in accessions exhibiting the highest trait values were identified as superior haplotypes.

Candidate genes with significant functional associations to the traits were further analyzed by searching the 2 kb upstream and downstream to identify the SNPs in the vicinity of the genes. Following manual curation, 20 SNP sites associated with agronomic traits were selected for validation using the KASP assay on an appropriate panel representing high and low trait values. A total of 46 accessions were used in different panels (Supplementary Table S26). High-quality genomic DNA was extracted from these accessions using the HiPurA Plant Genomic DNA Miniprep Purification Kit (Cat. No. MB507). The primers were designed using the Web-based Allele-Specific Primer design tool (WASP) [145], and the assay was designed using the web-based tool PrimerDigital [146]. PCR reactions were performed as per the user manual (LGC Genomics). The plate was read with the FRET-capable plate reader (Victor X3; PerkinElmer) with the relevant filter sets for fluorescence detection. Fluorescence data were analyzed using KlusterCaller software (version 3.4.1.36; LGC Genomics), and genotypes were assigned based on the clustering of allele-specific fluorescence signals.

### qRT-PCR validation of candidate genes for OC, OA-LA, and SW

Candidate genes associated with post harvest traits (OC, OA-LA, and SW) were prioritized for expression profiling. Genes localized near QTNs exhibiting robust *P* values were selected (detailed in Results) (Table 2, Supplementary Table S23). Further, the expression profiles of these genes were assessed with the published RNA-seq dataset [14].

Primer sequences of selected candidate genes were designed using NCBI Primer-BLAST (RRID:SCR_003095) (Supplementary Table S28). Developing seeds were harvested at 5 DAP, 10 DAP, 20 DAP, and 30 DAP from 3 contrasting safflower accessions: S116, CC106, and CC113 (Supplementary Table S30). Total RNA was isolated using the PureLink RNA Mini Kit (Invitrogen) according to the manufacturer’s protocol. Approximately 1.5 µg DNase-treated RNA was used for first-strand cDNA synthesis using the iScript cDNA Synthesis Kit (Bio-Rad) in a total reaction volume of 10 µL. The qRT-PCR was performed using the CFX Connect Real-Time PCR Detection System (Bio-Rad). Each reaction was set up in a total volume of 10 µL containing 1 µL diluted cDNA (1:10) template, 2.5 µL PowerUP SYBR Green Master Mix (Applied Biosystems), and 0.5 µM of each forward and reverse primer. Three biological replicates were analyzed for each sample. Eukaryotic initiation factor (*EIF*) gene was used as an internal control for normalization [147, 148]. Relative expression levels were calculated using the 2^⁻ΔΔCT^ method [149], using CC106_5DAP as the calibrator sample. Statistical significance of expression differences across developmental stages was determined using the Student *t*-test.

### Pan-genome assembly, annotation, and PAV analysis

A pan-genome was constructed through an iterative mapping and assembly approach using sequencing data of the core collection (Illumina short reads, 123 samples) and available chromosomal-level assemblies of safflower [14, 15]. We constructed the pan-genome assembly in 4 major steps detailed as follows (Fig. 6A). In step 1, the chromosomal-level assemblies of safflower (Anhui 1 and Chaunhangua 1) were iteratively mapped to the Safflower_A2 reference sequence using Minimap2 (RRID:SCR_018550), and identified novel segments were integrated into the reference genome. In step 2, filtered Illumina sequencing reads from the core collection were mapped to the reference genome using BWA-mem2 v0.7.17 (RRID:SCR_022192). In step 3, unmapped and discordant reads were extracted using SAMtools view v1.20 (-f4, -f8 and -f12) (RRID:SCR_002105) and assembled *de novo* using MaSurca v3.2.3 (RRID:SCR_010691) [150] with default settings (SOAP_ASSEMBLY = 0, close gap = 1). Contaminated reads from nonplants in the resultant contigs were identified using BLASTn (RRID:SCR_004870) (e-value = 1 × 10^−5^) against the NCBI-NR and RefSeq (RRID:SCR_003496) databases and discarded from further analyses. To eliminate any other potential contamination, contigs were screened using NCBI-FCS (RRID:SCR_026367) [151], and contigs containing nonplant sequences were removed. In step 4, all the novel sequences (Safflower_A2) were further assembled, resulting in novel additional sequences. Repeat regions were identified using EDTA v2.0.0 [122] with the parameters –anno 1 and –sensitive 1. The genome was then masked using RepeatMasker v4.1.7 [123] (RRID:SCR_012954), which utilizes the Repbase (v20181026) library. The masked genome was subsequently used for gene prediction with BRAKER v3.0.4. For homology-based gene prediction, protein sequences of *A. thaliana, H. annus, Lactuca sativa, Cyanara cardunculus*, and *C. tinctorius* were downloaded from RefSeq (RRID:SCR_003496) and Swiss-prot (RRID:SCR_021164) databases and were used as a hint. Predicted genes were clustered using CD-HIT v4.8.1 [152], and redundancy was removed. Genes intersecting with repeat regions (>30%) were removed using BEDtools intersect v2.21.0 BEDTools (RRID:SCR_006646) [153]. The genes retained from the above steps were aligned to the Safflower_A2 genome, followed by the removal of genes showing high similarity (perc_identity = 0.8 and query_cov = 0.8). The remaining genes were considered pan-genes and used for downstream analyses. Proteins encoded by the pan-genes were subjected to functional annotation using OmicsBox v3.1.2 [131]. Functional enrichment was performed using the Database for Annotation, Visualization, and Integrated Discovery (DAVID) (RRID:SCR_001881) [154]. Contigs from the above assembly were concatenated with the Safflower_A2 genome to construct a pan-genome (named Safpg_v1). PAV analysis was performed by aligning raw reads of genomes to genic sequences of Safpg_v1 using bowtie2 (RRID:SCR_016368) (–no-mixed, –local). Genes were considered present if 80% of the gene was covered by the reads with a minimum depth of 3, else marked as absent. Based on the presence of a gene in the accessions, it was assigned as core (≥97%), soft core (90–96%), shell (15–89%), cloud (<15%), or private (only in 1 accession). To check whether a pan-genome was saturated or not, core genome size and pan-genome size were fitted using the nls function in R (RRID:SCR_001905).

## Supplementary Material

giaf151_Supplemental_File

giaf151_Authors_Response_To_Reviewer_Comments

giaf151_Authors_Response_To_Reviewer_Comments_Original_Submission

giaf151_GIGA-D-25-00014_Original_Submission

giaf151_GIGA-D-25-00014_Revision_1

giaf151_GIGA-D-25-00014_Revision_2

giaf151_Reviewer_1_Report_Original_Submission Zhihua Wu -- 2/21/2025

giaf151_Reviewer_1_Report_Revision_1 Zhihua Wu -- 7/12/2025

giaf151_Reviewer_2_Report_Original_Submission Cheng Dai -- 3/9/2025

giaf151_Reviewer_2_Report_Revision_1 Cheng Dai -- 6/29/2025

## Data Availability

The raw sequencing data and genome assembly generated during this study have been deposited at NCBI under the BioProject PRJNA1089929. Genome assembly, functional annotation, protein, and transcript sequence files of the pan-genome assembly and its annotation and PAV matrix are available at the Safflower Genome Resource (SGR) [155]. All additional supporting data are available in the *GigaScience* repository, GigaDB [156].

## Additional Files

**Supplementary Fig. S1**. Phenotype of Safflower accession “A2” (PI:560169) used in this study. (A) At flowering stage. (B) Safflower_A2 seeds.

**Supplementary Fig. S2**. Estimation of Safflower_A2 genome size (A) using flow cytometry analysis, *Solanum lycopersicum* L. “Stupicke” polní rané (reference; coefficient of variation 4.61%) used as a control. (B) *Carthamus tinctorius* L. (A2; coefficient of variation 5.23%). (C) Calculation of Safflower_A2 genome size based on average peak mean positions of A2 versus reference. (D) *k*-mer–based genome size estimation displaying 17-mer depth distribution in Safflower_A2 derived using error-corrected CCS reads from PacBio HiFi reads.

**Supplementary Fig. S3**. Workflow of genome assembly, curation, and annotation for Safflower_A2. (A) Chromosome-level genome assembly was constructed using a combination of PacBio HiFi reads, Bionano optical mapping, Illumina reads, and Hi-C. (B) Genome assembly was error-corrected using a high-density linkage map constructed from GBS data of RIL population A. (C) Manually curated and error-corrected genome assembly was subjected to repeat masking, followed by gene prediction using high-quality transcripts from the Iso-Seq pipeline as evidence, and finally functional annotation was carried out, utilizing various public nucleotide and protein databases.

**Supplementary Fig. S4**. Hi-C interaction map of chromosome-level assembly of Safflower_A2.

**Supplementary Fig. S5**. Telomeres and centromeres of the safflower genome. The x-axis represents the length of chromosomes (Mb); the y-axis represents the count of the telomeric or centromeric repeats in the genome. Color code: purple: telomeric repeats; red: CEN repeats of 342 bp; green: CEN repeats of 348 bp; orange: CEN repeats of 349 bp; and blue: CEN repeats of 350 bp.

**Supplementary Fig. S6**. Fully annotated chloroplast genome from Safflower_A2 assembled using Illumina paired-end reads.

**Supplementary Fig. S7**. Comparison of the Safflower_A2 genome assembly with earlier assemblies. (A) Dot plot showing one-to-one alignment between A2 genome assembly and Anhui 1 genome assembly. (B) Dot plot showing alignment between A2 genome assembly and Chuanhanghua 1 genome assembly. Translocation events detected in the (C) Chuanhanghua 1 and (D) Anhui 1 genome. (E) Statistics of structural variants, including deletions (DEL), inversion (INV), duplication (DUP:INT), and tandem repeats duplications (DUP:INV) detected in this study. Chromosomal location of the structural variants in (F) Anhui 1 and (G) Chuanhanghua 1.

**Supplementary Fig. S8**. SNP-based high-density linkage map of safflower. Colors indicate marker density.

**Supplementary Fig. S9**. Alignment of the genome assembly of Safflower_A2 with the high-density linkage map constructed using GBS data for RIL population A.

**Supplementary Fig. S10**. Characteristics of the repetitive elements in the Safflower_A2 genome. (A) Proportion of the repetitive element in the safflower genome. (B) Relative proportion of different classes of repetitive elements in the safflower genome.

**Supplementary Fig. S11**. Analysis of complete LTR-TE in the safflower genome. (A) Different families of the LTR-RT with the associated domains detected in this study. (B) Distribution of the different families of LTR-RT based on the presence/absence of PBS and TSD. Phylogenetic tree of (C) Ty3/G*ypsy* elements and (D) Ty1/*Copia*. (E) Insertion time of the different classes of LTR-RT.

**Supplementary Fig. S12**. Distribution of the NLR genes on the safflower genome. Red bars represent the TNL genes, black bars represent CNL genes and green bar represents the RNL genes.

**Supplementary Fig. S13**. Collinear blocks detected between (A) safflower and *Arabidopsis thaliana* and (B) safflower and *Helianthus annus*.

**Supplementary Fig. S14**. (A) Principal component analysis showing distribution of the accession using PC1 and PC2. The circle within the quadrant represents the distribution of the admixture clusters within the PCA. (B) Linkage disequilibrium along the 12 chromosomes.

**Supplementary Fig. S15**. Haplo-pheno analysis for agronomically important traits depicting QTNs for (A–C) oil content (OC), (D–G) 100-seed weight (SW), (H–K) oleic acid and linoleic acid (OA-LA), (L, M) days to 50% flowering (DTF), (N, O) plant height (PH), and (P–R) primary branches (PB).

**Supplementary Fig. S16**. Validated SNP markers through KASP associated with different traits: (A–D) oil content, (E, F) oleic acid and linoleic acid, (G, H) 100-seed weight, (I) days to 50% flowering, (J) plant height, and (K) primary branches.

**Supplementary Fig. S17**. Graphs showing the relative expression of candidate genes in core collection accessions CC113, CC106, and S116 for (A) OC (g16872: myosin-binding protein), (B) OC (g64666: probable UDP-N-acetylglucosamine–peptide N-acetylglucosaminyltransferase SPINDLY isoform X1), (C) OA-LA (g43426: cytochrome P450 71A4-like), and (D) SW (g57921: protein FRIGIDA-ESSENTIAL 1-like isoform). Letters x, y, and z above error bars indicate statistically significant differences between CC113 and CC106, CC113 and S116, and CC113 and S116, respectively, as determined by Student *t*-test (*P* < 0.05).

**Supplementary Fig. S18**. Pangenome of safflower. (A) Modeling of the pan-genome depicting core and pan-genes. (B) Distribution of distinct pan-genes among core collection accessions.

**Supplementary Fig. S19**. Comparison of the Safflower_A2 genome assembly with Anhui 1 assemblies. (A) Chromosome-wise sequence length comparison with Anhui 1 genome assembly. (B) Comparison of assembly characteristics with Anhui 1 genome assembly.

**Supplementary Table S1**. Summary table of *k*-mer distribution at *k* = 17 in the safflower genome.

**Supplementary Table S2**. Summary table of different datasets used in the assembly of the reference genome sequence.

**Supplementary Table S3**. Mapping back statistics of the PacBio long reads and Illumina reads to the reference assembly.

**Supplementary Table S4**. Assessment of genome completeness using BUSCO through the Eudicot database.

**Supplementary Table S5**. Assessment of genome completeness using Merqury.

**Supplementary Table S6**. Filters applied to GBS SNP file, filtering criteria, and number of variants retained after each step.

**Supplementary Table S7**. Summary table for high-density SNP linkage map.

**Supplementary Table S8**. Circular consensus sequencing counts per sample for Iso-seq data.

**Supplementary Table S9**. Characterization of alternative splicing (AS) events by SUPPA2.

**Supplementary Table S10**. Annotation of repetitive elements in the Safflower_A2 genome.

**Supplementary Table S11**. Summary table comprises of total LTR-RT elements and their subfamilies.

**Supplementary Table S12**. Chromosomal distribution of predicted rRNA and tRNA genes.

**Supplementary Table S13**. Completeness assessment of the proteins in Safflower_A2 genome using BUSCO through the Eudicot database.

**Supplementary Table S14**. Number of annotated transcripts in the Safflower_A2 genome against various publicly available databases.

**Supplementary Table S15**. Major classes of resistance gene analogues (RGAs) in Safflower_A2 based on the domain, their count, percentage, and composition.

**Supplementary Table S16**. Functional annotation of the 236 NLRs and their association in diseases in different plants.

**Supplementary Table S17**. Accessions of core collections, their geographic distribution, and resequencing data.

**Supplementary Table S18**. Filtering criteria for core collection SNPs from resequencing data of the core collection.

**Supplementary Table S19**. Chromosome-wise distribution of filtered SNPs from core collection resequencing data.

**Supplementary Table S20**. Distribution of accession belonging to different gene pools in different clusters of the ADMIXTURE analysis.

**Supplementary Table S21**. Filtration criteria used for SNPs utilized for GWAS analysis.

**Supplementary Table S22**. Chromosomal distribution of SNPs after filtration (GWAS).

**Supplementary Table S23**. QTNs detected for traits OC, SW, PH, PB, HN, DTF, and OA-LA.

**Supplementary Table S24**. LD-block analysis indicating the chromosomal positions and size of LD blocks around the QTN regions.

**Supplementary Table S25**. KASP primers designed for multiple traits across different sites.

**Supplementary Table S26**. Validated SNP markers through KASP associated with different traits and their corresponding genotype panels.

**Supplementary Table S27**. Phenotypic values of post harvest traits (OC, OA-LA, and SW) for 3 contrasting accessions CC106, CC113, and S116.

**Supplementary Table S28**. Candidate genes selected for qRT-PCR analysis. Primer sequences, Tm, and corresponding product size have also been indicated.

**Supplementary Table S29**. qRT-PCR analysis of candidate genes during seed development. Relative expression (fold change) of 3 candidate genes measured by qRT-PCR in 3 genotypes (CC113, CC106, S116) at 4 seed developmental stages (5, 10, 20, and 30 days after pollination, DAP). Fold-change values are shown as means across 3 biological replicates with accompanying standard deviation (Std. Dev.) and standard error (Std. Err.). Fold change was calculated using the 2^−ΔΔCT^ method, and values are expressed relative to the calibrator sample (CC106_5DAP).

**Supplementary Table S30**. Accessions with a high number of variable genes that can be useful in regional breeding programs.

**Supplementary Table S31**. Distribution of the stress-related genes across different regional pools.

**Supplementary Table S32**. Number of the stress-related genes across different regional pools.

## Abbreviations

100SW: 100-seed weight; AS: alternative splicing; BLINK: Bayesian-information and Linkage-disequilibrium Iteratively Nested Keyway; BUSCO: Benchmarking Single Copy Orthologs; CCS: circular consensus sequence; CGA: candidate gene analysis; DANTE: Domain-based Annotation of Transposable Elements; DAP: days after pollination; DTF: days to 50% flowering; EC: Enzyme Code; FarmCPU: Fixed and random model Circulating Probability Unification; FM: functional marker; GBS: genotyping by sequencing; GO: Gene Ontology; GWAS: genome-wide association study; HN: number of heads; KASP: Kompetitive Allele-Specific PCR; KEGG: Kyoto Encyclopedia of Genes and Genomes; KOG: Cluster of Orthologous Groups; LAI: LTR assembly index; LD: linkage decay; LINE: long interspersed nuclear element; LTR: long terminal repeat; LTR-TE: LTR retrotransposon; LOD: logarithm of the odds (LOD) ; MITE: miniature inverted-repeat transposable element; MLMM: multilocus mixed model; MTA: marker–trait association; OA-LA: oleic and linoleic acid; OC: oil content; PAV: presence–absence variation; PB: primary branch; PBS: primer binding site; PC: principal component; PCA: principal component analysis; PH: plant height; Q-Q: quantile–quantile; qRT-PCR: quantitative reverse transcription PCR; QTN: quantitative trait nucleotide; QV: quality value; RGA: resistance gene analogue; RIL: recombinant inbred line; RNA-seq: RNA sequencing; rRNA: ribosomal RNA; SINE: short interspersed nuclear element; SNP: single-nucleotide polymorphism; SSR: simple sequence repeat; SSR: simple sequence repeat; SV: structural variant; TE: transposable element; TIR: tandem inverted repeat; tRNA: transfer RNA; TSD: target site duplication.
