# Supplementary material for: Improved reference assembly and core collection resequencing to facilitate exploration of important agronomical traits for the improvement of oilseed crop, Carthamus tinctorius L
Source: Gigascience. 2025 Dec 11;15:giaf151. doi: 10.1093/gigascience/giaf151 (PMC12888819; doi:10.1093/gigascience/giaf151)
Supplement: giaf151_Supplemental_File [file giaf151_supplemental_file.docx]

**Improved reference assembly and core collection re-sequencing to facilitate exploration of important agronomical traits for the improvement of oilseed crop, Carthamus tinctorius L.**

Megha Sharma^1#^, Varun Bhardwaj^1#^, Praveen Kumar Oraon^1^, Shivani Choudhary^1^, Heena Ambreen^2^, Rohit Nandan Shukla^3^, Harsha Rayudu Jamedar^4^, Ajitha Vijjeswarapu^4^, Vandana Jaiswal^5^, Palchamy Kadirvel^4^, Arun Jagannath^1*^, Shailendra Goel^1*^

**Supplementary Figures**


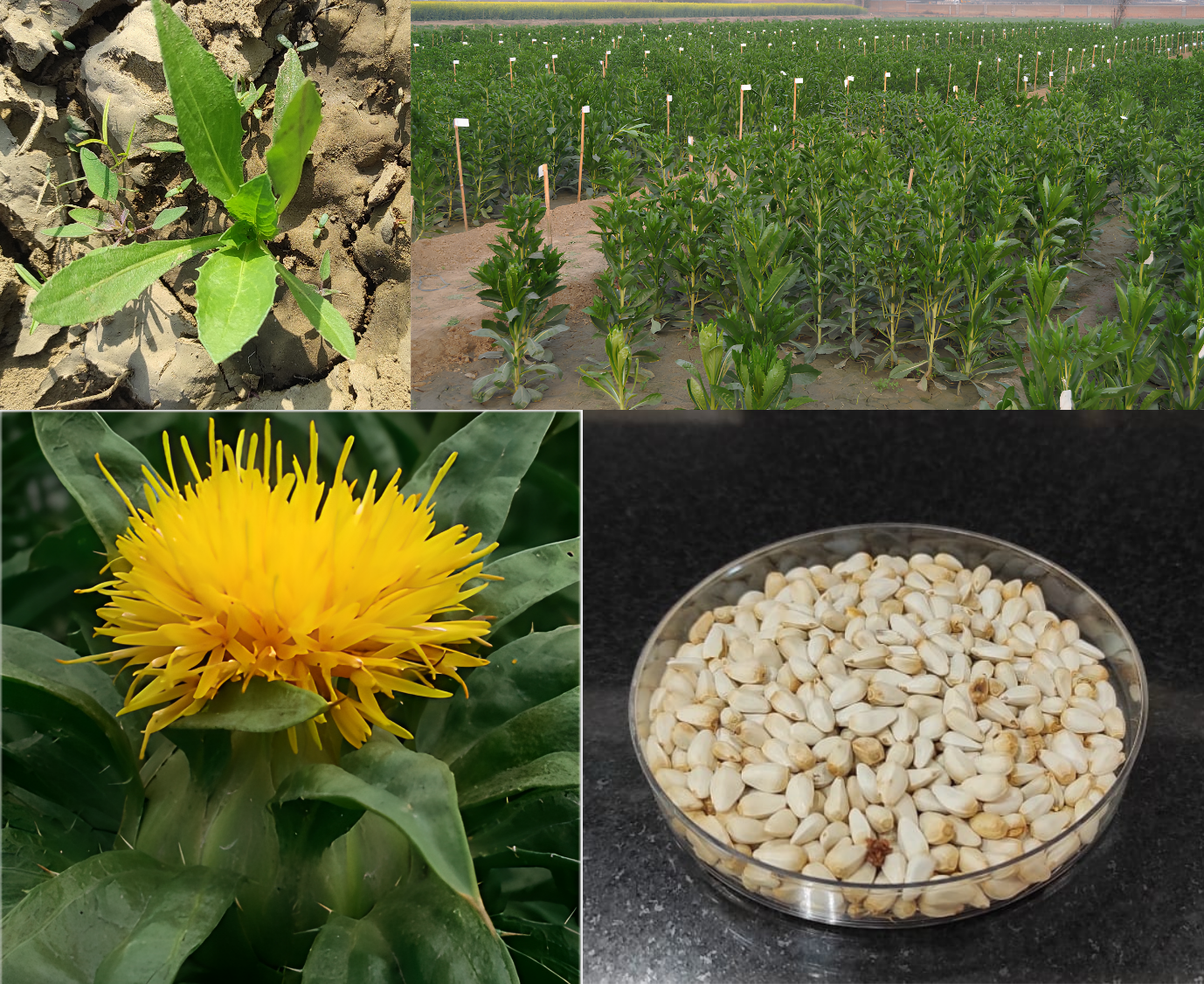


a

b

**Supplementary Fig. S1**: Phenotype of Safflower accession “A2” (PI:560169) used in this study. (a) at flowering stage. (b) Safflower_A2 seeds.


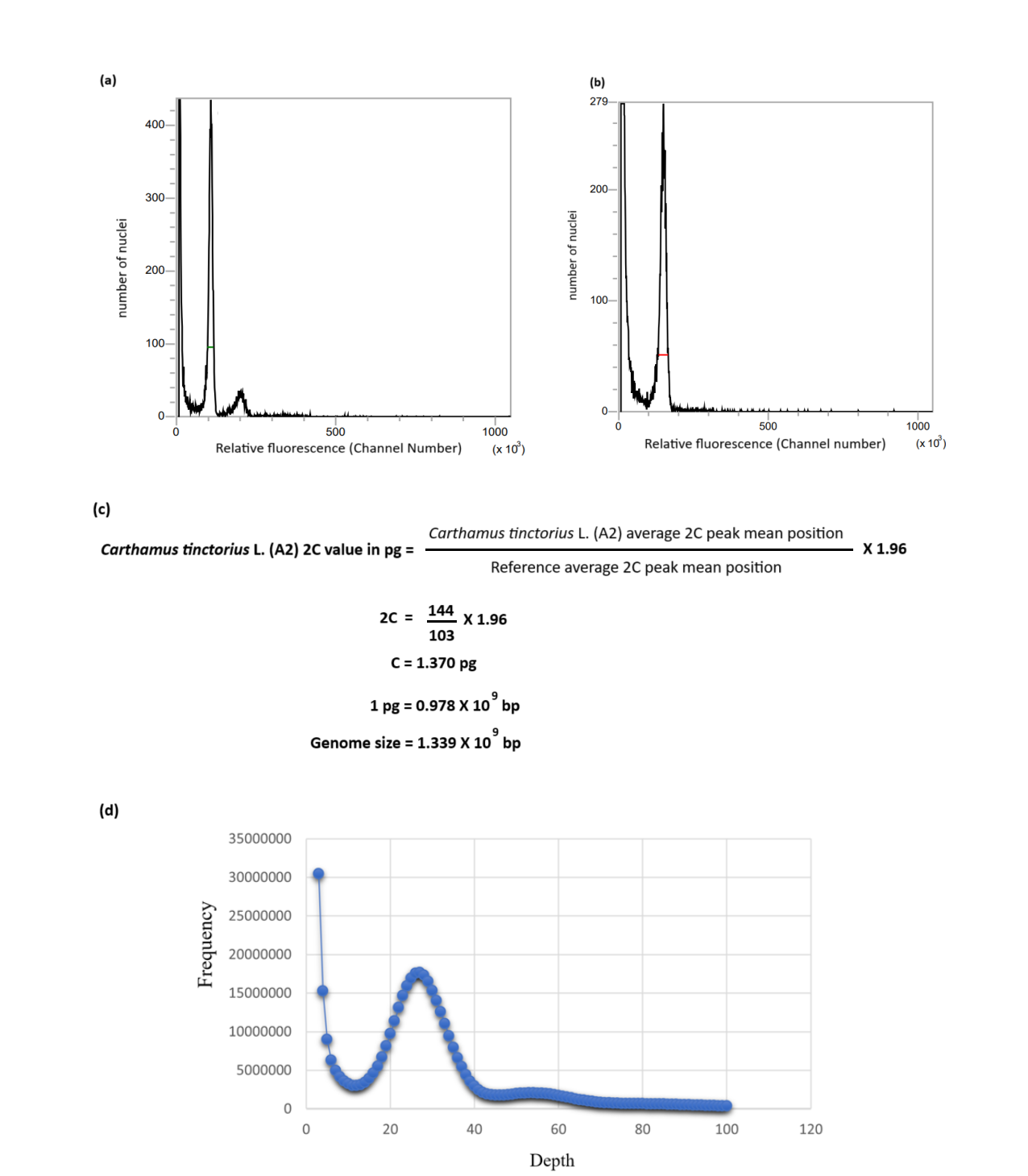


a

b

c

d

**Supplementary Fig. S2**: Estimation of Safflower_A2 genome size (a) using flow cytometry analysis, *Solanum lycopersicum* L. ‘Stupicke’ polni’ rane’ (reference; Coefficient of Variation 4.61%) used as a control (b) *Carthamus tinctorius* L. (A2; Coefficient of Variation 5.23%) (c) Calculation of Safflower_A2 genome size based on average peak mean positions of A2 versus reference (d) K-mer-based genome size estimation displaying 17-mer depth distribution in Safflower_A2 derived using error-corrected CCS reads from PacBio HiFi reads.


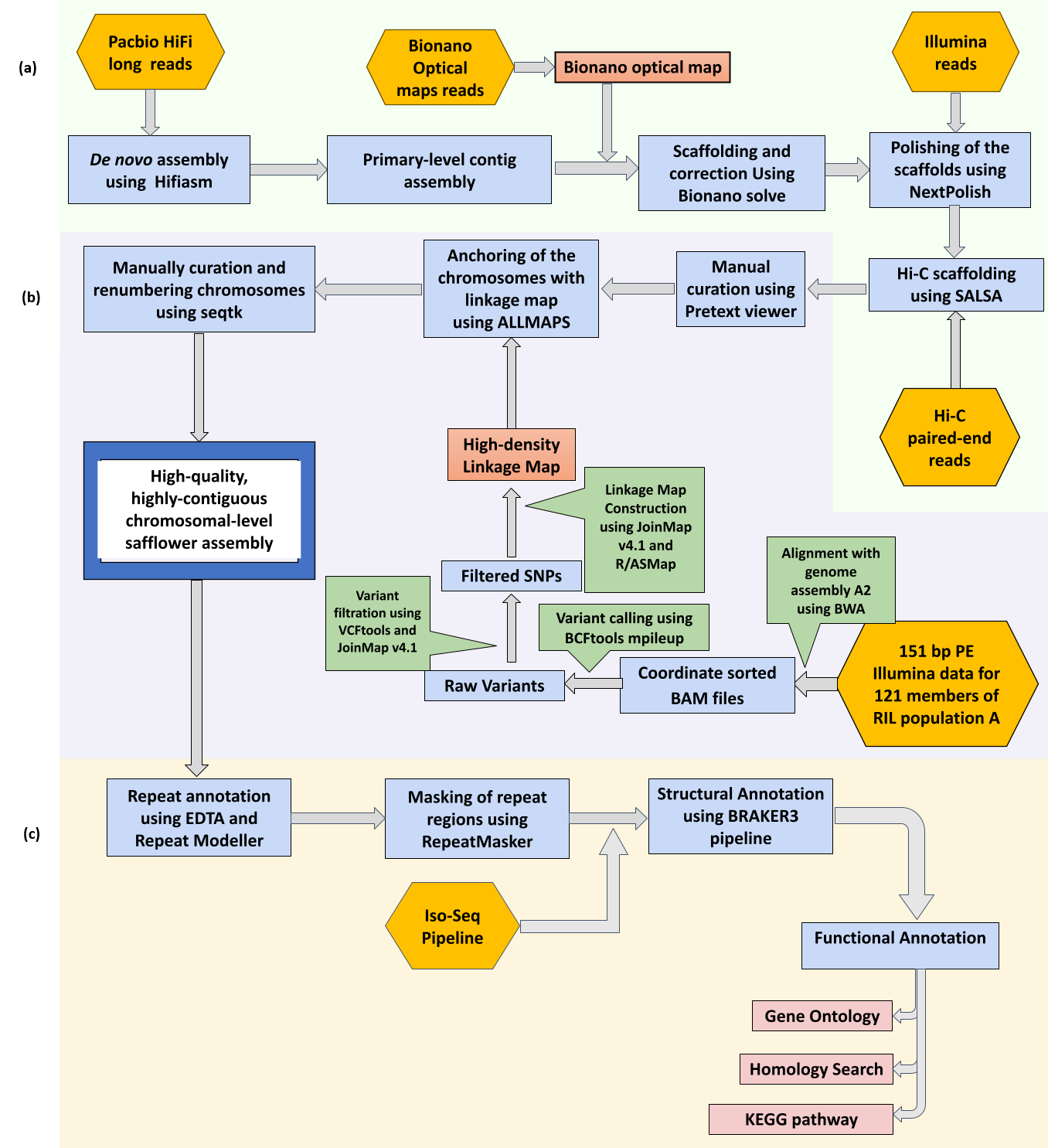


a

b

c

**Supplementary Fig. S3:** Workflow of Genome Assembly, Curation and Annotation for Safflower_A2. (a) Chromosome-level genome assembly was constructed using a combination of PacBio HiFi reads, Bionano optical mapping, Illumina reads and Hi-C (b) Genome assembly was error-corrected using a high-density linkage map constructed from GBS data of RIL population A (c) Manually curated and error-corrected genome assembly was subjected to repeat masking, followed by gene prediction using high-quality transcripts from the Iso-Seq pipeline as evidence and finally functional annotation was carried out, utilizing various public nucleotide and protein databases.


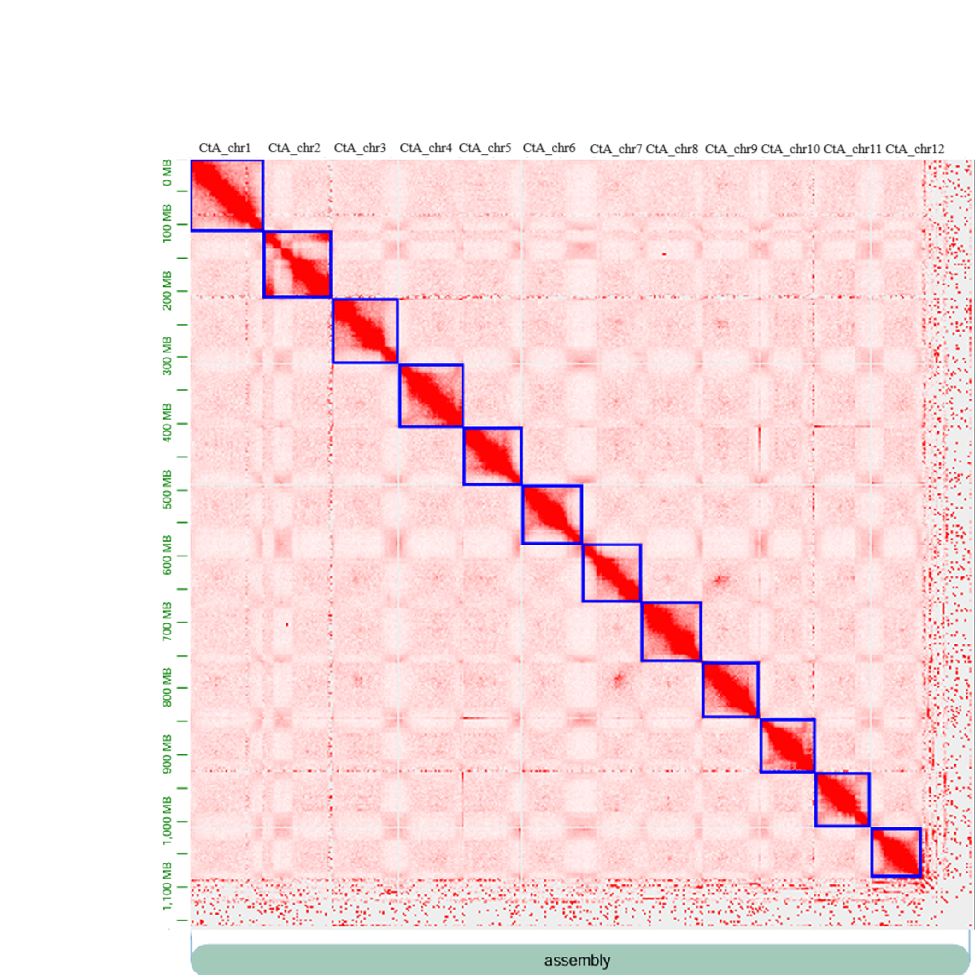


**Supplementary Fig. S4:** Hi-C interaction map of chromosome-level assembly of Safflower_A2.


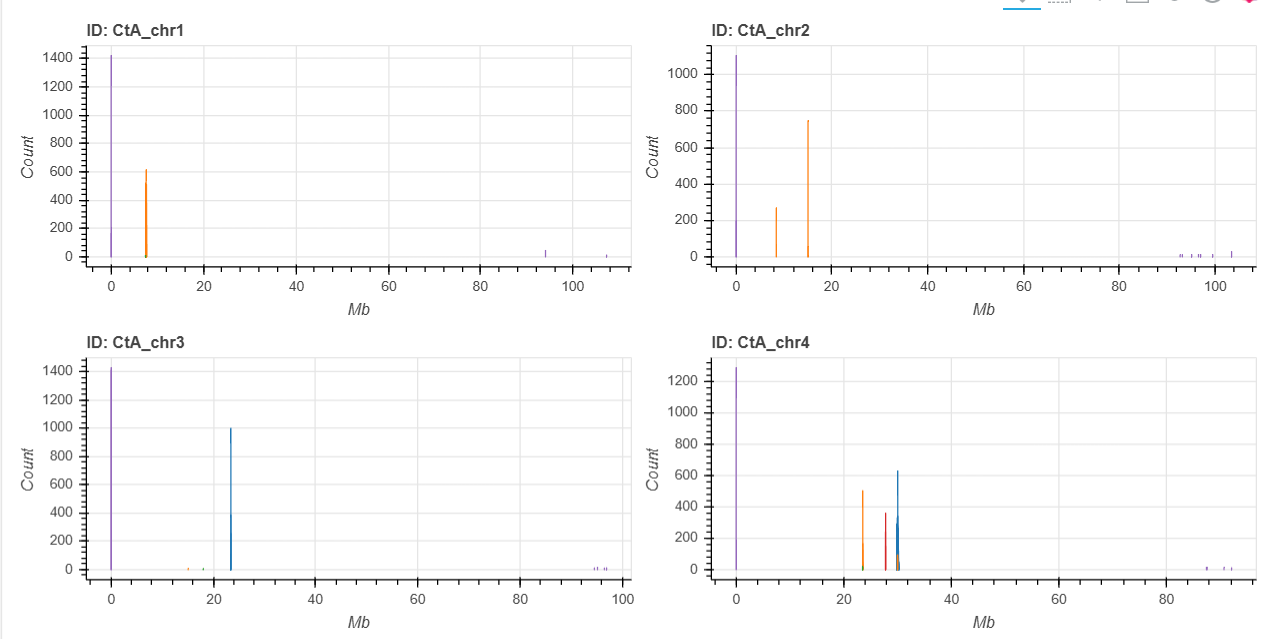

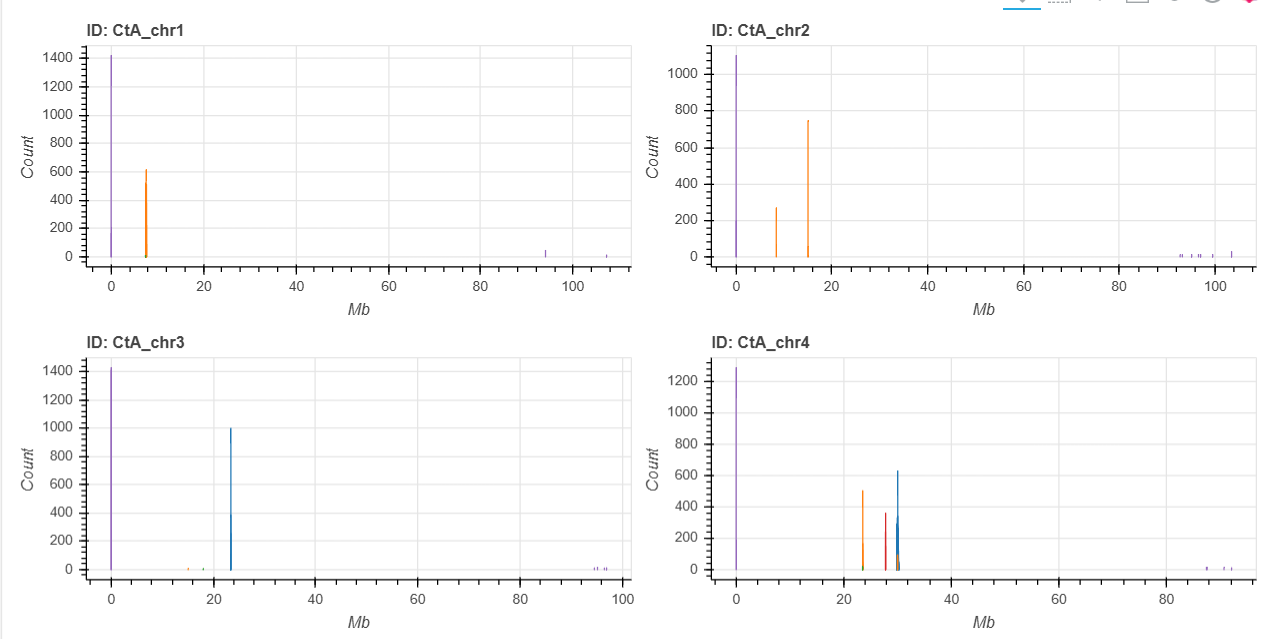


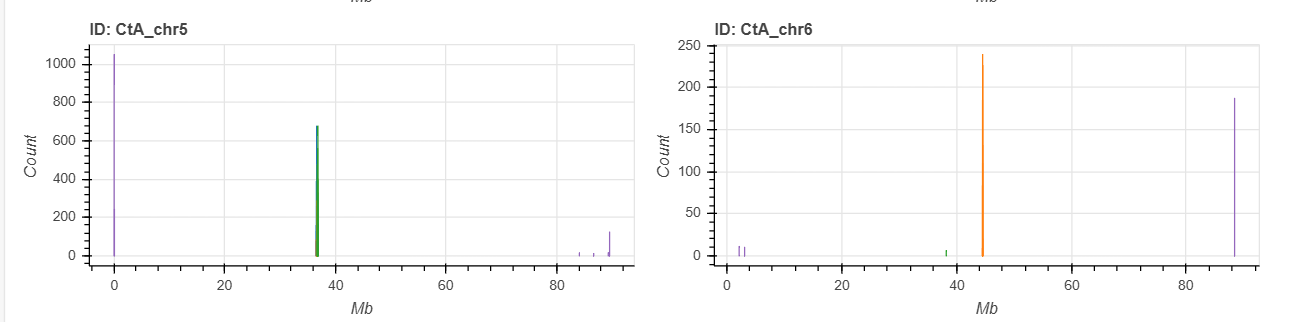


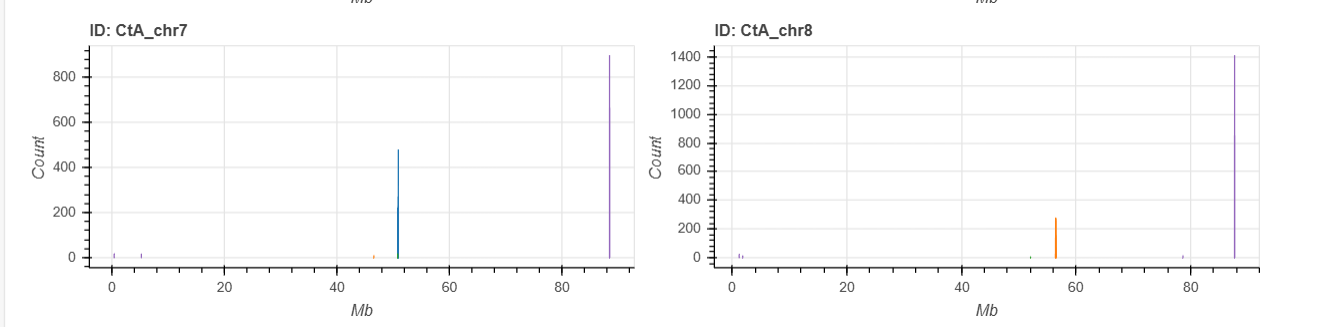


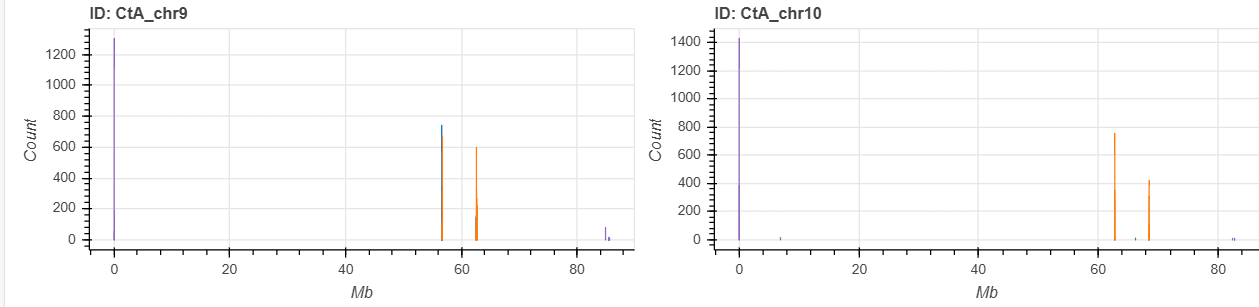


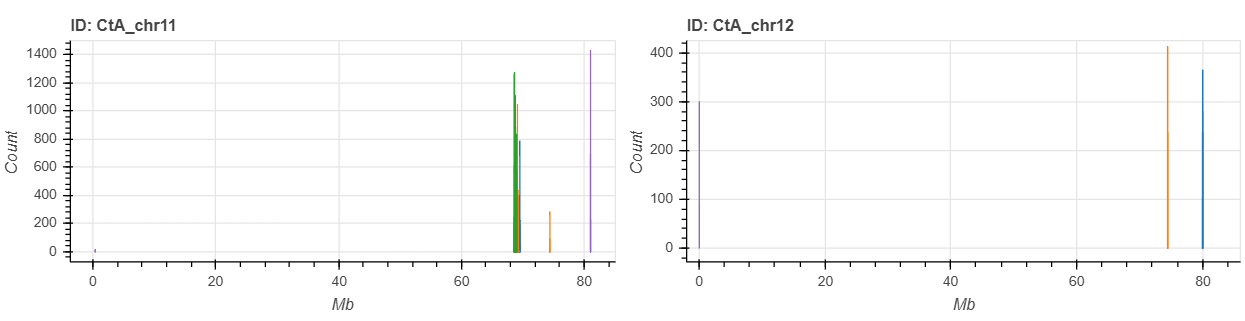


**Supplementary Fig. S5:** Telomeres and centromeres of the safflower genome. The x-axis represents the length of chromosomes (Mb), y-axis represents the count of the telomeric or centromeric repeats in the genome. Colour code: Purple: Telomeric repeats, Red: CEN repeats of 342bp, Green: CEN repeats of 348bp, Orange: CEN repeats of 349 bp and blue: CEN repeats of 350bp.


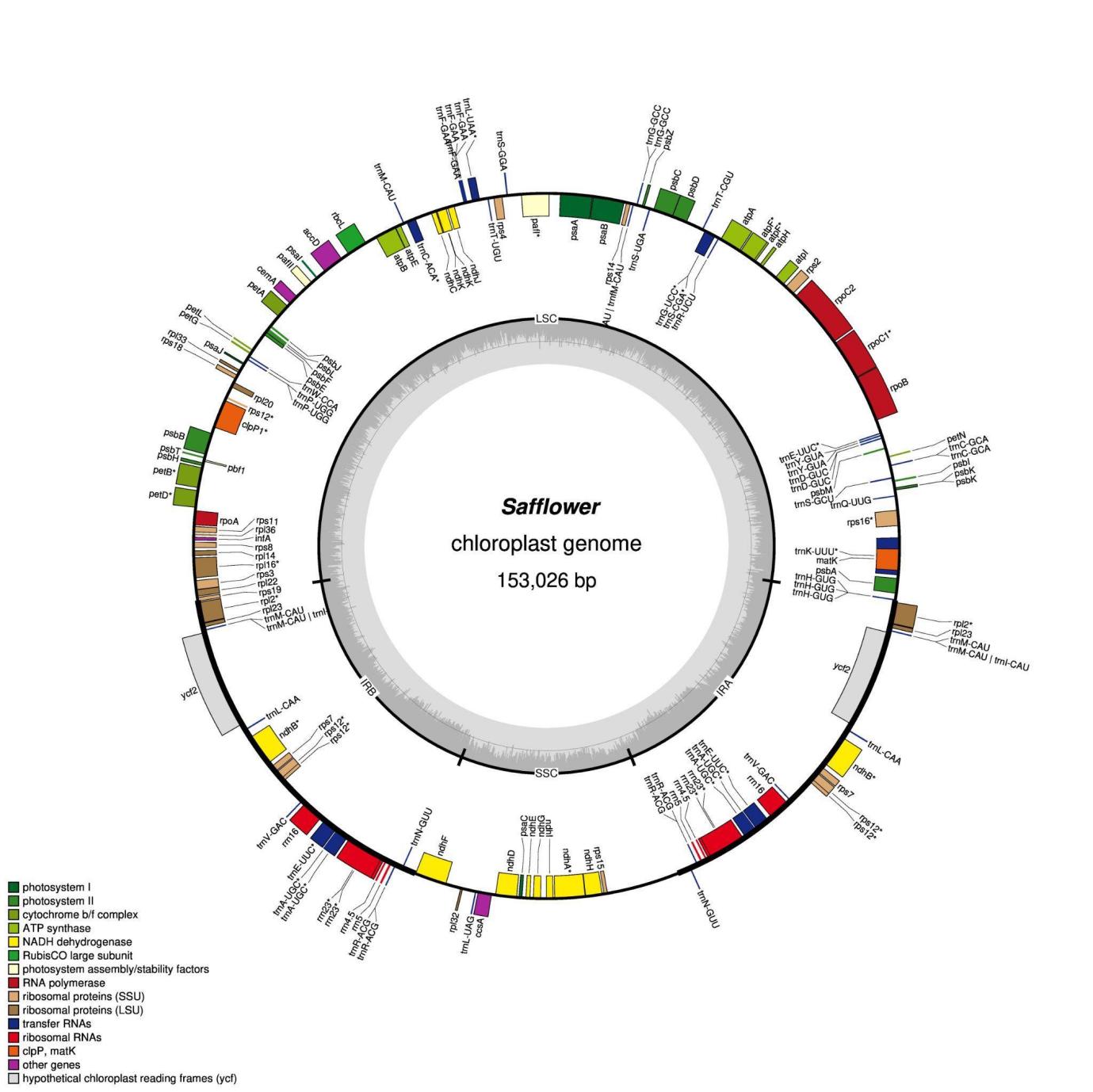


**Supplementary Fig. S6:** Fully annotated chloroplast genome from Safflower_A2 assembled using Illumina paired end reads.


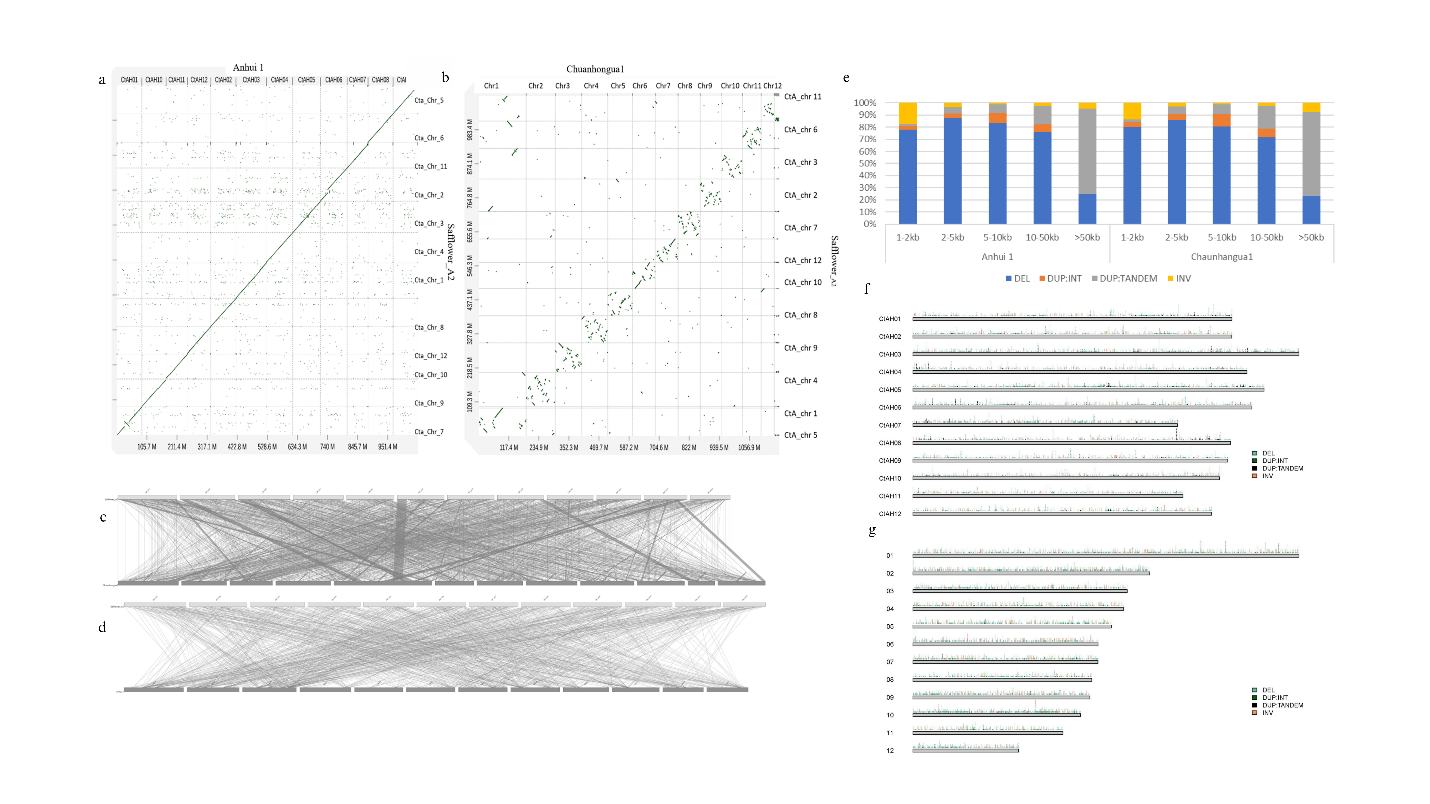

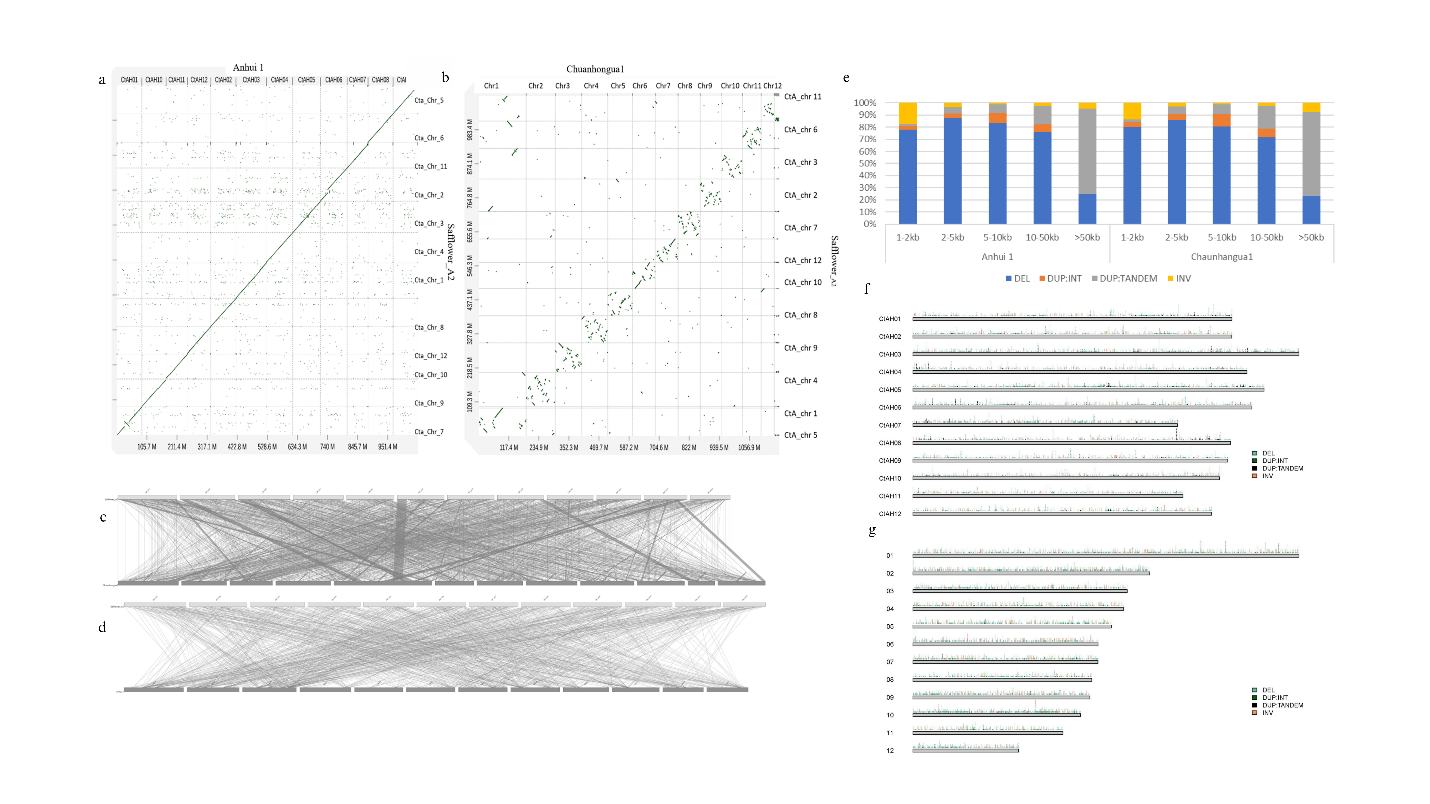


**Supplementary Fig. S7:** Comparison of the Safflower_A2 genome assembly with earlier assemblies (a) Dot plot showing one-to-one alignment between A2 genome assembly and Anhui 1 genome assembly (b) Dot plot showing alignment between A2 genome assembly and Chuanhanghua 1 genome assembly. Translocation events detected in the (c) Chuanhanghua 1 and (d) Anhui 1 genome. (e) Statistics of structural variants including deletions (DEL), Inversion (INV), duplication (DUP:INT) and tandem repeats duplications (DUP: INV) detected in this study. Chromosomal location of the structural variants in (f) Anhui 1 and (g) Chuanhanghua 1.


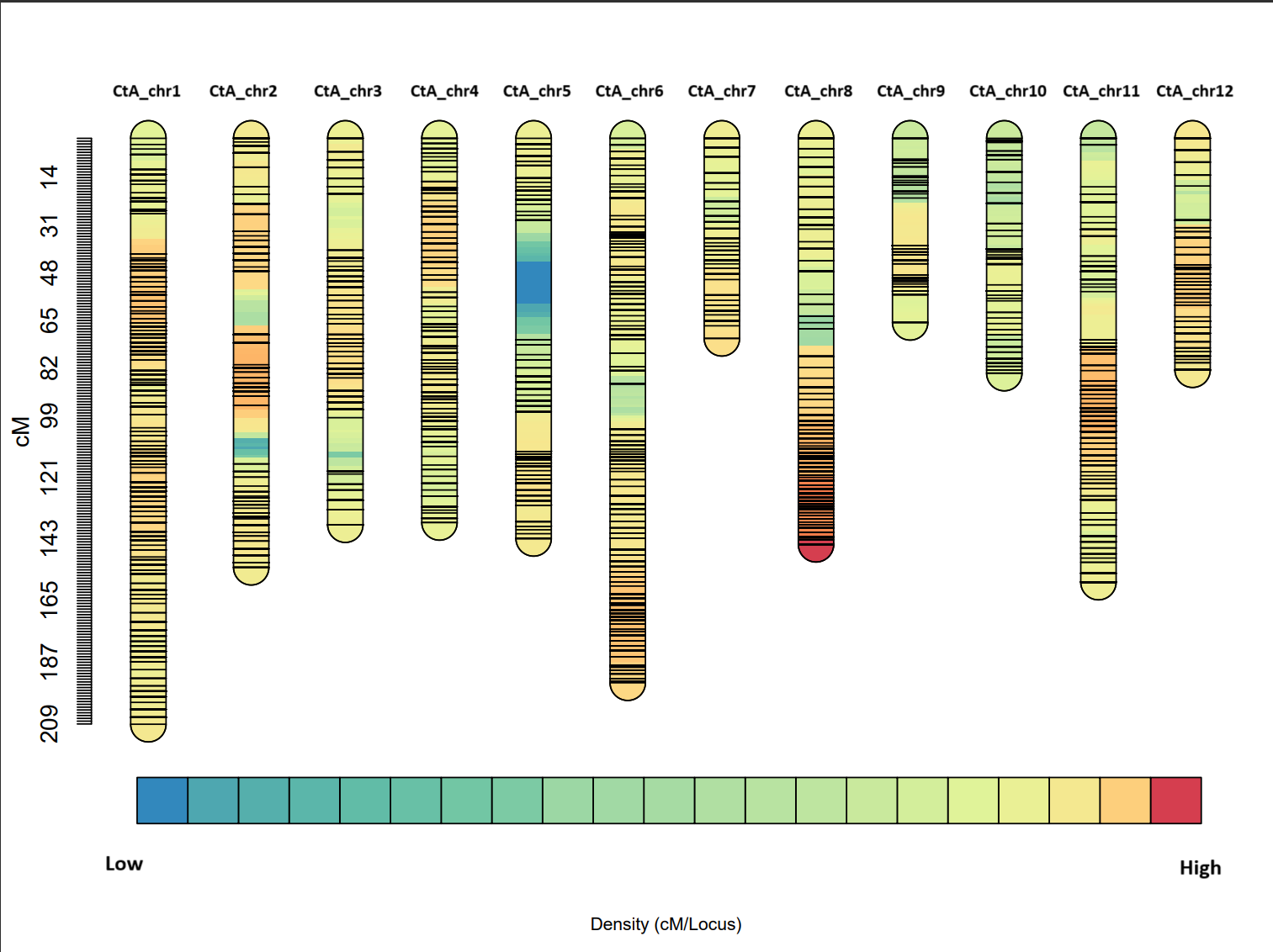


**Supplementary Fig. S8:** SNP-based high-density linkage map of safflower. Colours indicate marker density.


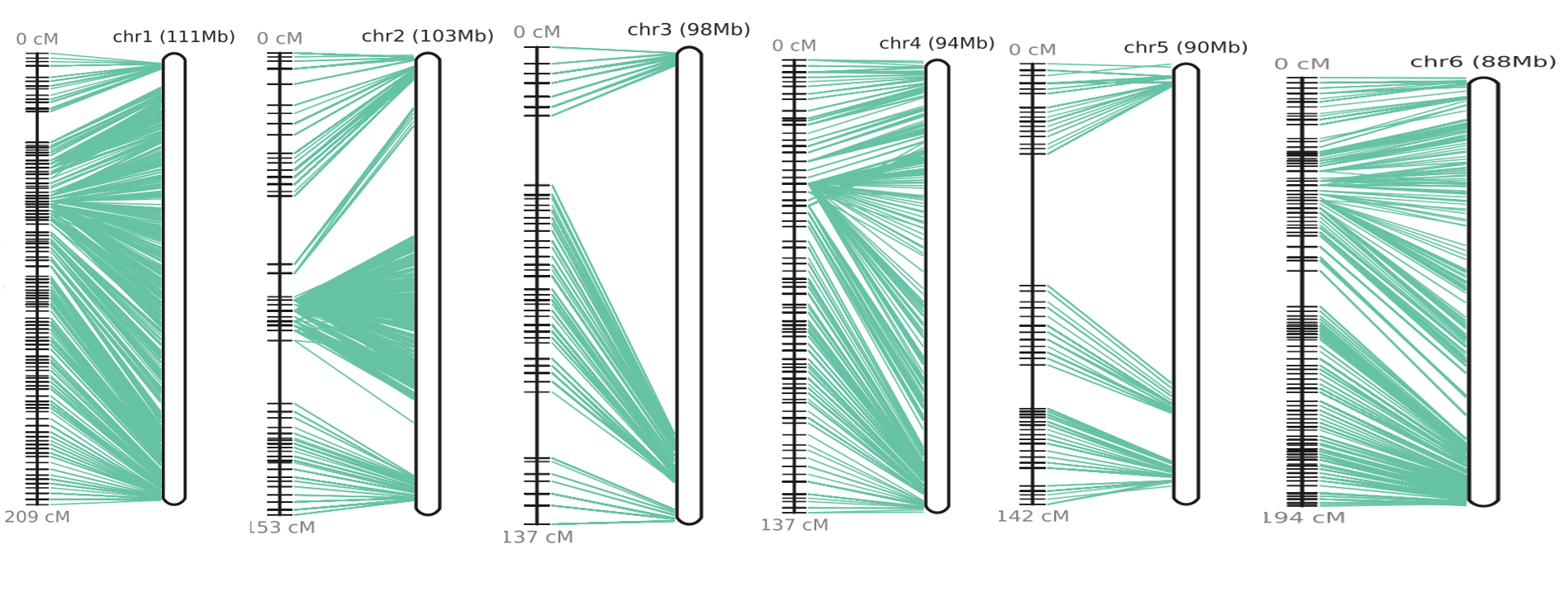


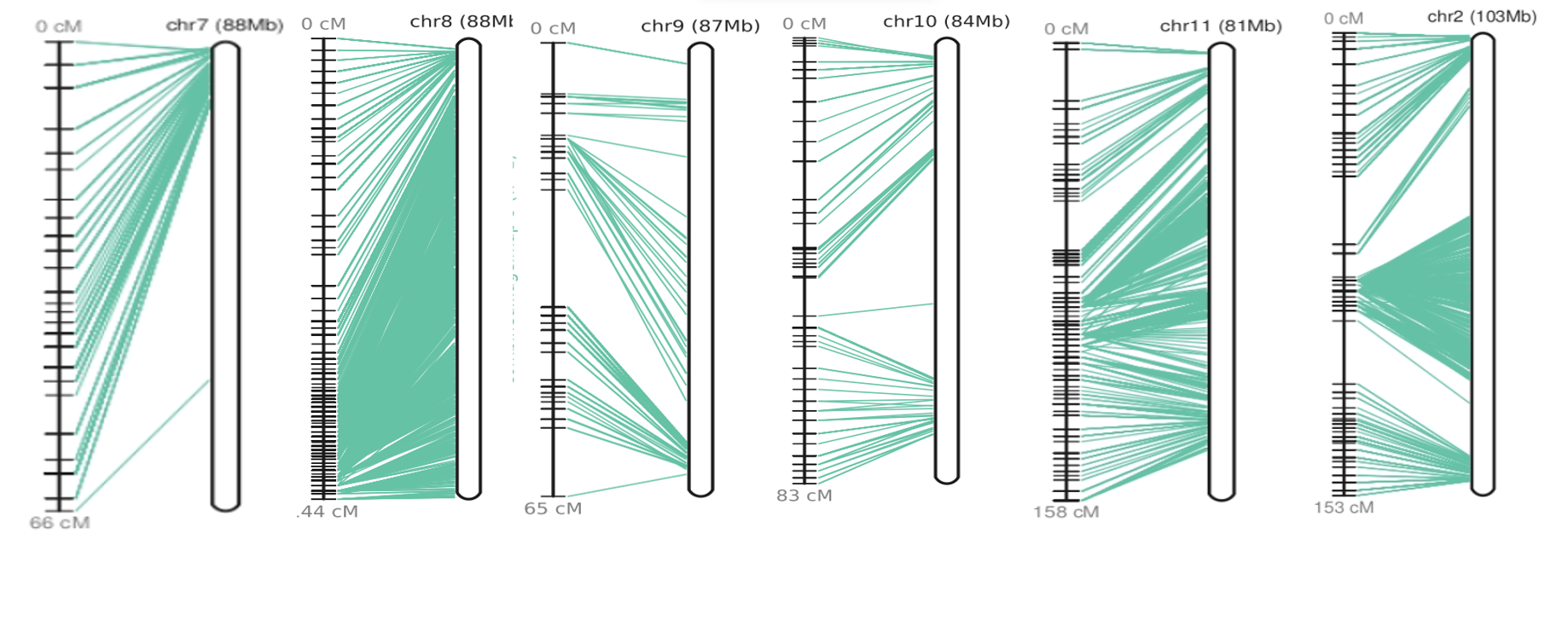


**Supplementary Fig. S9:** Alignment of the genome assembly of Safflower_A2 with the high-density linkage map constructed using GBS data for RIL population A.

**
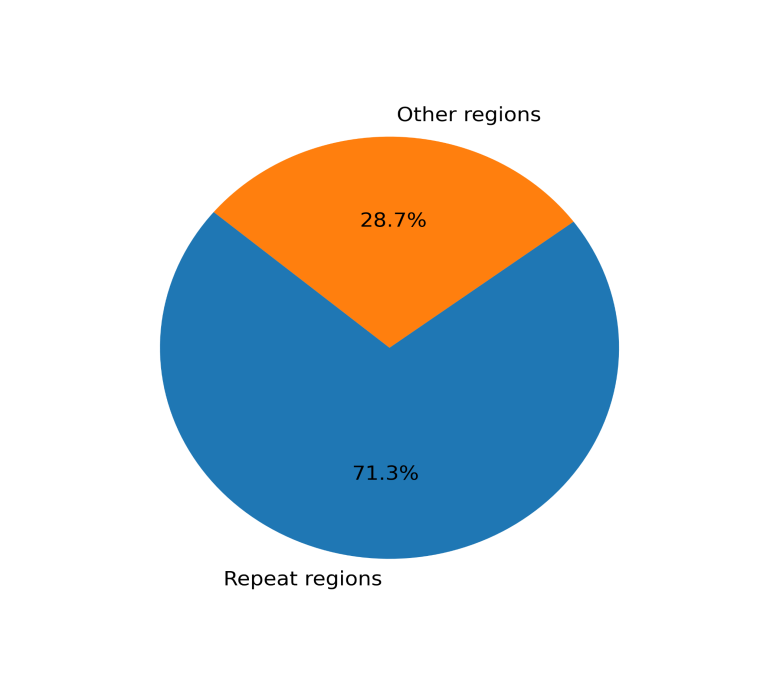
**

a

**
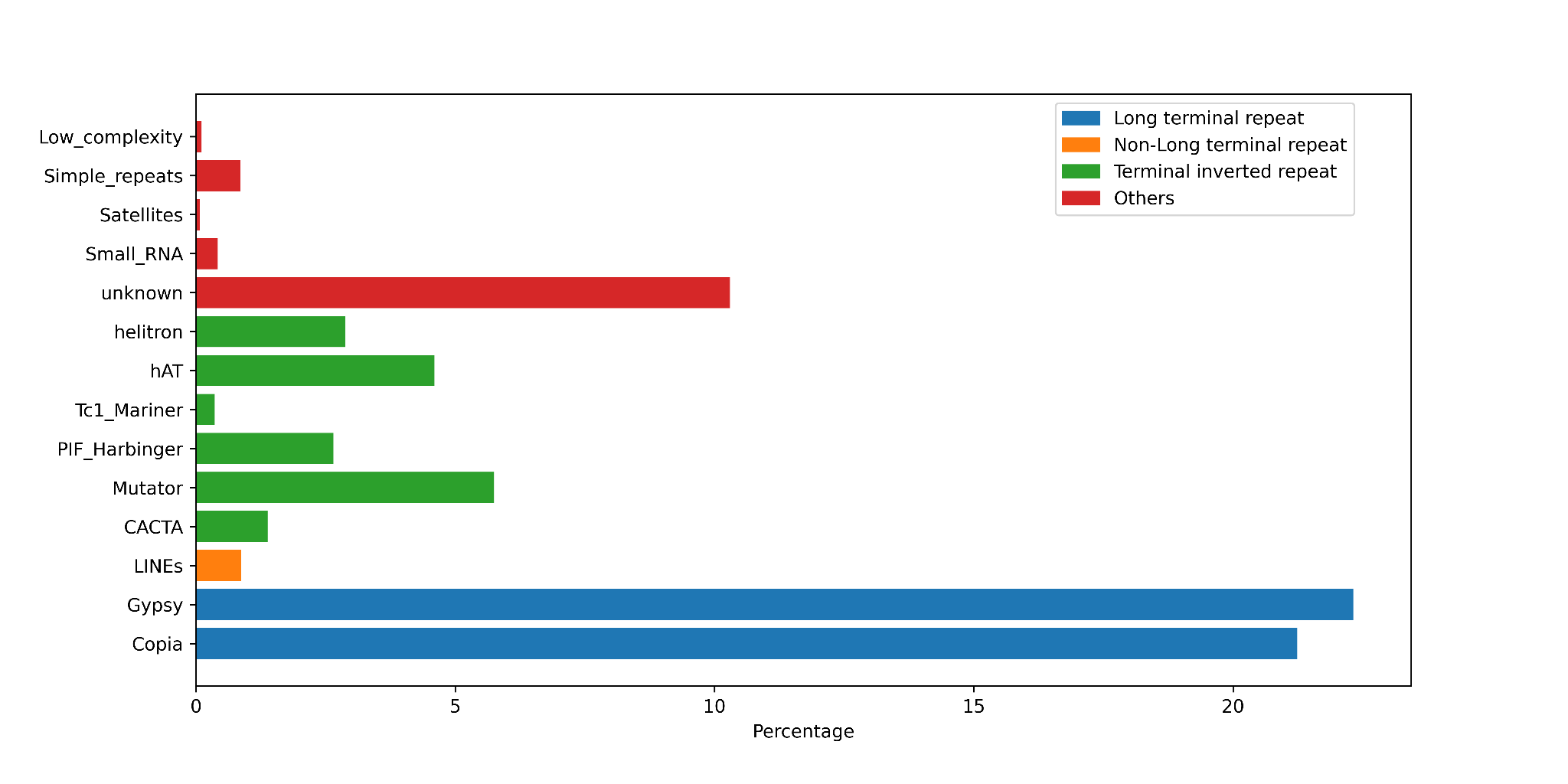
Supplementary Fig. S10**: Characteristics of the repetitive elements in the Safflower_A2 genome. (a) Proportion of the repetitive element in the safflower genome (b) Relative proportion of different classes of repetitive elements in the safflower genome.

b

**
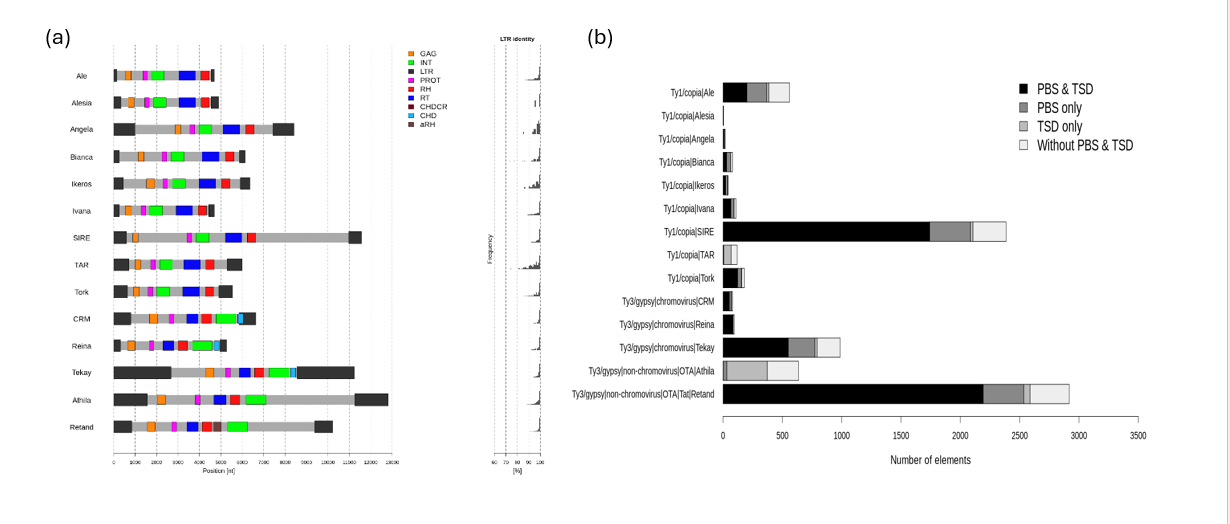

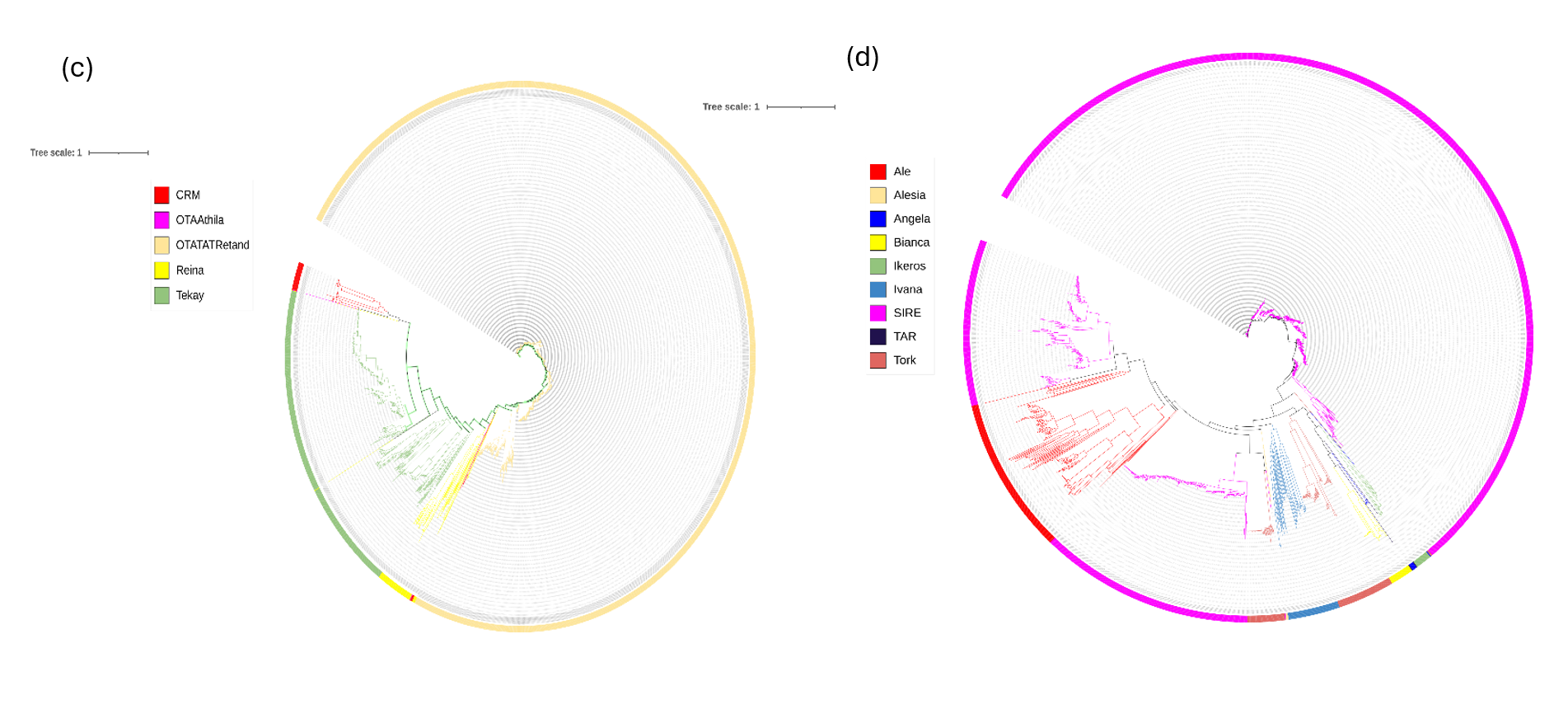
**

b

a

c

d

**
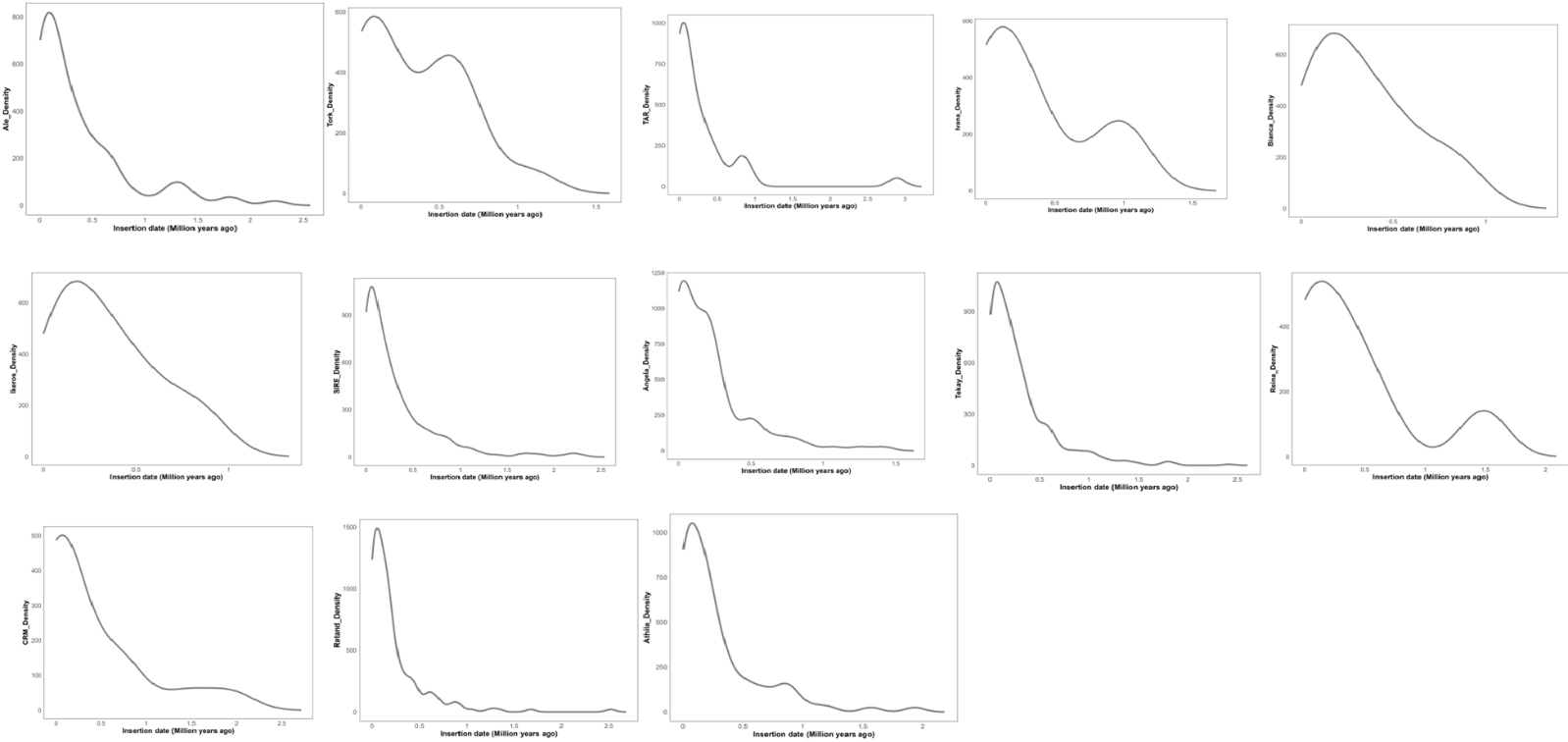
**

e

**Supplementary Fig. S11:** Analysis of complete LTR-TE in safflower genome. (a) Different families of the LTR-RT with the associated domains detected in this study. (b) Distribution of the different families of LTR-RT based on presence/absence of PBS and TSD. Phylogenetic tree of (c) Ty3/G*ypsy* elements and (d) Ty1/*Copia.* (e) Insertion time of the different classes of LTR-RT.


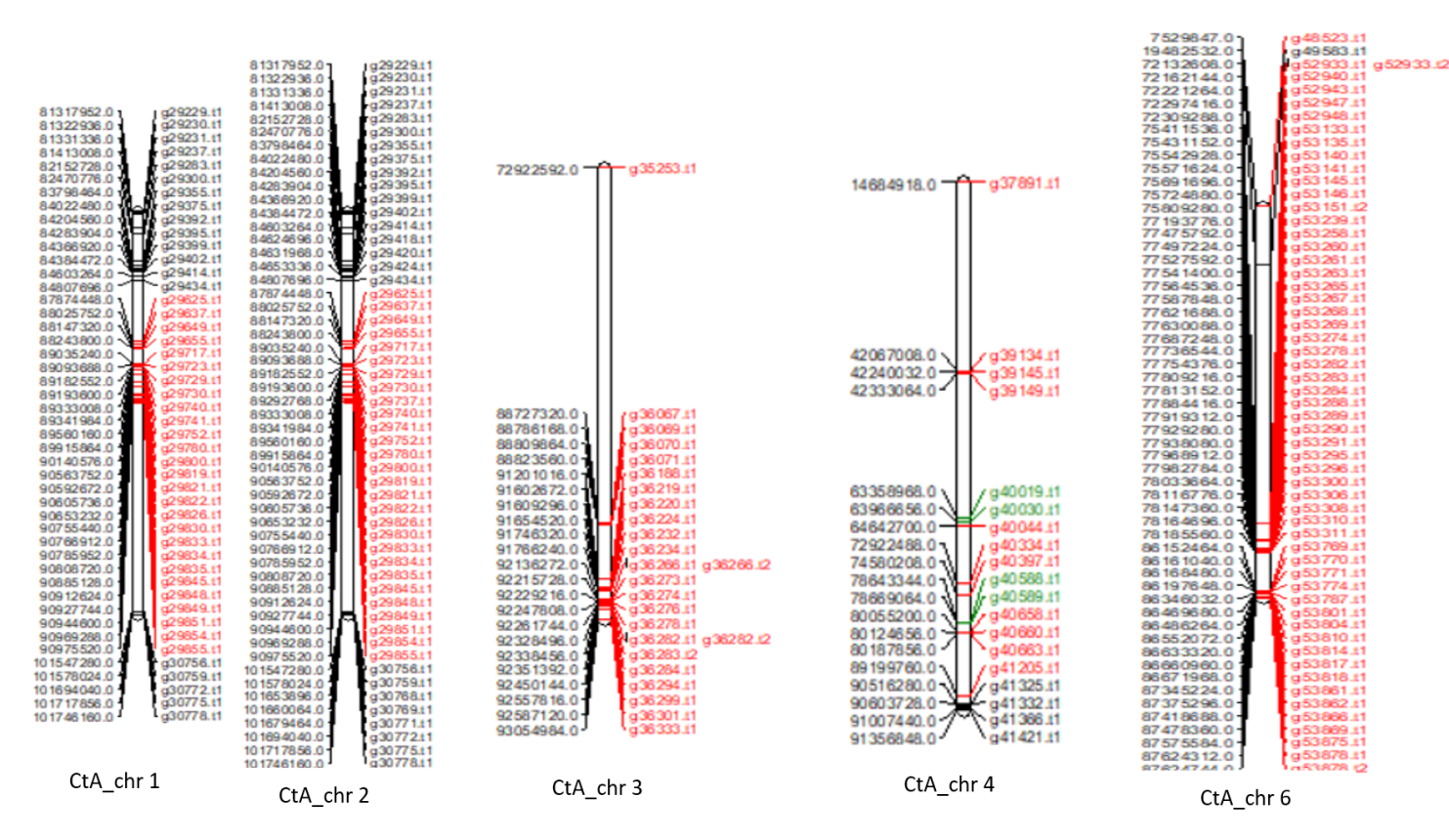
**
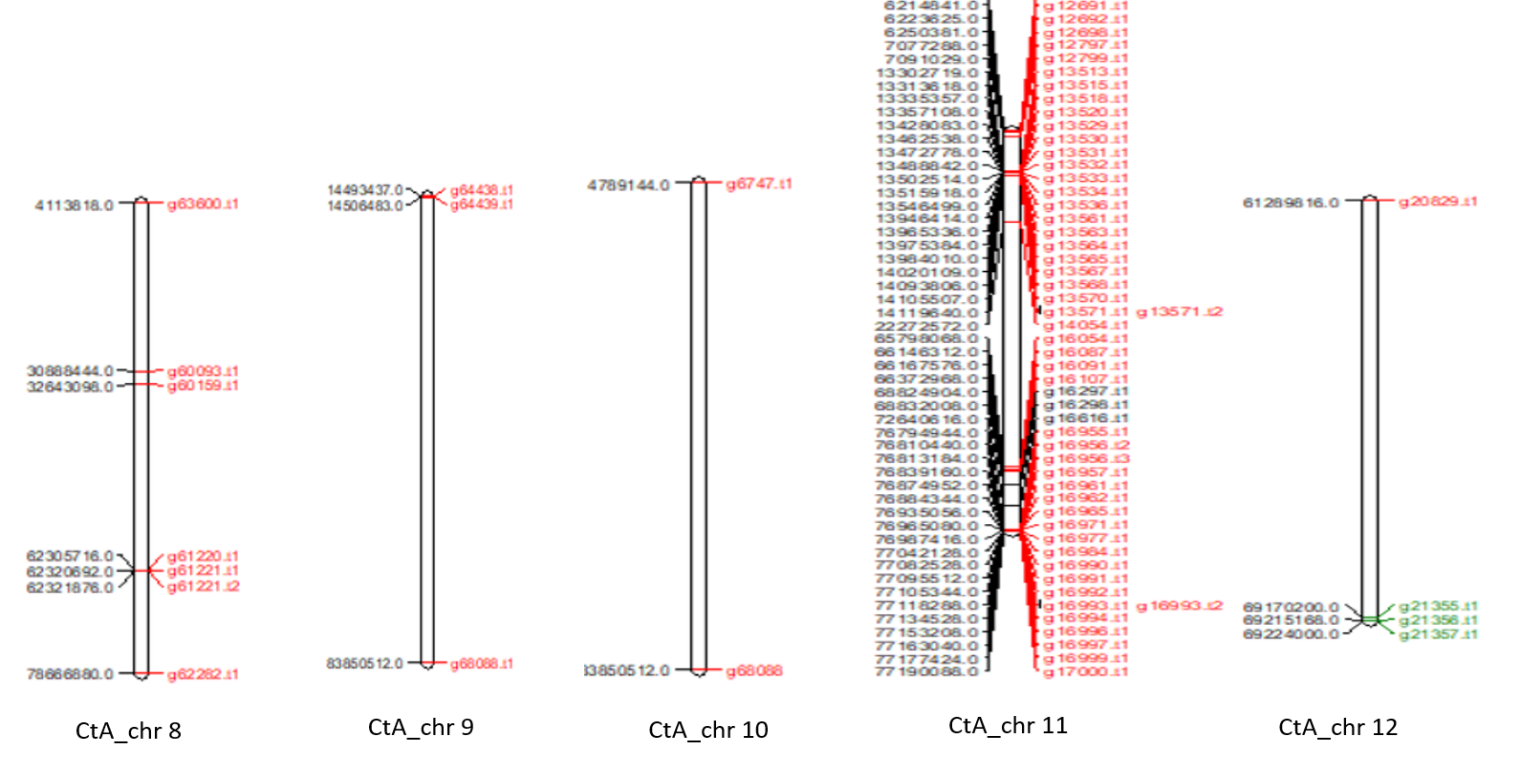
**

**Supplementary Fig. S12:** Distribution of the NLR genes on safflower genome. Red bars represent the TNL genes, black bars represent CNL genes and green bar represents the RNL genes.


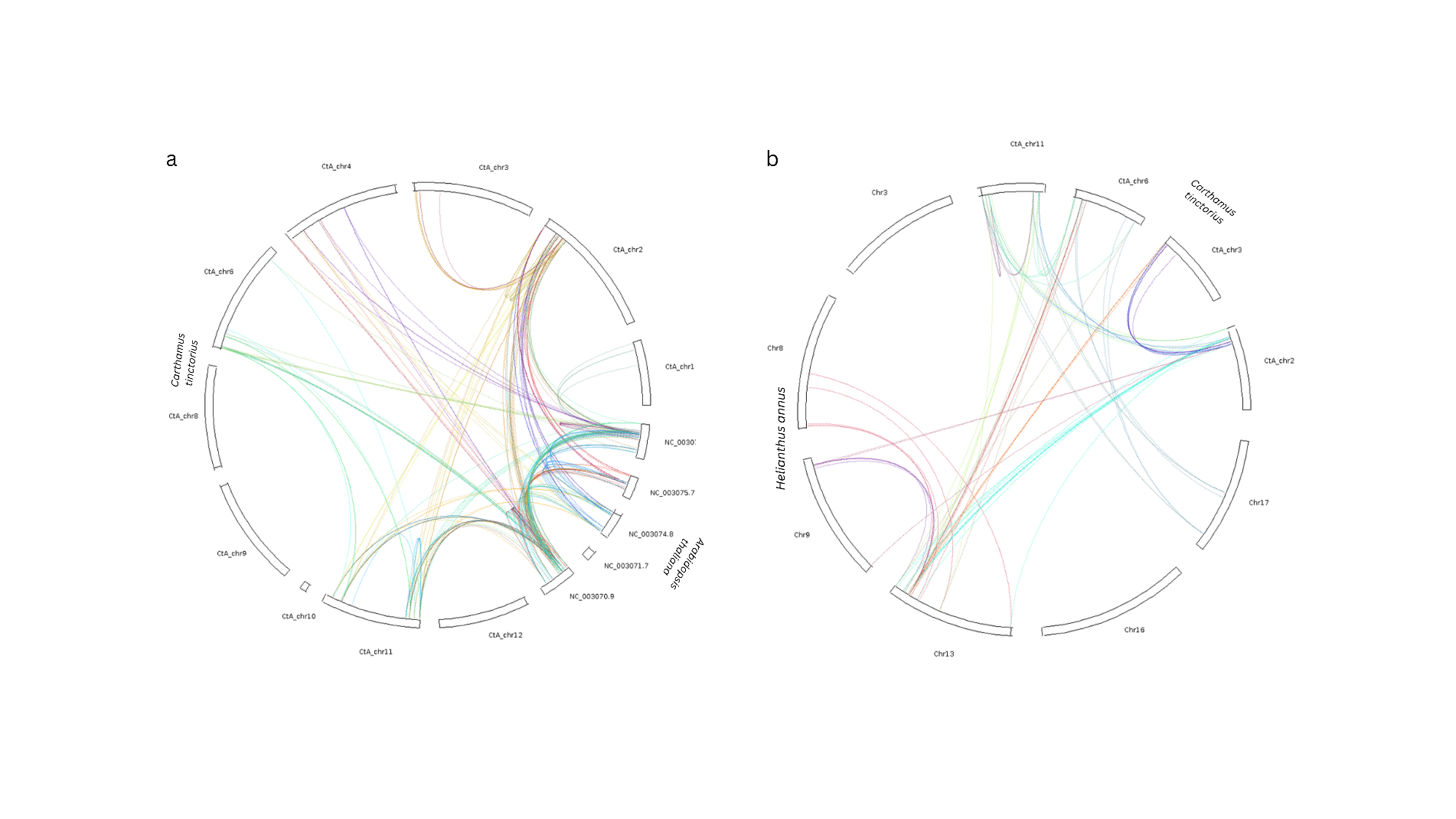

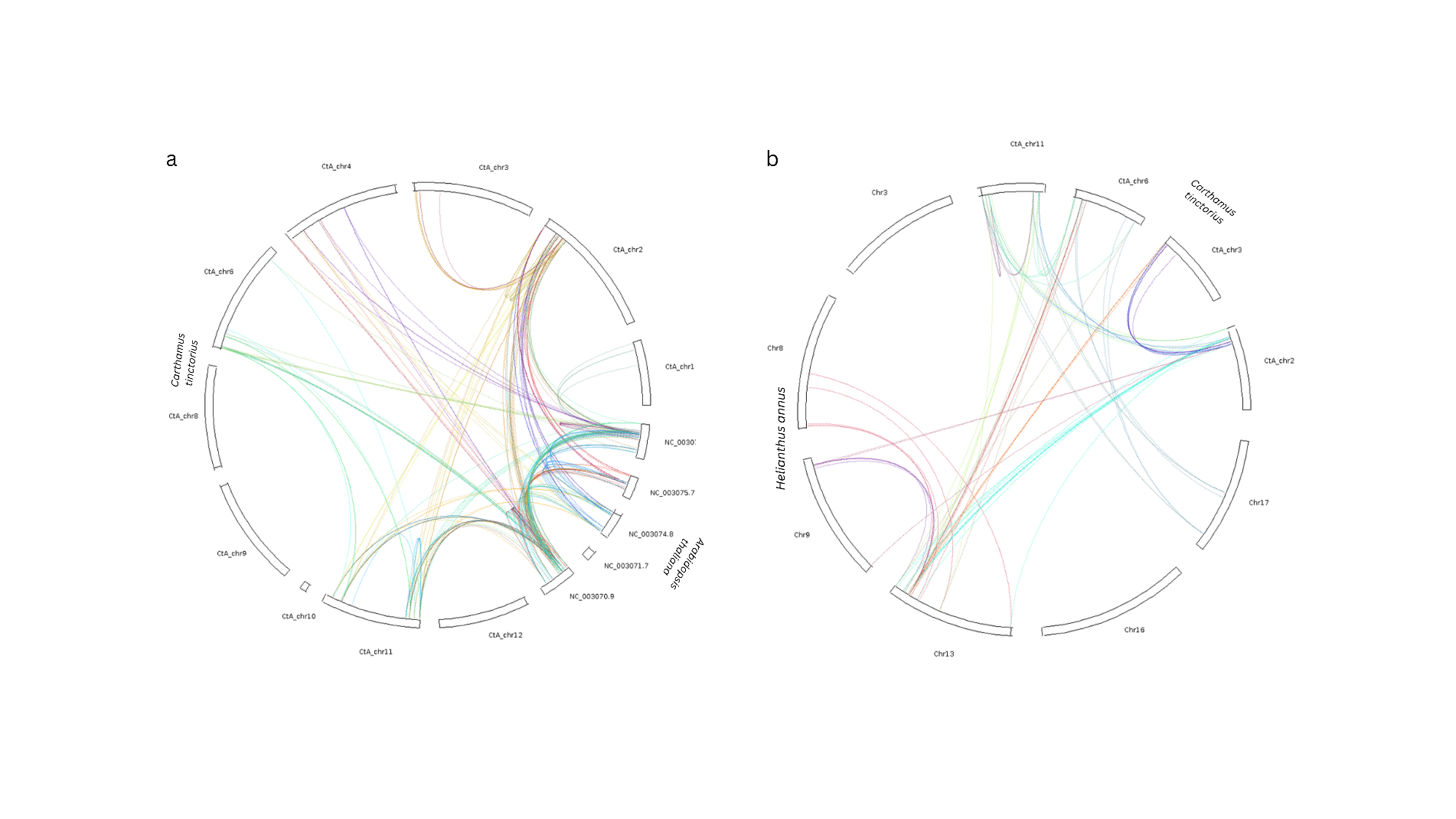


**Supplementary Fig. S13:** Collinear blocks detected between (a) Safflower and *Arabidopsis thaliana* and (b) Safflower and *Helianthus annus*


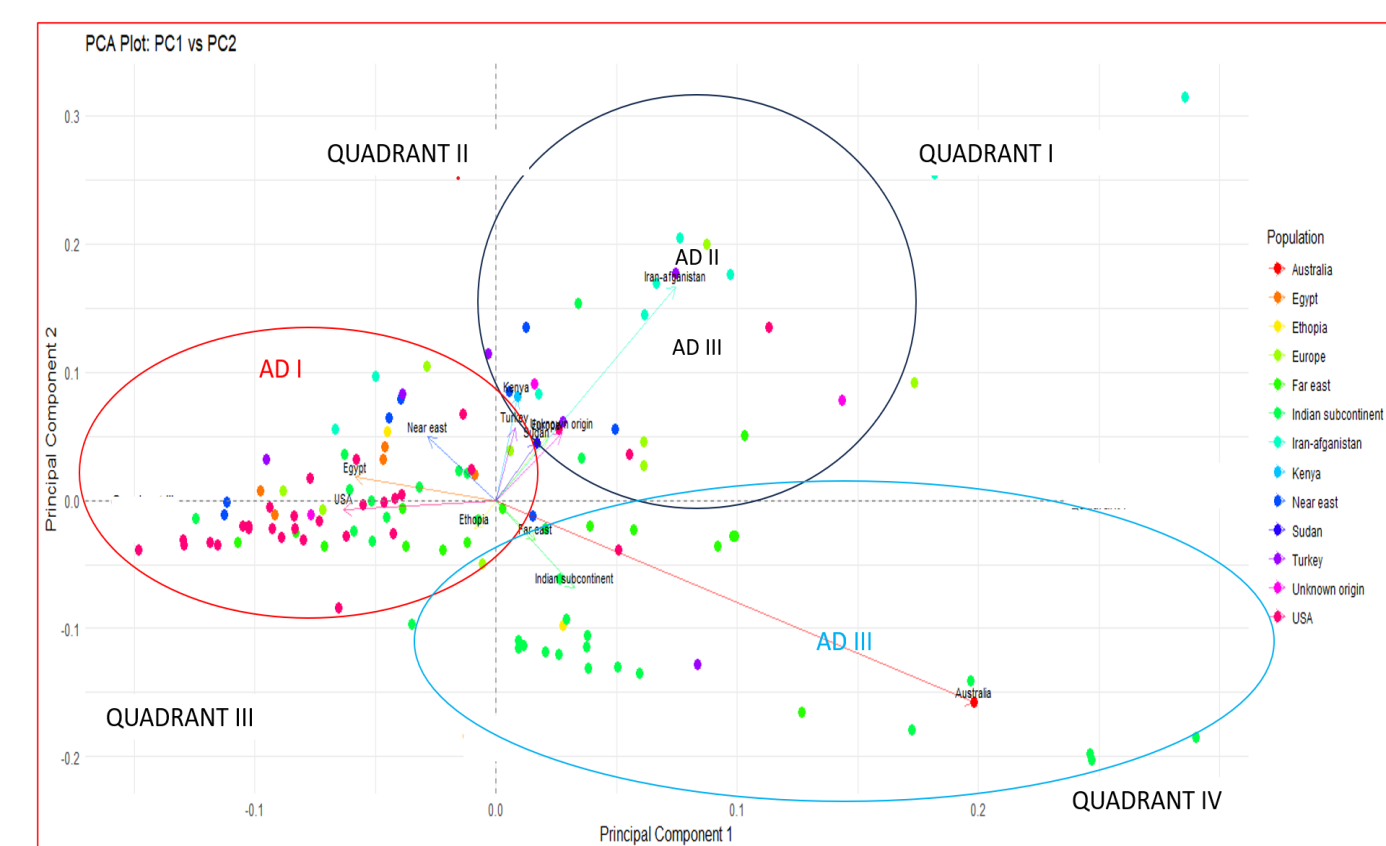


a

b


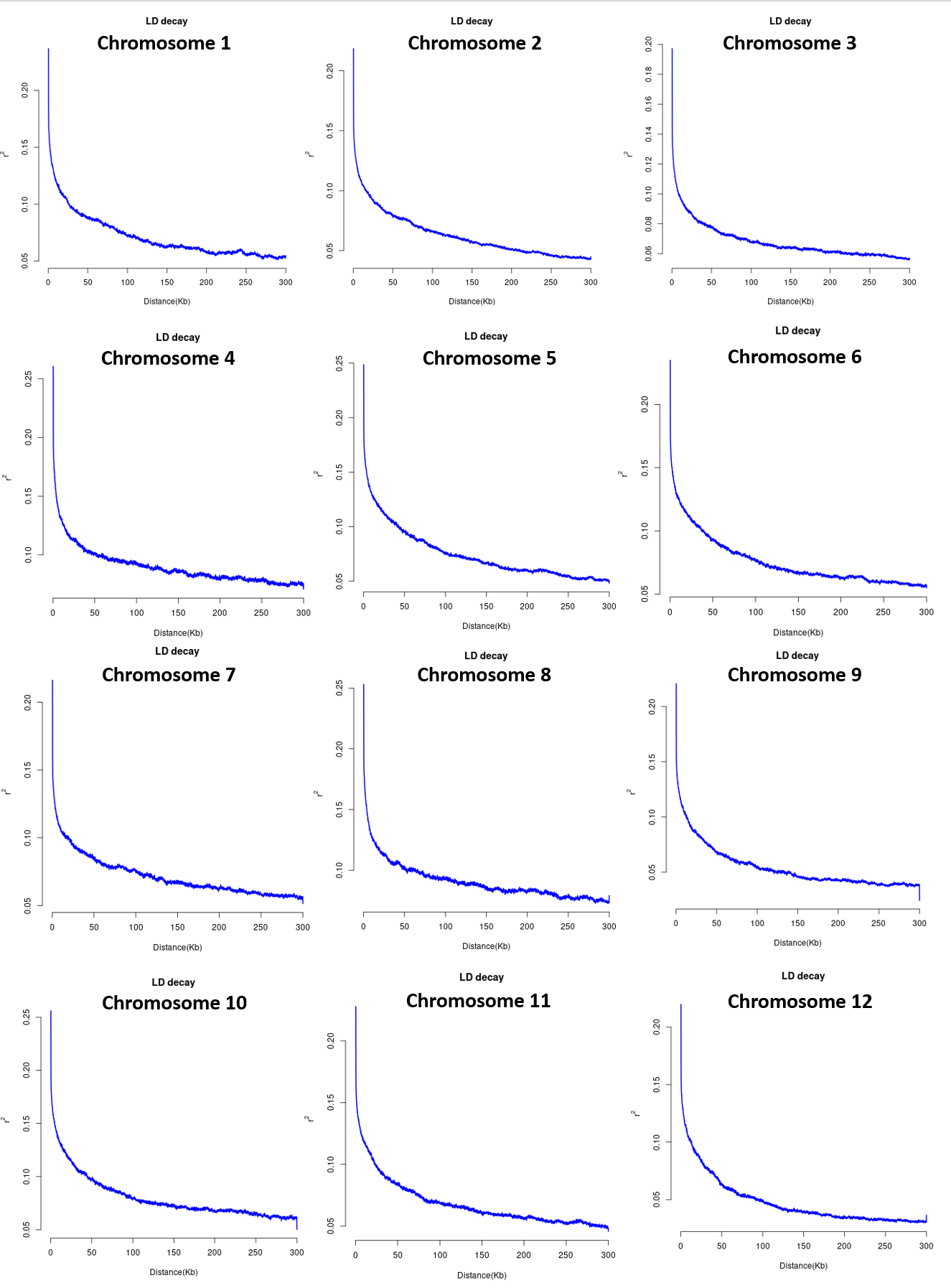


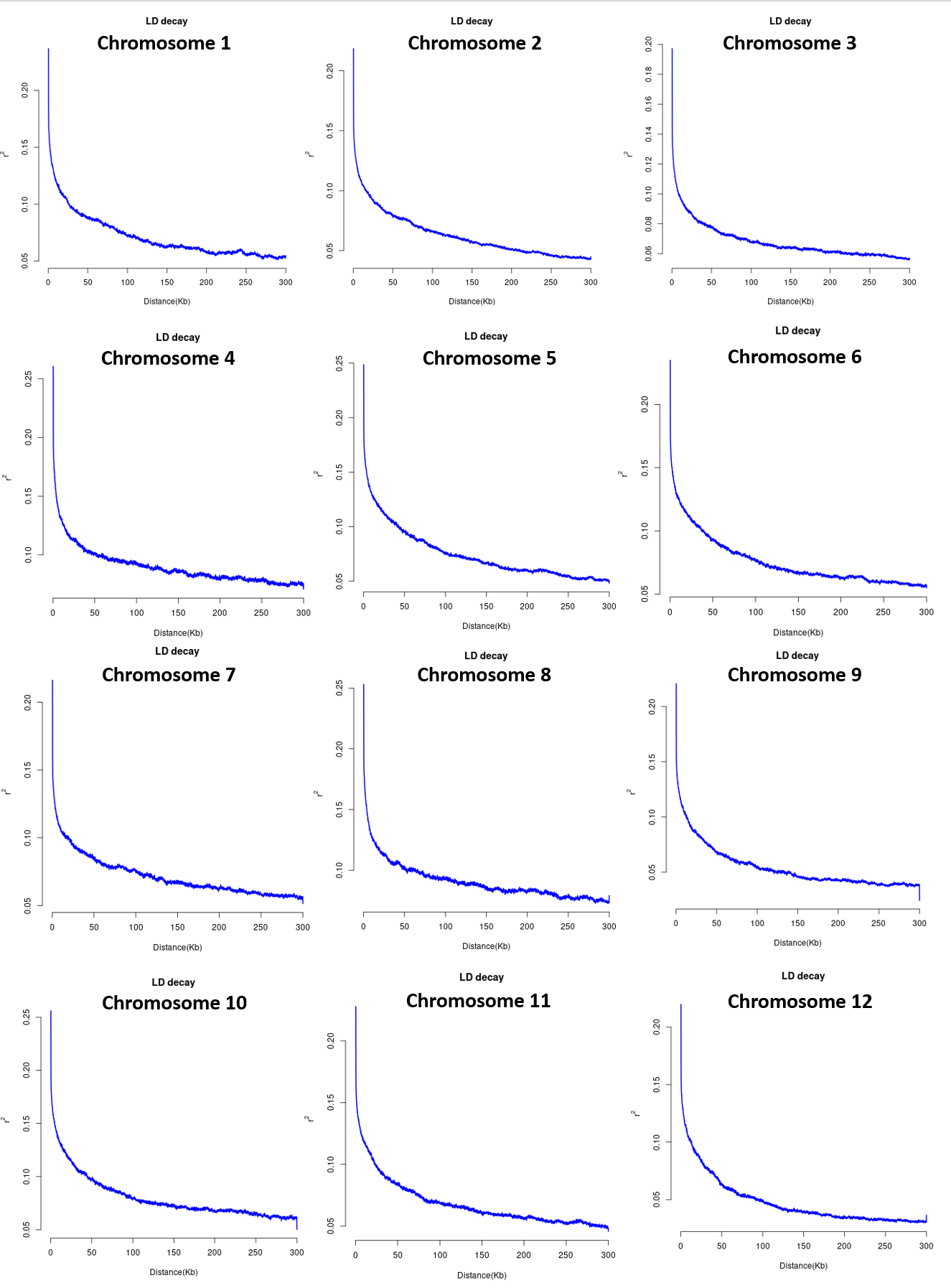


**Supplementary Fig. S14:** (a) Principal component analysis showing distribution of the accession using PC1 and PC2. The circle within the quadrant represents the distribution of the Admixture clusters within the PCA. (b) Linkage disequilibrium along the twelve chromosomes.


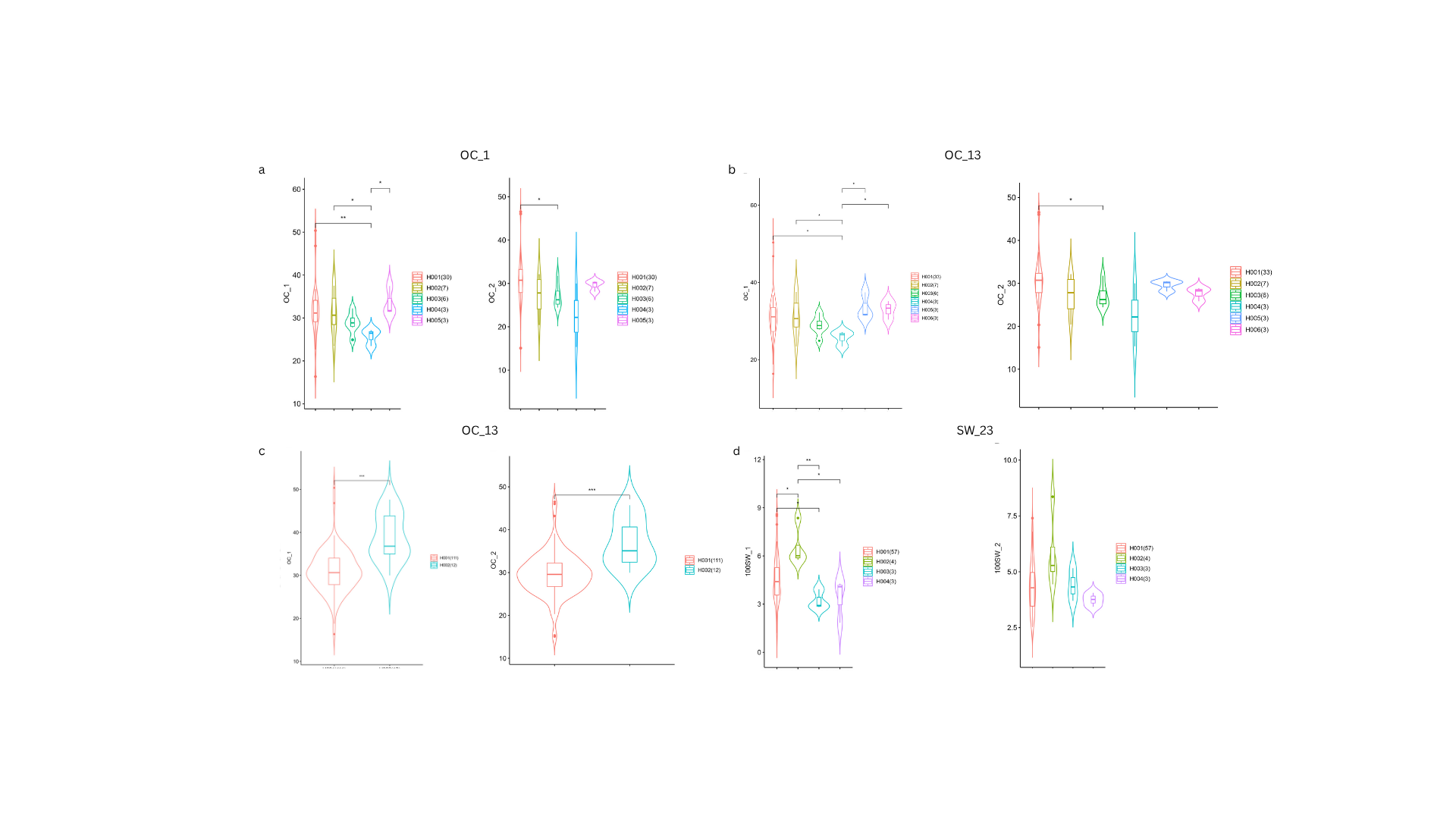


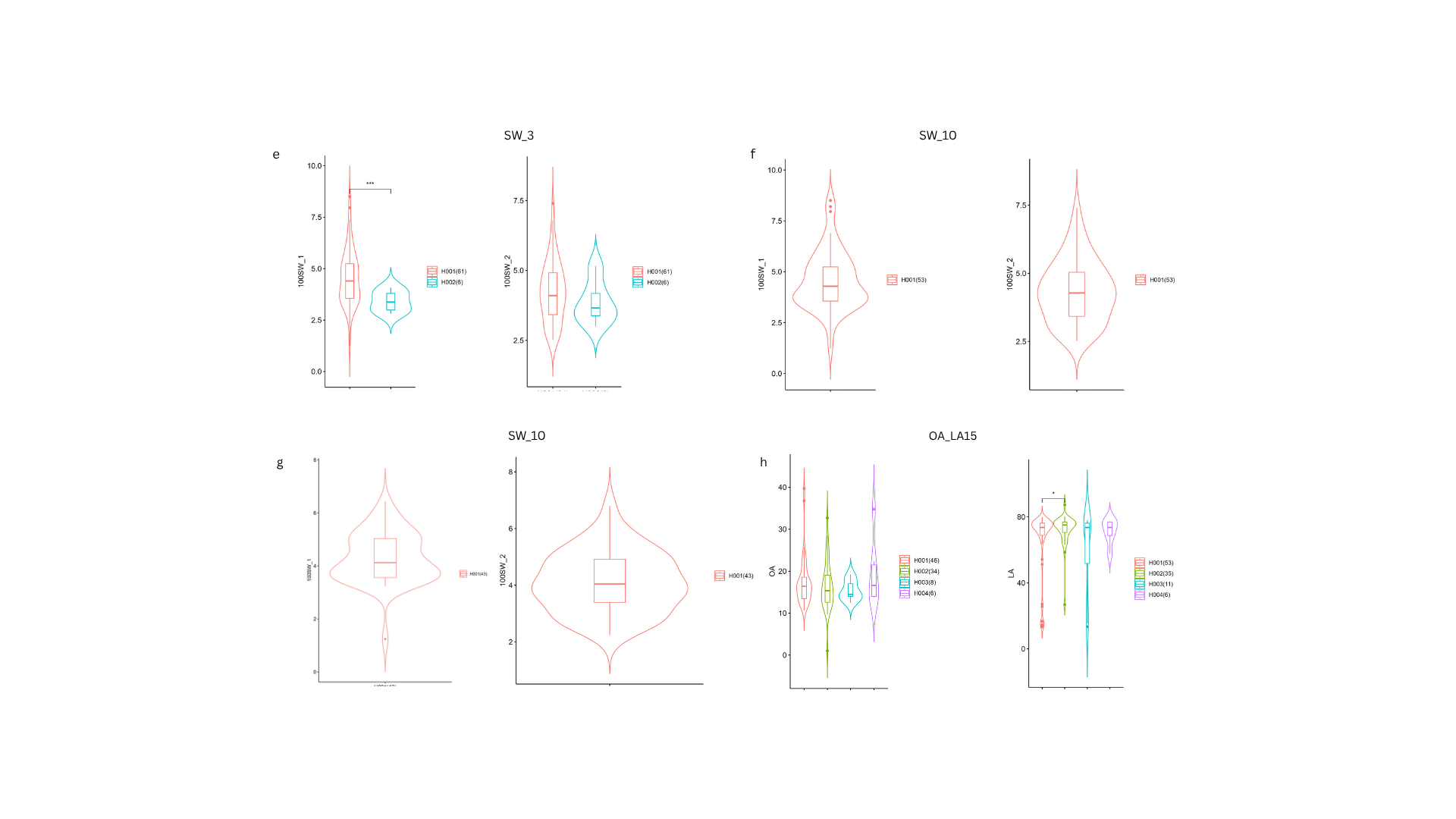


SW_39


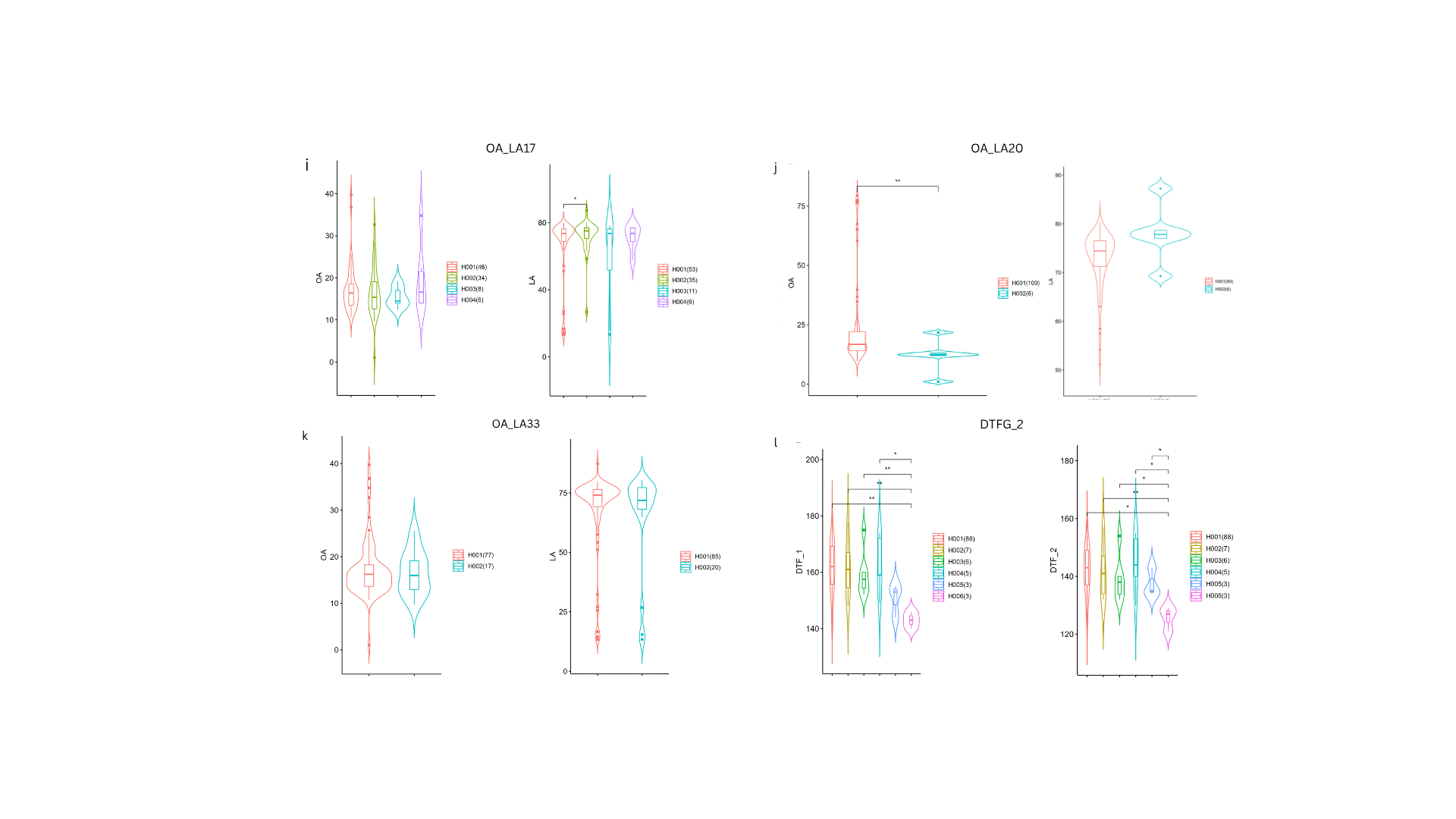


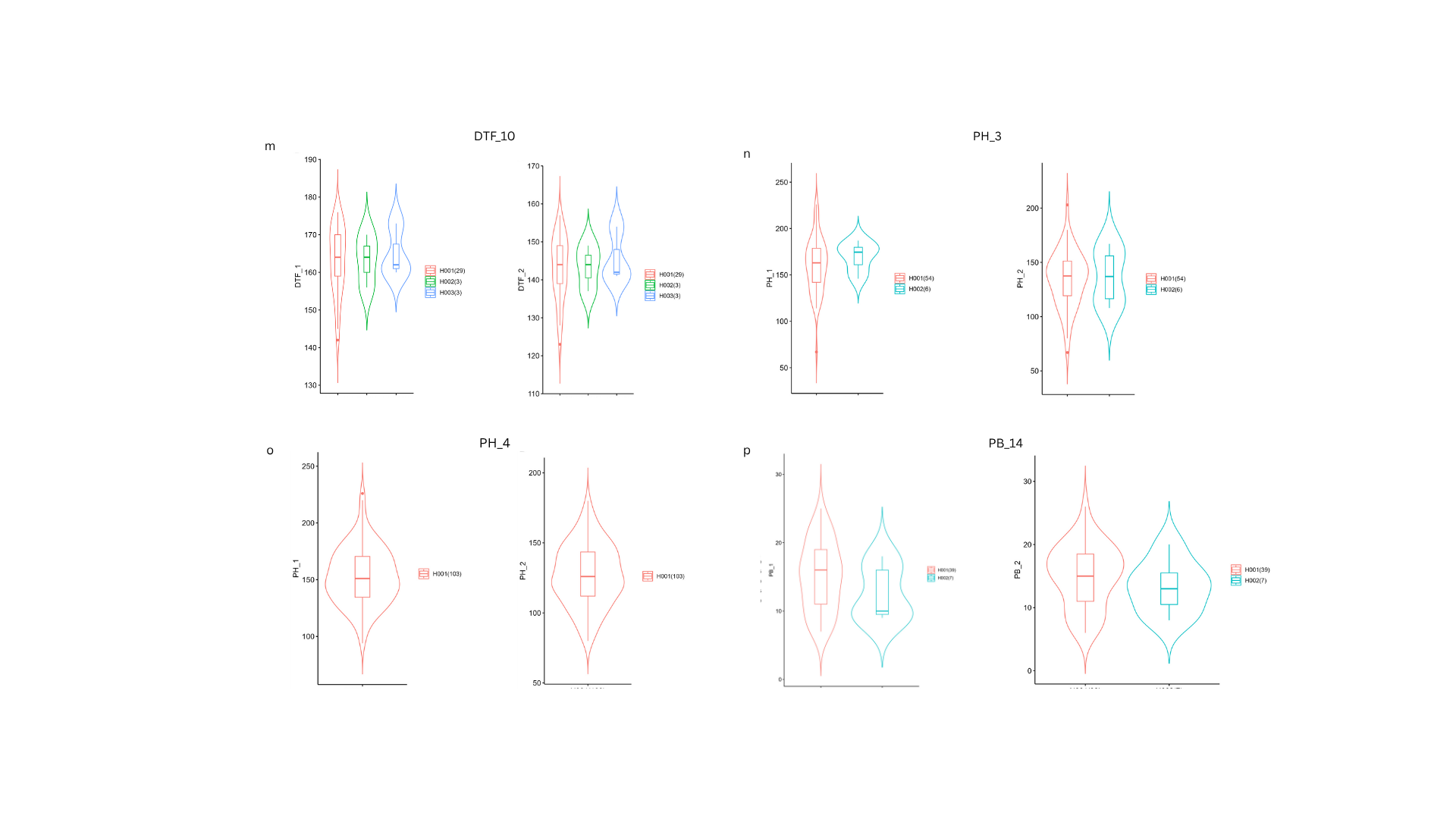


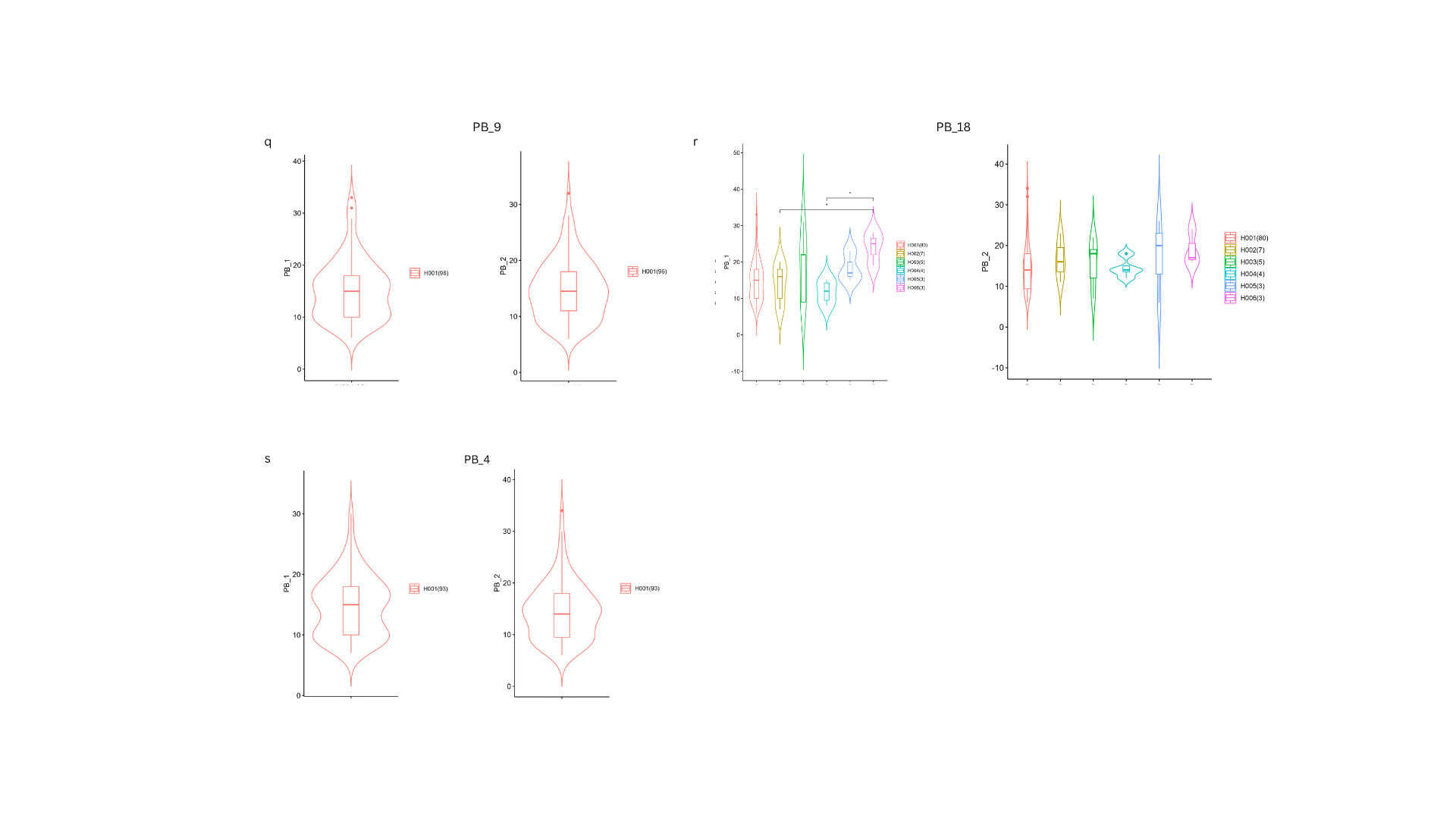


**Supplementary Figure S15:** Haplo-pheno analysis for agronomically important traits depicting QTNs for Oil Content (OC) (a,b,c), 100 Seed weight (SW) (d,e,f,g), Oleic acid and Linoleic acid (OA-LA) (h,i,j,k), Days to 50% flowering (DTF) (l,m), Plant height (PH) (n,o), Primary branches (PB) (p,q,r)


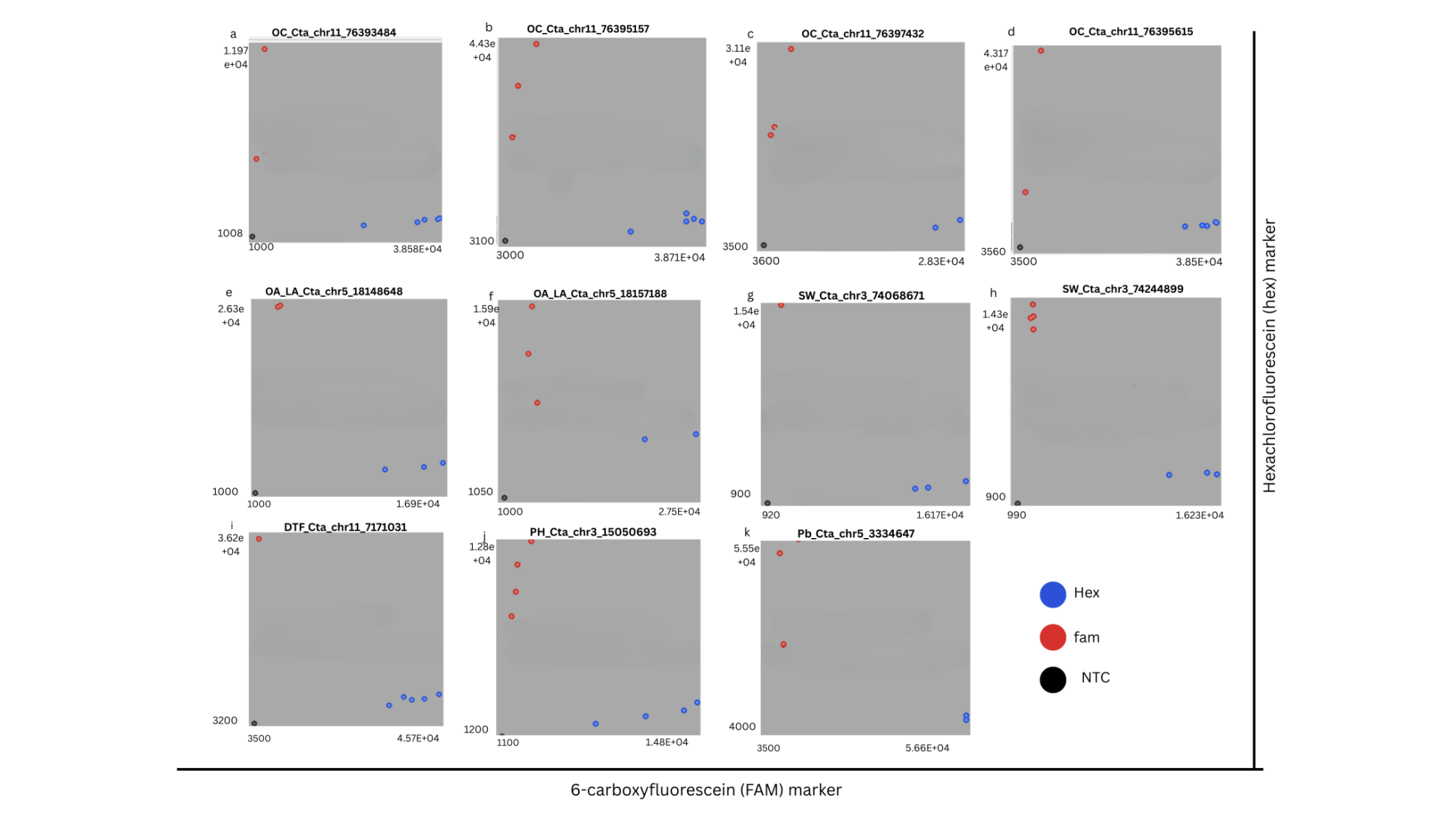


**Supplementary Figure S16:** Validated SNP markers through KASP associated with different traits (a-d) oil content, (e-f) Oleic acid and linoleic acid (g-h) Hundred seed weight (i) Days to 50% flowering (j) plant height and (k) Primary branches.


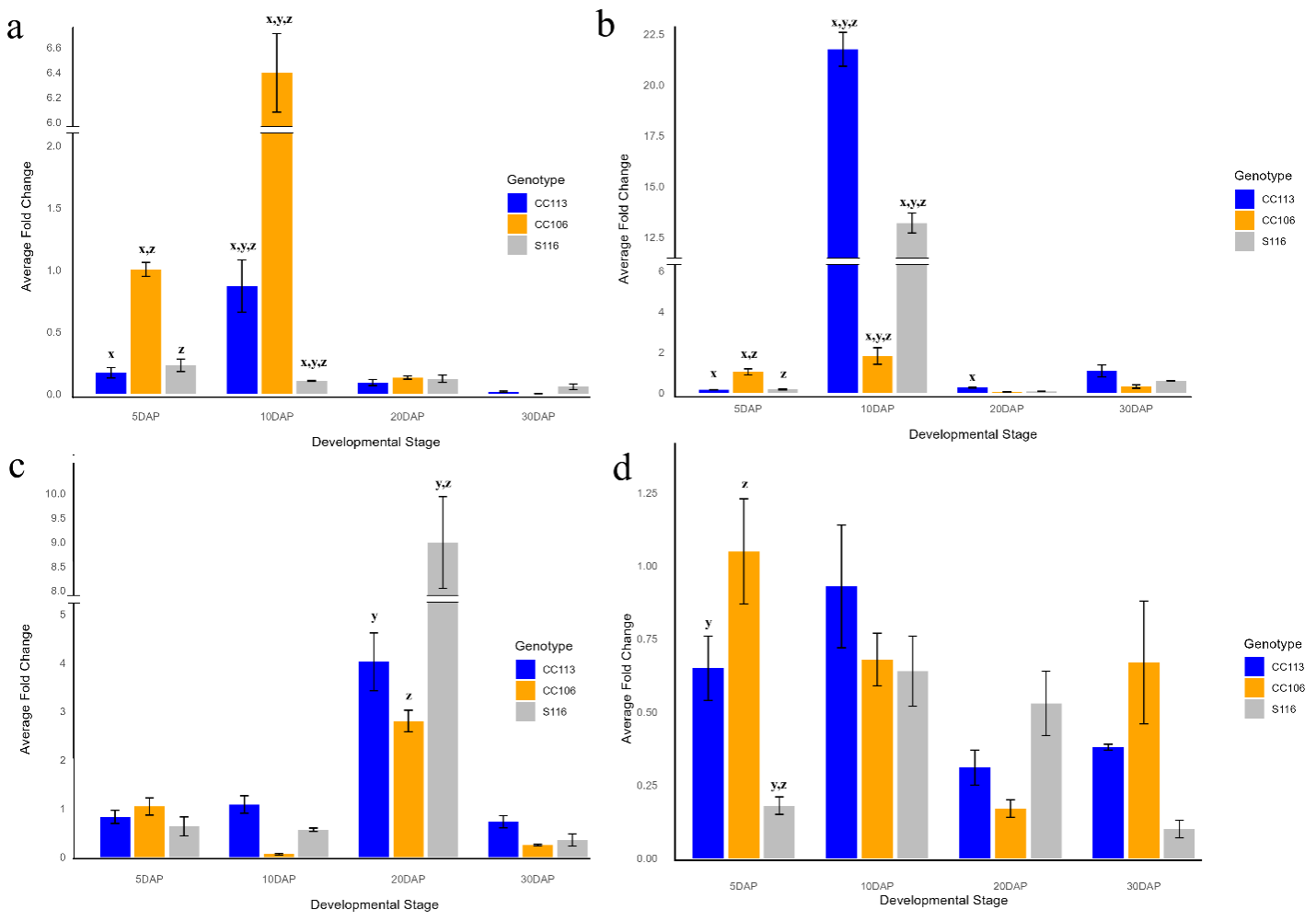


**Supplementary Fig. S17:** Graphs showing the relative expression of candidate genes in core collection accessions CC113, CC106, and S116 for (a) OC (g16872: myosin-binding protein), (b) OC(g64666: probable UDP-N-acetylglucosamine--peptide N-acetylglucosaminyltransferase SPINDLY isoform X1) (c) OA–LA (g43426: cytochrome P450 71A4-like, and (d)SW (g57921: protein FRIGIDA-ESSENTIAL 1-like isoform). Letters x, y, and z above error bars indicate statistically significant differences between CC113 and CC106, CC113 and S116, and CC113 and S116, respectively, as determined by Student’s t-test (p < 0.05).


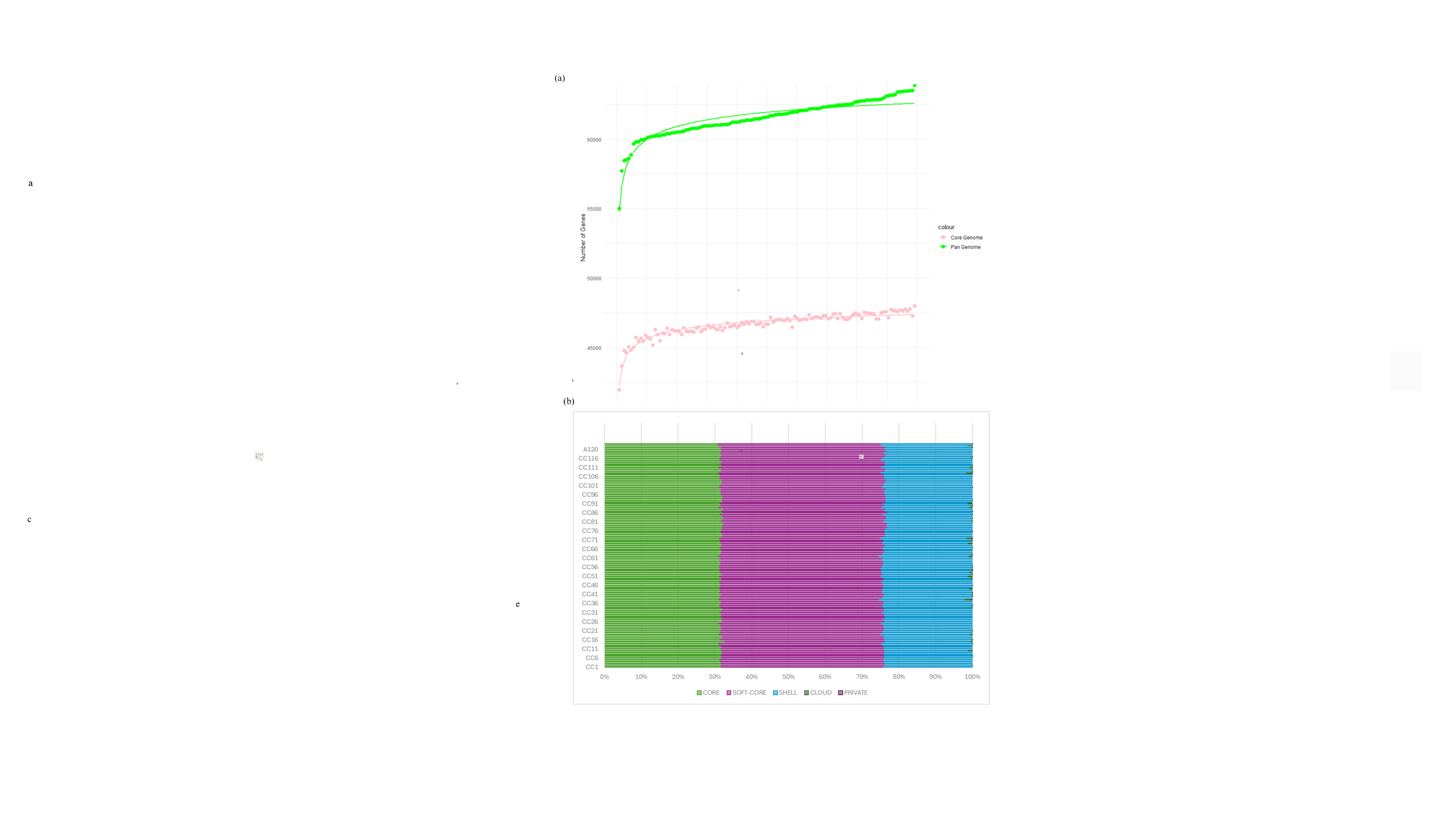


**Supplementary Fig. S18:** Pangenome of Safflower: (a) Modelling of the pan-genome depicting core and pan-genes (b) Distribution of distinct pan-genes amongst core collection accessions

a


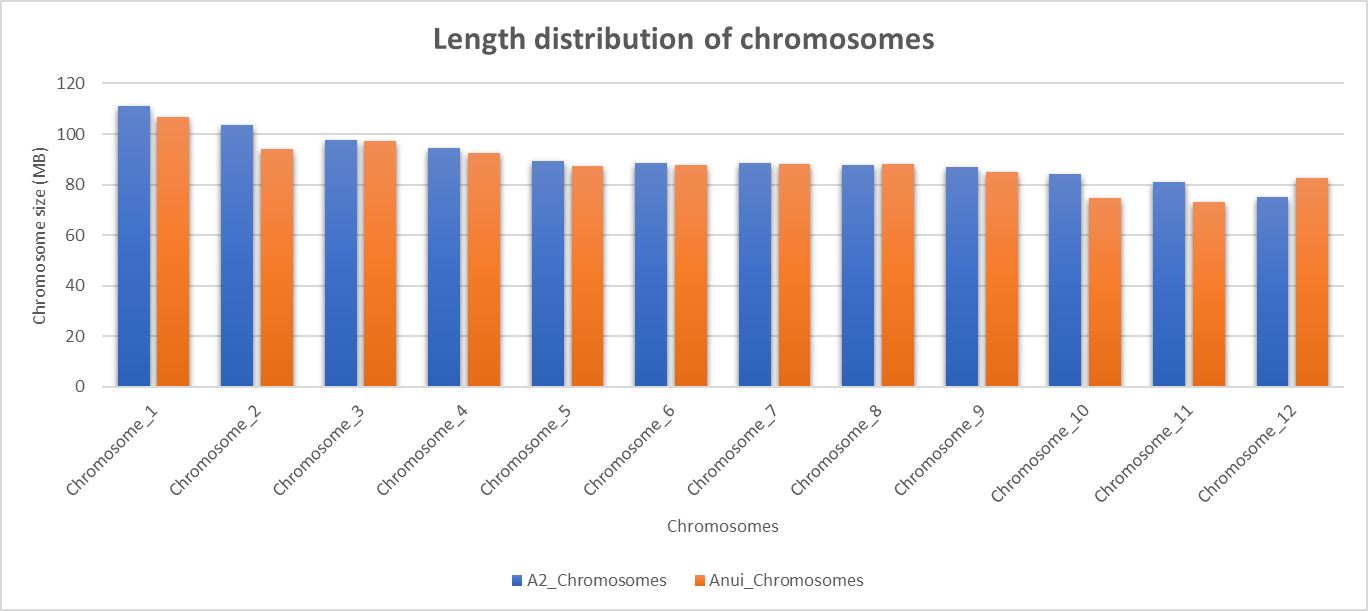

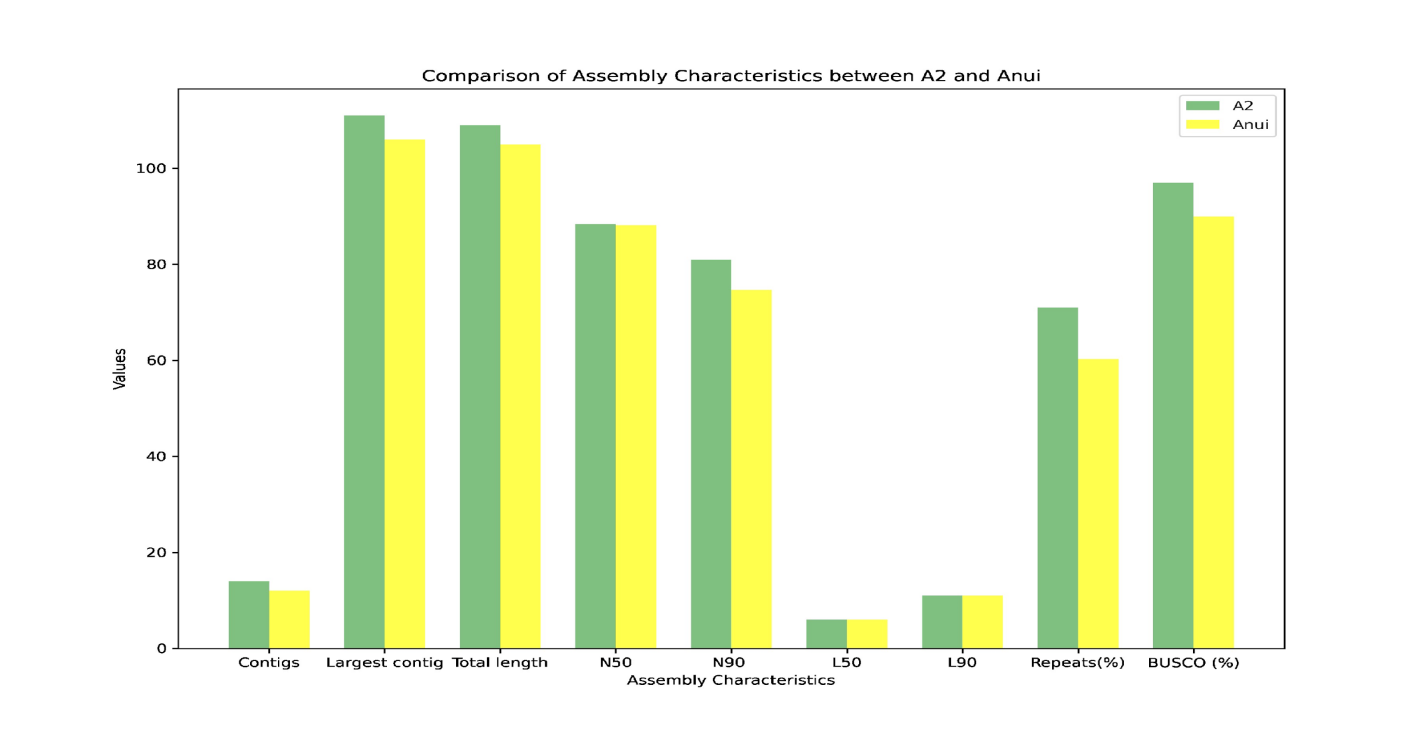


b

**Supplementary Fig. S19:** Comparison of the Safflower_A2 genome assembly with Anhui 1 assemblies (a) Chromosome-wise sequence length comparison with Anhui 1 genome assembly (b) Comparison of assembly characteristics with Anhui 1 genome assembly.

**Supplementary Tables**

**Supplementary Table S1:** Summary table of k-mer distribution at k=17 in the Safflower genome.

| **Raw peak** | **Effective k-mer species** | **Effective k-mer individuals** | **Coverage depth** | **Genome size** |
| --- | --- | --- | --- | --- |
| 27 | 373280371 | 32056342141 | 27.4315 | 1168600000 |

**Supplementary Table S2:** Summary table of different datasets used in the assembly of reference genome sequence.

| **Sequencing platform** | **Insert length** | **Sequencing model** | **Reads number** |
| --- | --- | --- | --- |
| **PacBio HiFi read sequencing** | 13Kb | PacBio Sequel II | 7458042 |
| **Illumina short reads sequencing** | 150bp | Nova seq | 516561866 |
| **Bionano Optical map** | NA | Saphyr | 3497908 |
| **Hi-C pair end link reads sequencing** | NA | Phase Genomics | 487120866 |
| **Iso-Seq** | 1,483 to 1,644 bp | PacBio Sequel II | 3772953 |

**Supplementary Table S3:** Mapping back statistics of the PacBio long reads and Illumina reads to the reference assembly.

| **Feature** | **Number of reads** | **Percentage** |
| --- | --- | --- |
| Illumina short reads | 3419964 | 99.29% |
| PacBio HiFi reads | 495432347 | 95.91% |

**Supplementary Table S4:** Assessment of genome completeness using BUSCO through Eudicot database

| **Feature** | **Number** | **Percentage** |
| --- | --- | --- |
| Complete BUSCOs | 2277 | 97.90% |
| Complete and single-copy BUSCOs | 2164 | 93% |
| Complete and duplicated BUSCOs | 112 | 4.90% |
| Fragmented BUSCOs | 11 | 0.50% |
| Missing BUSCOs | 38 | 1.60% |

**Supplementary Table S5:** Assessment of genome completeness using Merqury.

| **Features** | **Number** |
| --- | --- |
| k-mer set used for measuring completeness | all |
| Solid k-mers in the assembly | 485020450 |
| Total solid k-mers in the read set | 496281072 |
| Completeness (%) | 97.731 |
| QV | 68.94 |

**Supplementary Table S6:** Filters applied to GBS SNP file, filtering criteria and number of variants retained after each step.

| **S. No.** | **Filter Applied** | **Filtration Criteria** | **Remaining number of Variants** |
| --- | --- | --- | --- |
| 1 | Raw Variants |  | 1664500 |
| 2 | Remove Indels | Remove Indels | 1499453 |
| 3 | Read Depth | >=3 | 1499453 |
| 4 | SNP Quality | >=20 | 793204 |
| 5 | Maximum and Minimum no. of alleles | 2,2 | 789701 |
| 6 | Minor Allele Frequency | >=0.05 | 430455 |
| 7 | Data Missingness | <=50% | 82037 |

**Supplementary Table S7**: Summary table for high-density SNP linkage map.

| **Linkage group** | **Number of SNP Markers** | **Linkage group length (cM)** | **Marker interval**  **(No. of markers/cM)** |
| --- | --- | --- | --- |
| 1 | 2159 | 209.0892043 | 10.32573636 |
| 2 | 2117 | 153.1117421 | 13.82650326 |
| 3 | 789 | 137.938027 | 5.71996002 |
| 4 | 967 | 137.0689479 | 7.054843675 |
| 5 | 521 | 142.8017946 | 3.648413533 |
| 6 | 1843 | 194.2938879 | 9.485630352 |
| 7 | 410 | 71.41111908 | 5.741402813 |
| 8 | 3587 | 144.850694 | 24.76342986 |
| 9 | 295 | 65.70961464 | 4.489449552 |
| 10 | 217 | 83.78798318 | 2.589870191 |
| 11 | 1916 | 158.3894757 | 12.09676332 |
| 12 | 911 | 82.59754572 | 11.0293834 |
| Total | 15732 | 1581.050036 | 9.950349224 |

**Supplementary Table S8:** Circular consensus sequencing counts per sample for Iso-Seq data.

| **Sample Name** | **Barcode Name** | **Number of bases sequenced** | **Number of CCS reads** | **Length Distribution** |
| --- | --- | --- | --- | --- |
| A2 5DAP | bc1001 | 813119227 | 531517 | 52-8733 |
| A2 10DAP | bc1002 | 722799568 | 423606 | 53-11624 |
| A2 20DAP | bc1003 | 1021570176 | 666098 | 66-9032 |
| A2 30DAP | bc1004 | 858629410 | 564399 | 53-8261 |
| A2 leaf | bc1005 | 975589410 | 566883 | 56-9280 |
| A2 shoot | bc1006 | 666027617 | 359645 | 53-9885 |
| A2 root | bc1012 | 638202793 | 395693 | 71-10616 |
| A2 Flower and Bud | bc1023 | 443906403 | 265112 | 87-14476 |

**Supplementary Table S9:** Characterization of Alternative Splicing (AS) events by SUPPA2.
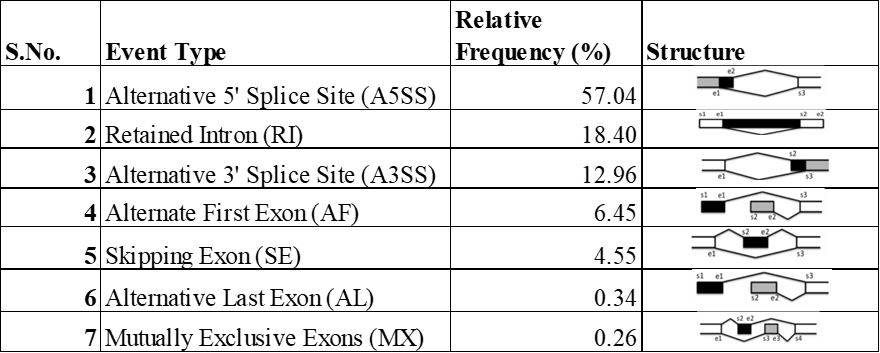


**Supplementary Table S10:** Annotation of repetitive elements in the Safflower_A2 genome.

| **Features** | **Class** | **Length** | **Percentage** |
| --- | --- | --- | --- |
| Class I: Retrotransposons |  |  |  |
| Long terminal repeat (LTR) | Copia | 231764118 | 21.24% |
|  | Gypsy | 243449148 | 22.32% |
| Non-Long terminal repeat (non-LTR) | LINEs | 9555581 | 0.87% |
|  | SINEs | 36460 | 0.00% |
| Class II: DNA Retrotransposons |  |  |  |
| Terminal inverted repeat (TIR) transposons | CACTA | 15057995 | 1.38% |
|  | Mutator | 62707685 | 5.75% |
| Miniature inverted repeat transposable element (MITES) | PIF/Harbinger | 28863650 | 2.65% |
|  | Tc1/Mariner | 3899027 | 0.36% |
|  | hAT | 50194918 | 4.60% |
| Helitron |  | 31449858 | 2.88% |
| Unknown | - | 110777917 | 10.00% |
| Small RNA | - | 4585902 | 0.42% |
| Satellites | - | 713833 | 0.07% |
| Simple repeats | - | 9402360 | 0.86% |
| Low complexity | - | 1179035 | 0.11% |
| Single sequence repeats |  | - | 0.38% |

**Supplementary Table S11:**  Summary table comprises of total LTR-RT elements and their sub families.

| Lineage | Original count | Number of elements with all domains | Number of elements with PBS and TSD | Transposable elements mean length | LTR mean length |
| --- | --- | --- | --- | --- | --- |
| Ty1/Copia | | | | | |
| Ale | 13024 | 560 | 201 | 4696 | 150 |
| Alesia | 63 | 3 | 3 | 4896 | 346 |
| Angela | 17129 | 19 | 7 | 8414 | 989 |
| Bianca | 2260 | 80 | 30 | 6130 | 266 |
| Ikeros | 987 | 42 | 21 | 6354 | 446 |
| Ivana | 15403 | 109 | 66 | 4700 | 258 |
| SIRE | 65067 | 2386 | 1742 | 11565 | 595 |
| TAR | 2573 | 118 | 2 | 5993 | 702 |
| Tork | 2317 | 180 | 123 | 5545 | 642 |
| **Total** | 118823 | 3497 | 2195 | NA | NA |
| Ty3/Gypsy | | | | | |
| Chromovirus\|CRM | 2767 | 80 | 53 | 6643 | 800 |
| Chromovirus\|Reina | 2308 | 95 | 81 | 5266 | 316 |
| Chromovirus\|Tekay | 71143 | 987 | 550 | 11231 | 2678 |
| Non-chromovirus\|OTA\|Athila | 10756 | 636 | 2 | 12814 | 1564 |
| Non-chromovirus\|OTA\|Tat\|Retand | 28035 | 2918 | 2193 | 10222 | 840 |
| **Total** | 115009 | 4716 | 2879 | NA | NA |

***PBS: Primer binding site; TSD: Target site Duplications**

**Supplementary Table S12:** Chromosomal distribution of predicted rRNA and tRNA genes

| **Chromosome** | **rRNA** | **tRNA** |
| --- | --- | --- |
| **1** | 0 | 43 |
| **2** | 1 | 54 |
| **3** | 361 | 44 |
| **4** | 0 | 35 |
| **5** | 0 | 61 |
| **6** | 426 | 56 |
| **7** | 0 | 31 |
| **8** | 75 | 28 |
| **9** | 280 | 27 |
| **10** | 1 | 62 |
| **11** | 0 | 45 |
| **12** | 7 | 48 |
| **Remaining contigs** | 3612 | 576 |

**Supplementary Table S13:** Completeness assessment of the proteins in Safflower_A2 genome using BUSCO through Eudicot database.

| **Feature** | **Number** | **Percentage** |
| --- | --- | --- |
| Complete BUSCOs | 2129 | 91.5% |
| Complete and single-copy BUSCOs | 1767 | 76% |
| Complete and duplicated BUSCOs | 362 | 15.6% |
| Fragmented BUSCOs | 34 | 1.5% |
| Missing BUSCOs | 163 | 7% |

**Supplementary Table S14**: Number of annotated transcripts in Safflower_A2 genome against various publicly available databases.

| **Database** | **Number of transcripts annotated** |
| --- | --- |
| NCBI RefSeq | 46,374 |
| InterProScan | 39,742 |
| KOG | 33,057 |
| KEGG | 16,236 |
| Enzyme Code | 9,518 |

**Supplementary Table S15:** Major classes of Resistance Gene Analogues (RGAs) in Safflower_A2 based on the domain, their count, percentage and composition.

| Domains | Abbreviation | Count | Percent |
| --- | --- | --- | --- |
| **Single domain** | | | |
| Transmembrane | TRAN | 8 | 0.050128 |
| Toll/interleukin-1 receptor | T | 155 | 0.971239 |
| Receptor-Like Protein | RLP | 1447 | 9.066984 |
| Receptor-Like Kinase | RLK | 3076 | 19.27439 |
| Nucleotide-binding site | N | 517 | 3.239551 |
| Leucine-rich repeat | L | 108 | 0.676734 |
| Kinase | KIN | 6020 | 37.72166 |
| Cytoplasmic Kinase | CK | 563 | 3.684441 |
| Coiled Coil | C | 3 | 0.025064 |
| **Double domains** | | | |
| Transmembrane Kinase | TK | 41 | 0.256908 |
| Toll/interleukin-1 receptor- Nucleotide-binding site | TN | 518 | 3.245817 |
| Toll/interleukin-1 receptor- Leucine-rich repeat | TL | 4 | 0.025064 |
| Nucleotide-binding site- Leucine-rich repeat Kinase | NLK | 23 | 0.144119 |
| Nucleotide-binding site- Leucine-rich repeat | NL | 641 | 4.016542 |
| Nucleotide-binding site Kinase | NK | 5 | 0.03133 |
| Cytoplasmic Leucine-rich repeat Kinase | CLK | 118 | 0.739395 |
| Cytoplasmic Leucine-rich repeat | CL | 38 | 0.250642 |
| Coiled Coil-Toll/interleukin-1 receptor | CT | 11 | 0.068927 |
| Coiled Coil- Nucleotide-binding site Kinase | CNK | 12 | 0.075193 |
| Coiled Coil- Nucleotide-binding site | CN | 320 | 2.01767 |
| **Triple domains** | | | |
| Coiled Coil- Nucleotide-binding site-Toll/interleukin-1 receptor | CNT | 38 | 0.23811 |
| Coiled Coil- Nucleotide-binding site- Leucine-rich repeat | CNL | 568 | 3.565386 |
| Toll/interleukin-1- Nucleotide-binding site- Leucine-rich repeat | TNL | 1503 | 9.417883 |
| **Four domains** | | | |
| Coiled Coil-Toll/interleukin-1 receptor- Nucleotide-binding site- Leucine-rich repeat | CTNL | 172 | 1.084028 |

**Supplementary Table S16:** Functional annotation of the 236 NLRs and their association in diseases in different plants.

| S.no | Protein | Number of genes | Function |  | Pathogen associated | Associated disease in Safflower | References |
| --- | --- | --- | --- | --- | --- | --- | --- |
| 1 | At4g11170 | 4 | Encodes for Resistance methylated gene 1 | *Arabidopsis thaliana*; *Pistacia*; Common bean | *Fusarium oxysporum;*  *Phytophthora parsiana* | *Fusarium* wilt in safflower; *Phytophthora* root rot disease in safflower | (Hajabdollahi et al., 2021; Zhu et al., 2013) |
| 2 | RGA3 | 6 | RGA3 likely triggers a series of signalling events, leading to the activation of defence-related genes, such as those encoding pathogenesis-related proteins. | Rice, sugarcane, Banana, common bean | Fusarium fujikuroi *Colletotrichum falcatum* | *Fusarium wilt* | (Kanazin et al., 1996; López et al., 2003; Saile et al., 2020; Sutanto et al., 2014; Thakur et al., 2024; Xu et al., 2014) |
| 3 | Recognition of XopQ 1  (Roq1-like isoform X2) | 2 | Recognizes bacterial effectors like XopQ, HopQ1 | *Nicotiana benthamiana*, *Solanum lycopersicum* (Tomato) | *Xanthomonas, Pseudomonas, Ralsotonia* | Head rot; Bacterial leaf and stem blight of safflower | (Athanasopoulos et al., 2010; Schultink et al., 2017; Thomas et al., 2020) |
| 4 | Recognition of Peronospora parasitica 13-like (RPP13) | 23 | Interacting with WPP1 contributes to quantitative and/or basal resistance. | Wheat; Nicotiana; Potato; Barley; Arabisopsis | *Blumeria graminis, Puccinia striiformis; Meloidogyne incognita* | Safflower rust | (Dong et al., 2021; Yuan et al., 2025, p. 202; Zhang et al., 2022) |
| 5 | Resistance to *Plasmopara viticola* 1 (RPV1); | 15 | Autoactive signaling function and cell-death signaling | Grapevine, Soyabean, Arabidopsis thaliana | *Plasmopara viticola; Potyvirus* | Turnip mosaic viruses in safflower (Potyvirus); Cucumber mosaic virus in safflower | (Calonnec et al., 2008; Gore, 2000; Gore et al., 2002; Poque et al., 2015; Williams et al., 2016) |
| 6 | Resistance to Ralstonia solanacearum 1 (RRS1-like) | 1 | RRS1-R detects specific pathogen effectors most; notably the PopP2 effector from the soil-borne bacterium | Arabidopsis; Brassica | *Pseudomonas,* *Ralsotonia* | Bacterial leaf and stem blight of safflower | (Guo et al., 2020; Ma et al., 2018; Saucet et al., 2015) |
| 7 | Resistance to Uncinula necator 1 (RUN1) | 3 | Recognizes specific effectors from the powdery mildew fungus , leads to a hypersensitive response and localized cell death | Grapevine | *Erysiphae nector* | Powdery mildew pathogen of safflower | (Agurto et al., 2017; Barker et al., 2005; Feechan et al., 2015; Massonnet et al., 2022) |
| 8 | Dominant Suppressor of CAMTA3-1 (DSC1) | 14 | Work with WRKY’s to provide basal plants immunity | *Arabidopsis thaliana.*  Cotton | *Verticillium dahlia*; Meloidogyne incognita | *Verticillium* wilt of safflower.  reduced growth upon attack of the M. incognita | (T. Li et al., 2019; Warmerdam et al., 2020) |
| 9 | Probable At5g66900 | 7 | Encodes for  Helper *NLR.1.1* called NRG1  NRG1.1 facilitates defence responses, including the hypersensitive response | *Arabidopsis thaliana.*  *Populus trichocarpa* | *Alternaria* | Leaf blight disease in safflower | (Chini & Loake, 2005; Kohler et al., 2008; Peele et al., 2014; Saile et al., 2020; Tan et al., 2007) |
| 10 | Resistance to Peronospora parasitica 8 (RPP8-like protein 2) | 1 | Induces by pathogens identification and salicylic acid.  regulated expression through WRKY transcription factors | Tobacco, *Arabidopsis thaliana*,  Vitis, Roselle, Lotus, Capsicum | *Potato virus Y*, Turnip crinkle virus, *Phytophthora* | Turnip mosaic viruses in safflower (Potyvirus); *Phytoph*ora root rot disease in safflower | (Jia et al., 2013; Y.-F. Li et al., 2021; Michel et al., 2018; Yong & Atheeqah-Hamzah, 2024) |
| 11 | putative At3g14460 | 6 | Encodes for adenylyl cyclase catalytic core motif against biotrophic and hemibiotrophic pathogens | *Arabidopsis thaliana* | *Golovinomyces orontii, Pseudomonas syringae; Magnaporthe oryzae* | Bacterial leaf and stem blight of safflower. | (Alqurashi, 2013; Bianchet et al., 2019; Tsele, 2018) |
| 12 | TMV resistance protein N-like | 121 | Recognizes specific viral effectors or viral RNA that are produced during infection | Tobacco, potato, common bean, cotton | *Synchytriumendobioticum*  *Tobacco mosaic virus* | NA | (Hehl et al., 1999; Kong et al., 2025; Qin et al., 2015) |
| 13 | RML1A-like | 11 | Broader disease resistance strategy; Involve in hype response of the plant | Lettuce, Brassica, Soyabean | *Verticillium longisporum; S*oyabean mosaic virus, | *Verticillium* wilt of safflower | (Raza et al., 2025; Tsai et al., 2020; Wang et al., 2024) |

**Supplementary Table S17**: Accessions of core collections, their geographic distribution and resequencing data.

| **Sample label** | **Accession** | **Geographical location** | **PI Number** | **No. of reads** | **No. of bases** | **Data (Gb)** | **Coverage** |
| --- | --- | --- | --- | --- | --- | --- | --- |
| **Core collection** | | | | | | | |
| CC1 | 1(8) | Australia | 242419 | 108667970 | 16408863470 | 16.41 | 15.02 |
| CC2 | 2(3) 56 | Far east | 514618 | 108401468 | 16368621668 | 16.37 | 14.98 |
| CC3 | 2(3) 58 | Far east | 514619 | 102325600 | 15451165600 | 15.45 | 14.14 |
| CC4 | 2(3) 61 | Far east | 514622 | 128471082 | 19399133382 | 19.40 | 17.75 |
| CC5 | 2(3) 76 | Far east | 543974 | 128692638 | 19432588338 | 19.43 | 17.78 |
| CC6 | 2(3) 79 | Far east | 543977 | 103084146 | 15565706046 | 15.57 | 14.25 |
| CC7 | 2(3) 84 | Far east | 543981 | 112873962 | 17043968262 | 17.04 | 15.60 |
| CC8 | 2(3) 96 | Far east | 543993 | 91206534 | 13772186634 | 13.77 | 12.60 |
| CC9 | 2(3) 109 | Far east | 544007 | 88139626 | 13309083526 | 13.31 | 12.18 |
| CC10 | 2(3) 115 | Far east | 544011 | 124192210 | 18753023710 | 18.75 | 17.16 |
| CC11 | 2(3) 134 | Far east | 544029 | 90200660 | 13620299660 | 13.62 | 12.47 |
| CC12 | 2(3) 136 | Far east | 544031 | 93942808 | 14185364008 | 14.19 | 12.98 |
| CC13 | 2(3) 138 | Far east | 544033 | 131895472 | 19916216272 | 19.92 | 18.23 |
| CC14 | 2(3) 145 | Far east | 544040 | 133068966 | 20093413866 | 20.09 | 18.39 |
| CC15 | 2(3) 149 | Far east | 544043 | 64581014 | 9751733114 | 9.75 | 8.92 |
| CC16 | 2(3) 161 | Far east | 544055 | 143757842 | 21707434142 | 21.71 | 19.87 |
| CC17 | 2(6) 381 | Indian subcontinent | 401470 | 83042284 | 12539384884 | 12.54 | 11.48 |
| CC18 | 2(6) 384 | Indian subcontinent | 401473 | 105428448 | 15919695648 | 15.92 | 14.57 |
| CC19 | 2(6) 387 | Indian subcontinent | 401476 | 110284616 | 16652977016 | 16.65 | 15.24 |
| CC20 | 2(6) 388 | Indian subcontinent | 401477 | 137335622 | 20737678922 | 20.74 | 18.98 |
| CC21 | 2(6) 389 | Indian subcontinent | 401478 | 102868102 | 15533083402 | 15.53 | 14.22 |
| CC22 | 2(6) 390 | Indian subcontinent | 401479 | 100386776 | 15158403176 | 15.16 | 13.87 |
| CC23 | 2(6) 392 | Indian subcontinent | 470942 | 114173092 | 17240136892 | 17.24 | 15.78 |
| CC24 | 2(7) 699 | Indian subcontinent | 283764 | 110933196 | 16750912596 | 16.75 | 15.33 |
| CC25 | 2(7) 771 | Indian subcontinent | 305193 | 118994110 | 17968110610 | 17.97 | 16.44 |
| CC26 | 2(7) 780 | Indian subcontinent | 305204 | 94234718 | 14229442418 | 14.23 | 13.02 |
| CC27 | 2(7) 793 | Indian subcontinent | 305218 | 97934498 | 14788109198 | 14.79 | 13.53 |
| CC28 | 2(7) 803 | Indian subcontinent | 306825 | 99355298 | 15002649998 | 15.00 | 13.73 |
| CC29 | 2(7) 813 | Indian subcontinent | 306836 | 104855898 | 15833240598 | 15.83 | 14.49 |
| CC30 | 2(7) 841 | Indian subcontinent | 306866 | 108821694 | 16432075794 | 16.43 | 15.04 |
| CC31 | 2(7) 851 | Indian subcontinent | 306876 | 106932544 | 16146814144 | 16.15 | 14.78 |
| CC32 | 2(7) 866 | Indian subcontinent | 306892 | 96641702 | 14592897002 | 14.59 | 13.36 |
| CC33 | 2(7) 869 | Indian subcontinent | 306897 | 96641702 | 14592897002 | 14.59 | 13.36 |
| CC34 | 2(7) 877 | Indian subcontinent | 306906 | 123302946 | 18618744846 | 18.62 | 17.04 |
| CC35 | 2(7) 885 | Indian subcontinent | 306912 | 117088184 | 17680315784 | 17.68 | 16.18 |
| CC36 | 2(7) 887 | Indian subcontinent | 306915 | 104572210 | 15790403710 | 15.79 | 14.45 |
| CC37 | 2(7) 915 | Indian subcontinent | 306944 | 100552278 | 15183393978 | 15.18 | 13.90 |
| CC38 | 2(7) 943 (I) | Indian subcontinent | 306972 | 123729600 | 18683169600 | 18.68 | 17.10 |
| CC39 | 2(7) 965 | Indian subcontinent | 306993 | 124668808 | 18824990008 | 18.82 | 17.23 |
| CC40 | 2(7) 975 | Indian subcontinent | 307001 | 106318218 | 16054050918 | 16.05 | 14.69 |
| CC41 | 2(7) 993 | Indian subcontinent | 307020 | 104358452 | 15758126252 | 15.76 | 14.42 |
| CC42 | 2(7) 1047 | Indian subcontinent | 307067 | 138396460 | 20897865460 | 20.90 | 19.13 |
| CC43 | 2(7) 1125 | Indian subcontinent | 401577 | 111596392 | 16851055192 | 16.85 | 15.42 |
| CC44 | 2(7) 1137 | Indian subcontinent | 401589 | 107602690 | 16248006190 | 16.25 | 14.87 |
| CC45 | 2(8) 1236 | Indian subcontinent | 304408 | 116125706 | 17534981606 | 17.53 | 16.05 |
| CC46 | 2(8) 1241 | Indian subcontinent | 374222 | 116300826 | 17561424726 | 17.56 | 16.07 |
| CC47 | 2(9) 1276 | Iran-Afghanistan | 268374 | 123944776 | 18715661176 | 18.72 | 17.13 |
| CC48 | 2(10) 1291 | Near East | 369845 | 117316956 | 17714860356 | 17.71 | 16.21 |
| CC49 | 2(11) 1296 | Near East | 305537 | 90721958 | 13699015658 | 13.70 | 12.54 |
| CC50 | 3(1) 1385 | Iran-Afghanistan | 304442 | 114077344 | 17225678944 | 17.23 | 15.77 |
| CC51 | 3(1) 1428 | Iran-Afghanistan | 343777 | 113451534 | 17131181634 | 17.13 | 15.68 |
| CC52 | 3(1) 1437 | Iran-Afghanistan | 388902 | 82110626 | 12398704526 | 12.40 | 11.35 |
| CC53 | 3(1) 1441 | Iran-Afghanistan | 388906 | 136154412 | 20559316212 | 20.56 | 18.82 |
| CC54 | 3(1) 1452 | Iran-Afghanistan | 405960 | 134378600 | 20291168600 | 20.29 | 18.57 |
| CC55 | 3(1) 1461 | Iran-Afghanistan | 405968 | 112728254 | 17021966354 | 17.02 | 15.58 |
| CC56 | 3(1) 1463 | Iran-Afghanistan | 405970 | 143825462 | 21717644762 | 21.72 | 19.88 |
| CC57 | 3(1) 1484 | Iran-Afghanistan | 405987 | 120791344 | 18239492944 | 18.24 | 16.69 |
| CC58 | 3(4) 1529 | Near East | 253895 | 114738018 | 17325440718 | 17.33 | 15.86 |
| CC59 | 3(4) 1537 | Near East | 386173 | 93861546 | 14173093446 | 14.17 | 12.97 |
| CC60 | 3(6) 1551 | Near East | 198990 | 140355988 | 21193754188 | 21.19 | 19.40 |
| CC61 | 3(6) 1573 | Near East | 306684 | 113808034 | 17185013134 | 17.19 | 15.73 |
| CC62 | 4(1) 1581 | Europe | 369849 | 153525354 | 23182328454 | 23.18 | 21.22 |
| CC63 | 4(3) 1639 | Turkey | 304503 | 109041498 | 16465266198 | 16.47 | 15.07 |
| CC64 | 4(3) 1651 | Turkey | 340076 | 102160424 | 15426224024 | 15.43 | 14.12 |
| CC65 | 4(3) 1654 | Turkey | 340079 | 96844892 | 14623578692 | 14.62 | 13.38 |
| CC66 | 4(3) 1656 | Turkey | 340081 | 109700524 | 16564779124 | 16.56 | 15.16 |
| CC67 | 4(3) 1674 | Turkey | 392026 | 114454626 | 17282648526 | 17.28 | 15.82 |
| CC68 | 4(3) 1698 | Turkey | 407617 | 104638212 | 15800370012 | 15.80 | 14.46 |
| CC69 | 4(9) 1728 | Europe | 253515 | 118464212 | 17888096012 | 17.89 | 16.37 |
| CC70 | 4(11) 1739 | Europe | 576985 | 123955284 | 18717247884 | 18.72 | 17.13 |
| CC71 | 4(12) 1741 | Europe | 253522 | 118531528 | 17898260728 | 17.90 | 16.38 |
| CC72 | 4(13) 1761 | Europe | 262442 | 112841982 | 17039139282 | 17.04 | 15.59 |
| CC73 | 4(14) 1802 | Europe | 258421 | 85725810 | 12944597310 | 12.94 | 11.85 |
| CC74 | 4(17) 1811 | Europe | 576990 | 102748216 | 15514980616 | 15.51 | 14.20 |
| CC75 | 5(2) 1838 | Ethiopia | 193475 | 103385320 | 15611183320 | 15.61 | 14.29 |
| CC76 | 5(3) 1860 | Kenya | 209300 | 102521166 | 15480696066 | 15.48 | 14.17 |
| CC77 | 5(4) 1831 | Ethiopia | 273876 | 99084686 | 14961787586 | 14.96 | 13.69 |
| CC78 | 5(4) 1871 | Egypt | 250081 | 100731324 | 15210429924 | 15.21 | 13.92 |
| CC79 | 5(4) 1907 | Egypt | 306593 | 97755862 | 14761135162 | 14.76 | 13.51 |
| CC80 | 5(4) 1908 | Egypt | 306594 | 73829264 | 11148218864 | 11.15 | 10.20 |
| CC81 | 5(4) 1911 | Egypt | 306597 | 88281388 | 13330489588 | 13.33 | 12.20 |
| CC82 | 5(4) 1929 | Egypt | 306610 | 94024994 | 14197774094 | 14.20 | 12.99 |
| CC83 | 5(5) 1952 | Sudan | 305527 | 80818870 | 12203649370 | 12.20 | 11.17 |
| CC84 | 5(8) 1973 | Europe | 253560 | 101439482 | 15317361782 | 15.32 | 14.02 |
| CC85 | 6(1) 1977 | USA | 348915 | 125857534 | 19004487634 | 19.00 | 17.39 |
| CC86 | 6(2) 2015 | USA | 537607 | 95737356 | 14456340756 | 14.46 | 13.23 |
| CC87 | 6(2) 2023 | USA | 537614 | 96158750 | 14519971250 | 14.52 | 13.29 |
| CC88 | 6(2) 2029 | USA | 537619 | 96372390 | 14552230890 | 14.55 | 13.32 |
| CC89 | 6(2) 2045 | USA | 537634 | 128341640 | 19379587640 | 19.38 | 17.74 |
| CC90 | 6(2) 2046 | USA | 537635 | 101657384 | 15350264984 | 15.35 | 14.05 |
| CC91 | 6(2) 2053 | USA | 537641 | 113362684 | 17117765284 | 17.12 | 15.67 |
| CC92 | 6(2) 2065 | USA | 537653 | 96940300 | 14637985300 | 14.64 | 13.40 |
| CC93 | 6(2) 2068 | USA | 537656 | 83423272 | 12596914072 | 12.60 | 11.53 |
| CC94 | 6(2) 2070 | USA | 537658 | 106154620 | 16029347620 | 16.03 | 14.67 |
| CC95 | 6(2) 2071 | USA | 537659 | 90827564 | 13714962164 | 13.71 | 12.55 |
| CC96 | 6(2) 2076 | USA | 537663 | 113130832 | 17082755632 | 17.08 | 15.63 |
| CC97 | 6(2) 2084 | USA | 537671 | 104789826 | 15823263726 | 15.82 | 14.48 |
| CC98 | 6(2) 2088 | USA | 537674 | 101151408 | 15273862608 | 15.27 | 13.98 |
| CC99 | 6(2) 2096 | USA | 537681 | 108457320 | 16377055320 | 16.38 | 14.99 |
| CC100 | 6(2) 2097 | USA | 537682 | 112619726 | 17005578626 | 17.01 | 15.56 |
| CC101 | 6(2) 2124 | USA | 537701 | 105735002 | 15965985302 | 15.97 | 14.61 |
| CC102 | 6(2) 2129 | USA | 537706 | 87085380 | 13149892380 | 13.15 | 12.03 |
| CC103 | 6(2) 2131 | USA | 537707 | 89515892 | 13516899692 | 13.52 | 12.37 |
| CC104 | 6(2) 2134 | USA | 537710 | 105391834 | 15914166934 | 15.91 | 14.56 |
| CC105 | 6(2) 2144 | USA | 560168 | 111308072 | 16807518872 | 16.81 | 15.38 |
| CC106 | 6(2) 2145 | USA | 560169 | 104534448 | 15784701648 | 15.78 | 14.45 |
| CC107 | 6(2) 2147 | USA | 560171 | 112361392 | 16966570192 | 16.97 | 15.53 |
| CC108 | 6(2) 2148 | USA | 560172 | 242315032 | 36589569832 | 36.59 | 33.49 |
| CC109 | 6(2) 2151 | USA | 560175 | 118524242 | 17897160542 | 17.90 | 16.38 |
| CC110 | 6(2) 2154 | USA | 560178 | 104310426 | 15750874326 | 15.75 | 14.42 |
| CC111 | 6(2) 2157 | USA | 560181 | 143274170 | 21634399670 | 21.63 | 19.80 |
| CC112 | 6(2) 2159 | USA | 560183 | 108988384 | 16457245984 | 16.46 | 15.06 |
| CC113 | 6(3) 2402 | USA | 537111 | 110420808 | 16673542008 | 16.67 | 15.26 |
| CC114 | 7(1) 2406 | Unknown origin | 209289 | 102498400 | 15477258400 | 15.48 | 14.17 |
| CC115 | 2(7) 1006 | USA | 307030 | 103451878 | 15621233578 | 15.62 | 14.30 |
| CC116 | 3(5) 1540 | USA | 251262 | 135895792 | 20520264592 | 20.52 | 18.78 |
| **Additional lines** | | | | | | | |
| A120 | 6(2) 2061 | Indian subcontinent | 537649 | 111964252 | 16906602052 | 16.91 | 15.47 |
| A121 | 2(7) 797 | USA | 306689 | 122760228 | 18536794428 | 18.54 | 16.97 |
| NC132 | 6(2) 2060 | USA | 537648 | 131040522 | 19787118822 | 19.79 | 18.11 |
| S116 | 4(14) 1806 | Unknown origin | 613459 | 136939574 | 20677875674 | 20.68 | 18.92 |
| S117 | 1(9) | Near East | 262418 | 120139322 | 18141037622 | 18.14 | 16.60 |
| S118 | 3(6) 1576 | USA | 306687 | 122136174 | 18442562274 | 18.44 | 16.88 |
| S119 | 2(7) 598 | Unknown origin | 248859 | 128360962 | 19382505262 | 19.38 | 17.74 |

**Supplementary Table S18:** Filtering criteria for core collection SNPs from resequencing data of core collection.

| **S. No.** | **Filter applied** | **SNPs retained after the filter** |
| --- | --- | --- |
| 0 | Raw variants | 15503654 |
| 1 | Removal of indels | 13177686 |
| 2 | GATK variant filtration | 12216993 |
| 3 | 5>DP>1000 | 11004073 |
| 4 | MAF 0.02, miss 95% | 5001377 |
| 5 | Thin 50 | 1796351 |

**Supplementary Table S19:** Chromosome wise distribution of filtered SNPs from Core Collection resequencing data

| **Chromosome** | **No. of SNPs** |
| --- | --- |
| 1 | 160827 |
| 2 | 177068 |
| 3 | 261522 |
| 4 | 171646 |
| 5 | 132197 |
| 6 | 152620 |
| 7 | 137470 |
| 8 | 153665 |
| 9 | 101854 |
| 10 | 120999 |
| 11 | 124781 |
| 12 | 101702 |
| Total | 1796351 |

**Supplementary Table S20:** Distribution of accession belonging to different gene pool in the different clusters of the ADMIXTURE analysis.

| **S. No** | **Regional gene pool** | **Number** | **ADI** | **ADII** | **ADIII** | **ADIV** | **ADMIXTURE** |
| --- | --- | --- | --- | --- | --- | --- | --- |
| 1 | Australia | 1 | - | - | 1 | - | - |
| 2 | Egypt | 5 | 5 | - | - | - | - |
| 3 | Ethiopia | 2 | 1 | - | 1 | - | - |
| 4 | Europe | 9 | 4 | 1 |  | 1 | 3 |
| 5 | Far east | 15 | 5 | 0 | 1 | 5 | 4 |
| 6 | Indian subcontinent | 32 | 12 | 1 | 15 | - | 4 |
| 7 | Iran-Afghanistan | 9 | 2 | 4 | - | 2 | 1 |
| 8 | Kenya | 1 | - | - | - | 1 |  |
| 9 | Near East | 7 | 3 | - | - | 2 | 2 |
| 10 | Sudan | 1 | - | - | - | - | 1 |
| 11 | Turkey | 6 | 2 | 1 | 1 | 1 | 1 |
| 12 | Unknown origin | 3 | 1 | - | - | 2 | - |
| 13 | USA | 32 | 27 | 0 | 0 | 2 | 3 |
|  | **Total** | **123** | **62** | **7** | **19** | **16** | **19** |

**Supplementary Table S21:** Filtration criteria used for SNPs utilized for GWAS analysis.

| **Filter applied** | **SNPs retained after the filter** |
| --- | --- |
| SNPs from GATK | 17,96,351 |
| Filtration using TASSEL v5 (MAF=0.05, missing data=5%, Max'm and Min'm Heterozygosity=0%) | 320,399 |

**Supplementary Table S22:** Chromosomal distribution of SNPs after filtration (GWAS).

| **Chromosome** | **SNP Count** |
| --- | --- |
| 1 | 30934 |
| 2 | 34218 |
| 3 | 55599 |
| 4 | 23073 |
| 5 | 27710 |
| 6 | 30695 |
| 7 | 27415 |
| 8 | 20587 |
| 9 | 13573 |
| 10 | 18926 |
| 11 | 19612 |
| 12 | 18045 |
| **Total** | 320399 |

**Supplementary Table S23:** QTNs detected for traits OC, SW, PH, PB, HN, DTF and OA-LA

| **Oil Content** | | | | | | | | |
| --- | --- | --- | --- | --- | --- | --- | --- | --- |
| **SNP Name** | **Chromosome** | **Position** | MLMM_Y1 | MLMM_Y2 | FarmCPU_Y1 | FarmCPU_Y2 | BLINK_Y1 | BLINK_Y2 |
| **OC16** | 2 | 28928928 | 2.58E-05 | 1.99E-04 | 5.76E-06 | 4.97E-05 | 5.76E-06 | 4.97E-05 |
| **OC17** | 2 | 52439307 | 1.17E-05 | 4.39E-05 | 2.39E-06 | 8.77E-06 | 2.39E-06 | 8.77E-06 |
| **OC18** | 2 | 54455605 | 2.58E-05 | 1.99E-04 | 5.76E-06 | 4.97E-05 | 5.76E-06 | 4.97E-05 |
| **OC19** | 2 | 63624248 | 1.73E-05 | 1.07E-04 | 3.82E-06 | 2.47E-05 | 3.82E-06 | 2.47E-05 |
| **OC2** | 2 | 65505220 | 6.65E-06 | 9.96E-05 | 1.66E-06 | 2.37E-05 | 1.66E-06 | 2.37E-05 |
| **OC3** | 5 | 4375180 | 7.79E-05 | 5.15E-05 | 1.56E-05 | 8.99E-06 | 1.56E-05 | 8.99E-06 |
| **OC20** | 5 | 4456661 | 2.57E-04 | 4.80E-05 | 7.52E-05 | 8.69E-06 | 7.52E-05 | 8.69E-06 |
| **OC4** | 5 | 5164369 | 9.67E-06 | 2.02E-05 | 1.73E-06 | 3.14E-06 | 1.73E-06 | 3.14E-06 |
| **OC5** | 5 | 5653803 | 7.23E-06 | 4.24E-05 | 1.73E-06 | 7.71E-06 | 1.73E-06 | 7.71E-06 |
| **OC21** | 5 | 6136837 | 2.27E-06 | 1.58E-04 | 2.99E-07 | 3.36E-05 | 2.99E-07 | 3.36E-05 |
| **OC22** | 6 | 79620364 | 2.84E-04 | 9.22E-06 | 9.57E-05 | 1.36E-06 | 9.57E-05 | 1.36E-06 |
| **OC7** | 9 | 18518779 | 1.09E-04 | 4.04E-05 | 1.03E-05 | 1.41E-05 | 1.03E-05 | 1.41E-05 |
| **OC6** | 9 | 18596298 | 4.82E-05 | 7.01E-05 | 1.89E-05 | 1.63E-05 | 1.89E-05 | 1.63E-05 |
| **OC9** | 9 | 79566941 | 2.64E-05 | 1.18E-05 | 6.96E-06 | 1.83E-06 | 6.96E-06 | 1.83E-06 |
| **OC10** | 11 | 74922851 | 1.56E-05 | 1.30E-05 | 6.44E-06 | 2.46E-06 | 6.44E-06 | 2.46E-06 |
| **OC11** | 11 | 74923516 | 1.72E-04 | 4.95E-05 | 8.88E-05 | 1.19E-05 | 8.88E-05 | 1.19E-05 |
| **OC12** | 11 | 75926734 | 2.96E-06 | 3.69E-05 | 8.00E-07 | 7.20E-06 | 8.00E-07 | 7.20E-06 |
| **OC1** | 11 | 76393484 | 2.78E-05 | 2.07E-05 | 8.52E-06 | 3.58E-06 | 8.52E-06 | 3.58E-06 |
| **OC8** | 11 | 76395157 | 6.96E-05 | 7.35E-07 | 1.73E-05 | 7.38E-08 | 1.73E-05 | 7.38E-08 |
| **OC13** | 11 | 76395310 | 3.82E-05 | 1.98E-04 | 1.11E-05 | 4.93E-05 | 1.11E-05 | 4.93E-05 |
| **OC14** | 12 | 5331771 | 2.85E-05 | 1.11E-04 | 1.02E-05 | 2.71E-05 | 1.02E-05 | 2.71E-05 |
| **OC15** | 12 | 5453048 | 2.42E-06 | 6.36E-05 | 8.13E-07 | 1.55E-05 | 8.13E-07 | 1.55E-05 |
| **100 Seed Weight** | | | | | | | | |
| **SNP Name** | **Chromosome** | **Position** | MLMM_Y1 | MLMM_Y2 | FarmCPU_Y1 | FarmCPU_Y2 | BLINK_Y1 | BLINK_Y2 |
| **SW40** | 3 | 20739035 | 7.48E-05 | 2.80E-02 | 1.98E-06 | 4.02E-05 | 8.18E-02 | 4.02E-05 |
| **SW3** | 3 | 72485086 | 7.61E-03 | 3.55E-02 | 1.30E-05 | 1.21E-05 | 7.12E-01 | 1.21E-05 |
| **SW4** | 3 | 72764686 | 7.46E-05 | 1.00E-01 | 3.23E-06 | 2.45E-05 | 6.68E-01 | 2.45E-05 |
| **SW9** | 3 | 73313276 | 1.19E-03 | 9.66E-02 | 1.73E-05 | 7.51E-05 | 4.36E-01 | 7.51E-05 |
| **SW23** | 3 | 74075377 | 3.22E-04 | 5.89E-01 | 1.95E-05 | 9.01E-05 | 3.66E-01 | 1.46E-04 |
| **SW24** | 3 | 74226259 | 8.36E-04 | 1.11E-01 | 4.20E-05 | 7.09E-05 | 7.61E-01 | 7.09E-05 |
| **SW10** | 3 | 74251721 | 6.71E-05 | 5.54E-01 | 8.28E-05 | 8.52E-05 | 1.28E-01 | 8.52E-05 |
| **SW11** | 3 | 82496854 | 3.64E-04 | 2.48E-01 | 5.12E-06 | 8.72E-05 | 6.09E-01 | 8.72E-05 |
| **SW28** | 3 | 82659465 | 5.11E-04 | 2.49E-01 | 1.47E-05 | 9.03E-05 | 5.29E-01 | 9.03E-05 |
| **SW31** | 5 | 9066250 | 5.63E-03 | 4.78E-01 | 7.25E-05 | 9.56E-05 | 9.05E-02 | 1.26E-04 |
| **SW41** | 5 | 24345193 | 1.21E-02 | 8.32E-01 | 1.47E-05 | 5.98E-05 | 4.30E-01 | 5.98E-05 |
| **SW5** | 5 | 25170673 | 5.28E-03 | 1.13E-01 | 1.69E-06 | 4.43E-05 | 2.53E-01 | 4.43E-05 |
| **SW19** | 5 | 38541826 | 9.92E-03 | 5.73E-02 | 3.85E-05 | 7.19E-06 | 6.57E-01 | 7.19E-06 |
| **SW39** | 7 | 23298736 | 4.07E-04 | 4.25E-02 | 4.29E-07 | 8.95E-05 | 5.48E-14 | 8.95E-05 |
| **SW2** | 7 | 77035521 | 2.13E-03 | 2.80E-19 | 2.24E-05 | 1.72E-06 | 1.02E-01 | 1.72E-06 |
| **SW35** | 7 | 81382676 | 2.98E-02 | 9.64E-01 | 3.46E-05 | 6.52E-05 | 8.38E-01 | 6.52E-05 |
| **SW42** | 7 | 81476614 | 6.51E-03 | 2.51E-01 | 7.19E-06 | 8.97E-05 | 6.70E-01 | 8.97E-05 |
| **SW37** | 7 | 81516758 | 1.44E-01 | 6.25E-01 | 6.24E-05 | 6.94E-05 | 2.39E-01 | 6.94E-05 |
| **SW38** | 7 | 81581870 | 1.41E-03 | 5.34E-01 | 3.95E-05 | 6.41E-05 | 2.24E-01 | 6.41E-05 |
| **SW7** | 7 | 81681768 | 1.08E-04 | 7.89E-01 | 4.50E-06 | 6.49E-06 | 1.76E-02 | 6.49E-06 |
| **SW21** | 11 | 50866098 | 5.84E-03 | 2.65E-01 | 9.75E-05 | 6.01E-05 | 3.36E-01 | 6.01E-05 |
| **Plant Height** | | | | | | | | |
| **SNP Name** | **Chromosome** | **Position** | MLMM_Y1 | MLMM_Y2 | FarmCPU_Y1 | FarmCPU_Y2 | BLINK_Y1 | BLINK_Y2 |
| **PH3** | 2 | 10029119 | 2.85E-04 | 5.40E-05 | 2.88E-05 | 3.21E-05 | 2.88E-05 | 3.21E-05 |
| **PH4** | 3 | 15050693 | 5.41E-05 | 6.04E-05 | 9.63E-05 | 3.61E-05 | 1.16E-04 | 3.61E-05 |
| **PH5** | 7 | 6817882 | 2.80E-02 | 9.39E-05 | 1.03E-06 | 5.75E-05 | 1.03E-06 | 5.75E-05 |
| **PH6** | 8 | 17412298 | 6.74E-04 | 1.60E-05 | 5.40E-05 | 8.93E-06 | 5.40E-05 | 8.93E-06 |
| **PH7** | 8 | 20169659 | 1.57E-02 | 1.34E-04 | 1.39E-05 | 8.39E-05 | 1.39E-05 | 8.39E-05 |
| **PH1** | 11 | 66556075 | 6.45E-04 | 1.41E-04 | 7.51E-06 | 8.83E-05 | 7.51E-06 | 8.83E-05 |
| **PH2** | 11 | 79858586 | 2.58E-07 | 6.44E-05 | 1.47E-07 | 3.87E-05 | 1.47E-07 | 3.87E-05 |
| **Number of Primary Branches** | | | | | | | | |
| **SNP Name** | **Chromosome** | **Position** | MLMM_Y1 | MLMM_Y2 | FarmCPU_Y1 | FarmCPU_Y2 | BLINK_Y1 | BLINK_Y2 |
| **PB11** | 2 | 102429506 | 1.67E-05 | 2.89E-04 | 1.68E-05 | 2.13E-05 | 1.68E-05 | 2.13E-05 |
| **PB17** | 4 | 70786298 | 1.65E-04 | 6.68E-02 | 4.89E-05 | 4.26E-05 | 4.89E-05 | 4.26E-05 |
| **PB9** | 5 | 3333942 | 2.05E-04 | 2.78E-04 | 3.13E-05 | 2.46E-05 | 3.13E-04 | 2.46E-05 |
| **PB3** | 5 | 5004100 | 2.38E-06 | 6.73E-09 | 2.34E-06 | 1.06E-06 | 2.34E-06 | 1.06E-06 |
| **PB4** | 5 | 5018372 | 1.02E-05 | 2.07E-02 | 1.76E-05 | 3.58E-05 | 1.76E-05 | 3.58E-05 |
| **PB12** | 5 | 42277991 | 9.02E-05 | 6.45E-01 | 8.01E-05 | 6.79E-05 | 8.01E-05 | 6.79E-05 |
| **PB13** | 5 | 82276627 | 9.56E-06 | 2.67E-02 | 6.93E-06 | 3.05E-06 | 6.93E-06 | 3.05E-06 |
| **PB14** | 6 | 14089337 | 3.51E-05 | 3.27E-02 | 7.80E-05 | 8.72E-05 | 7.80E-05 | 8.72E-05 |
| **PB10** | 6 | 17371115 | 7.39E-05 | 1.16E-09 | 5.71E-05 | 3.83E-05 | 5.71E-05 | 3.83E-05 |
| **PB15** | 6 | 19302718 | 4.81E-05 | 8.35E-02 | 1.71E-05 | 2.31E-06 | 1.71E-05 | 2.31E-06 |
| **PB16** | 6 | 64254157 | 1.17E-05 | 3.80E-02 | 6.23E-06 | 5.60E-05 | 6.23E-06 | 5.60E-05 |
| **PB18** | 10 | 82676657 | 6.81E-05 | 1.87E-01 | 8.38E-05 | 9.22E-05 | 8.38E-05 | 1.82E-04 |
| **PB7** | 11 | 61127419 | 3.63E-06 | 1.03E-01 | 4.09E-06 | 9.26E-05 | 4.09E-06 | 1.23E-04 |
| **PB1** | 12 | 63076322 | 6.71E-05 | 1.13E-03 | 9.35E-05 | 4.19E-05 | 9.35E-05 | 4.19E-05 |
| **Number of Heads** | | | | | | | | |
| **SNP Name** | **Chromosome** | **Position** | MLMM_Y1 | MLMM_Y2 | FarmCPU_Y1 | FarmCPU_Y2 | BLINK_Y1 | BLINK_Y2 |
| **HN2** | 1 | 51435786 | 2.56E-03 | 2.94E-05 | 7.82E-05 | 7.41E-06 | 7.82E-05 | 7.41E-06 |
| **HN4** | 3 | 6291070 | 7.41E-05 | 3.49E-05 | 1.31E-03 | 8.99E-06 | 1.31E-03 | 8.99E-06 |
| **HN5** | 4 | 5068529 | 7.23E-05 | 5.87E-05 | 7.12E-04 | 1.62E-05 | 7.12E-04 | 1.62E-05 |
| **HN6** | 8 | 70637757 | 4.63E-03 | 7.81E-05 | 1.58E-05 | 2.24E-05 | 1.58E-05 | 2.24E-05 |
| **Days to 50% Flowering** | | | | | | | | |
| **SNP Name** | **Chromosome** | **Position** | MLMM_Y1 | MLMM_Y2 | FarmCPU_Y1 | FarmCPU_Y2 | BLINK_Y1 | BLINK_Y2 |
| **DTF7** | 3 | 54974331 | 1.94E-05 | 1.69E-03 | 2.20E-05 | 2.90E-05 | 2.20E-05 | 2.90E-05 |
| **DTF16** | 3 | 71505376 | 3.64E-05 | 1.64E-04 | 4.04E-05 | 6.24E-05 | 4.04E-05 | 6.24E-05 |
| **DTF14** | 7 | 23722756 | 1.90E-05 | 5.39E-03 | 1.10E-05 | 9.18E-05 | 1.10E-05 | 9.18E-05 |
| **DTF18** | 7 | 60728673 | 1.48E-04 | 4.44E-04 | 8.62E-05 | 2.68E-05 | 8.62E-05 | 2.68E-05 |
| **DTF10** | 7 | 86666550 | 8.17E-06 | 1.38E-05 | 1.95E-05 | 4.87E-05 | 1.95E-05 | 4.87E-05 |
| **DTF17** | 9 | 10130804 | 4.30E-05 | 2.70E-04 | 5.20E-05 | 9.57E-05 | 5.20E-05 | 9.57E-05 |
| **DTF11** | 9 | 10172594 | 4.78E-05 | 6.06E-05 | 4.92E-05 | 2.14E-05 | 4.92E-05 | 2.14E-05 |
| **DTF1** | 10 | 5542556 | 1.19E-05 | 3.02E-05 | 1.31E-05 | 4.33E-05 | 1.31E-05 | 4.33E-05 |
| **DTF15** | 10 | 25735101 | 5.94E-05 | 7.04E-04 | 3.59E-05 | 2.73E-05 | 3.59E-05 | 2.73E-05 |
| **DTF2** | 11 | 7169035 | 1.11E-04 | 2.49E-04 | 2.26E-04 | 5.47E-05 | 1.56E-05 | 5.47E-05 |
| **DTF3** | 12 | 67791959 | 1.25E-04 | 9.45E-04 | 9.28E-05 | 4.19E-05 | 9.28E-05 | 4.19E-05 |
| **DTF4** | 12 | 73502054 | 1.59E-05 | 2.10E-07 | 5.40E-06 | 5.17E-07 | 5.40E-06 | 5.17E-07 |
| **Oleic and Linoliec acid** | | | | | | | | |
| **SNP Name** | **Chromosome** | **Position** | MLMM_OA | MLMM_LA | FarmCPU_OA | FarmCPU_LA | BLINK_OA | BLINK_LA |
| **OA-LA41** | 1 | 6622051 | 6.28E-06 | 7.91E-06 | 5.12E-05 | 2.61E-04 | 2.78E-05 | 1.09E-05 |
| **OA-LA1** | 1 | 87484658 | 7.10E-16 | 4.10E-16 | 8.16E-07 | 2.88E-07 | 8.95E-05 | 2.55E-08 |
| **OA-LA42** | 3 | 95192517 | 1.95E-07 | 1.83E-07 | 5.88E-01 | 1.61E-01 | 4.05E-04 | 1.40E-05 |
| **OA-LA47** | 4 | 91337544 | 2.64E-03 | 3.73E-03 | 7.32E-03 | 2.13E-02 | 6.71E-09 | 1.62E-05 |
| **OA-LA45** | 5 | 18141864 | 6.56E-05 | 4.10E-05 | 4.72E-01 | 1.34E-01 | 1.76E-02 | 2.10E-03 |
| **OA-LA20** | 5 | 18141916 | 3.38E-06 | 2.27E-06 | 2.87E-01 | 1.25E-01 | 2.02E-02 | 3.66E-03 |
| **OA-LA15** | 5 | 18156971 | 1.71E-05 | 8.44E-06 | 1.99E-01 | 7.10E-01 | 1.46E-02 | 2.15E-03 |
| **OA-LA17** | 5 | 18157188 | 2.37E-05 | 1.28E-05 | 5.06E-03 | 2.06E-01 | 3.15E-02 | 2.79E-03 |
| **OA-LA44** | 5 | 18172726 | 9.77E-05 | 7.00E-05 | 4.18E-01 | 4.96E-01 | 9.42E-02 | 1.57E-02 |
| **OA-LA36** | 5 | 18179456 | 2.19E-05 | 1.36E-05 | 3.68E-01 | 8.54E-03 | 3.68E-02 | 9.48E-04 |
| **OA-LA43** | 5 | 20097188 | 9.60E-05 | 6.11E-05 | 6.36E-08 | 4.66E-08 | 1.63E-11 | 5.29E-11 |
| **OA-LA48** | 6 | 200251 | 1.88E-04 | 2.12E-04 | 1.73E-02 | 7.72E-03 | 7.97E-13 | 1.48E-11 |
| **OA-LA35** | 6 | 84242096 | 6.90E-05 | 5.38E-05 | 1.88E-01 | 3.20E-02 | 4.00E-02 | 3.24E-03 |
| **OA-LA6** | 7 | 66993411 | 1.93E-06 | 2.17E-06 | 3.99E-01 | 3.27E-01 | 9.93E-01 | 5.14E-01 |
| **OA-LA46** | 10 | 6049142 | 1.22E-03 | 1.69E-03 | 1.48E-02 | 5.43E-02 | 4.99E-05 | 8.59E-05 |
| **OA-LA2** | 12 | 13995580 | 5.64E-08 | 7.68E-08 | 7.68E-05 | 6.17E-06 | 2.86E-03 | 3.49E-04 |

**Supplementary Table S24:** LD-block analysis indicating the chromosomal positions and size of LD blocks around the QTN regions.

| **Chromosome** | **Start** | **Stop** | **Size (bp)** | **QTNs in block** |
| --- | --- | --- | --- | --- |
| **1** | 51434278 | 51437588 | 3310 | HN2 |
| **2** | 28922612 | 28933586 | 10974 | OC16 |
| **2** | 65504522 | 65505295 | 773 | OC2 |
| **3** | 15050021 | 15050758 | 737 | PH4 |
| **3** | 20738872 | 20740163 | 1291 | SW40 |
| **3** | 71505376 | 71506444 | 1068 | DTF16 |
| **3** | 72485086 | 72491942 | 6856 | SW3 |
| **3** | 72757736 | 72765262 | 7526 | SW4 |
| **3** | 73313276 | 73318787 | 5511 | SW9 |
| **3** | 74075377 | 74075578 | 201 | SW23 |
| **3** | 74226092 | 74226470 | 378 | SW24 |
| **3** | 74248951 | 74255191 | 6240 | SW10 |
| **3** | 82490504 | 82498275 | 7771 | SW11 |
| **3** | 82652706 | 82660036 | 7330 | SW28 |
| **4** | 5067299 | 5068529 | 1230 | HN5 |
| **4** | 70781902 | 70787659 | 5757 | PB17 |
| **5** | 3330817 | 3334647 | 3830 | PB9 |
| **5** | 4452839 | 4456661 | 3822 | OC20 |
| **5** | 5001779 | 5004100 | 2321 | PB3 |
| **5** | 5018372 | 5019193 | 821 | PB4 |
| **5** | 5158195 | 5168536 | 10341 | OC4 |
| **5** | 5653803 | 5654266 | 463 | OC5 |
| **5** | 6136837 | 6138595 | 1758 | OC21 |
| **5** | 9066127 | 9066250 | 123 | SW31 |
| **5** | 18134952 | 18148648 | 13696 | OA-LA20,OA-LA45 |
| **5** | 18150161 | 18162355 | 12194 | OA-LA15,OA-LA17 |
| **5** | 18173768 | 18182752 | 8984 | OA-LA36,OA-LA44 |
| **5** | 20090503 | 20102092 | 11589 | OA-LA43 |
| **5** | 82276318 | 82276627 | 309 | PB13 |
| **6** | 14089115 | 14089337 | 222 | PB14 |
| **6** | 17371029 | 17373561 | 2532 | PB10 |
| **6** | 19302664 | 19302718 | 54 | PB15 |
| **6** | 64253789 | 64254157 | 368 | PB16 |
| **7** | 6817882 | 6817938 | 56 | PH5 |
| **7** | 23721628 | 23723311 | 1683 | DTF14 |
| **7** | 60728506 | 60728819 | 313 | DTF18 |
| **7** | 66992541 | 66993411 | 870 | OA-LA6 |
| **7** | 77035444 | 77035521 | 77 | SW2 |
| **7** | 81379207 | 81382676 | 3469 | SW35 |
| **7** | 81471897 | 81476976 | 5079 | SW42 |
| **7** | 81516758 | 81517827 | 1069 | SW37 |
| **7** | 81581870 | 81583725 | 1855 | SW38 |
| **7** | 81675000 | 81688655 | 13655 | SW7 |
| **8** | 17412298 | 17412639 | 341 | PH6 |
| **8** | 70636629 | 70637900 | 1271 | HN6 |
| **9** | 10130459 | 10130804 | 345 | DTF17 |
| **9** | 10172257 | 10172594 | 337 | DTF11 |
| **9** | 18512732 | 18519143 | 6411 | OC7 |
| **10** | 5542348 | 5542793 | 445 | DTF1 |
| **10** | 6045112 | 6049491 | 4379 | OA-LA46 |
| **10** | 25735101 | 25735598 | 497 | DTF15 |
| **10** | 82675855 | 82677204 | 1349 | PB18 |
| **11** | 7168739 | 7176012 | 7273 | DTF2 |
| **11** | 50861872 | 50866098 | 4226 | SW21 |
| **11** | 66553639 | 66556075 | 2436 | PH1 |
| **11** | 74922797 | 74923516 | 719 | OC10,OC11 |
| **11** | 76392069 | 76399253 | 7184 | OC1,OC13,OC8 |
| **11** | 79858586 | 79859722 | 1136 | PH2 |
| **12** | 5453048 | 5455742 | 2694 | OC15 |
| **12** | 63075676 | 63076322 | 646 | PB1 |
| **12** | 73501990 | 73502634 | 644 | DTF4 |

**Supplementary Table S25:** KASP primers designed for multiple traits across different sites.

| Trait | Gene | Site | Allele | FAM-Forward 1 | HEX-forward 2 | ROX-reverse |
| --- | --- | --- | --- | --- | --- | --- |
| **Oil Content** | | | | | | |
| OC1/8 | Protein BIG GRAIN 1-like E | **CtA_chr11_76395157** | C/**T** | acagaaaatgaaatgaccgaatcat | acagaaaatgaaatgaccgaatcat | gtcccacaaagtcattgattagtc |
|  |  | **CtA_chr11_76393484** | G/**A** | cgggcccatcagcaatca | cgggcccatcagcaatca | gaccaggatcggttcagg |
|  |  | CtA_chr11_76395211 | **A**/T | gaccgtattttcaagataagttagcttta | accgtattttcaagataagttagctttt | agttgattatggattgcaaacgag |
|  |  | CtA_chr11_76395615 | G/**A** | agttgattatggattgcaaacgag | gcaagtgataaagatagggatttgaaag | ggcaaaagtgttcttggattctt |
|  |  | CtA_chr11_76395777 | **G**/A | gctttgttccacttctattggaa | gctttgttccacttctattggaa | gctaggacatattgatgttataatggag |
|  |  | CtA_chr11_76397432 | **G**/A | tcttgcaagtctcaagaaaaacaa | tcttgcaagtctcaagaaaaacag | cacgtacatatagggttgggtt |
| OC12 | Myosin-binding protein 2 | **CtA_chr11_75926734** | C/T | tttaggttgctaagaatcagattctttt | tttaggttgctaagaatcagattctttt | tttaggttgctaagaatcagattctttt |
| **Oleic and Linoleic acid** | | | | | | |
| OA_LA20 |  | CtA_chr5_18148648 | G**/A** | gcatggagggcatggtgt | gcatggagggcatggtgc | gttgccattaataggaaacttgca |
|  |  | CtA_chr5_18150161 | C**/T** | actttttaaaagagaaatgatttattttaaactttttattacata | actttttaaaagagaaatgatttattttaaactttttattacata | tgaagtaactccttatcagtgttgatta |
| OA_LA17 | Cytochrome P450 71A4-like | **CtA_chr5_18157188** | G**/A** | ttttgaacgcccaattaatctactt | tttgaacgcccaattaatctactc | tttgaacgcccaattaatctactc |
| **Days to 50% flowering** | | | | | | |
| DTF2 | 40S ribosomal protein S8 | **CtA_chr11_7169035** | T**/C** | tcaagcttgtaatggagttcct | caagcttgtaatggagttccc | ccttggatcaagttctagatagtcaatt |
| DTF2 |  | CtA_chr11_7171031 | C/T | gcccatttttcattaagcccattt | gcccatttttcattaagcccattt | gcccatttttcattaagcccattt |
| **Seed weight** | | | | | | |
| SW23 | Peptidyl-prolyl cis-trans isomerase CYP57 isoform X1 | **CtA_chr3_74075377** | **T**/C | gataccctgaccatgggtc | ggagaacacttctctatttcacac | acttagttctcccttaactgtcc |
|  |  | CtA_chr3_74068671 | C/**T** | tctcttacttgatcgcacactt | tctcttacttgatcgcacactt | tctcttacttgatcgcacactt |
|  |  | CtA_chr3_74068722 | **A**/G | ccaccattttgtcgatcaaaca | ccaccattttgtcgatcaaacg | ccctgtctgtaggttaacagtt |
| SW10 | RNA-binding protein 2-like isoform X2 | **CtA_chr3_74244899** | **C**/T | gcatagaatttggtgattttttcaaaggt | agaatttggtgattttttcaaaggc | agggacaaagtagaacttgagaatt |
| SW39 | Probable transcription repressor OFP9 | **CtA_chr7_23298736** | **T**/C | acacaaaatcgatgaggtatccaa | cacaaaatcgatgaggtatccag | gataccctgaccatgggtc |
| Plant height | | | | | | |
| PH4 | Trafficking protein particle complex subunit 6B (TRAPPC6B) | **CtA_chr3_15050693** | **G**/A | tgctatttagaaacaaacaaatcacaaaaaa | tgctatttagaaacaaacaaatcacaaaaaa | tgctatttagaaacaaacaaatcacaaaaaa |
| **Primary branches** | | | | | | |
| PB9 | F-box/LRR-repeat protein 14-like isoform X1 | CtA_chr5_3334647 | **C**/T | tgaaattgaggacagtgtgttatttt | tgaaattgaggacagtgtgttatttt | ctacacgaagtttgtgccattaa |
| PB18 | Probable protein phosphatase 2C 65 | **CtA_chr7_82676657** | A/G | aatttacttggagttgtcaaacatga | ttacttggagttgtcaaacatgg | gttgtgcgattatggctatactg |
| PB14 | Transcription factor TCP20 isoform X1 | **CtA_chr6_14089337** | C**/G** | aggtcttcctttatttcctaatttaatttactac | aggtcttcctttatttcctaatttaatttactac | aggtcttcctttatttcctaatttaatttactac |

**Bold sites refer to QTN sites, Bold allele refers to superior alleles**

**Supplementary Table S26:** Validated SNP markers through KASP associated with different traits and their corresponding genotype panels.

| Trait | Site | Accessions |
| --- | --- | --- |
| Oil Content | **CtA_chr11_76395157** | **CC093(40.45)*,CC101(46.66)*,CC108(45.35)*,CC004(26.905),CC006(26.015),CC007(26.215),CC009(21.25),CC010(23.45)** |
|  | **CtA_chr11_76393484** | **CC093(40.45)*,CC108(45.35)*,CC004(26.905),CC006(26.015),CC007(26.215),CC009(21.25),CC010(23.45)** |
|  | CtA_chr11_76395211 | CC093(40.45)*,CC101(46.66)*,CC105,CC108(45.35)*,CC009(21.25),CC006(26.015),CC010(23.45),CC004(26.015),CC007(26.215) |
|  | CtA_chr11_76395615 | CC101(46.66)*,CC108(45.35)*,CC004(26.905),CC006(26.015),CC007(26.215),CC009(21.25),CC010(23.45) |
|  | CtA_chr11_76395777 | CC101(46.66)*,CC108(45.35)*,CC004(26.905),CC006(26.015),CC007(26.215),CC009(21.25),CC010(23.45) |
|  | CtA_chr11_76397432 | CC093(40.45)*,CC101(46.66)*,CC004(26.905),CC010(23.45),CC105,CC004(26.905),CC007(26.215) |
|  | **CtA_chr11_75926734*** | **CC093(40.45)*,CC101(46.66)*,CC105,CC108(45.35)*,CC009(21.25),CC006(26.015),CC010(23.45), CC004(26.905), CC007(26.215)** |
| OA_LA20 | CtA_chr5_18148648 | CC063 (77.36)*,CC082 (78.32)*,CC087 (78.8)*,CC014 (45.67),CC108 (14.67) |
|  | CtA_chr5_18150161 | CC082(78.32)*,CC014(45.67),CC108(14.67) |
|  | CtA_chr5_18157188 | CC063(77.36)*,CC078(87.26)*,CC082(78.32)*,CC087(78.8)*,CC014(45.67),CC108(14.67) |
| Days to 50% flower | **CtA_chr11_7169035** | **CC047(165)*,CC072(171)*,CC099(167.5)*,CC080(167.5)*,CC056(143),CC085(138),CC034(137.5),CC078(135),CC004(130),CC082(128.5)** |
|  | CtA_chr11_7171031 | CC072(171)*,CC080(167.5)*,CC056(143),CC085(138),CC094(134),CC004(130),CC082(128.5) |
| Seed weight | CtA_chr3_74068671 | CC028(5.92)*,CC109(3.56),CC112(3.82),CC001(2.41) |
|  | CtA_chr3_74068722 | CC028(5.92)*,CC109(3.56)*,CC112(3.82)*,CC001(2.41) |
|  | **CtA_chr3_74244899** | **CC045(5.48)*,CC029(5.62)*,CC037(7.7)*,CC043(8.36)*,CC006(3.03),CC009(2.02),CC002(3.515)** |
|  | **CtA_chr3_74075377** | **CC028(5.92)*,CC030(5.13)*,CC036(5.73)*,CC001(2.41),CC109(3.56),CC112(3.82),CC071(2.86)** |
|  | **CtA_chr7_23298736** | **CC037(7.7)*,CC029(5.62)*,CC043(8.36)*,CC009(2.02),CC006(3.03),CC002(3.515)** |
| Plant height | **CtA_chr3_15050693** | **CC096(210)*,CC067(191.5)*,CC079(181)*,CC082(179)*,CC097(103.5),CC002(89.5),CC023(99),CC017(107)** |
| Primary branch | CtA_chr5_3334647 | CC003(7.5)*,CC066(32.5),CC063(7),CC021(6.5),CC046(29),CC059(11) |
|  | CtA_chr7_82676657 | CC003 |
|  | CtA_chr6_14089337 | CC046(29)*,CC048(29.5)*,CC093(31)*,CC094(28)*,CC003(7.5),CC018(7),CC021(6.5) |

***** Accessions with high trait value**, Bold sites** refer to QTN

**Supplementary Table S27:** Phenotypic values of post-harvest traits (OC, OA-LA and SW) for three contrasting accessions CC106, CC113 and S116.

| **Trait** | **CC106** | **CC113** | **S116** |
| --- | --- | --- | --- |
| **OC (%)** | 46.66 | 34.59 | 18.20 |
| **OA-LA (%)** | 79.38/14.63 | 12.56/77.38 | 9.68/80.32 |
| **SW (g)** | 2.26 | 3.6 | 4.2 |

**Supplementary Table S28:** Candidate genes selected for qRT PCR analysis. Primer sequences, Tm and corresponding product size have also been indicated.

| **Candidate Genes** |  |  |  |  |  |  |  |
| --- | --- | --- | --- | --- | --- | --- | --- |
| **Trait** | **Candidate gene and its annotation** | **Wu et al. 2021 gene model** | **FP (Sequence)** | **FP Tm** | **RP (Sequence)** | **RP Tm** | **Product size (bp)** |
| **100 seed weight** | g57921; protein FRIGIDA-ESSENTIAL 1-like isoform | **CtAH01G0212700** | TGCACCGGAGATTGACGAAG | 60.39 | ACTGGCATTCGTGGGTTCTC | 60.32 | 124 |
| **Oil Content** | g64666; probable UDP-N-acetylglucosamine--peptide N-acetylglucosaminyltransferase SPINDLY isoform X1 | **CtAH12G0121500** | CGGTTCACGGAGCAGTAGTT | 60.04 | GCCAAATTCTCGTCGTCCCT | 60.39 | 129 |
| **Oil Content** | g16872; myosin-binding protein 2 | **CtAH07G0039400** | ACGAAACAACTTCGGTTCTTCA | 58.74 | ACGGGAGGCTTTTCATCAGA | 59.02 | 172 |
| **Oleic and Linoleic Acid** | g43426; cytochrome P450 71A4-like | **CtAH09G0125900** | TAACGTGGGTGGATCGGTTG | 60.04 | TCTTGGCCTTCATCGTGGAC | 60.04 | 130 |
|  | | | | | | | |
|  | **Annotation** | **FP (Sequence)** | **FP Tm** | **RP (Sequence)** | **RP Tm** | **Product size (bp)** |  |
| **Reference Gene** | g31039; eukaryotic initiation factor 4A | GACTGGCTCACCGACAAGA | 59.33 | GATGACCAGCGAGACCTGC | 60.52 | 180 |  |

**Supplementary Table S29:** qRT-PCR analysis of candidate genes during seed development. Relative expression (fold change) of three candidate genes measured by qRT-PCR in three genotypes (CC113, CC106, S116) at four seed developmental stages (5, 10, 20 and 30 days after pollination, DAP). Fold-change values are shown as means across three biological replicates with accompanying standard deviation (Std. Dev.) and standard error (Std. Err.). Fold change was calculated using the 2^-ΔΔC^_T_ method and values are expressed relative to the calibrator sample (CC106_5DAP).

| **Candidate Gene** | **Stage** | **CC113** | | | **CC106** | | | **S116** | | |
| --- | --- | --- | --- | --- | --- | --- | --- | --- | --- | --- |
|  |  | **Fold Change** | **Std. Dev.** | **Std. Err.** | **Fold Change** | **Std. Dev.** | **Std. Err.** | **Fold Change** | **Std. Dev.** | **Std. Err.** |
| **g16872: myosin-binding protein 2 (OC)** | 5DAP | 0.172 | 0.073 | 0.042 | 1.005 | 0.098 | 0.057 | 0.232 | 0.087 | 0.05 |
|  | 10DAP | 0.87 | 0.365 | 0.211 | 6.397 | 0.547 | 0.316 | 0.107 | 0.007 | 0.005 |
|  | 20DAP | 0.093 | 0.042 | 0.024 | 0.134 | 0.023 | 0.013 | 0.124 | 0.051 | 0.029 |
|  | 30DAP | 0.02 | 0.01 | 0.005 | 0.005 | 0.002 | 0.001 | 0.059 | 0.039 | 0.023 |
|  | | | | | | | | | | |
| **g43426: cytochrome P450 71A4-like (OA-LA)** | 5DAP | 0.153 | 0.014 | 0.008 | 1.032 | 0.256 | 0.148 | 0.173 | 0.052 | 0.03 |
|  | 10DAP | 21.76 | 1.443 | 0.833 | 1.814 | 0.707 | 0.408 | 13.197 | 0.692 | 0.489 |
|  | 20DAP | 0.253 | 0.034 | 0.019 | 0.054 | 0.017 | 0.01 | 0.071 | 0.015 | 0.01 |
|  | 30DAP | 1.079 | 0.509 | 0.294 | 0.311 | 0.148 | 0.085 | 0.598 | 0.014 | 0.01 |
|  | | | | | | | | | | |
| **g57921: protein FRIGIDA-ESSENTIAL 1-like isoform (SW)** | 5DAP | 0.828 | 0.235 | 0.136 | 1.043 | 0.306 | 0.177 | 0.632 | 0.339 | 0.195 |
|  | 10DAP | 1.084 | 0.311 | 0.18 | 0.061 | 0.024 | 0.017 | 0.563 | 0.049 | 0.035 |
|  | 20DAP | 4.022 | 1.032 | 0.596 | 2.802 | 0.384 | 0.222 | 8.992 | 1.636 | 0.945 |
|  | 30DAP | 0.728 | 0.178 | 0.126 | 0.251 | 0.024 | 0.017 | 0.353 | 0.176 | 0.125 |
|  | | | | | | | | | | |
| **g64666: SPINDLY isoform X1 (OC)** | 5DAP | 0.653 | 0.192 | 0.111 | 1.047 | 0.315 | 0.182 | 0.178 | 0.052 | 0.030 |
|  | 10DAP | 0.932 | 0.366 | 0.211 | 0.677 | 0.160 | 0.092 | 0.640 | 0.212 | 0.122 |
|  | 20DAP | 0.307 | 0.101 | 0.058 | 0.174 | 0.049 | 0.028 | 0.526 | 0.152 | 0.107 |
|  | 30DAP | 0.385 | 0.014 | 0.010 | 0.673 | 0.371 | 0.214 | 0.098 | 0.042 | 0.029 |

**Supplementary Table S30: Accessions with high number of variable genes that can be useful in regional breeding programmes**

| Accessions | Regional gene pool | Core | Soft core | Shell | Cloud | Private |
| --- | --- | --- | --- | --- | --- | --- |
| CC38 | Indian subcontinent | 19583 | 27579 | 14667 | 1110 | 204 |
| CC108 | USA | 19620 | 27982 | 14734 | 1044 | 11 |
| CC72 | Europe | 19555 | 27521 | 14788 | 981 | 1 |
| NC132 | USA | 19659 | 28355 | 15047 | 813 | 0 |
| CC51 | Iran-Afghanistan | 19598 | 27505 | 14851 | 798 | 0 |
| CC91 | USA | 19629 | 27812 | 14469 | 782 | 0 |
| CC69 | Europe | 19116 | 26391 | 14054 | 698 | 0 |
| CC10 | Far east | 19503 | 27192 | 14101 | 647 | 0 |
| CC62 | Europe | 19583 | 27677 | 15632 | 606 | 0 |
| CC44 | Indian subcontinent | 19490 | 27680 | 14762 | 532 | 0 |
| CC53 | Iran-Afghanistan | 19575 | 27471 | 14951 | 492 | 0 |
| CC89 | USA | 19643 | 28079 | 15189 | 486 | 0 |
| Anhui1 | Far east | 19565 | 28066 | 15815 | 8 | 0 |
| CC25 | Indian subcontinent | 19663 | 27997 | 15765 | 0 | 0 |
| CC14 | Far east | 19660 | 28147 | 15679 | 3 | 0 |
| CC60 | Near East | 19668 | 27982 | 15570 | 0 | 0 |
| CC42 | Indian subcontinent | 19718 | 27951 | 15503 | 1 | 0 |
| CC56 | Iran-Afghanistan | 19714 | 27883 | 15485 | 1 | 0 |
| CC115 | Europe | 19379 | 27106 | 15433 | 0 | 0 |
| CC48 | Near East | 19546 | 27500 | 15432 | 0 | 0 |
| CC57 | Iran-Afghanistan | 19636 | 27772 | 15431 | 1 | 0 |
| CC13 | Far east | 19723 | 28023 | 15415 | 0 | 0 |
| CC20 | Indian subcontinent | 19727 | 27843 | 15383 | 0 | 0 |
| CC19 | Indian subcontinent | 19542 | 27536 | 15379 | 391 | 0 |
| CC31 | Indian subcontinent | 19583 | 27557 | 15377 | 0 | 0 |

**Supplementary Table S31: Distribution of the stress related genes across different regional pools**

| Gene name | Function | Australia (1) | Egypt (5) | Ethopia (2) | Europe (9) | Far East (16) | Indian subcontinent (31) | Iran Afghanistan (9) | Kenya (1) | Near East (7) | Sudan (1) | Turkey (7) | Unknown (4) | USA (32) |
| --- | --- | --- | --- | --- | --- | --- | --- | --- | --- | --- | --- | --- | --- | --- |
| pang21384.t1 | putative disease resistance protein At4g11170 isoform X2 [Cynara cardunculus var | 0 | 0 | 0 | 0 | 0 | 0 | 6 | 0 | 0 | 0 | 0 | 0 | 0 |
| pang25176.t1 | putative late blight resistance protein homolog R1A-10 [Cynara cardunculus var | 0 | 0 | 0 | 0 | 0 | 0 | 7 | 0 | 0 | 0 | 0 | 0 | 0 |
| pang4435.t2 | TMV resistance protein N-like [Cynara cardunculus var | 0 | 0 | 0 | 0 | 0 | 0 | 7 | 0 | 0 | 0 | 0 | 0 | 0 |
| pang21366.t1 | TMV resistance protein N-like [Cynara cardunculus var | 0 | 0 | 0 | 0 | 0 | 0 | 8 | 0 | 0 | 0 | 0 | 0 | 0 |
| pang24904.t1 | TMV resistance protein N-like isoform X[Cynara cardunculus var | 0 | 0 | 0 | 0 | 0 | 0 | 8 | 0 | 0 | 0 | 0 | 0 | 0 |
| pang27672.t1 | disease resistance protein RML1A-like [Cynara cardunculus var | 0 | 0 | 0 | 0 | 0 | 0 | 8 | 0 | 0 | 0 | 0 | 0 | 0 |
| pang28566.t1 | probable disease resistance protein At1g61180 [Cynara cardunculus var | 0 | 0 | 0 | 0 | 0 | 0 | 8 | 0 | 0 | 0 | 0 | 0 | 0 |
| pang12563.t1 | TMV resistance protein N-like [Cynara cardunculus var | 0 | 0 | 0 | 0 | 0 | 0 | 9 | 0 | 0 | 0 | 0 | 0 | 0 |
| pang12675.t1 | TMV resistance protein N-like isoform X2 [Cynara cardunculus var | 0 | 0 | 0 | 0 | 0 | 0 | 9 | 0 | 0 | 0 | 0 | 0 | 0 |
| pang24300.t1 | receptor-like protein 33 [Cynara cardunculus var | 0 | 0 | 0 | 0 | 0 | 0 | 9 | 0 | 0 | 0 | 0 | 0 | 0 |
| pang29092.t1 | putative late blight resistance protein homolog R1B-14 [Cynara cardunculus var | 0 | 0 | 0 | 0 | 0 | 0 | 9 | 0 | 0 | 0 | 0 | 0 | 0 |
| pang36791.t1 | replication protein A 70 kDa DNA-binding subunit A-like [Cynara cardunculus var | 0 | 2 | 0 | 6 | 3 | 5 | 3 | 0 | 1 | 0 | 1 | 1 | 9 |
| pang37101.t1 | protein TIFY 8-like [Cynara cardunculus var | 0 | 1 | 0 | 6 | 2 | 5 | 3 | 0 | 1 | 0 | 0 | 1 | 11 |
| pang37044.t1 | replication protein A 70 kDa DNA-binding subunit A-like [Cynara cardunculus var | 0 | 2 | 0 | 5 | 2 | 7 | 3 | 0 | 1 | 0 | 0 | 1 | 10 |
| pang36759.t1 | TMV resistance protein N-like [Cynara cardunculus var | 0 | 1 | 0 | 6 | 5 | 7 | 3 | 0 | 1 | 0 | 1 | 1 | 10 |
| pang37029.t1 | receptor-like protein kinase 2 [Cynara cardunculus var | 0 | 1 | 0 | 5 | 4 | 6 | 7 | 0 | 1 | 0 | 0 | 1 | 9 |
| pang36454.t1 | disease resistance protein RPP13-like [Cynara cardunculus var | 0 | 2 | 0 | 5 | 5 | 10 | 6 | 0 | 2 | 0 | 1 | 1 | 8 |
| pang36058.t2 | replication protein A 70 kDa DNA-binding subunit A-like [Cynara cardunculus var | 0 | 3 | 0 | 4 | 3 | 15 | 3 | 0 | 4 | 0 | 1 | 1 | 10 |
| pang35887.t2 | probable LRR receptor-like serine/threonine-protein kinase At5g10290 [Cynara cardunculus var | 1 | 4 | 0 | 6 | 8 | 18 | 5 | 0 | 5 | 0 | 2 | 1 | 15 |
| pang35479.t1 | receptor-like protein kinase HAIKU2 [Cynara cardunculus var | 1 | 4 | 0 | 6 | 6 | 18 | 7 | 0 | 6 | 0 | 3 | 1 | 17 |
| pang35489.t1 | uncharacterized protein LOC | 0 | 4 | 0 | 6 | 5 | 18 | 6 | 0 | 5 | 0 | 3 | 1 | 20 |
| pang2148.t1 | disease resistance protein RML1B-like [Cynara cardunculus var | 1 | 3 | 2 | 3 | 11 | 23 | 0 | 1 | 6 | 1 | 4 | 2 | 20 |
| pang2137.t1 | putative inactive disease susceptibility protein LOV[Cynara cardunculus var | 1 | 4 | 2 | 5 | 12 | 22 | 0 | 1 | 6 | 1 | 4 | 3 | 21 |
| pang1316.t1 | root allergen protein-like [Cynara cardunculus var | 1 | 3 | 2 | 4 | 11 | 22 | 0 | 1 | 6 | 1 | 6 | 3 | 20 |
| pang24698.t1 | TMV resistance protein N-like [Cynara cardunculus var | 1 | 5 | 2 | 7 | 8 | 23 | 0 | 1 | 5 | 1 | 6 | 4 | 20 |
| pang26560.t1 | WD repeat-containing protein 76 [Cynara cardunculus var | 1 | 4 | 0 | 7 | 10 | 21 | 5 | 1 | 5 | 0 | 5 | 1 | 23 |
| pang23374.t1 | protein TIFY 8-like [Cynara cardunculus var | 1 | 3 | 2 | 9 | 13 | 18 | 8 | 1 | 3 | 1 | 5 | 3 | 23 |
| pang25537.t1 | putative disease resistance RPP13-like protein [Cynara cardunculus var | 0 | 2 | 1 | 7 | 11 | 24 | 0 | 1 | 5 | 1 | 4 | 4 | 23 |
| pang28098.t1 | E3 ubiquitin-protein ligase PRT6 isoform X6 [Cynara cardunculus var | 1 | 2 | 2 | 8 | 10 | 23 | 0 | 0 | 5 | 1 | 6 | 4 | 23 |
| pang437.t1 | receptor-like protein kinase HAIKU2 [Cynara cardunculus var | 1 | 3 | 2 | 3 | 10 | 22 | 6 | 1 | 7 | 1 | 5 | 4 | 16 |
| pang988.t1 | DNA polymerase zeta catalytic subunit isoform X3 [Cynara cardunculus var | 1 | 4 | 1 | 5 | 12 | 26 | 5 | 1 | 6 | 1 | 4 | 4 | 16 |
| pang1887.t1 | histone deacetylase HDT | 1 | 3 | 2 | 5 | 10 | 20 | 6 | 0 | 6 | 1 | 6 | 4 | 20 |
| pang27430.t1 | WD40 repeat-containing protein HOS | 1 | 4 | 1 | 5 | 11 | 21 | 8 | 0 | 4 | 0 | 5 | 4 | 21 |
| pang13320.t1 | TMV resistance protein N-like [Cynara cardunculus var | 1 | 4 | 2 | 5 | 12 | 24 | 4 | 1 | 6 | 1 | 5 | 4 | 19 |
| pang25155.t1 | TMV resistance protein N-like isoform X4 [Cynara cardunculus var | 0 | 4 | 2 | 8 | 13 | 22 | 6 | 1 | 4 | 0 | 4 | 1 | 26 |
| pang2582.t1 | TMV resistance protein N-like [Cynara cardunculus var | 1 | 4 | 1 | 4 | 13 | 22 | 7 | 1 | 6 | 1 | 5 | 3 | 19 |
| pang30834.t1 | disease resistance-like protein DSC[Cynara cardunculus var | 1 | 4 | 2 | 7 | 15 | 22 | 0 | 1 | 6 | 0 | 5 | 2 | 28 |
| pang436.t1 | receptor-like protein kinase HAIKU2 [Cynara cardunculus var | 1 | 3 | 2 | 3 | 9 | 21 | 7 | 1 | 5 | 1 | 5 | 3 | 21 |
| pang5044.t1 | TMV resistance protein N-like [Cynara cardunculus var | 1 | 4 | 1 | 7 | 12 | 25 | 7 | 1 | 6 | 1 | 3 | 2 | 19 |
| pang20832.t1 | probable LRR receptor-like serine/threonine-protein kinase At2g24230 [Cynara cardunculus var | 1 | 5 | 2 | 8 | 14 | 24 | 7 | 1 | 6 | 0 | 4 | 4 | 19 |
| pang24975.t2 | TMV resistance protein N-like [Cynara cardunculus var | 1 | 2 | 2 | 5 | 11 | 25 | 0 | 1 | 5 | 1 | 4 | 3 | 26 |
| pang7954.t1 | TMV resistance protein N-like [Cynara cardunculus var | 0 | 5 | 2 | 8 | 12 | 27 | 5 | 1 | 5 | 1 | 4 | 1 | 21 |
| pang3683.t1 | TMV resistance protein N-like [Cynara cardunculus var | 1 | 2 | 2 | 5 | 12 | 27 | 3 | 0 | 5 | 1 | 6 | 4 | 20 |
| pang750.t1 | TMV resistance protein N-like [Cynara cardunculus var | 1 | 3 | 2 | 4 | 10 | 23 | 9 | 1 | 7 | 0 | 6 | 3 | 17 |
| pang19950.t1 | E3 ubiquitin-protein ligase CHIP [Cynara cardunculus var | 1 | 4 | 1 | 7 | 12 | 21 | 6 | 1 | 6 | 1 | 3 | 4 | 25 |
| pang677.t1 | peroxidase | 1 | 1 | 2 | 5 | 11 | 25 | 6 | 1 | 7 | 1 | 6 | 3 | 18 |
| pang1382.t2 | TMV resistance protein N-like [Cynara cardunculus var | 1 | 4 | 2 | 4 | 12 | 24 | 9 | 1 | 4 | 1 | 5 | 3 | 20 |
| pang18465.t1 | E3 ubiquitin-protein ligase PRT6 isoform X6 [Cynara cardunculus var | 1 | 5 | 2 | 7 | 13 | 26 | 6 | 1 | 4 | 1 | 4 | 3 | 22 |
| pang3228.t1 | disease resistance protein RML1A-like [Cynara cardunculus var | 1 | 5 | 2 | 4 | 12 | 24 | 7 | 1 | 6 | 0 | 4 | 3 | 22 |
| pang16724.t1 | E3 ubiquitin-protein ligase RFWD3-like [Cynara cardunculus var | 1 | 3 | 1 | 9 | 8 | 24 | 8 | 1 | 5 | 1 | 4 | 2 | 23 |
| pang15798.t1 | glutathione peroxidase | 0 | 5 | 1 | 7 | 11 | 20 | 9 | 1 | 5 | 1 | 4 | 3 | 25 |
| pang2575.t1 | replication protein A 70 kDa DNA-binding subunit A-like [Cynara cardunculus var | 1 | 3 | 2 | 6 | 12 | 24 | 6 | 1 | 6 | 1 | 6 | 3 | 21 |
| pang1127.t2 | TMV resistance protein N-like [Cynara cardunculus var | 1 | 3 | 2 | 3 | 11 | 25 | 8 | 1 | 6 | 1 | 6 | 3 | 18 |
| pang23334.t1 | putative disease resistance RPP13-like protein [Cynara cardunculus var | 0 | 3 | 1 | 6 | 11 | 25 | 6 | 0 | 4 | 1 | 6 | 4 | 22 |
| pang24752.t1 | TMV resistance protein N-like [Cynara cardunculus var | 0 | 2 | 2 | 7 | 10 | 21 | 6 | 1 | 5 | 0 | 5 | 2 | 28 |
| pang35472.t1 | TMV resistance protein N-like [Cynara cardunculus var | 1 | 5 | 0 | 7 | 9 | 25 | 8 | 1 | 5 | 0 | 3 | 3 | 23 |
| pang4664.t1 | protein SRC2 homolog [Cynara cardunculus var | 1 | 5 | 2 | 4 | 11 | 27 | 0 | 0 | 7 | 1 | 6 | 3 | 24 |
| pang13817.t1 | autophagy-related protein | 1 | 2 | 2 | 9 | 11 | 23 | 9 | 1 | 6 | 0 | 6 | 3 | 21 |
| pang14968.t1 | replication protein A 70 kDa DNA-binding subunit A-like [Cynara cardunculus var | 1 | 3 | 1 | 7 | 14 | 23 | 8 | 1 | 5 | 1 | 3 | 3 | 25 |
| pang24586.t1 | serine/threonine-protein phosphatase PP-X isozyme 2-like isoform X2 [Cynara cardunculus var | 1 | 4 | 1 | 9 | 7 | 21 | 8 | 1 | 6 | 0 | 5 | 4 | 24 |
| pang5273.t1 | WD repeat and HMG-box DNA-binding protein | 1 | 4 | 2 | 7 | 13 | 25 | 6 | 1 | 5 | 0 | 5 | 3 | 24 |
| pang13895.t1 | putative disease resistance protein At3g14460 [Cynara cardunculus var | 1 | 3 | 2 | 7 | 12 | 25 | 5 | 1 | 6 | 0 | 5 | 2 | 25 |
| pang25742.t1 | BTB/POZ domain and ankyrin repeat-containing protein NPR[Cynara cardunculus var | 0 | 4 | 2 | 6 | 11 | 23 | 9 | 1 | 5 | 1 | 5 | 3 | 22 |
| pang27333.t1 | probable LRR receptor-like serine/threonine-protein kinase At1g34110 [Cynara cardunculus var | 1 | 1 | 2 | 6 | 13 | 23 | 6 | 0 | 4 | 1 | 5 | 3 | 27 |
| pang3708.t1 | putative late blight resistance protein homolog R1B-14 [Cynara cardunculus var | 1 | 5 | 2 | 4 | 13 | 24 | 3 | 1 | 6 | 1 | 6 | 3 | 25 |
| pang9233.t1 | probable disease resistance protein At1g61180 [Cynara cardunculus var | 1 | 5 | 1 | 8 | 14 | 24 | 7 | 0 | 6 | 1 | 6 | 3 | 22 |
| pang24029.t1 | beta-amylase | 1 | 5 | 2 | 8 | 7 | 24 | 8 | 0 | 6 | 1 | 4 | 3 | 24 |
| pang23688.t1 | DNA mismatch repair protein MLH | 1 | 3 | 2 | 8 | 11 | 25 | 8 | 1 | 7 | 1 | 6 | 3 | 19 |
| pang20660.t1 | replication protein A 70 kDa DNA-binding subunit A-like [Cynara cardunculus var | 1 | 4 | 1 | 6 | 12 | 26 | 7 | 0 | 6 | 1 | 5 | 4 | 21 |
| pang32189.t1 | replication protein A 70 kDa DNA-binding subunit A-like [Cynara cardunculus var | 1 | 3 | 1 | 7 | 11 | 24 | 9 | 1 | 4 | 1 | 4 | 4 | 23 |
| pang3109.t1 | replication protein A 70 kDa DNA-binding subunit A-like [Cynara cardunculus var | 1 | 5 | 1 | 5 | 10 | 26 | 6 | 1 | 5 | 1 | 6 | 4 | 21 |
| pang3722.t1 | uncharacterized protein LOC | 1 | 4 | 1 | 6 | 10 | 26 | 6 | 1 | 5 | 1 | 5 | 4 | 22 |
| pang12578.t1 | TMV resistance protein N-like isoform X6 [Cynara cardunculus var | 0 | 5 | 1 | 5 | 12 | 28 | 0 | 1 | 6 | 0 | 5 | 3 | 27 |
| pang13399.t1 | TMV resistance protein N-like [Cynara cardunculus var | 1 | 5 | 1 | 7 | 13 | 29 | 4 | 0 | 6 | 1 | 3 | 2 | 25 |
| pang14008.t1 | LRR receptor-like serine/threonine-protein kinase ERL[Cynara cardunculus var | 1 | 5 | 2 | 6 | 14 | 24 | 6 | 1 | 6 | 1 | 6 | 4 | 22 |
| pang15918.t1 | kirola-like [Cynara cardunculus var | 1 | 4 | 2 | 4 | 12 | 24 | 7 | 1 | 5 | 1 | 5 | 2 | 25 |
| pang18891.t1 | cytochrome P450 CYP82D47-like [Cynara cardunculus var | 0 | 2 | 1 | 6 | 13 | 23 | 8 | 1 | 5 | 1 | 4 | 1 | 27 |
| pang24760.t1 | TMV resistance protein N-like [Cynara cardunculus var | 1 | 4 | 2 | 8 | 14 | 23 | 9 | 0 | 6 | 0 | 5 | 2 | 25 |
| pang24852.t1 | TMV resistance protein N-like [Cynara cardunculus var | 1 | 2 | 2 | 6 | 12 | 24 | 7 | 1 | 6 | 1 | 3 | 2 | 26 |
| pang28330.t1 | TMV resistance protein N-like [Cynara cardunculus var | 1 | 5 | 1 | 7 | 13 | 24 | 7 | 1 | 7 | 1 | 4 | 3 | 23 |
| pang3868.t1 | TMV resistance protein N-like [Cynara cardunculus var | 1 | 4 | 2 | 4 | 8 | 23 | 6 | 1 | 7 | 1 | 6 | 4 | 22 |
| pang3961.t2 | TMV resistance protein N-like [Cynara cardunculus var | 1 | 5 | 2 | 5 | 15 | 25 | 7 | 1 | 7 | 0 | 5 | 3 | 22 |
| pang4074.t1 | TMV resistance protein N-like [Cynara cardunculus var | 0 | 4 | 2 | 6 | 12 | 20 | 8 | 1 | 7 | 1 | 5 | 3 | 25 |
| pang8105.t1 | putative late blight resistance protein homolog R1A-10 [Cynara cardunculus var | 1 | 4 | 1 | 7 | 14 | 25 | 8 | 1 | 6 | 1 | 6 | 2 | 21 |
| pang22128.t1 | alpha-ketoglutarate-dependent dioxygenase alkB [Cynara cardunculus var | 0 | 5 | 2 | 6 | 12 | 25 | 8 | 1 | 4 | 0 | 4 | 3 | 26 |
| pang26142.t1 | peroxidase 4-like [Cynara cardunculus var | 1 | 5 | 2 | 8 | 10 | 24 | 6 | 1 | 6 | 1 | 4 | 4 | 25 |
| pang13355.t1 | replication protein A 70 kDa DNA-binding subunit A-like [Cynara cardunculus var | 0 | 4 | 1 | 8 | 9 | 23 | 8 | 1 | 6 | 0 | 6 | 4 | 23 |
| pang20755.t1 | replication protein A 70 kDa DNA-binding subunit A-like [Cynara cardunculus var | 1 | 4 | 2 | 9 | 10 | 24 | 8 | 1 | 5 | 0 | 5 | 3 | 25 |
| pang34754.t1 | uncharacterized protein LOC | 1 | 4 | 2 | 9 | 12 | 27 | 6 | 1 | 7 | 0 | 3 | 2 | 25 |
| pang14130.t1 | WD repeat and HMG-box DNA-binding protein | 1 | 4 | 2 | 7 | 8 | 24 | 8 | 1 | 6 | 1 | 5 | 1 | 25 |
| pang15961.t1 | TMV resistance protein N-like [Cynara cardunculus var | 1 | 3 | 1 | 7 | 13 | 27 | 6 | 1 | 6 | 1 | 5 | 2 | 23 |
| pang18699.t1 | TMV resistance protein N-like [Cynara cardunculus var | 1 | 4 | 2 | 6 | 14 | 24 | 8 | 1 | 5 | 0 | 6 | 3 | 24 |
| pang19234.t1 | phospholipase A I isoform X2 [Cynara cardunculus var | 1 | 5 | 2 | 8 | 8 | 25 | 7 | 1 | 5 | 0 | 5 | 2 | 26 |
| pang21674.t1 | TMV resistance protein N-like [Cynara cardunculus var | 1 | 2 | 1 | 6 | 13 | 23 | 8 | 0 | 7 | 1 | 4 | 4 | 24 |
| pang22976.t1 | putative disease resistance protein At4g11170 [Cynara cardunculus var | 0 | 5 | 0 | 7 | 13 | 24 | 6 | 1 | 7 | 1 | 5 | 1 | 26 |
| pang24146.t1 | TMV resistance protein N-like [Cynara cardunculus var | 1 | 3 | 1 | 8 | 11 | 23 | 6 | 1 | 7 | 1 | 5 | 3 | 25 |
| pang25579.t1 | disease resistance protein RML1A-like [Cynara cardunculus var | 1 | 4 | 1 | 6 | 15 | 25 | 8 | 0 | 6 | 1 | 3 | 2 | 26 |
| pang3761.t1 | TMV resistance protein N-like [Cynara cardunculus var | 1 | 5 | 1 | 4 | 11 | 24 | 7 | 1 | 7 | 1 | 6 | 3 | 22 |
| pang4435.t1 | TMV resistance protein N-like isoform X2 [Cynara cardunculus var | 1 | 4 | 2 | 5 | 14 | 27 | 5 | 1 | 7 | 1 | 5 | 3 | 22 |
| pang5931.t1 | receptor-like protein Cf-9 homolog [Cynara cardunculus var | 1 | 5 | 2 | 6 | 12 | 24 | 7 | 1 | 7 | 0 | 4 | 3 | 25 |
| pang7992.t1 | TMV resistance protein N-like [Cynara cardunculus var | 0 | 5 | 2 | 6 | 12 | 25 | 9 | 1 | 7 | 1 | 5 | 3 | 20 |
| pang18037.t1 | AAA-ATPase At3g50940-like [Cynara cardunculus var | 1 | 5 | 2 | 8 | 13 | 28 | 9 | 1 | 5 | 1 | 5 | 2 | 21 |
| pang19211.t1 | DNA repair protein UVH3 isoform X5 [Cynara cardunculus var | 1 | 3 | 2 | 8 | 10 | 25 | 8 | 1 | 6 | 1 | 4 | 3 | 24 |
| pang34876.t1 | FACT complex subunit SPT | 1 | 3 | 1 | 8 | 11 | 27 | 7 | 0 | 5 | 0 | 5 | 3 | 25 |
| pang27512.t1 | protein ROS | 1 | 4 | 1 | 7 | 11 | 26 | 9 | 1 | 6 | 1 | 6 | 2 | 21 |
| pang12283.t1 | protein timeless homolog isoform X2 [Cynara cardunculus var | 1 | 4 | 2 | 8 | 12 | 24 | 6 | 1 | 5 | 1 | 5 | 4 | 26 |
| pang26899.t1 | putative Peroxidase 48 [Cynara cardunculus var | 1 | 4 | 2 | 5 | 12 | 20 | 6 | 1 | 6 | 1 | 6 | 4 | 28 |
| pang16256.t1 | putative poly [ADP-ribose] polymerase 3 [Cynara cardunculus var | 0 | 3 | 1 | 9 | 12 | 25 | 8 | 1 | 6 | 1 | 6 | 3 | 22 |
| pang24130.t1 | replication protein A 70 kDa DNA-binding subunit A-like [Cynara cardunculus var | 0 | 3 | 2 | 7 | 12 | 23 | 7 | 1 | 7 | 1 | 5 | 3 | 25 |
| pang19552.t1 | superoxide dismutase [Fe], chloroplastic [Cynara cardunculus var | 1 | 3 | 1 | 8 | 10 | 26 | 8 | 1 | 5 | 1 | 4 | 2 | 25 |
| pang11464.t1 | regulatory protein NPR3-like [Cynara cardunculus var | 1 | 4 | 2 | 8 | 12 | 23 | 6 | 0 | 6 | 1 | 6 | 4 | 26 |
| pang15107.t1 | plant intracellular Ras-group-related LRR protein 3 [Cynara cardunculus var | 1 | 5 | 2 | 6 | 13 | 24 | 7 | 0 | 4 | 1 | 5 | 4 | 27 |
| pang18971.t1 | protein argonaute 4-like [Cynara cardunculus var | 1 | 5 | 2 | 8 | 14 | 24 | 8 | 0 | 5 | 1 | 6 | 4 | 24 |
| pang20114.t2 | disease resistance protein RML1A-like [Cynara cardunculus var | 0 | 4 | 2 | 8 | 14 | 23 | 7 | 1 | 6 | 1 | 4 | 2 | 28 |
| pang21329.t1 | TMV resistance protein N-like [Cynara cardunculus var | 1 | 3 | 2 | 7 | 14 | 26 | 9 | 1 | 7 | 1 | 5 | 2 | 21 |
| pang21899.t1 | LRR receptor-like serine/threonine-protein kinase [Cynara cardunculus var | 1 | 5 | 2 | 6 | 14 | 24 | 8 | 1 | 7 | 1 | 6 | 2 | 23 |
| pang2372.t1 | kirola-like [Cynara cardunculus var | 1 | 3 | 2 | 3 | 13 | 25 | 7 | 1 | 6 | 1 | 5 | 3 | 24 |
| pang23918.t1 | protein argonaute 1-like [Cynara cardunculus var | 0 | 3 | 2 | 6 | 11 | 26 | 5 | 1 | 5 | 0 | 5 | 4 | 26 |
| pang28329.t1 | TMV resistance protein N-like isoform X[Cynara cardunculus var | 1 | 4 | 1 | 6 | 13 | 25 | 8 | 1 | 6 | 1 | 5 | 3 | 23 |
| pang28820.t1 | TMV resistance protein N-like [Cynara cardunculus var | 1 | 3 | 1 | 8 | 12 | 28 | 0 | 1 | 5 | 1 | 5 | 3 | 29 |
| pang16460.t1 | AAA-ATPase At5g | 1 | 4 | 2 | 7 | 8 | 25 | 7 | 1 | 5 | 1 | 5 | 3 | 26 |
| pang25055.t1 | chaperone protein ClpB3, chloroplastic [Cynara cardunculus var | 1 | 4 | 2 | 6 | 13 | 26 | 9 | 0 | 7 | 1 | 4 | 4 | 22 |
| pang14393.t1 | probable inactive shikimate kinase like 2, chloroplastic [Cynara cardunculus var | 1 | 3 | 2 | 8 | 11 | 25 | 8 | 0 | 6 | 1 | 5 | 2 | 26 |
| pang19657.t1 | replication protein A 70 kDa DNA-binding subunit A-like [Cynara cardunculus var | 1 | 4 | 2 | 8 | 12 | 27 | 6 | 0 | 6 | 0 | 6 | 2 | 26 |
| pang16284.t1 | replication protein A 70 kDa DNA-binding subunit A-like [Cynara cardunculus var | 1 | 4 | 2 | 7 | 11 | 27 | 7 | 1 | 5 | 1 | 4 | 2 | 26 |
| pang10960.t1 | uncharacterized protein LOC | 1 | 3 | 1 | 8 | 12 | 23 | 8 | 1 | 6 | 1 | 5 | 4 | 25 |
| pang26827.t1 | vacuolar protein sorting-associated protein 4 | 1 | 3 | 2 | 5 | 12 | 25 | 9 | 1 | 7 | 1 | 3 | 3 | 24 |
| pang14501.t1 | putative disease resistance protein At4g11170 isoform X[Cynara cardunculus var | 1 | 4 | 2 | 8 | 11 | 25 | 7 | 1 | 4 | 1 | 6 | 3 | 26 |
| pang14782.t1 | LRR receptor-like serine/threonine-protein kinase RCH[Cynara cardunculus var | 1 | 4 | 0 | 7 | 13 | 26 | 7 | 1 | 7 | 1 | 6 | 4 | 21 |
| pang15951.t1 | putative disease resistance protein RGA4 [Cynara cardunculus var | 1 | 4 | 1 | 4 | 14 | 24 | 6 | 1 | 7 | 1 | 5 | 4 | 25 |
| pang21222.t1 | TMV resistance protein N-like [Cynara cardunculus var | 1 | 4 | 2 | 7 | 13 | 24 | 9 | 1 | 5 | 1 | 4 | 4 | 25 |
| pang21862.t1 | TMV resistance protein N-like isoform X6 [Cynara cardunculus var | 1 | 5 | 1 | 6 | 14 | 26 | 8 | 1 | 7 | 1 | 5 | 1 | 24 |
| pang27506.t1 | actin cytoskeleton-regulatory complex protein pan[Cynara cardunculus var | 1 | 2 | 2 | 6 | 10 | 24 | 9 | 1 | 7 | 1 | 4 | 3 | 24 |
| pang6005.t1 | probable disease resistance protein At5g66900 [Cynara cardunculus var | 1 | 4 | 0 | 6 | 11 | 26 | 8 | 1 | 6 | 1 | 3 | 4 | 24 |
| pang8035.t1 | TMV resistance protein N-like [Cynara cardunculus var | 1 | 3 | 2 | 5 | 12 | 26 | 9 | 1 | 6 | 1 | 4 | 2 | 24 |
| pang25144.t1 | AAA-ATPase At5g57480 [Cynara cardunculus var | 1 | 5 | 1 | 7 | 10 | 24 | 7 | 1 | 6 | 1 | 5 | 4 | 26 |
| pang7289.t1 | protein TIFY 9 [Cynara cardunculus var | 0 | 5 | 2 | 7 | 12 | 26 | 7 | 1 | 5 | 0 | 6 | 3 | 26 |
| pang19934.t1 | replication protein A 70 kDa DNA-binding subunit A-like [Cynara cardunculus var | 0 | 4 | 1 | 8 | 10 | 26 | 8 | 1 | 7 | 1 | 5 | 3 | 23 |
| pang14288.t1 | replication protein A 70 kDa DNA-binding subunit A-like [Cynara cardunculus var | 1 | 3 | 2 | 8 | 13 | 28 | 7 | 1 | 3 | 1 | 4 | 3 | 27 |
| pang8920.t1 | serine/threonine-protein kinase/endoribonuclease IRE | 1 | 4 | 1 | 8 | 14 | 23 | 8 | 1 | 6 | 1 | 5 | 3 | 27 |
| pang12685.t1 | universal stress protein PHOS32 [Cynara cardunculus var | 1 | 4 | 2 | 6 | 13 | 25 | 7 | 1 | 7 | 0 | 4 | 2 | 28 |
| pang14915.t1 | TMV resistance protein N-like [Cynara cardunculus var | 1 | 4 | 2 | 6 | 10 | 26 | 8 | 1 | 5 | 1 | 5 | 3 | 25 |
| pang17214.t1 | TMV resistance protein N-like [Cynara cardunculus var | 1 | 3 | 2 | 2 | 15 | 22 | 6 | 1 | 7 | 1 | 6 | 4 | 27 |
| pang17945.t1 | cell division control protein 48 homolog D [Cynara cardunculus var | 1 | 5 | 2 | 8 | 11 | 25 | 6 | 1 | 5 | 1 | 6 | 4 | 27 |
| pang22012.t1 | DELLA protein GAI-like [Cynara cardunculus var | 1 | 2 | 2 | 6 | 9 | 28 | 8 | 1 | 4 | 0 | 5 | 3 | 26 |
| pang6254.t1 | DNA polymerase zeta catalytic subunit isoform X5 [Cynara cardunculus var | 1 | 5 | 2 | 7 | 10 | 24 | 9 | 1 | 5 | 1 | 5 | 3 | 27 |
| pang28774.t1 | peroxidase 3-like [Cynara cardunculus var | 1 | 4 | 1 | 9 | 12 | 23 | 7 | 1 | 5 | 1 | 6 | 3 | 29 |
| pang16871.t1 | probable E3 ubiquitin ligase SUD | 1 | 5 | 2 | 7 | 12 | 27 | 9 | 1 | 4 | 1 | 4 | 3 | 26 |
| pang11409.t1 | replication protein A 70 kDa DNA-binding subunit A-like [Cynara cardunculus var | 1 | 3 | 2 | 8 | 14 | 24 | 8 | 1 | 7 | 0 | 5 | 3 | 27 |
| pang8173.t1 | replication protein A 70 kDa DNA-binding subunit A-like [Cynara cardunculus var | 1 | 5 | 2 | 9 | 10 | 26 | 8 | 1 | 3 | 1 | 6 | 4 | 26 |
| pang13257.t1 | replication protein A 70 kDa DNA-binding subunit A-like [Cynara cardunculus var | 1 | 2 | 2 | 9 | 10 | 29 | 9 | 1 | 6 | 1 | 5 | 2 | 22 |
| pang11670.t1 | TMV resistance protein N-like [Cynara cardunculus var | 0 | 3 | 2 | 6 | 13 | 28 | 5 | 1 | 7 | 1 | 4 | 3 | 26 |
| pang19113.t1 | protein argonaute 1-like [Cynara cardunculus var | 0 | 3 | 2 | 7 | 14 | 27 | 8 | 1 | 5 | 1 | 5 | 2 | 26 |
| pang21198.t1 | putative late blight resistance protein homolog R1B-14 [Cynara cardunculus var | 1 | 4 | 1 | 5 | 12 | 24 | 7 | 1 | 6 | 1 | 6 | 1 | 29 |
| pang21577.t1 | putative disease resistance RPP13-like protein [Cynara cardunculus var | 1 | 4 | 1 | 8 | 14 | 25 | 6 | 1 | 6 | 0 | 6 | 3 | 28 |
| pang21952.t1 | TMV resistance protein N-like [Cynara cardunculus var | 0 | 4 | 0 | 7 | 12 | 26 | 8 | 1 | 5 | 1 | 5 | 3 | 26 |
| pang27659.t1 | protein SRC2 homolog [Cynara cardunculus var | 0 | 4 | 1 | 2 | 14 | 23 | 9 | 1 | 6 | 1 | 4 | 4 | 27 |
| pang32849.t1 | TMV resistance protein N-like [Cynara cardunculus var | 0 | 5 | 1 | 7 | 13 | 22 | 9 | 1 | 7 | 1 | 5 | 2 | 28 |
| pang9165.t1 | TMV resistance protein N-like [Cynara cardunculus var | 1 | 5 | 2 | 8 | 14 | 23 | 8 | 1 | 6 | 1 | 5 | 3 | 28 |
| pang10579.t1 | peroxidase 9-like [Cynara cardunculus var | 1 | 2 | 2 | 8 | 11 | 27 | 9 | 1 | 3 | 1 | 5 | 4 | 26 |
| pang16579.t1 | probable plastid-lipid-associated protein | 1 | 2 | 2 | 8 | 12 | 28 | 9 | 1 | 4 | 1 | 5 | 3 | 25 |
| pang3814.t1 | protein MICRORCHIDIA 7-like [Cynara cardunculus var | 1 | 4 | 1 | 4 | 12 | 28 | 7 | 1 | 7 | 1 | 3 | 4 | 25 |
| pang30017.t1 | replication protein A 70 kDa DNA-binding subunit A-like [Cynara cardunculus var | 1 | 5 | 2 | 7 | 14 | 28 | 7 | 1 | 6 | 1 | 5 | 4 | 24 |
| pang29272.t1 | replication protein A 70 kDa DNA-binding subunit A-like [Cynara cardunculus var | 1 | 4 | 2 | 8 | 11 | 27 | 8 | 1 | 7 | 1 | 5 | 4 | 23 |
| pang5926.t1 | replication protein A 70 kDa DNA-binding subunit A-like [Cynara cardunculus var | 1 | 4 | 2 | 6 | 11 | 25 | 8 | 1 | 7 | 1 | 6 | 3 | 25 |
| pang23367.t1 | replication protein A 70 kDa DNA-binding subunit B [Cynara cardunculus var | 0 | 2 | 1 | 8 | 10 | 27 | 8 | 0 | 7 | 1 | 5 | 4 | 24 |
| pang29371.t1 | uncharacterized protein LOC | 0 | 5 | 2 | 8 | 14 | 25 | 9 | 1 | 5 | 1 | 5 | 4 | 26 |
| pang11075.t1 | TMV resistance protein N-like [Cynara cardunculus var | 1 | 5 | 2 | 6 | 15 | 26 | 8 | 1 | 6 | 1 | 6 | 2 | 26 |
| pang11156.t1 | disease resistance-like protein DSC[Cynara cardunculus var | 0 | 5 | 1 | 6 | 14 | 26 | 7 | 1 | 4 | 1 | 6 | 3 | 28 |
| pang17590.t2 | putative disease resistance protein At4g11170 [Cynara cardunculus var | 1 | 3 | 2 | 6 | 13 | 27 | 8 | 1 | 5 | 0 | 5 | 4 | 26 |
| pang3767.t1 | TMV resistance protein N-like [Cynara cardunculus var | 1 | 4 | 2 | 5 | 14 | 28 | 8 | 1 | 5 | 1 | 6 | 4 | 23 |
| pang8468.t1 | senescence-associated carboxylesterase 101-like [Cynara cardunculus var | 0 | 4 | 2 | 5 | 14 | 26 | 8 | 1 | 5 | 1 | 5 | 4 | 26 |
| pang9203.t1 | disease resistance-like protein DSC[Cynara cardunculus var | 1 | 4 | 1 | 8 | 12 | 27 | 8 | 1 | 7 | 0 | 5 | 4 | 24 |
| pang9271.t1 | TMV resistance protein N-like [Cynara cardunculus var | 1 | 4 | 2 | 7 | 14 | 29 | 7 | 1 | 7 | 1 | 5 | 3 | 23 |
| pang28493.t1 | DNA polymerase delta subunit 3 [Cynara cardunculus var | 1 | 4 | 2 | 6 | 13 | 26 | 8 | 1 | 6 | 1 | 6 | 4 | 25 |
| pang18183.t2 | DNA replication licensing factor MCM2 [Cynara cardunculus var | 1 | 4 | 2 | 7 | 10 | 27 | 7 | 1 | 6 | 1 | 6 | 3 | 26 |
| pang15582.t1 | peroxidase | 1 | 4 | 2 | 7 | 13 | 27 | 9 | 1 | 5 | 0 | 5 | 3 | 27 |
| pang9414.t1 | poly [ADP-ribose] polymerase 2 [Cynara cardunculus var | 0 | 4 | 1 | 8 | 11 | 28 | 6 | 1 | 5 | 1 | 4 | 3 | 29 |
| pang9934.t1 | probable inactive shikimate kinase like 2, chloroplastic [Cynara cardunculus var | 0 | 4 | 2 | 7 | 13 | 25 | 9 | 1 | 6 | 1 | 6 | 2 | 27 |
| pang10154.t1 | replication protein A 70 kDa DNA-binding subunit A-like [Cynara cardunculus var | 1 | 3 | 2 | 7 | 12 | 26 | 8 | 0 | 7 | 1 | 6 | 4 | 25 |
| pang15358.t1 | replication protein A 70 kDa DNA-binding subunit A-like [Cynara cardunculus var | 1 | 4 | 2 | 5 | 11 | 26 | 9 | 1 | 6 | 1 | 5 | 4 | 25 |
| pang11337.t1 | disease resistance protein RML1A-like [Cynara cardunculus var | 0 | 4 | 2 | 8 | 13 | 28 | 6 | 1 | 6 | 1 | 5 | 3 | 27 |
| pang17296.t1 | disease resistance protein RML1A-like [Cynara cardunculus var | 1 | 5 | 2 | 6 | 14 | 25 | 8 | 1 | 4 | 1 | 5 | 4 | 29 |
| pang32090.t1 | disease resistance protein RML1A-like [Cynara cardunculus var | 1 | 3 | 2 | 8 | 11 | 26 | 7 | 1 | 7 | 1 | 6 | 3 | 26 |
| pang33030.t1 | TMV resistance protein N-like [Cynara cardunculus var | 1 | 5 | 2 | 7 | 15 | 29 | 7 | 1 | 6 | 1 | 6 | 3 | 24 |
| pang35069.t1 | thaumatin-like protein [Cynara cardunculus var | 1 | 5 | 2 | 7 | 15 | 26 | 9 | 0 | 6 | 1 | 4 | 4 | 27 |
| pang5201.t1 | disease resistance-like protein DSC[Cynara cardunculus var | 1 | 5 | 2 | 7 | 12 | 26 | 9 | 1 | 6 | 1 | 6 | 3 | 25 |
| pang11421.t1 | DNA repair protein RAD4 isoform X4 [Cynara cardunculus var | 1 | 2 | 1 | 9 | 12 | 29 | 8 | 1 | 7 | 0 | 4 | 2 | 27 |
| pang11944.t1 | helicase-like transcription factor CHR28 isoform X4 [Cynara cardunculus var | 0 | 4 | 1 | 9 | 14 | 26 | 7 | 1 | 6 | 1 | 6 | 3 | 28 |
| pang9731.t1 | peroxidase | 1 | 3 | 1 | 9 | 11 | 28 | 9 | 1 | 6 | 1 | 4 | 4 | 25 |
| pang28152.t1 | probable aquaporin PIP | 1 | 5 | 2 | 5 | 11 | 24 | 8 | 1 | 6 | 1 | 5 | 4 | 29 |
| pang10051.t1 | protein ACTIVITY OF BC | 1 | 5 | 1 | 8 | 12 | 27 | 8 | 1 | 7 | 1 | 4 | 4 | 26 |
| pang17175.t1 | regulatory-associated protein of TOR | 1 | 4 | 2 | 9 | 12 | 26 | 8 | 1 | 6 | 1 | 5 | 4 | 27 |
| pang32861.t1 | replication protein A 70 kDa DNA-binding subunit A-like [Cynara cardunculus var | 1 | 5 | 2 | 9 | 12 | 27 | 6 | 1 | 6 | 0 | 6 | 4 | 28 |
| pang14365.t1 | replication protein A 70 kDa DNA-binding subunit A-like [Cynara cardunculus var | 1 | 5 | 2 | 7 | 13 | 29 | 8 | 1 | 6 | 1 | 5 | 3 | 25 |
| pang33317.t1 | replication protein A 70 kDa DNA-binding subunit A-like [Cynara cardunculus var | 1 | 3 | 2 | 8 | 13 | 25 | 8 | 1 | 7 | 1 | 6 | 3 | 27 |
| pang10592.t1 | probable inactive receptor kinase At5g10020 [Cynara cardunculus var | 1 | 5 | 2 | 7 | 13 | 25 | 8 | 1 | 7 | 0 | 6 | 4 | 27 |
| pang11716.t1 | probable LRR receptor-like serine/threonine-protein kinase At4g36180 [Cynara cardunculus var | 1 | 5 | 2 | 7 | 13 | 29 | 7 | 1 | 7 | 1 | 3 | 4 | 26 |
| pang15188.t1 | disease resistance-like protein DSC[Cynara cardunculus var | 1 | 5 | 0 | 7 | 16 | 26 | 7 | 1 | 6 | 1 | 6 | 4 | 27 |
| pang16028.t1 | E3 ubiquitin-protein ligase KEG isoform X2 [Cynara cardunculus var | 1 | 4 | 2 | 7 | 13 | 27 | 8 | 1 | 7 | 1 | 6 | 3 | 25 |
| pang33824.t1 | TMV resistance protein N-like isoform X5 [Cynara cardunculus var | 1 | 5 | 2 | 8 | 13 | 28 | 8 | 1 | 5 | 1 | 5 | 4 | 26 |
| pang33865.t1 | probable LRR receptor-like serine/threonine-protein kinase IRK [Cynara cardunculus var | 1 | 2 | 2 | 6 | 16 | 26 | 8 | 1 | 6 | 1 | 6 | 1 | 29 |
| pang34055.t1 | disease resistance protein RML1A-like [Cynara cardunculus var | 0 | 4 | 2 | 6 | 16 | 26 | 9 | 1 | 7 | 1 | 5 | 2 | 27 |
| pang8383.t1 | plant intracellular Ras-group-related LRR protein 4-like [Cynara cardunculus var | 1 | 2 | 1 | 6 | 13 | 28 | 8 | 1 | 5 | 1 | 5 | 3 | 27 |
| pang12771.t1 | replication protein A 70 kDa DNA-binding subunit A-like [Cynara cardunculus var | 1 | 3 | 1 | 8 | 10 | 27 | 7 | 1 | 7 | 1 | 6 | 4 | 26 |
| pang18831.t1 | vacuolar protein sorting-associated protein 4 | 1 | 3 | 2 | 9 | 10 | 29 | 9 | 1 | 7 | 1 | 5 | 4 | 23 |
| pang15408.t2 | probable disease resistance protein At1g61180 [Cynara cardunculus var | 1 | 4 | 2 | 7 | 14 | 27 | 9 | 1 | 6 | 1 | 5 | 3 | 27 |
| pang8251.t1 | TMV resistance protein N-like [Cynara cardunculus var | 1 | 3 | 2 | 8 | 15 | 27 | 8 | 1 | 4 | 1 | 4 | 4 | 30 |
| pang4258.t1 | peroxidase | 1 | 3 | 1 | 7 | 10 | 30 | 7 | 0 | 5 | 1 | 6 | 4 | 27 |
| pang27494.t1 | protein FORGETTER | 0 | 5 | 2 | 7 | 10 | 27 | 8 | 1 | 7 | 1 | 5 | 4 | 27 |
| pang9402.t1 | putative poly [ADP-ribose] polymerase 3 [Cynara cardunculus var | 0 | 5 | 2 | 6 | 13 | 26 | 9 | 1 | 7 | 1 | 5 | 4 | 27 |
| pang10362.t1 | toll/interleukin-receptor-like protein [Cynara cardunculus var | 1 | 5 | 2 | 7 | 15 | 29 | 7 | 1 | 7 | 1 | 5 | 2 | 28 |
| pang18380.t1 | thaumatin-like protein 1b [Cynara cardunculus var | 1 | 4 | 1 | 7 | 14 | 27 | 7 | 1 | 6 | 1 | 6 | 3 | 29 |
| pang32543.t1 | putative late blight resistance protein homolog R1B-14 [Cynara cardunculus var | 1 | 4 | 2 | 5 | 14 | 28 | 7 | 1 | 7 | 1 | 6 | 2 | 28 |
| pang34131.t1 | putative disease resistance protein RGA3 [Cynara cardunculus var | 1 | 4 | 2 | 7 | 15 | 27 | 8 | 1 | 7 | 0 | 6 | 4 | 27 |
| pang14857.t1 | heat shock 70 kDa protein, mitochondrial-like isoform X | 1 | 3 | 2 | 7 | 12 | 27 | 9 | 1 | 7 | 0 | 4 | 3 | 30 |
| pang34577.t1 | peroxidase | 0 | 4 | 2 | 8 | 11 | 28 | 8 | 1 | 7 | 1 | 6 | 4 | 26 |
| pang30364.t1 | peroxidase | 1 | 3 | 2 | 9 | 10 | 27 | 6 | 1 | 7 | 0 | 6 | 3 | 31 |
| pang10941.t1 | peroxidase | 1 | 5 | 2 | 8 | 12 | 27 | 8 | 1 | 7 | 1 | 5 | 3 | 29 |
| pang12224.t1 | peroxidase | 1 | 5 | 2 | 8 | 12 | 28 | 9 | 1 | 7 | 1 | 4 | 4 | 27 |
| pang12571.t1 | peroxidase N | 1 | 5 | 1 | 7 | 12 | 29 | 8 | 1 | 6 | 1 | 5 | 3 | 28 |
| pang33269.t1 | TMV resistance protein N-like [Cynara cardunculus var | 1 | 4 | 2 | 8 | 14 | 30 | 8 | 1 | 6 | 0 | 3 | 4 | 29 |
| pang34174.t1 | putative late blight resistance protein homolog R1A-10 [Cynara cardunculus var | 1 | 5 | 1 | 7 | 14 | 24 | 8 | 1 | 7 | 1 | 5 | 4 | 31 |
| pang33383.t1 | peroxidase | 1 | 5 | 1 | 6 | 12 | 29 | 8 | 1 | 6 | 1 | 6 | 3 | 28 |
| pang32526.t1 | replication protein A 70 kDa DNA-binding subunit A-like [Cynara cardunculus var | 1 | 5 | 1 | 8 | 14 | 29 | 9 | 1 | 6 | 1 | 6 | 4 | 26 |
| pang14889.t1 | replication protein A 70 kDa DNA-binding subunit A-like [Cynara cardunculus var | 1 | 5 | 1 | 8 | 12 | 29 | 9 | 1 | 7 | 1 | 4 | 3 | 28 |
| pang5933.t1 | replication protein A 70 kDa DNA-binding subunit A-like [Cynara cardunculus var | 1 | 4 | 2 | 5 | 14 | 27 | 9 | 1 | 6 | 1 | 5 | 4 | 29 |
| pang32041.t1 | replication protein A 70 kDa DNA-binding subunit A-like [Cynara cardunculus var | 1 | 4 | 2 | 7 | 10 | 28 | 8 | 1 | 6 | 1 | 6 | 4 | 28 |
| pang29119.t1 | RNA polymerase sigma factor sigE, chloroplastic/mitochondrial [Cynara cardunculus var | 1 | 4 | 1 | 9 | 9 | 28 | 9 | 1 | 7 | 1 | 6 | 4 | 26 |
| pang16995.t1 | beclin-1-like protein [Cynara cardunculus var | 0 | 3 | 2 | 6 | 15 | 27 | 9 | 1 | 6 | 1 | 5 | 4 | 29 |
| pang34382.t1 | receptor-like protein kinase HAIKU2 [Cynara cardunculus var | 1 | 4 | 1 | 7 | 14 | 30 | 8 | 1 | 6 | 1 | 6 | 3 | 28 |
| pang28855.t1 | disease resistance protein RML1A-like [Cynara cardunculus var | 1 | 4 | 2 | 5 | 13 | 30 | 8 | 1 | 7 | 1 | 4 | 3 | 30 |
| pang33798.t1 | replication protein A 70 kDa DNA-binding subunit A-like [Cynara cardunculus var | 1 | 2 | 1 | 8 | 9 | 31 | 8 | 1 | 6 | 1 | 6 | 4 | 28 |
| pang11977.t1 | probable L-type lectin-domain containing receptor kinase S | 1 | 4 | 2 | 7 | 12 | 28 | 9 | 1 | 6 | 1 | 6 | 4 | 30 |
| pang15431.t1 | TMV resistance protein N-like [Cynara cardunculus var | 1 | 4 | 1 | 7 | 14 | 28 | 9 | 1 | 7 | 1 | 5 | 3 | 31 |
| pang33561.t1 | disease resistance-like protein DSC[Cynara cardunculus var | 1 | 4 | 2 | 7 | 11 | 31 | 8 | 1 | 7 | 1 | 6 | 3 | 28 |

**Supplementary Table S32:** Number of the stress-related genes across different regional pools.

| Regional gene pool | Abiotic stress related | | | Biotic stress related | | |
| --- | --- | --- | --- | --- | --- | --- |
|  | Maximum | Minimum | Accession with maximum genes | Maximum | Maximum | Accession with maximum genes |
| Iran-Afghanistan | 94 | 79 | CC47 | 109 | 89 | CC56 |
| Indian subcontinent | 96 | 69 | CC30 | 107 | 78 | CC37 |
| Near east | 89 | 80 | CC58 | 106 | 96 | CC61 |
| USA | 88 | 64 | CC116 | 106 | 75 | CC94 |
| Europe | 91 | 74 | CC62 | 105 | 81 | CC115 |
| Turkey | 90 | 77 | CC66 | 105 | 95 | CC64 |
| Far east | 83 | 71 | CC13 | 104 | 81 | CC13 |
| Kenya | 87 | - | CC76 | 103 | - | CC76 |
| Egypt | 83 | 67 | CC78 | 100 | 91 | CC81 |
| Unknown origin | 88 | 77 | S119 | 98 | 73 | S116 |
| Australia | 83 | - | CC1 | 97 | - | CC1 |
| Sudan | 83 | - | CC83 | 97 | - | CC83 |
| Ethiopia | 79 | - | CC75, CC79 | 97 | - | CC75 |

* Maximum and Minimum indicate the highest and lowest number of stress-related genes identified in accessions from each gene pool. “—” cases where minimum is not present because that gene pool represented by one accession
