# Supplementary material for: Improved reference assembly and core collection resequencing to facilitate exploration of important agronomical traits for the improvement of oilseed crop, Carthamus tinctorius L
Source: Gigascience. 2025 Dec 11;15:giaf151. doi: 10.1093/gigascience/giaf151 (PMC12888819; doi:10.1093/gigascience/giaf151)
Supplement: giaf151_GIGA-D-25-00014_Original_Submission [file giaf151_giga-d-25-00014_original_submission.pdf]

GigaScience

Improved reference assembly and core collection re-sequencing to facilitate exploration of important agronomical traits for the improvement of oilseed crop, Carthamus tinctorius L.

--Manuscript Draft--

**Manuscript Number:**	GIGA-D-25-00014
**Full Title:**	Improved reference assembly and core collection re-sequencing to facilitate exploration of important agronomical traits for the improvement of oilseed crop, Carthamus tinctorius L.
**Article Type:**	Research
**Funding Information:**	Department of Biotechnology, Ministry of Science and Technology, India (BT/Ag/Network/Safflower/2019-20; Sub Projects 3 and 4) — Dr. Arun Jagannath
**Abstract:**	Background<br><br>Safflower (Carthamus tinctorius L.) is a drought-resilient oilseed crop. Besides producing edible oil rich in oleic and linoleic acid, it is also used in biofuels, cosmetics, colouring dyes, pharmaceuticals and nutraceuticals. Despite its significant economic uses, availability of genetic and genomic resources in safflower are limited.<br><br>Results<br><br>We report an improved de novo genome assembly of safflower (Safflower_A2). A chromosome-level assembly of 1.15 Gb with telomeres and centromeric repeats, was constructed using PacBio HiFi reads, optical maps, Illumina short reads, and Hi-C sequencing. Safflower_A2 shows better contiguity, completeness, and high-quality annotation than previous assemblies. The assembly was further validated with the help of a single nucleotide polymorphism (SNP)-based linkage map. A genome-wide survey identified genes for disease resistance, oil quality, oil content and pigments. Employing the de novo genome assembly as a reference, we used resequencing data of a global core-collection of 123 accessions to carry out a SNP-based genome-wide association study, which identified significant associations for several traits of agronomic value, including seed oil content. Resequencing data was also applied for a pan-genome analysis which provided critical insights into genome diversity identifying an additional ~11000 transcripts and their functional enrichment that will be useful for region-specific breeding lines.<br><br>Conclusion<br><br>Our study provides insights into the genomic architecture of safflower by leveraging an improved genome assembly and annotation. Additionally, resources including high-density linkage map, marker-trait associations, and pan-genome developed in this study provide valuable resources for use in breeding and crop improvement programs by the global research community.
**Corresponding Author:**	Shailendra Goel, Ph.D.<br>University of Delhi<br>New Delhi, Delhi INDIA
**Corresponding Author Secondary Information:**	
**Corresponding Author's Institution:**	University of Delhi
**Corresponding Author's Secondary Institution:**	
**First Author:**	Megha Sharma
**First Author Secondary Information:**	

Powered by Editorial Manager® and ProduXion Manager® from Aries Systems Corporation

Order of Authors:	Megha Sharma
	Varun Bhardwaj
	Praveen Kumar Oraon, Ph.D
	Heena Ambreen, Ph.D.
	Rohit Nandan Shukla
	Vandana Jaiswal, Ph.D
	Arun Jagannath, Ph.D
	Shailendra Goel, Ph.D.
Order of Authors Secondary Information:	
Additional Information:	
Question	Response
Are you submitting this manuscript to a special series or article collection?	No
**Experimental design and statistics**<br><br>Full details of the experimental design and statistical methods used should be given in the Methods section, as detailed in our Minimum Standards Reporting Checklist. Information essential to interpreting the data presented should be made available in the figure legends.<br><br>Have you included all the information requested in your manuscript?	Yes
**Resources**<br><br>A description of all resources used, including antibodies, cell lines, animals and software tools, with enough information to allow them to be uniquely identified, should be included in the Methods section. Authors are strongly encouraged to cite Research Resource Identifiers (RRIDs) for antibodies, model organisms and tools, where possible.<br><br>Have you included the information requested as detailed in our Minimum Standards Reporting Checklist?	Yes
**Availability of data and materials**	Yes

Powered by Editorial Manager® and ProduXion Manager® from Aries Systems Corporation

All datasets and code on which the conclusions of the paper rely must be either included in your submission or deposited in publicly available repositories (where available and ethically appropriate), referencing such data using a unique identifier in the references and in the “Availability of Data and Materials” section of your manuscript.<br><br>Have you have met the above requirement as detailed in our Minimum Standards Reporting Checklist?	
GigaScience has policies and guidelines in place for the use of generative AI-writing tools such as ChatGPT. If you have used such writing tools to assist with writing the manuscript this must be declared and cited in the text. Authors should not list AI-writing tools and other AI-assisted technologies as an author or co-author and should acknowledge that they are fully responsible for text generated or refined by AI-writing tools.<p><br><br>A summary of use (particularly in the introduction or among methods) needs to be included at the end of the paper, and the outputs should also be included as a supplementary file hosted in GigaDB or other open repositories. Please read our guidelines for more information. <p><br><br>By submitting to GigaScience, you are aware of the journal's AI-writing tools policy, and if you have declared use of such tools below, you have acknowledged this where appropriate in your manuscript and have made a summary of use and outputs available. <p> <b>AI-assisted writing tools have been used in the preparation of this manuscript?	No

Manuscript

Click here to access/download;Manuscript;Safflower_A2_genome_gwas_pg_fi

Improved reference assembly and core collection re-sequencing to facilitate exploration of important agronomical traits for the improvement of oilseed crop, *Carthamus tinctorius* L.

Megha Sharma[1#], Varun Bhardwaj[1#], Praveen Kumar Oraon[1], Heena Ambreen[2], Rohit Nandan Shukla[3], Vandana Jaiswal[4], Arun Jagannath[1*], Shailendra Goel[1*]

1. Department of Botany, University of Delhi, Delhi 110007, India
2. Department of Biosciences, University of Exeter, Exeter, EX4 4QD, United Kingdom
3. Bionivid Technology Pvt. Limited, Bengaluru 560064, India
4. CSIR-Institute of Himalayan Bioresource Technology, Palampur, Himachal Pradesh 176061, India

*Corresponding authors: shailendragoel@gmail.com, jagannatharun@yahoo.co.in

#Co-first author- These authors contributed equally to the work.

Abstract:

Background - Safflower (*Carthamus tinctorius* L.) is a drought-resilient oilseed crop. Besides producing edible oil rich in oleic and linoleic acid, it is also used in biofuels, cosmetics, colouring dyes, pharmaceuticals and nutraceuticals. Despite its significant economic uses, availability of genetic and genomic resources in safflower are limited.

Results - We report an improved *de novo* genome assembly of safflower (Safflower_A2). A chromosome-level assembly of 1.15 Gb with telomeres and centromeric repeats, was constructed using PacBio HiFi reads, optical maps, Illumina short reads, and Hi-C sequencing. Safflower_A2 shows better contiguity, completeness, and high-quality annotation than previous assemblies. The assembly was further validated with the help of a single nucleotide polymorphism (SNP)-based

linkage map. A genome-wide survey identified genes for disease resistance, oil quality, oil content and pigments. Employing the *de novo* genome assembly as a reference, we used resequencing data of a global core-collection of 123 accessions to carry out a SNP-based genome-wide association study, which identified significant associations for several traits of agronomic value, including seed oil content. Resequencing data was also applied for a pan-genome analysis which provided critical insights into genome diversity identifying an additional ~11000 genes and their functional enrichment that will be useful for region-specific breeding lines.

Conclusion - Our study provides insights into the genomic architecture of safflower by leveraging an improved genome assembly and annotation. Additionally, resources including high-density linkage map, marker-trait associations, and pan-genome developed in this study provide valuable resources for use in breeding and crop improvement programs by the global research community.

Keywords: Safflower, Genome assembly, Core collection, Optical mapping, Resistance genes, Oleosins, Genome Wide Association Study, Candidate gene analysis, Haplotypes, Pan-genome

Background

Safflower *(Carthamus tinctorius L. 2n = 24)*, a member of the family Asteraceae, is a drought-resilient diploid oilseed crop. The crop produces edible oil with a unique profile consisting of nutritionally desirable unsaturated fatty acids [1]. The seed oil is also a rich source of phospholipids, phytosterols, phenols, and tocopherols which makes it highly valuable for diverse pharmaceutical and nutraceutical applications [2,3]. Safflower is currently cultivated across ~23 countries in a total area of ~100,000 hectares, producing one million tonnes of seed [4] . The largest global producers of the crop are Kazakhstan, Russian Federation, United States of America, Mexico and India, accounting for more than 85% of the global seed production [4]. Currently, safflower has a market value of $232.1 million, however due to its ability to grow under drought condition, its market is expected to increase to ~$355 million in a decade as drought conditions become more prevalent [5]. Despite its economic scope, safflower has observed a decline in acreage due to multiple factors like spiny nature of the plant, susceptibility to various biotic and abiotic stresses, and scarcity of cultivars with high yield and oil content [6].

Several studies have established the diverse genetic pool of safflower harbouring significant morphological, geographical and molecular diversity [7–10]. Further, primary and secondary diversification of safflower has also led to the development of varieties with distinct traits that emerged over time [11]. Owing to the vast variability observed in the crop, a single genome sequence would not sufficiently reflect the full repertoire of genes available in the crop. Resequencing multiple diverse genotypes encompassing the global diversity would enable generation of crucial genomic resources that would be valuable for expediting safflower breeding programmes. Currently, reference genomes are available for two safflower varieties, ‘Anhui 1’ [12] and ‘Chuanhonghua 1’ [13] which are region-specific and do not represent all desirable traits

of global relevance. Moreover, while resequencing of 220 accessions reported by [13] provides valuable insights, ~70% accessions of the sequenced panel are from the Chinese gene pool which does not adequately encapsulates the global diversity of the crop. This necessitates comprehensive exploration of global germplasm for study and deployment of broader genomic diversity.

Here, we report an improved *de novo* chromosome-level genome assembly of an elite safflower accession (hereafter designated as "Safflower_A2") characterized by several desirable traits of high agronomic value *viz*., high oil content (~47%), high oleic acid (~80%), higher seed yield, large head diameter (~24mm) and high head number (~60 per plant) (Supplementary Fig. S1). The genome assembly was derived through integration of multiple sequencing technologies including PacBio HiFi, Bionano optical maps, Hi-C and Illumina paired-end short reads. Additional support for anchoring the generated genome assembly was provided by a SNP based high-density genetic map generated by Genotyping by Sequencing (GBS) of a Recombinant Inbred Line (RIL) mapping population (F_8) developed using A2 as one of the parents. The genome assembly generated in this study have been utilized to generate a repertoire of Resistance Gene Analogs (RGAs) and genes associated with important traits *viz*., oil content, oil composition, and anthocyanin pigments for ready implementation in crop improvement studies. Further, we performed re-sequencing of 116 accessions of a core collection reported earlier by our group [14] and 7 additional accessions of agronomical importance and employed it for genome-wide association studies (GWAS) which revealed crucial loci for several agricultural traits of interest including seed oil content. Subsequently, we have constructed a pan-genome for safflower revealing distinct functional enrichments among pan-genes. To ensure direct availability of safflower resources and datasets to the scientific community, we present "Safflower Genome Resource", a comprehensive database housing several genomic resources including reference genome, protein-coding genes, Simple

Sequence Repeats (SSRs) and SNPs. The database will provide essential support to the global plant breeder community in advancing trait improvement efforts in safflower.

Analysis

Development and Evaluation of Safflower Genome assembly

The genome size of Safflower_A2 was estimated using flow cytometry and k-mer analysis. Flow cytometric analysis indicated an approximate size of 1.37 picograms (2C) which corresponds to ~1.34 Gb (Supplementary Fig. S2 a, b, c), and is in line with previously predicted size for safflower [15,16]. Genome size estimation using k-mer (k=17) distribution of HiFi long reads (Supplementary Table S1) gave an estimate of ~1.17 Gb (Supplementary Fig. S2 d) in consonance with earlier reports [12,13].

A combination of four different sequencing technologies including PacBio HiFi reads, optical mapping, Hi-C and Illumina paired-end sequencing was used for generation of the *de novo* reference genome assembly of safflower (Safflower_A2) (Supplementary Fig. S3). A total of 38.5 Gb of HiFi reads (~30X coverage) with a mean length of 13 kb and an accuracy of >99% were generated for the construction of contig-level genome assembly (Supplementary Table S2). Firstly, 3,444,538 HiFi long reads were constituted into a contig level assembly of 1.15 Gb comprising 2,427 contigs. Thereafter, the contigs were scaffolded using long optical maps, resulting in a scaffold-level assembly of 1.09 Gb comprising 31 scaffolds. Using paired-end linked reads produced by Hi-C sequencing, the 31 scaffolds generated above were integrated into 21 super-scaffolds and an additional scaffold that corresponded to the chloroplast genome. Finally, through careful manual curation, the scaffolds were anchored into 12 pseudochromosomes (2n=24),

resulting in a chromosomal level assembly of 1.09 Gb (Fig. 1a, Supplementary Fig. S4). The total length of the final anchored assembly was ~1.09 Gb with an N50 of 88.40 Mb and N90 of 81.10 Mb (Table 1). Our assembly further consist of 1,680 unplaced small contigs that were < 0.5 Mb in length with a total size of 68.5 Mb, making total length of assembly to 1.15Gb. The contiguity, completeness and accuracy of the genome was evaluated by mapping the short Illumina (99.29%) and long PacBio reads (95.29%) onto the generated assembly (Supplementary Table S3), Benchmarking Single Copy Orthologs (BUSCO) score (97.90%) (Supplementary Table S4), k-mer completeness score (97.73%), consensus quality value (QV) (68.31%) (Supplementary Table S5) and LTR assembly index (LAI) (22.49). The contiguous nature of the assembly enabled delineation of telomeres and centromeres in the safflower genome for the first time. The most frequent telomeric repeat was AACCCTG with counts ranging from 7 to 1,429. We identified telomeres at one end of nine chromosomes and at both ends of three chromosomes (Supplementary Fig. S5). We detected centromeric repeats on all the chromosomes. These were of four different lengths (342 bp, 348 bp, 349 bp and 350 bp) with counts ranging from 5 to 1,272 in the genome. The 349 bp repeat was the most abundant and present on all chromosomes except chromosomes 3 and 7 (Supplementary Fig. S5). Further, the chloroplast (cp) genome was assembled as a single circular contig of 153,026 bp (Supplementary Fig. S6). Its annotation identified 205 protein coding genes which was higher than the 127 cp genes reported earlier [15].

Construction of a high-density genetic linkage map and assignment of chromosomes

A total of 151 Gb paired-end GBS data was generated for 121 lines of a RIL population (A2 X A1; designated "Population A") with average coverage of ~1.15x (0.33x-1.82x) per individual. Variant calling yielded 1.49 million SNPs, which were filtered using stringent criteria

(Supplementary Table S6) and a final set of 15,732 high quality SNPs were used to construct the first SNP-based high-density linkage map in cultivated safflower comprising 12 linkage groups (LG1-LG12; Supplementary Fig. S7, Supplementary Table S7). The map spanned 1,581.05 cM, with linkage groups ranging in length from 71.41 cM (LG 7) to 209.08 cM (LG 1). The average number of markers per linkage group was 1,311 and ranged from 3,587 in LG 8 to 217 in LG 10. Average marker distance was 9.28 per cM, ranging from 5.72 per cM (LG3) to 24.76 cM (LG8). This genetic linkage map was anchored to the genome assembly, which showed concordance with the genetic maps confirming the accuracy of assembled genome (Supplementary Fig. S8)

Full-length transcriptome sequencing and detection of alternative splicing events

Transcriptomic libraries were generated from eight different tissues of safflower *viz*., shoot, leaf, root, flower, bud and various seed developmental stages (5 DAP, 10 DAP, 20 DAP and 30 DAP; DAP: days after pollination) (Supplementary Table S8). Long read PacBio sequencing yielded 3,772,953 circular consensus sequences (CCS) reads, which resulted in 2,22,133 full-length high-quality transcripts. The obtained transcripts were aligned to the repeat-masked reference Safflower_A2 genome. The alignment data was used as evidence for gene prediction and detection of alternative splicing events. SUPPA2 detected seven types of Alternative Splicing (AS) events (totalling 3,826), namely: retained intron (RI), skipping exon (SE), alternative 5'/3' splice site events (A5S/A3S), alternative first/last exons (AF/AL) and mutually exclusive exons (MX). A5S were the most abundant and MX were the rarest type of splicing events accounting for 57.05% and 0.26% of total local events, respectively (Supplementary Table S9).

Annotation of the repeatome and detection of LTR for the genome expansion

We identified 787.75 Mb of repetitive elements constituting ~71.3% of the total length in the Safflower_A2 genome (Fig. 1b). Retrotransposons [(class I Transposable Elements (TE)] were the most dominant repetitive category with its sub-class long terminal repeats (LTRs) representing the major component (43.6%) of repetitive elements comprising 22.32% Ty3/*Gypsy* and 21.3% Ty1/*Copia* elements. The non-LTR retroelements (long interspersed nuclear elements: LINEs and short interspersed nuclear elements: SINEs) constituted 0.87% of the repetitive elements. DNA transposons (class II elements) constituted around 17.63% of the total repetitive elements (Supplementary Table S10, Supplementary Fig. S9). DNA transposons were further classified into Tandem inverted repeats (TIR; 7.13%), Miniature inverted-repeat transposable element (MITES; 7.61%) and Helitrons (2.88%). Around 11.4% of the identified TEs were structurally intact. Simple Sequence Repeats (SSRs) constituted 0.38% of the total repeats (Fig. 1d).

Using Domain based Annotation of Transposable Elements (DANTE), we assigned 70% of the Ty1/*Copia* elements and 65% of the Ty3/*Gypsy* elements detected by the EDTA to distinct LTR lineages (Supplementary Table S11). Among these, Ty1/*Copia*/SIRE and Ty3/*Gypsy* /Tekay were identified as the most abundant lineages in the safflower genome, with copy numbers of 65,067 and 71,143, respectively. Our results are concordant with the other members of Asteraceae [16–19]. Based on the presence of complete functional domains of LTR TEs, 3,497 Ty1/*Copia* elements and 4,716 Ty3/*Gypsy* elements were classified as intact (Supplementary Fig. S10 a,b). Further, 2,195 Ty1/*Copia* and 2,879 Ty3/*Gypsy* elements were defined autonomous owing to the presence of target site duplication (TSD) and primer binding sites (PBS). At the hierarchical level of lineages, Ty1/*Copia*/SIRE (1,742) and Ty3/*Gypsy*/Retand (2,193) exhibited the highest number of complete members. Phylogenetic tree of Ty1/*Copia* (Supplementary Fig. S10c) and Ty3/*Gypsy*

(Supplementary Fig. S10d) subfamilies were divided into distinct clades, except Ty1/*Copia*/SIRE. An assessment of insertion times revealed that 87.19% of the complete LTR-TEs were inserted within the past 1 million years, indicating that transposon activity might be one of the major drivers of genome expansion in safflower (Supplementary fig S10 e). Further in-depth analysis highlighted Ty3/*Gypsy*/Tekay, Ty3/*Gypsy*/Retand, Ty3/*Gypsy*/Athila, Ty1/*Copia* /SIRE, Ty1/*Copia* /TAR, and Ty1/*Copia*/Angela as the dominant contributors to this recent transposon burst. Notably, the most recent transposon burst aligns with the recent whole-genome duplication (γ) event in safflower [13].

Non-coding RNA (tRNA and rRNA) genes were also surveyed in Safflower_A2 genome assembly. We identified 4,763 rRNA genes comprising 3,619 5S type, 398 5.8S type, 377 18S type and 370 28S type. Additionally, 1,110 tRNA genes were predicted, of which 766 coded for 20 amino acids (Supplementary Table S12).

Annotation of protein-coding genes

The safflower genome harboured a total of 59,995 transcripts with an average of ~4.4 exons per transcript and at an average intergenic distance of ~14 kb, identified by evidences from ab initio homology and RNA-seq methods (Fig. 1c, Table 1). After clustering through cd-hit, these 59,995 transcripts corresponded to 39,945 unigene models (at 80% similarity). The derived transcript set yielded a combined BUSCO score of 91.5%, indicating completeness of the safflower gene repertoire (Supplementary Table S13). Collectively, ~80% (47,704) of the predicted protein coding transcripts were annotated with at least one functional term using publicly available databases RefSeq, Gene Ontology (GO), Enzyme Code (EC), Cluster of Orthologous Groups

(KOG), Kyoto Encyclopaedia of Genes and Genomes (KEGG) and Interproscan. In addition, we could delineate a total of 2,893 transcription factors and regulators. We also identified 1,587 protein kinases were also identified (Fig. 2 a-d, Supplementary Table S14).

Identification of Resistance Gene Analogs (RGAs)

To facilitate genetic dissection of disease resistance in safflower, we determined a total of 2,461 putative genes encoding for RGAs, that were categorized into 24 major classes based on their constituent domains (Fig. 3a-b, Supplementary Table S15). Among the RGAs, the most characterized and well-known gene family for disease resistance in plants is the Nucleotide-binding-site Leucine-rich repeat Receptor (NLR) gene family [20] which includes Toll/interleukin-1 receptor- Nucleotide-binding site- Leucine-rich repeat (TNL), Coiled-coil-Nucleotide-binding site- Leucine-rich repeat (CNL), and resistance to the powdery mildew RPW8-NBS-LRR (RNL) genes. The NLR gene family is known to be associated with effector-triggered immunity (ETI) [21]. We identified 236 non-redundant, high-confidence NLR genes in the safflower genome of which 191, 38 and 7 members encode TNL, CNL, and RNL genes, respectively. Phylogenetic analysis of all the identified NLR genes segregated TNL, CNL and RNL genes into distinct clades (Fig. 3c). The localization of the NLR-RGAs on different safflower chromosomes exhibited biased distribution with chromosomes 2, 6 and 11 encompassing high proportions of RGAs while no NLR genes were found on chromosomes 5 and 7 (Fig. 3d, Supplementary Fig. S11). Further, to determine the Ka/Ks of the NLR genes within the safflower genome, 75 closely related homologous gene pairs identified based on their phylogenetic relationships were used which revealed an average Ka/Ks value for TNL, CNL and RNL to be 0.49, 0.52, and 0.33, respectively (Fig. 3e). All the NLR genes showed a positive value (<1)

indicating that these genes are under positive selection maintaining protein conservation. However, we also detected two TNL gene pairs exhibiting Ka/Ks >1 indicating purifying selection on these loci. We delineated several types of duplication events including tandem (163), proximal (50), dispersed (11), and segmental (12), which accounted for ~70%, 21%, 4.6%, and 5% of all NLR genes, respectively. We also analysed intra-genomic synteny among NLR genes of safflower which identified 6 NLR gene pairs located on chromosomes 4 and 11 in the safflower genome (Fig. 3f).

Genome-wide identification and phylogenetic analysis of gene families associated with oil quality (FAD), oil content (Oleosins) and pigments (PAL and CHS)

To elucidate genetic diversity and gene duplication of gene families pertaining to agronomic traits such as oil content, composition and flower colour, genome-wide identification and phylogenetic analysis was conducted for gene families including Fatty Acid Desaturase (FAD), Oleosins, Phenylalanine Ammonium Lyase (PAL) and Chalcone Synthase (CHS). We identified 30 putative FAD genes localized on all safflower chromosomes except chromosome 1 (Supplementary Fig. S12a, Supplementary Table S16). Physicochemical properties of FAD protein sequences are given in Supplementary Table S16, indicating the wide variety range of isoelectric points (pI) and molecular size. Predicted intracellular localization predominantly placed most genes in plasma membrane (13) and endoplasmic reticulum (ER) (14). Motif analysis identified a varied distribution of motifs ranging from 1-8 in number (Supplementary Fig. S13a). Phylogenetic analysis of FAD genes from safflower (31), *A. thaliana* (25) and *Helianthus annuus* (71), revealed multiple clades where sequences from safflower and sunflower were clustered together as expected since both are members of the Asteraceae family (Supplementary Fig. S14a).

We identified 13 Oleosin genes in the Safflower_A2 genome, distributed on chromosomes 1, 5, 6, 9, 10, 11 and 12 (Supplementary Fig. S12b, Supplementary Table S16). Physicochemical properties of these proteins (Supplementary Table S16) indicated a both basic and acidic pI, confirming their role in oil body stabilization across different pH conditions. The number of motifs ranged from 4-6 per paralog (Supplementary Fig. S13b). Phylogenetic tree constructed with 16 sequences from *A. thaliana*, 12 sequences from *H. annuus* and 13 sequences from safflower revealed 2 major clades (Supplementary Fig. S14b). Two paralogs from Arabidopsis, namely, AT2G5890.1 and AT2G5890.2, did not cluster into the two clades. All predicted oleosin candidates from safflower were closer to sunflower revealing high homology between sequences from the two species.

We identified 9 PAL paralogs in the Safflower_A2 genome which were distributed on chromosomes 1, 6, 10 and 12 (Supplementary Fig. S12c, Supplementary Table S16). Most of these genes were clustered in groups. A summary of their physicochemical properties is given in Supplementary Table S16. Motif analysis revealed that three members contained a single motif while the remaining 6 members had 10 motifs each (Supplementary Fig. S13c). A phylogenetic tree constructed with protein sequences from *A. thaliana* (4), *H. annuus* (11), and safflower (9) exhibited clustering of all sequences into a single clade. The high degree of sequence similarity observed among PAL genes suggests strong evolutionary conservation, indicating their critical role in the phenylpropanoid pathway (Supplementary Fig. S14c).

A total of 8 CHS genes distributed on chromosomes were identified in this study (Supplementary Table S16, Supplementary Fig. S12d) and physicochemical properties of their encoded proteins are summarized in Supplementary Table S16. The number of distinct motifs varied from two to nine (Supplementary Fig. S13d). To explore the evolutionary connections among CHS genes, an

ML tree was constructed using sequences from *A. thaliana* (1), *H. annuus* (8) and Safflower (8) from our genome. The resulting unrooted tree (Supplementary Fig. S14 d) revealed 2 major clades. First clade consisted of Arabidopsis CHS gene (AT5G13930.1) and a subset of sunflower and safflower CHS genes. This clade represents a more divergent lineage, with Arabidopsis showing the most distinct branching. Second clade was further divided into smaller subclades, reflecting species-specific gene expansions and duplications within the Asteraceae family. The close grouping of some safflower and sunflower genes suggested conservation of CHS genes between these two species, indicating their functional importance in flavonoid and phenylpropanoid pathways.

Exploring genetic basis of various agronomically important traits in safflower

A total of 2.05 Tb of resequencing data was generated for 123 accessions with an average coverage of 15.3x per accession (Supplementary Table S17). Variant calling of the safflower core collection identified ~13 million raw SNPs and ~2.3 million small indels. We obtained a final set of ~1.8 million SNPs after robust filtering (Supplementary Table S18; Supplementary Table 19).

The population structure analysis of core collection identified four major clusters (K=4; membership coefficient ($q_i \geq 0.5$) designated as ADI, ADII, ADIII and ADIV and comprising 62, 6, 19 and 16 accessions, respectively (Supplementary Table S20). ADI comprised large number of accessions from different continents while ADIII delimited primarily Indian accessions (Fig. 4a). Principal component analysis (PCA) was performed and the first two principal axes, PC1 (18%) and PC2 (13%) were plotted (Supplementary Fig. S15a). All accessions from ADI clustered together in quadrants 2 and 3 of PCA. Quadrant 3 comprised accessions from USA while quadrant

2 consisted of accessions from other regional gene pools. All accessions from ADIII clustered in quadrant 4 whereas ADII and ADIV accessions were clustered in quadrant 1. Based on the phylogenetic tree, four major clusters (NJI – NJIV) were observed (Fig. 4b). Most ADI accessions clustered together in NJI. ADII accessions, along with some ADI accessions, were in NJII. NJIII comprised accessions from ADIII while NJIV had accessions from ADIV and ADI. Our population genetic structure analysis based on ADMIXTURE, PCA and phylogenetic analysis was able to infer consistent phylogenetic relationships between the accessions. F_{ST} divergence was estimated between the populations. AD1 and AD III showed high genetic divergence (F_{ST} = 0.45) whereas AD II and AD IV showed minimum genetic divergence (F_{ST}= 0.239). Linkage decay (LD) of the re-sequenced accessions indicated that the LD decreases to half (r^2 = 0.15) from its maximum at ~6 kb (Supplementary Fig. S15b). The overall LD is similar for all chromosomes ranging from 0.15 to 0.10 which is in consonance with earlier reports on Sunflower [22].

Phenotypic data for all traits represented in the core collection is sourced from our earlier study [14] which has been shown to have broad spectrum of variability with a normal distribution. Pearson's correlation analysis demonstrated strong concordance (<90%) in phenotypic data across two growing seasons for traits including oil content (OC), plant height (PH), and days to 50% flowering (DTF). However, for traits like 100-seed weight (100SW), the number of primary branches (PB), and the number of heads (HN), the correlation was moderate (0.61 to 0.81). SNPs (~1.8 million) generated were further filtered to a final set of 320,399 (filtering criteria summarized in Supplementary Table S21 and SNP distribution in Supplementary Table S22 and Fig. 4c). We identified 1,611 significant Marker Trait Associations (MTAs) collectively for all eight agronomic traits over two growing seasons at p<0.0001. QQ plots were analysed to identify the best fitting models for each trait and the analysis revealed that multi-locus models were best suited for

detecting significant associations. Only the MTAs which were following specific criteria were retained (outlined below) and called as Quantitative Trait Nucleotides (QTNs). For traits OC, PH, and DTF, the QTNs were those associations which were consistently identified across all three multi-locus models in both growing seasons. In contrast, traits HN, PB, and 100SW showed more seasonal variability, hence QTNs were defined as those associations which were present in at least one multi-locus model and both growing seasons. Oleic acid (OA) and linoleic acid (LA) data were available for only one growing season; thus, QTNs for these traits were identified using the best-fitting model, MLMM. A total of 81 QTNs were identified for eight traits on all chromosomes (Fig. 4d, Supplementary Table S23).

LD block analysis revealed that the average size of the LD block in the QTN region is ~ 6.7 kb (Supplementary Table S24). Consequently, candidate genes were searched within 7 kb upstream and downstream of the QTN positions. A total of 24 candidate genes corresponding to 23 QTNs were identified across all eight traits based on their putative functions reported in the literature. Haplotypes were identified in the LD block for QTNs with putative functions, and their associations with the phenotypes were further analysed (Table 2, Supplementary fig. S16).

Being an oil crop, OC is the most important trait for safflower. Two QTNs OC1 and OC8 (Fig. 5a, b) marked the gene BIG GRAIN 1-like protein, a positive regulator of auxin transport and signalling, reported to control grain size in rice by modulating cell division [23]. Interestingly, we found OC1, OC8, and BIG GRAIN within the same haploblock (Fig. 5c). OC8_H01 is most geographically distributed haplotype whereas OC8_H04 is the least represented by the haplonets (Fig. 5d). OC8_H05, and OC8_H06 encode for moderate to high oil (<25%) (Fig. 5e-f, Supplementary fig. S16 a-b). Another QTN, OC2 (on chromosome 2) was located near a gene coding for a pentatricopeptide repeat-containing protein (PPR) (InterPro: IPR002885), which is an

integral membrane protein (GO:0016020). PPR protein (EMP9) has been shown to affect the seed development in Maize [24] . OC2_H03 is the haplotype that encodes for the high oil (25% to 50%) (Supplementary fig. S16 c-d). For trait 100SW, QTN SW23 is downstream of a CYP57 isoform, known to regulate cell division and elongation processes that influence seed size and weight in Arabidopsis [25]. QTN SW3 was in the regulatory region of the xyloglucan galactosyltransferase gene, involved in hemicellulose modification of primary cell walls of most dicotyledonous plants [26]. Haplotype analysis detected that SW23_H02 encodes for the high seed weight whereas SW3_H02 encodes for low to moderate seed weight (2.5g-5g) (Supplementary fig. S16 e-h). SW31 lies in the upstream region of the oleosin-B6-like protein, which is associated with lipid droplet stability and oil body formation in seeds, potentially playing a role in seed weight and energy storage[27]. QTN SW37 lies near FRIGIDA-ESSENTIAL 1 gene, associated with biological processes connected with reproduction, such as embryonic development and seed maturation [30]. Another QTN SW10 is located downstream of the gene encoding RNA-binding protein 2 (RBP) implicated in post-transcriptional regulation by directly interacting with target mRNAs and influencing various aspects of mRNA metabolism. RBPs such as APUM24 are known regulators of seed development [28]. For oleic and linoleic acid, we detected four QTNs (OA_LA15, OA_LA17, OA_LA20, and OA_LA33) on chromosome 5 encoding for cytochrome P450 71A4-like, which is involved in fatty acid catabolism via epoxidation [29]. OA_LA15 and OA_LA17 are present within the same haploblock. Haplo-pheno analysis (Supplementary fig. S16 i-k) suggests that QTNs associated with cytochrome P450 71A4-like resulted in low to moderate OA, however no correlation was detected with LA. It is possible that FAD and cytochrome P450 71A4 are competing for the same substrate (oleic acid), however, further studies are required to explore this hypothesis [30].

For DTF, we identified QTN DTF2 is associated with the gene encoding E3 ubiquitin-protein ligase UPL1-like, known to regulate various developmental processes, including flowering (Shu and Yang, 2017). QTN DTF10 was found to be in the proximity of E3 ubiquitin-protein ligase COP1-like, which accelerates the degradation of GIGANTEA (GI) via the 26S proteasome, thereby delaying flowering under low-temperature conditions [31]. Haplotype analysis identified DTF2_H05 and DTF2_H06, associated with a reduction in number of days to flowering whereas DTF10_H02 and DTF10_H03 were responsible for increasing DTF (Supplementary fig. S16 o-s).

For pre-harvest traits such as PH, QTN PH3 (on chromosome 2) is downstream of the gene encoding a vignain-like protein, a cysteine protease, involved in resource allocation or tissue remodelling, which are critical during active growth [32]. For PH3, we detected two haplotypes PH3_H02 led to the increased height in season 1 but not in season 2 (Supplementary fig. S16 t-u). QTN PH4 lies in the vicinity of the gene coding for trafficking protein particle complex subunit 6B, a component of the transport protein particle (TRAPP) complex, which is involved in vesicle transport, important for plant growth, and development [33] . For PB, QTN PB1 (on chromosome 12) lies downstream to gene coding for another PPR. Another QTN PB9 lies upstream of a gene encoding F-box/LRR-repeat protein 14, involved in auxin signalling pathway and programmed cell death. Haplotype analysis revealed PB1_H02 shows an association with low number of primary branches in year 1 but not in season 2 (Supplementary fig. S16 v-w). For HN, QTN HN5 lies upstream of the gene coding for alpha-xylosidase 1, critical for maintaining cell wall integrity and enhanced cell wall loosening in the elongating flower stem, however only one major haplotype was detected (Supplementary fig. S16 x-y) [34].

Pan-genome assembly, annotation, and Presence Absence Variation analysis

Using the three chromosomal-level safflower assemblies (this study, [12,13]) and accessions from core collection (Fig 6a), we assembled a pan-genome of safflower of 1.26 Gb (henceforth called Safpg_v1) including an additional 99.9 Mb which represents an increase of 8.9% over the reference genome. This additional sequence comprised 63,814 contigs with a length range from 1 kb to 47.85 kb. We predicted 11,479 transcripts which increased the total number of predicted transcripts of the safflower genome to 71470 (size> 150 bp). Functional annotation of the newly added transcripts against the Refseq database, assigned functions to ~5300 transcripts. Furthermore, domains were identified through InterProScan exhibiting domains related to reverse RNA transcriptase, Zinc-finger, reverse transcriptase, disease resistance gene, and Leucine rich repeats. GO mapping of these transcripts indicated that these additional genes were largely involved in metabolic processes helping the plant in combating biotic and abiotic stresses [35].

The breeding history and genetic changes of the crop can be revealed in the Presence-Absence Variation (PAV) among different accessions. Through the PAV matrix (Fig 6b), we identified 14,542 cores, 33,184 soft cores, 20,699 shell, 1,452 cloud, and 214 private genes (Fig 6c). All the core and soft core were assigned as conserved (47,726) whereas shell, cloud and private genes were assigned as variable (22371) genes. Modelling of the pan-genome showed that the number of core genes remain constant however, the number of pan-genes continues to increase with the addition of the new genome suggesting that saturation has not yet been achieved (Fig 6d). We detected larger gene length and more exons in the core genes as compared to variable genes, showing the conserved nature and long evolutionary history of core genes and comparably a new origin of the variable genes. The maximum number of the genes were contributed by NC132, an accession from the USA. The largest number of the variable genes were harboured by CC62, a

Russian accession. The maximum number of shell genes were present in Anhui1 belonging to China and accession CC25 from India. Interestingly, an Indian accession, CC38 tends to be the most diverse accession of the core collection with 1,326 cloud genes and 203 private genes. CC108 (USA), CC72 (Spain), CC51 (Iran) and NC132 (USA) are also diverse accessions based on cloud genes. The number of core and soft-core genes are similar among the regional pools. The highest number of variable genes were seen in accessions of the Indian subcontinent and USA depicting their high genetic diversity (Fig. 6e).

Discussion

In the present study, we generated an improved safflower genome assembly, which was validated with the help of a GBS-based linkage map. This reference assembly was used for calling SNPs from core collection resequencing data. GWAS and candidate gene analysis was conducted leading to identification of genomic regions associated with important safflower agronomic traits which is an invaluable information for crop improvement. The resequencing data was also used to identify a pan-genome for the crop, further enriching the genomic resources for safflower.

Improved quality of the Safflower_A2 genome assembly

We report an improved, contiguous and richly annotated genome sequence of a safflower accession (Safflower_A2) with high oil and nutritionally desirable high oleic acid content. The two earlier reported chromosomal-level genome assemblies were derived from Chinese safflower accessions, Anhui [12] and Chuanhonghua 1 [13] which were rich in the linoleic acid. K-mer

analysis (K=17) of our genome demonstrated a genome size of 1.17 Gb with low heterozygosity and high repeat content, which is in consonance with earlier studies [12,13]. The estimated genome size through flow cytometry was 1.34 Gb, which was 8.7% higher as compared to k-mer distribution analysis. A lower estimation by k-mer analysis could be attributed to the high amount of repeat sequences in the safflower genome [36]. The genome assembly of Safflower_A2 is 1.15 Gb which is better than the Anhui 1 genome assembly (1.07 Gb). Although the primary genome assembly of Chuanhonghua 1 was reportedly higher at 1.17 Gb, our analysis shows fragmented nature of this genome (explained by indices described below). Hence, comparison of chromosomal lengths could not be accurately conducted (Supplementary Fig. S17a). Whole genome alignment of our Safflower_A2 assembly with Anhui 1 genome revealed one-to-one alignment representing integrity and consonance between the two genomes (Supplementary Fig. S17b). However, Safflower_A2 chromosome lengths were significantly longer than those of Anhui 1 (Supplementary Fig. S17c, S17d). Safflower_A2 genome also showed higher completeness with a significantly higher BUSCO score (97.9%) as compared to Anhui 1 (90%) and Chuanhonghua 1 (89.25%) genomes. A higher rate of mapping back of raw long reads of Safflower_A2 genome (99.29%) as compared to Anhui 1 (98%) and Chuanhonghua 1 (93%) genomes further support superior quality of the safflower_A2 genome (Table 1). We have validated our genome assembly using a high-density linkage map constructed using GBS data from a RIL population. A high concordance between the genome assembly and the linkage map confirms the accuracy and completeness of our genome assembly. As compared to earlier studies, we detected higher number of LTRs in our genome, which is supported by a high LAI score (22.49) that is comparable to that of the Anhui 1 genome (23.08) and significantly higher than that of Chuanhonghua 1 genome (14.65; Table 1). Further, we detected telomeric repeats at both ends of three chromosomes and

one end of nine chromosomes which indicates near completeness of the Safflower_A2 genome assembly. We also detected centromeric repeats of four different lengths (342 bp, 348 bp, 349 bp and 350 bp) on all the chromosomes representing resolution of the repetitive regions of the genome. Centromeric and telomeric regions are being reported for the first time in the Safflower_A2 genome. We detected 59,995 protein coding transcripts corresponding to 39,945 Unigene models (at 80% similarity) after clustering. A higher number of unigenes can be attributed to improved quality of the safflower_A2 genome and use of BRAKER3 pipeline in the current study which outperforms MAKER2 (used in earlier studies) in prediction of exons, genes and transcripts [37]. Use of a comprehensive Iso-Seq dataset as evidence for annotation, improved the BUSCO score of predicted protein sequences (Table 1). Furthermore, our results are in consonance with the recent reports on other plant species including *Eriobotrya japonica* [38] and *Lonicera caerulea* [39].

Identification of genes associated with disease resistance and other traits of agronomic value

Generation of a repertoire of R genes and their analysis is important to facilitate breeding for resistance to biotic stresses. A comprehensive analysis of R genes in the safflower genome is lacking. We identified 236 non-redundant, high-confidence NLR genes in the safflower genome which would expedite the cloning of resistance genes and enhance our understanding of their associated mechanisms [40]. The protein coded by an NLR gene consists of the NBS domain, which hydrolyses energy, and the LRR domain for pathogen recognition [41]. The TIR domain is involved in downstream signalling of Enhanced Disease Susceptibility 1 (EDS1), providing immunity against biotic and hemi-biotic pathogens, exhibiting both local and systematic

immunity. In contrast, non-TNL domains (CNL and RNL) are involved in NPR1-mediated immunity, contributing to broad-spectrum systemic immunity in plants [42]. In our study, the ratio of TNL and non-TNL is ~4:1, suggesting higher contribution of TNL in disease resistance of Safflower. The number of TNL-RGAs is higher than non-TNL RGAs in safflower, which is in contrast with sunflower wherein non-TNL genes were higher [43]. However, our observations are in consonance with those of Arabidopsis [44]. In our study, phylogenetic relationships among NLR genes indicated that non-TNL are more diverse and less conserved than TNL genes which is in consonance with earlier studies [21,45]. Interestingly, we found RNL and CNL groups together in the phylogenetic tree as a sister clade in contrast to observations in sunflower wherein RNL and TNL were found to be sister clades. However, our results were similar to results reported in other Angiosperms [46].

We analysed the Safflower_A2 genome for important genes contributing to oil composition (*FAD*), oil content (Oleosins) and pigment production (*CHS* and *PAL*). Unsaturated fatty acids play a key role in plant growth and development and *FAD* genes are responsible for introducing double bonds at specific positions in fatty acid chains. Previous reports on *FAD* genes in safflower [12,13] identified 20 *FAD2* paralogs. *FAD2* proteins are involved in the conversion of oleic acid to linoleic acid and are therefore key regulators of fatty acid composition in seeds. In contrast to earlier reports, our study focuses on the entire *FAD* gene family, to gain a broader overview of genetic diversity and functional overview of all *FAD* genes in safflower. We identified 31 *FAD* genes in our genome, each with a characteristic desaturase domain. Phylogenetic analysis of *FAD* genes from *Arabidopsis thaliana* (25 genes), *Helianthus annuus* (71 genes), and safflower (31 genes) revealed multiple clades with interspersed sequences from safflower and sunflower, suggesting significant gene duplication events followed by diversification within the Asteraceae

family. Clustering of safflower and sunflower sequences together in the same clades also implied that some *FAD* genes have conserved functions across these species. A wide range of theoretical pI (6.61 to 9.63) indicated subcellular localization of FAD proteins with acidic pI proteins localised in vacuoles. Those with neutral and basic pI values localised to endoplasmic reticulum and chloroplast, respectively which were cross-verified by *in-silico* subcellular localization analysis.

Oleosins are small hydrophobic proteins that are embedded in the phospholipid monolayer surrounding oil bodies and play a crucial role in their formation and stabilization in safflower seeds [48]. An earlier study on Oleosin in safflower [49] identified 8 paralogs wherein expression of oleosin genes was significantly correlated with high oil accumulation in selected safflower genotypes. In the current study, we identified thirteen oleosin paralogs with a broad spectrum of theoretical pI, highlighting functional versatility. *In-silico* subcellular localization analysis categorized all identified genes as membrane-bound proteins confirming their role as stabilizers of oil bodies. Phylogenetic analysis indicated high homology between sunflower and safflower oleosin members, highlighting a similar role of seed oil body stabilization in both species.

PAL catalyses the first step of the flavonoid biosynthetic pathway. Flavonoids serve as precursors for production of anthocyanins, a major class of pigments in safflower. Nine PAL paralogs were identified in our study. Theoretical isoelectric points for all except two paralogs were acidic and subsequently (Supplementary Table S16), they were targeted to cytoplasm while those with basic pI were predicted to be in nucleus and extracellular space. Phylogenetic analysis revealed that all sequences utilized in the study showed high sequence diversity suggesting strong selection pressure, which could have promoted rapid diversification.
Chalcone synthases catalyze the first step of the flavonoid pathway leading to the production of 2′,4,4′,6′-tetrahydroxychalcone (THC). Eight CHS genes identified in the current study were found

to be distributed on chromosomes 1, 6, 7 and 8 (Supplementary Table S16). Understanding the genetic diversity of *FAD*, Oleosin, *PAL* and *CHS* genes can guide breeding programs in safflower and other crops. Additionally, the diverse nature of these genes provides a rich source for genetic engineering aimed at improving the oil composition, oil content and secondary metabolite production.

Exploring genetic basis of various agronomically important traits in safflower

To decipher the full repertoire of genes available in a crop, availability of sequence data from diverse accessions is important. However, due to the significantly large diversity harboured by safflower, resequencing of a core collection is more practical, cost-effective and timesaving. Thus, we re-sequenced a core collection comprising 123 accessions developed earlier by our group [14]. This core collection was developed through maximization strategy from a germplasm collection of 531 accessions representing the global genetic, morphological and geographical diversity available for safflower. In the earlier study by [13], a collection of 220 accessions was re-sequenced. However, this collection mainly consisted of accessions of Chinese origin (149) with a limited representation of global genetic diversity. Through ADMIXTURE analysis, we predicted four hypothetical sub-populations and identified nineteen admixtures in our population. Our earlier study [50] based on SSR data indicated two clusters (highest peak at K=2 and a smaller peak at K=4) and 16 admixtures. Use of a larger SNP dataset in the current study increased the resolution. We further inferred genetic relationships among accessions using distance-based methods, NJ and PCA, and observed its concordance with Bayesian based ADMIXTURE methods. All accessions from ADI clustered together in quadrants 2 and 3 of PCA. Quadrant 3 comprised accessions from

USA while quadrant 2 consisted of accessions from other regional gene pools. All accessions from ADIII clustered in quadrant 4 whereas ADII and ADIV accessions were clustered in quadrant 1. Most ADI accessions clustered together in NJI. ADII accessions, along with some ADI accessions, were in NJII. NJIII comprised accessions from ADIII while NJIV had accessions from ADIV and ADI. We observed lack of geographical structuring among accessions of the core collection which could be attributed to maximization strategy used for core collection development, emphasising allelic diversity with minimum redundancy [14]. Further, low F_{st} and kinship value between the sub-populations represents the low differentiation and low genetic similarity between the sub-populations. Thus, low molecular relatedness and weak population structure among the core collection accessions reduce the likelihood of false marker-trait associations [49,50], affirming its appropriateness for association mapping.

GWAS is a powerful tool in crop genetics, providing comprehensive insights into the genetic basis of complex traits across diverse germplasm accessions, accelerating breeding programs by eliminating the need of developing biparental populations [51]. In the current study, five different models including two single-locus models (GLM and MLM) and three multi-locus models (MLMM, FarmCPU and BLINK) were utilized to detect significant MTAs. We report a total of 81 QTNs, wherein 9 QTNs were identified for OC, 7 for PH, 11 for OA and LA, 31 for 100SW, 3 for HN, 6 for PB and 14 for DTF (Supplementary table S24). In a previous study [13], QTNs have been reported for oil content, flower colour, ball (head) number, branch height, bract spine, first branch number, plant height, and stem diameter. While QTNs reported in the study show strong correlations with traits, EMMA (Efficient Mixed-Model Association) model for detection of MTAs is computationally less robust compared to MLMM, FarmCPU and BLINK used in the current study, especially for large datasets. Thus, by leveraging a multi-model GWAS approach,

phenotypic data from 2 consecutive growing seasons, and a globally diverse germplasm collection, our study provides more robust QTNs. The QTN-marked regions were subjected to candidate gene analysis, identifying genes invaluable for breeding programs that could lead to developing varieties with improved oil content and cultivars optimized for specific growing environments. We identified several candidate genes associated with key agronomic traits, including OC, PH, DTF, OA, LA, 100SW, PB, and HN as detailed in Table 2. These candidate genes could also be used for genome editing approaches, although further validation is required. Similar approaches have been employed in apple [52], rice [53] and soyabean [54]. To increase the confidence in the identified candidate genes, we performed haplotype analysis. A similar approach has been employed for the detection of haplotypes associated with various agronomically important traits [55,56]. We detected robust haplotypes exhibiting significant variation in the core collection for OC, SW, DTF, OA, and LA. However, only one or two major haplotypes were identified for PH, PB, and HN, highlighting their complexity due to strong interactions with environmental conditions. We observed many SNPs (range from 11-40) within each haplotype. The number of haplotypes ranged from 11 to 83, with OC, DTF, SW, OA, and LA having more than 50 haplotypes, indicating presence of diverse SNP combinations present in germplasm. Interestingly, we found that putative candidate genes were in proximity (within 1 Kb) of the haplotypes, suggesting that these SNP groups may play crucial roles in gene function. In this study, we identified favourable haplotypes, which are prevalent in large populations and encoded a wide range of trait values and likely selected through evolution [57,58]. However, we also detected superior haplotypes, contributing to exceptional trait values associated with rare haplotypes which are of agronomic values. For superior OC, we identified three accessions—CC106, CC101, and CC090—characterized by high oil content coupled with superior alleles for OA or LA. However,

these accessions had favourable haplotypes for other traits. The information generated identified superior haplotypes through haplopheno analysis, but to create varieties with multiple superior haplotypes approaches like pyramid breeding are required [59]. Nonetheless, the information generated in current analysis is invaluable for safflower breeding programs.

Pan-genome analysis reveals distinct functional enrichments among pan-genes

A single reference genome cannot encompass the entire genetic variability present in a species like *C. tinctorius* which has gone through extensive diversification [60]. Pan-genomes developed from diverse individuals acts as an important resource to capture the available genetic variability and allele mining. In recent years, pan-genomes have been constructed for various plant species [60–63] . We constructed the pangenome of safflower using unmapped reads of 123 accessions of our core collection through iterative mapping and assembly approach. The newly assembled sequence includes those regions that were absent in the reference genome and thus, act as a complete repertoire of available genes for the crop. We further performed PAV analysis using the map-to-pan approach and identified core genes that are common across multiple genomes and define the species, as well as variable genes found in only a few genomes, which contribute to the unique characteristics of each genome. The enrichment of variable genes was mainly detected in the categories *viz*. regulation of biological process, response to stimulus, catalytic and binding as well as disease resistance. We further analysed core and variable genes among regional gene pools. The secondary regional gene pools of safflower, *i.e.*, USA, India, Far-east and Europe comprise accessions (Supplementary Table S25) with large numbers of variable genes and thus, exhibit high genetic diversity that might have arisen during diversification of safflower. In our study, the Indian accession, CC38, was found to be the most diverse accession of the core collection consisting of

large number of cloud and private genes. The pangenome study would facilitate use of agronomically important genes for regional breeding programs.

Methods

Plant material

For generating genome assembly, a safflower accession from USA (PI:560169; USDA, USA; named as “Safflower_A2”) attributed with substantially high seed oil content (~47%) and naturally enriched with high oleic acid (~87%) was selected. Safflower core collection reported earlier by our group [14] comprising 116 globally distributed accessions and 7 additional accessions (Supplementary table 17) with agronomically important traits were subjected to Illumina-based resequencing (~15x coverage) using Novaseq sequencer.

Genome Sequencing and assembly

HiFi long read sequencing was performed on PacBio Sequel II platform following manufacturer’s instructions (PacBio, California). A Bionano Saphyr chip (Bionano genomics, California, USA) was used for generating optical mapping data. The Proximo Hi-C (Plant) Kit Protocol (Phase genomics, USA) was used to construct Hi-C library (Phase genomics, USA). The Hi-C libraries were sequenced on Illumina Novaseq 6000 (Illumina, USA).

Genome size was estimated through two approaches: flow cytometry and k-mer frequency distribution. For flow cytometry, CyStain PI Absolute P kit (Sysmex, Germany) was used for sample preparation following manufacturer’s instructions. A minimum of 5,000 stained nuclei per

sample were evaluated on CyFlow Cube 8 flow cytometer (Sysmex, Germany) using tomato ‘Stupicke´ polnı´ rane´’ as a reference [64]. K-mer-based genome size estimation was performed using clean HiFi reads from PacBio SMRT sequencing applying Kmerfreq [65].

Long PacBio HiFi reads were assembled into contigs using Hifiasm v.0.16 [66]. Due to homozygous nature of safflower genome, purging was disabled (-I=0) and other parameters were applied at default settings. Using optical maps and contig-level assembly, hybrid scaffolding was performed using Bionano Solve v3.6 with default parameters. Scaffold-level assembly was polished using Illumina paired-end short reads through NextPolish [67]. For construction of pseudochromosomes, hybrid scaffolds were linked using paired-end Hi-C reads with SALSA [68] at default settings. Additional two rounds of polishing were done using Pilon [69]. Finally, Hi-C raw reads were aligned to pseudochromosomes through BWA2 [70] to generate Hi-C heatmap using PretextMap [71] and uploaded on Juicer [72] for visualisation, manual curation and generation of final chromosomal-level genome assembly.

The genome assembly was validated for integrity and completeness. Sequencing reads from Illumina and PacBio HiFi were mapped back to the assembly using BWA2 and minimap2 [73] respectively. For estimation of base-level accuracy, Merqury analysis [74] was performed. BUSCO v5.6.0 [75] analysis was implemented to assess completeness of the genome. LTR_retreiver v2.9.8 [76] was used for estimation of LAI score. Telomeres were detected using Telomere identification toolkit (TIDK) [77]. Centromeric repeats were detected using TRASH [78]. Chloroplast genome was generated by assembling Illumina short reads by Navoplasty [79] using NC_030783.1 [80] as a reference.

Construction of a high-density genetic linkage map and chromosomal assignment

A RIL population comprising 121 individuals (designated as "population A"; F_8) was developed by crossing parents A1 (PI:537110) and A2 (PI:560169). The GBS library was prepared following [81] using a combination of *Mse*I and *Hae*II enzymes. Libraries were sequenced on Illumina NovaSeq 6000 platform. Filtered reads were aligned to Safflower_A2 genome and SNPs were called (Supplementary Fig. 3b). SNPs were filtered using criteria summarized in Supplementary Table S7. A high-density linkage map was constructed via JoinMap using the Kosambi mapping function at LOD 20, followed by marker order correction and calculation of genetic distances using R/ASMap [82] at LOD 5. The linkage map was utilized for evaluating and anchoring of chromosomes using ALLMAPS [83].

Full-length transcriptome sequencing and detection of alternate splicing events

Iso-Seq sequencing was performed on RNA from eight samples [shoots, seedling-roots, leaves, flowers and buds, and seed developmental stages (at 5 DAPS, 10 DAP, 20 DAP and 30 DAP)]. Size-selected SMRTbell libraries were sequenced on PacBio Sequel II platform. Raw data was processed via SMRTlink v9.0 to generate circular consensus sequences (CCS) using default parameters. IsoSeq v3 pipeline [84] was used to obtain full-length transcripts which were collapsed into transcript clusters using pbcluster. pbmm2 [85] was used to map FL-transcripts to repeat-masked Safflower_A2 genome using parameters --preset ISOSEQ –sort. Splicing patterns of FL transcripts were analysed using SUPPA2 [86,87].

Annotation of repeatome, gene prediction and functional annotation of protein-coding genes

Safflower-specific *de novo* TE libraries were identified using Extensive De novo TE Annotator tool (EDTA v2.0.0) [88]. Non-Long TE were detected using RepeatModeler v2.0.5 [89]. Identified repeats were masked using RepeatMasker [90].

The classification of LTR retrotransposons (LTR-TEs) was carried out using the Domain-based Annotation of Transposable Elements (DANTE, version 0.2.5) pipeline [91]. This tool extracts information from the Viridiplantae data in the Rexdb database. Additionally, we employed DANTE_LTR to identify and classify LTR-TEs as complete/autonomous i.e. consisting of complete machinery for transposition. An LTR was classified as autonomous/complete if it contained all necessary domains, including reverse transcriptase (RT), capsid-related domain (GAG), RNase H (RH), protease (PROT), integrase (INT), target site duplications (TSD), and primer-binding site (PBS). For phylogenetic analysis, amino acid sequences of the identified complete Copia and Gypsy elements were extracted to generate a multiple sequence alignment using MAFFT. A phylogenetic tree was subsequently constructed with iqtree2. The insertion times of the complete LTRs were estimated using LTR_retriever[92].

The identification of noncoding RNAs, including ribosomal RNA (rRNA) and transfer RNA (tRNA), was performed in the safflower genome assembly. Predictions of rRNA genes were conducted using barrnap v0.9, applying specific parameters for eukaryotic genomes (--kingdom euk) [93]. For tRNA gene predictions, we utilized tRNAscan-SE v2.0 [94].

Masked genome was used for gene prediction using BRAKER3[37]. Mapping data generated by pbmm2 (as described above) was used as a training set for *ab initio* gene finders, AUGUSTUS [86] and GeneMark [95] for gene prediction. Transcripts <150 bases and protein sequences with <50 amino acids were removed. Subsequently, transcripts were filtered to remove those which showed a continuous repeat coverage of ≥30%. To identify the number of unigenes, CD-HIT

v4.8.1 [96] was employed using parameters -c 0.8 -n 5 -M 16000. BUSCO analysis was performed to assess completeness of filtered gene sets. Gene models were subjected to functional annotation using public nucleotide and protein databases in OmicsBox v3.1.2 [97]. Homology searches were conducted against the NCBI-RefSeq database using BLASTp with a threshold E-value of $1X10^{-3}$ against GO, EggNOG mapper v5 and KEGG expression database [98]. The motifs and domains-based functional annotation and identification of conserved domains and families in protein-coding genes was implemented using all public databases in InterProScan v5.6 [99]. Transcription factors/regulators and Protein Kinases were identified using iTAK v1.6 [100] with default parameters.

Identification of resistance gene analogs (RGAs)

RGAs were identified using Disease Resistance Analysis and Gene Orthology (DRAGO2) pipeline [101]. DRAGO2 classifies RGAs into Coiled-coil (CC), Receptor-like kinases (RLKs), Receptor-like proteins (RLPs), Nucleotide binding site-leucine rich repeats (NBS-LRR) and others. Based on domain structures, NBS-LRR were further classified into CNL and TNL. HMMER software was used to classify NBS-LRR genes using the NB-ARC profile (PF00931) for the NBS domain [102]. NBS-LRR sequences were retrieved from NCBI, and BLASTp was performed. Common candidate genes identified from three analyses (HMMER, DRAGO2 and BLASTp) were retained for downstream analysis. PFAM [103] and Conserved Domains Database (CDD) [104] were used for functional domain annotation. Chromosome-wide distribution of NBS LRR genes was investigated using GFF files. For inferring the evolutionary history of NBS family members, CLUSTALW [105] was performed, followed by the construction of a phylogenetic tree using IQ-TREE v2.0.6 [106] with maximum likelihood (ML) at 1000 bootstrap. To analyse RGAs under

selection pressure, non-synonymous substitution to synonymous substitution (Ka/Ks) ratio was calculated using KaKs calculator 2.0 [100]. Synteny analysis was performed using MCScanX [77] for the detection of duplicated genes.

Genome-wide identification of FAD, Oleosin, PAL and CHS gene families

Safflower_A2 genome was mined for genes associated with oil biosynthesis, composition and storage (FAD and Oleosins) and flavonoid biosynthetic pathway (PAL and CHS) using BLASTp and Hidden Markov Models (HMMs) with a 10^{-5} E value cut-off. The existence of characteristic domains was confirmed using CDD [104] and Pfam database [103]. Physiochemical properties were explored using ExPaSy ProtParam [107]. Multiple Expectation Maximization for Motif Elucidation (MEME v5.0.3) [79] was used to survey conserved motifs (maximum number of motifs 10). Subcellular localization was deduced using CELLO 2.5 [108]. Chromosomal positions of all genes were obtained from the genomic GFF file and visualised using TBTools toolkit [109]. To explore evolutionary relationships among gene families, multiple sequence alignment was conducted using MUSCLE within MEGA v11.0.10 [110] using default parameters. The resulting alignment was utilized to construct a phylogenetic tree with the ML at 1000 bootstrap replicates using IQ-TREE v2.0.6 [106].

Exploring genetic basis of the safflower for the various agronomically important traits

Genomic DNA was sequenced on Illumina NovaSeq 6000 platform (Illumina, USA) to generate 150 bp paired end reads. Reads were cleaned and mapped to Safflower_A2 genome using BWA-mem v0.7.17 [83]. GATK v4.4.0 [84] was used for variant calling with parameters: --minimum-

mapping-quality 20, --min-base-quality-score 20, and hard filtering was applied based on GATK best practices recommendations [111] followed by filtration using VCFtools v0.1.15 (Supplementary Table S18).

Fast Tree v2.1.10 [86] with GTR model was used for construction of the phylogenetic tree using filtered SNPs and visualized using Interactive Tree of Life (iTOL) v5 [87]. Genetic structure of the core collection was assessed using ADMIXTURE v1.3.0 [88]. Number of clusters (K) were inferred based on lowest CV error. PCA was performed using PLINK v1.90b4.6 [89] with default parameters. The first two eigenvectors showing maximum variability were plotted using R. Pairwise F_{ST} between subpopulations inferred from ADMIXTURE (K=4) were calculated using VCFtools [90]. PopLD decay [91] with default settings was used for estimation of LD in safflower.

Phenotypic data described earlier [14] for eight traits including PH, HN, PB, DTF, OC, 100SW, OA and LA content from two independent growing seasons (2011-2012 and 2012-2013) were used. SNPs generated in the present study were filtered using TASSEL v5 [112]. GWAS analysis was conducted for data of two seasons independently using two single locus models [General Linear Model (GLM) and Mixed Linear Model (MLM); [113] and three multi-locus models [Multi-Locus Mixed Model [114], Fixed and random model Circulating Probability Unification [(FarmCPU; [27], and Bayesian-information and Linkage-disequilibrium Iteratively Nested Keyway [BLINK; [115]. These models were implemented using GAPIT v3 [116] in R programming software. MTAs were considered significant at $p<0.0001$.

QTNs identified for eight traits were used for candidate gene analysis (CGA). LD block analysis was conducted using LDBlockShow [117] to define the region for CGA. Candidate genes were subsequently searched within a 7 kb region upstream and downstream of the QTNs. To verify the

association of candidate genes with traits, putative candidates were screened based on their annotated functions. Genes associated with metabolic pathways, stress responses, and traits such as oil biosynthesis, plant architecture, and flowering time were selected for Haplo-pheno analysis. The GenhapR [57] package was utilized to detect haplotypes within the LD regions of candidate genes. Association analysis was conducted for haplogroups represented in three or more individuals in the core collection, while other haplotypes were classified as rare. Haplotypes with high average values and present in a large number of accessions were designated as favourable haplotypes. The haplotypes found in accessions exhibiting the highest trait values were identified as superior haplotypes.

Pan-genome assembly, annotation, and PAV analysis

A pan-genome was constructed through an iterative mapping and assembly approach using sequencing data of core collection (Illumina short reads, 123 samples) and available chromosomal-level assemblies of safflower (Anhui1 and Chaunhangua 1). We constructed pan-genome assembly in four major steps: (1) The chromosomal-level assemblies of safflower (Anhui1 and Chaunhangua1) were iteratively mapped to the Safflower_A2_reference sequence using BWA-mem v0.7.17 and novel segments were integrated. (2) Filtered Illumina sequencing reads from the core collection were mapped to the new reference genome using BWA-mem v0.7.17. (3) Unmapped and discordant reads were extracted using SAMtools view v1.20 (-f4, -f8 and -f12) and assembled *de novo* using MaSurca v3.2.3 [118]. Contaminated reads in the resultant contigs were identified using BLASTn against the NCBI-NR and RefSeq databases and discarded. (4) All the sequences novel from the reference genome (Safflower_A2) were further assembled resulting in novel additional sequences. Repeat regions were identified using EDTA and soft masked genome

was subjected to Braker3 for gene prediction. Protein sequences of *Arabidopsis thaliana*, *Helianthus annus*, *Lactuca sativa, Cyanara cardunculus* and *Carthamus tinctorius* from Refseq and Swiss prot databases were used as a hint. Predicted genes were clustered using CD-HIT v4.8.1[119] and redundancy was removed. Genes intersecting with repeat regions (>30%) were removed using BEDtools intersect v2.21.0 [120]. The remaining genes were aligned to the Safflower_A2 genome followed by the removal of genes showing high similarity (perc_identity =0.8 and query_cov =0.8). The remaining genes were considered pan-genes and used for downstream analysis. Proteins encoded by the pan-genes were subjected to functional annotation using OmicsBox v3.1.2. Contigs from the above assembly were concatenated with the Safflower_A2 genome to construct a pan-genome (named Safpg_v1). PAV analysis was performed by aligning raw reads of genomes to genic sequences of Safpg_v1 using bowtie2 (--no-mixed, --local). Genes were considered present if 80% of the gene is covered by the reads with a minimum depth of 3, else marked as absent. Based on the presence of a gene in the accessions, it was assigned a category as core (≥97%), softcore (90-96%), shell (15-89%), cloud (<15%), or private (only in one accession). To check whether a pan-genome is closed or open, core genome size and pan-genome size were fitted using the nls function in R.

Data availability

Genome assembly, functional annotation, protein, transcript sequence files and pan-genome assembly are available at the Safflower Genome Resource (SGR) (http://13.60.187.179:3002/). The raw sequencing data and genome assembly generated during this study has been deposited at NCBI under the BioProject PRJNA1089929. Bionano optical mapping data is available at SGR

(http://13.60.187.179:3002/downloads/). Phenotypic data of the core collection used for the GWAS analysis is available at https://figshare.com/s/c8efdcf9b799bf669685.

Abbreviations

SNP: Single Nucleotide Polymorphism; GBS: Genotyping by Sequencing; RIL: Recombinant Inbred Lines; RGAs: Resistance Gene Analogs; SSRs: Simple Sequence Repeats; BUSCO: Benchmarking Universal Single Copy Orthologs; LG: Linkage Group; DAP: Days After Pollination; CCS: Circular Consensus Sequences; FLNC: Full-Length Non-Chimeric; AS: Alternative Splicing; RI: Retained Intron; SE: Skipping Exon; A5S: Alternative 5' Splice Site ; A3S: Alternative 3' Splice Site; AF: Alternative First (exon); AL: Alternative Last (exon); MX: Mutually Exclusive (exon); TE: Transposable Elements; LTR: Long Terminal Repeat; LINEs: Long Interspersed Nuclear Elements; SINEs: Short Interspersed Nuclear Elements; TIR: Tandem Inverted Repeats; MITES: Miniature Inverted Repeat Transposable Elements; CDS: Coding Sequence; GO: Gene Ontology; COG: Cluster of Orthologous Groups; KEGG: Kyoto Encyclopaedia of Genes and Genomes; NLR: Nucleotide-binding site-Leucine-rich repeat Receptor; TNL: Toll/interleukin-1 receptor- Nucleotide-binding site- Leucine-rich repeat; RNL: RPW8-NBS-LRR; ETI: Effector Triggered Immunity; FAD: Fatty Acid Desaturase; PAL: Phenylalanine Ammonium Lyase; CHS: Chalcone Synthase; GWAS: Genome-Wide Association Studies; LD: Linkage Decay; MTA: Marker Trait Association ; QTN: Quantitative Trait Nucleotide; OC: Oil Content; OA: Oleic Acid; LA: Linoleic Acid; 100SW: 100 Seed Weight; PH: Plant Height; HN: Head Number; PB: Primary Branches; DTF: Days To 50% Flowering; PAV: Presence Absence Variation; SGR: Safflower Genome Resource; RE: Repeat Elements; PCA: Principal Component Analysis; EMMA: Efficient Mixed-Model Association

Declarations

Ethics approval: Not applicable

Consent for publication: Not applicable

Competing interests: The authors declare no competing interests.

Funding

This work was supported by Department of Biotechnology, Government of India grants to AJ and SG (BT/Ag/Network/Safflower/2019-20; Sub Projects 3 and 4).

Author contributions

MS and VB carried out all field and laboratory experiments; SG, AJ, MS and VB contributed to writing the manuscript; SG and AJ conceptualized, supervised the overall study and secured funding; MS, VB, PKO, HA, SG and RNS planned and performed bioinformatic analysis; VB, MS, AJ, SG and VJ planned and performed mapping studies.

Acknowledgements

MS, VB, PKO acknowledge junior and senior research fellowships provided by the Council of Scientific and Industrial Research, Ministry of Science and Technology, Government of India.

References

1. Fernandez-Martinez J, Del Rio M, De Haro A. Survey of safflower (Carthamus tinctorius L.) germplasm for variants in fatty acid composition and other seed characters. *Euphytica*. Springer; 69:115–221993;

2. Khalid N, Khan RS, Hussain MI, Farooq M, Ahmad A, Ahmed I. A comprehensive characterisation of safflower oil for its potential applications as a bioactive food ingredient - A review. *Trends Food Sci Technol*. 2017; doi: 10.1016/j.tifs.2017.06.009.

3. Sharma M, Bhardwaj V, Goswami P, Kalra A, Palchamy K, Jagannath A, et al.. Increasing Nutraceutical and Pharmaceutical Applications of Safflower: Genetic and Genomic Approaches. In: Kole C, editor. *Compend Crop Genome Des Nutraceuticals*. Singapore: Springer Nature Singapore;

4. : FAOSTAT. https://www.fao.org/faostat/en/#data/QCL (2024). Accessed 2024 Jan 13.

5. : Safflower Oil Market. https://www.futuremarketinsights.com/reports/safflower-oil-market Accessed 2023 May 26.

6. Weiss EA. Oilseed crops. Blackwell Science;

7. Ashri A. Evaluation of the germ plasm collection of safflower, Carthamus tinctorius LV Distribution and regional divergence for morphological characters. *Euphytica*. Springer; 24:651–91975;

8. Yang Y-X, Wu W, Zheng Y-L, Chen L, Liu R-J, Huang C-Y. Genetic diversity and relationships among safflower (Carthamus tinctorius L.) analyzed by inter-simple sequence repeats (ISSRs). *Genet Resour Crop Evol*. Springer; 54:1043–512007;

9. Ambreen H, Kumar S, Variath MT, Joshi G, Bali S, Agarwal M, et al.. Development of genomic microsatellite markers in Carthamus tinctorius L.(safflower) using next generation sequencing and assessment of their cross-species transferability and utility for diversity analysis. *PloS One*. Public Library of Science San Francisco, CA USA; 10:e01354432015;

10. Kumar S, Ambreen H, Murali TV, Bali S, Agarwal M, Kumar A, et al.. Assessment of genetic diversity and population structure in a global reference collection of 531 accessions of Carthamus tinctorius L.(Safflower) using AFLP markers. *Plant Mol Biol Report*. Springer; 33:1299–3132015;

11. Chapman MA, Burke JM. DNA sequence diversity and the origin of cultivated safflower (Carthamus tinctorius L.; Asteraceae). *BMC Plant Biol*. 2007; doi: 10.1186/1471-2229-7-60.

12. Wu Z, Liu H, Zhan W, Yu Z, Qin E, Liu S, et al.. The chromosome-scale reference genome of safflower (Carthamus tinctorius) provides insights into linoleic acid and flavonoid biosynthesis. *Plant Biotechnol J*. Association of Applied Biologists; :1–18 2021;

13. Chen J, Guo S, Hu X, Wang R, Jia D, Li Q, et al.. Whole-genome and genome-wide association studies improve key agricultural traits of safflower for industrial and medicinal use. *Hortic Res*. Oxford University Press; 10:uhad1972023;

14. Kumar S, Ambreen H, Variath MT, Rao AR, Agarwal M, Kumar A, et al.. Utilization of molecular, phenotypic, and geographical diversity to develop compact composite core collection in the oilseed crop, safflower (Carthamus tinctorius L.) through maximization strategy. *Front Plant Sci*. Frontiers Media SA; 7:15542016;

15. Lu C, Shen Q, Yang J, Wang B, Song C. The complete chloroplast genome sequence of Safflower (*Carthamus tinctorius* L.). *Mitochondrial DNA Part A*. 2016; doi: 10.3109/19401736.2015.1018217.

16. Ventimiglia M, Castellacci M, Usai G, Vangelisti A, Simoni S, Natali L, et al.. Discovering the Repeatome of Five Species Belonging to the Asteraceae Family: A Computational Study. *Plants*. MDPI; 12:14052023;

17. Kirov I, Omarov M, Merkulov P, Dudnikov M, Gvaramiya S, Kolganova E, et al.. Genomic and transcriptomic survey provides new insight into the organization and transposition activity of highly expressed LTR retrotransposons of sunflower (Helianthus annuus l.). *Int J Mol Sci*. MDPI; 21:93312020;

18. Masand M, Sharma S, Kumari S, Pal P, Majeed A, Singh G, et al.. High-quality haplotype-resolved chromosome assembly provides evolutionary insights and targeted steviol glycosides (SGs) biosynthesis in *Stevia rebaudiana* Bertoni. *Plant Biotechnol J*. 2024; doi: 10.1111/pbi.14446.

19. Mascagni F, Barghini E, Giordani T, Rieseberg LH, Cavallini A, Natali L. Repetitive DNA and plant domestication: variation in copy number and proximity to genes of LTR-retrotransposons among wild and cultivated sunflower (Helianthus annuus) genotypes. *Genome Biol Evol*. Oxford University Press; 7:3368–822015;

20. Kourelis J, Van Der Hoorn RA. Defended to the nines: 25 years of resistance gene cloning identifies nine mechanisms for R protein function. *Plant Cell*. American Society of Plant Biologists; 30:285–992018;

21. Mun J-H, Yu H-J, Park S, Park B-S. Genome-wide identification of NBS-encoding resistance genes in Brassica rapa. *Mol Genet Genomics*. 2009; doi: 10.1007/s00438-009-0492-0.

22. Filippi CV, Merino GA, Montecchia JF, Aguirre NC, Rivarola M, Naamati G, et al.. Genetic diversity, population structure and linkage disequilibrium assessment among international sunflower breeding collections. *Genes*. MDPI; 11:2832020;

23. Liu H, Zhou H, Wu Y, Li X, Zhao J, Zuo T, et al.. The impact of genetic relationship and linkage disequilibrium on genomic selection. *PloS One*. Public Library of Science San Francisco, CA USA; 10:e01323792015;

24. Li F, Chen B, Xu KUN, Wu J, Song W, Bancroft IAN, et al.. Genome-wide association study dissects the genetic architecture of seed weight and seed quality in rapeseed (Brassica napus L.). *DNA Res*. Oxford University Press; 21:355–672014;

25. Jameson PE, Song J. Cytokinin: a key driver of seed yield. *J Exp Bot*. Oxford University Press; 67:593–6062016;

26. Madson M, Dunand C, Li X, Verma R, Vanzin GF, Caplan J, et al.. The MUR3 gene of Arabidopsis encodes a xyloglucan galactosyltransferase that is evolutionarily related to animal exostosins. *Plant Cell*. American Society of Plant Biologists; 15:1662–702003;

27. Huang AH. Plant lipid droplets and their associated proteins: potential for rapid advances. *Plant Physiol*. American Society of Plant Biologists; 176:1894–9182018;

28. Lou L, Ding L, Wang T, Xiang Y. Emerging roles of RNA-binding proteins in seed development and performance. *Int J Mol Sci*. MDPI; 21:68222020;

29. Sauveplane V, Kandel S, Kastner P, Ehlting J, Compagnon V, Werck-Reichhart D, et al.. *Arabidopsis thaliana* CYP77A4 is the first cytochrome P450 able to catalyze the epoxidation of free fatty acids in plants. *FEBS J*. 2009; doi: 10.1111/j.1742-4658.2008.06819.x.

30. Xiang F, Liu W, Liu X, Song Y, Zhang Y, Zhu X, et al.. Direct balancing of lipid mobilization and reactive oxygen species production by the epoxidation of fatty acid catalyzed by a cytochrome P450 protein during seed germination. *New Phytol*. 2023; doi: 10.1111/nph.18669.

31. Jang G, Kim J, Yu J-K, Kim H-J, Kim Y, Kim D-W, et al.. Cost-effective unmanned aerial vehicle (UAV) platform for field plant breeding application. *Remote Sens*. MDPI; 12:9982020;

32. Li S-W, Shi R-F, Leng Y, Zhou Y. Transcriptomic analysis reveals the gene expression profile that specifically responds to IBA during adventitious rooting in mung bean seedlings. *BMC Genomics*. 2016; doi: 10.1186/s12864-016-2372-4.

33. Rosquete MR, Worden N, Ren G, Sinclair RM, Pfleger S, Salemi M, et al.. AtTRAPPC11/ROG2: a role for TRAPPs in maintenance of the plant trans-Golgi network/early endosome organization and function. *Plant Cell*. American Society of Plant Biologists; 31:1879–982019;

34. Shigeyama T, Watanabe A, Tokuchi K, Toh S, Sakurai N, Shibuya N, et al.. α-Xylosidase plays essential roles in xyloglucan remodelling, maintenance of cell wall integrity, and seed germination in Arabidopsis thaliana. *J Exp Bot*. Oxford University Press UK; 67:5615–292016;

35. Bayer PE, Golicz AA, Scheben A, Batley J, Edwards D. Plant pan-genomes are the new reference. *Nat Plants*. Nature Publishing Group UK London; 6:914–202020;

36. Hare EE, Johnston JS. Genome Size Determination Using Flow Cytometry of Propidium Iodide-Stained Nuclei. In: Orgogozo V, Rockman MV, editors. *Mol Methods Evol Genet*. Totowa, NJ: Humana Press;

37. Gabriel L, Brůna T, Hoff KJ, Ebel M, Lomsadze A, Borodovsky M, et al.. BRAKER3: Fully automated genome annotation using RNA-Seq and protein evidence with GeneMark-ETP, AUGUSTUS and TSEBRA. *BioRxiv Prepr Serv Biol*. 2023; doi: 10.1101/2023.06.10.544449.

38. Jiang S, An H, Xu F, Zhang X. Chromosome-level genome assembly and annotation of the loquat (Eriobotrya japonica) genome. *GigaScience*. Oxford University Press; 9:giaa0152020;

39. Wang J, Wang X, Ma Y, Gao R, Wang Y, An Z, et al.. Lonicera caerulea genome reveals molecular mechanisms of freezing tolerance and anthocyanin biosynthesis. *J Adv Res*. Elsevier; 2024;

40. Xue J-Y, Takken FL, Nepal MP, Maekawa T, Shao Z-Q. Evolution and functional mechanisms of plant disease resistance. *Front Genet*. Frontiers; 11:5932402020;

41. Monosi B, Wisser RJ, Pennill L, Hulbert SH. Full-genome analysis of resistance gene homologues in rice. *Theor Appl Genet*. Springer; 109:1434–472004;

42. Bai L, Zhou P, Li D, Ju X. Changes in the gastrointestinal microbiota of children with acute lymphoblastic leukaemia and its association with antibiotics in the short term. *J Med Microbiol*. Microbiology Society; 66:1297–3072017;

43. Neupane S, Andersen EJ, Neupane A, Nepal MP. Genome-wide identification of NBS-encoding resistance genes in sunflower (Helianthus annuus L.). *Genes*. MDPI; 9:3842018;

44. Cloutier M, Xiang D, Gao P, Kochian LV, Zou J, Datla R, et al.. Integrative Modeling of Gene Expression and Metabolic Networks of Arabidopsis Embryos for Identification of Seed Oil Causal Genes. *Front Plant Sci*. 122021;

45. Cannon SB, Zhu H, Baumgarten AM, Spangler R, May G, Cook DR, et al.. Diversity, distribution, and ancient taxonomic relationships within the TIR and non-TIR NBS-LRR resistance gene subfamilies. *J Mol Evol*. Springer; 54:548–622002;

46. Shao Z-Q, Zhang Y-M, Hang Y-Y, Xue J-Y, Zhou G-C, Wu P, et al.. Long-term evolution of nucleotide-binding site-leucine-rich repeat genes: understanding gained from and beyond the legume family. *Plant Physiol*. American Society of Plant Biologists; 166:217–342014;

47. Shao Z-Q, Xue J-Y, Wu P, Zhang Y-M, Wu Y, Hang Y-Y, et al.. Large-scale analyses of angiosperm nucleotide-binding site-leucine-rich repeat genes reveal three anciently diverged classes with distinct evolutionary patterns. *Plant Physiol*. American Society of Plant Biologists; 170:2095–1092016;

48. Chen K, Yin Y, Liu S, Guo Z, Zhang K, Liang Y, et al.. Genome-wide identification and functional analysis of oleosin genes in Brassica napus L. *BMC Plant Biol*. 2019; doi: 10.1186/s12870-019-1891-y.

49. Mosupiemang M, Malambane G, Emongor V, Mathapa B. Oleosin Expression Patterns and Size of Oil Bodies as a Factor in Determining Oil Content in Safflower (Carthamus Tinctorius L.) Genotypes. *Eur J Agric Food Sci*. 2022; doi: 10.24018/ejfood.2022.4.5.570.

50. Ambreen H, Kumar S, Kumar A, Agarwal M, Jagannath A, Goel S. Association mapping for important agronomic traits in safflower (Carthamus tinctorius L.) core collection using microsatellite markers. *Front Plant Sci*. Frontiers Media SA; 9:4022018;

51. Khan SU, Saeed S, Khan MHU, Fan C, Ahmar S, Arriagada O, et al.. Advances and challenges for QTL analysis and GWAS in the plant-breeding of high-yielding: a focus on rapeseed. *Biomolecules*. MDPI; 11:15162021;

52. Dujak C, Coleto-Alcudia V, Aranzana MJ. Genomic analysis of fruit size and shape traits in apple: unveiling candidate genes through GWAS analysis. *Hortic Res*. Oxford University Press; 11:uhad2702024;

53. Bukomarhe CB, Kimwemwe PK, Githiri SM, Mamati EG, Kimani W, Mutai C, et al.. Association mapping of candidate genes associated with iron and zinc content in rice (Oryza sativa L.) grains. *Genes*. MDPI; 14:18152023;

54. Jia Q, Hu S, Li X, Wei L, Wang Q, Zhang W, et al.. Identification of candidate genes and development of KASP markers for soybean shade-tolerance using GWAS. *Front Plant Sci*. Frontiers Media SA; 15:14795362024;

55. Bhat JA, Karikari B, Adeboye KA, Ganie SA, Barmukh R, Hu D, et al.. Identification of superior haplotypes in a diverse natural population for breeding desirable plant height in soybean. *Theor Appl Genet*. 2022; doi: 10.1007/s00122-022-04120-0.

56. Wang X, Pang Y, Zhang J, Wu Z, Chen K, Ali J, et al.. Genome-wide and gene-based association mapping for rice eating and cooking characteristics and protein content. *Sci Rep*. Nature Publishing Group UK London; 7:172032017;

57. Zhang R, Jia G, Diao X. geneHapR: an R package for gene haplotypic statistics and visualization. *BMC Bioinformatics*. 2023; doi: 10.1186/s12859-023-05318-9.

58. Bhat JA, Yu D, Bohra A, Ganie SA, Varshney RK. Features and applications of haplotypes in crop breeding. *Commun Biol*. Nature Publishing Group UK London; 4:12662021;

59. Dormatey R, Sun C, Ali K, Coulter JA, Bi Z, Bai J. Gene pyramiding for sustainable crop improvement against biotic and abiotic stresses. *Agronomy*. MDPI; 10:12552020;

60. Golicz AA, Bayer PE, Barker GC, Edger PP, Kim H, Martinez PA, et al.. The pangenome of an agronomically important crop plant Brassica oleracea. *Nat Commun*. Nature Publishing Group UK London; 7:133902016;

61. Montenegro JD, Golicz AA, Bayer PE, Hurgobin B, Lee H, Chan CK, et al.. The pangenome of hexaploid bread wheat. *Plant J*. 2017; doi: 10.1111/tpj.13515.

62. Qin P, Lu H, Du H, Wang H, Chen W, Chen Z, et al.. Pan-genome analysis of 33 genetically diverse rice accessions reveals hidden genomic variations. *Cell*. Elsevier; 184:3542–582021;

63. Bayer PE, Petereit J, Durant É, Monat C, Rouard M, Hu H, et al.. Wheat Panache: A pangenome graph database representing presence–absence variation across sixteen bread wheat genomes. *Plant Genome*. 2022; doi: 10.1002/tpg2.20221.

64. Doležel J, Greilhuber J, Suda J. Estimation of nuclear DNA content in plants using flow cytometry. *Nat Protoc*. Nature Publishing Group; 2007; doi: 10.1038/nprot.2007.310.

65. Wang H, Liu B, Zhang Y, Jiang F, Ren Y, Yin L, et al.. Estimation of genome size using k-mer frequencies from corrected long reads. *ArXiv Prepr ArXiv200311817*. 2020;

66. Cheng H, Concepcion GT, Feng X, Zhang H, Li H. Haplotype-resolved de novo assembly using phased assembly graphs with hifiasm. *Nat Methods*. Nature Publishing Group US New York; 18:170–52021;

67. Hu J, Fan J, Sun Z, Liu S. NextPolish: a fast and efficient genome polishing tool for long-read assembly. *Bioinformatics*. Oxford University Press; 36:2253–52020;

68. Ghurye J, Pop M, Koren S, Bickhart D, Chin C-S. Scaffolding of long read assemblies using long range contact information. *BMC Genomics*. 2017; doi: 10.1186/s12864-017-3879-z.

69. Walker BJ, Abeel T, Shea T, Priest M, Abouelliel A, Sakthikumar S, et al.. Pilon: an integrated tool for comprehensive microbial variant detection and genome assembly improvement. *PloS One*. Public Library of Science San Francisco, USA; 9:e1129632014;

70. Vasimuddin M, Misra S, Li H, Aluru S. Efficient architecture-aware acceleration of BWA-MEM for multicore systems. *2019 IEEE Int Parallel Distrib Process Symp IPDPS*. IEEE; p. 314–24.

71. . sanger-tol/PretextMap. Tree of Life programme;

72. Durand NC, Shamim MS, Machol I, Rao SS, Huntley MH, Lander ES, et al.. Juicer provides a one-click system for analyzing loop-resolution Hi-C experiments. *Cell Syst*. Elsevier; 3:95–82016;

73. Li H. Minimap2: pairwise alignment for nucleotide sequences. *Bioinformatics*. Oxford University Press; 34:3094–1002018;

74. Rhie A, Walenz BP, Koren S, Phillippy AM. Merqury: reference-free quality, completeness, and phasing assessment for genome assemblies. *Genome Biol*. 2020; doi: 10.1186/s13059-020-02134-9.

75. Simão FA, Waterhouse RM, Ioannidis P, Kriventseva EV, Zdobnov EM. BUSCO: assessing genome assembly and annotation completeness with single-copy orthologs. *Bioinformatics*. Oxford University Press; 31:3210–22015;

76. Ou S, Chen J, Jiang N. Assessing genome assembly quality using the LTR Assembly Index (LAI). *Nucleic Acids Res*. Oxford University Press; 46:e126–e1262018;

77. . tolkit/telomeric-identifier. tolkit;

78. Wlodzimierz P, Hong M, Henderson IR. TRASH: tandem repeat annotation and structural hierarchy. *Bioinformatics*. Oxford University Press; 39:btad3082023;

79. Dierckxsens N, Mardulyn P, Smits G. NOVOPlasty: de novo assembly of organelle genomes from whole genome data. *Nucleic Acids Res*. Oxford University Press; 45:e18–e182017;

80. Liu G, Zhao Y, Gowda M, Longin CFH, Reif JC, Mette MF. Predicting hybrid performances for quality traits through genomic-assisted approaches in Central European wheat. *PLoS One*. Public Library of Science San Francisco, CA USA; 11:e01586352016;

81. Elshire RJ, Glaubitz JC, Sun Q, Poland JA, Kawamoto K, Buckler ES, et al.. A robust, simple genotyping-by-sequencing (GBS) approach for high diversity species. *PloS One*. Public Library of Science San Francisco, USA; 6:e193792011;

82. Taylor J, Butler D. R Package ASMap: Efficient Genetic Linkage Map Construction and Diagnosis. *J Stat Softw*. 2017; doi: 10.18637/jss.v079.i06.

83. Tang H, Zhang X, Miao C, Zhang J, Ming R, Schnable JC, et al.. ALLMAPS: robust scaffold ordering based on multiple maps. *Genome Biol*. 2015; doi: 10.1186/s13059-014-0573-1.

84. : Iso-Seq Home. Iso-Seq Docs. https://isoseq.how/ Accessed 2024 Jul 13.

85. . PacificBiosciences/pbmm2. PacBio;

86. Stanke M, Keller O, Gunduz I, Hayes A, Waack S, Morgenstern B. AUGUSTUS: ab initio prediction of alternative transcripts. *Nucleic Acids Res*. 2006; doi: 10.1093/nar/gkl200.

87. Trincado JL, Entizne JC, Hysenaj G, Singh B, Skalic M, Elliott DJ, et al.. SUPPA2: fast, accurate, and uncertainty-aware differential splicing analysis across multiple conditions. *Genome Biol*. 2018; doi: 10.1186/s13059-018-1417-1.

88. Ou S, Su W, Liao Y, Chougule K, Agda JR, Hellinga AJ, et al.. Benchmarking transposable element annotation methods for creation of a streamlined, comprehensive pipeline. *Genome Biol*. BioMed Central; 20:1–182019;

89. Smit AFA, Hubley R. RepeatModeler Open-1.0 http://www. repeatmasker. org. RepeatModeler;

90. Chen N. Using RepeatMasker to Identify Repetitive Elements in Genomic Sequences. *Curr Protoc Bioinforma*. 2004; doi: 10.1002/0471250953.bi0410s05.

91. Novák P, Hoštáková N, Neumann P, Macas J. DANTE and DANTE_LTR: Lineage-centric annotation pipelines for long terminal repeat retrotransposons in plant genomes. *bioRxiv*. Cold Spring Harbor Laboratory; :2024–04 2024;

92. . LTR_Retriever.

93. . Barrnap.

94. Chan PP, Lin BY, Mak AJ, Lowe TM. tRNAscan-SE 2.0: improved detection and functional classification of transfer RNA genes. *Nucleic Acids Res*. Oxford University Press; 49:9077–962021;

95. Besemer J, Borodovsky M. GeneMark: web software for gene finding in prokaryotes, eukaryotes and viruses. *Nucleic Acids Res*. 2005; doi: 10.1093/nar/gki487.

96. Fu L, Niu B, Zhu Z, Wu S, Li W. CD-HIT: accelerated for clustering the next-generation sequencing data. *Bioinformatics*. Oxford University Press; 28:3150–22012;

97. : Bioinformatics Software OmicsBox | Biobam. https://www.biobam.com/omicsbox/ Accessed 2024 Jul 13.

98. : KEGG: Kyoto Encyclopedia of Genes and Genomes. https://www.genome.jp/kegg/ Accessed 2024 Jul 13.

99. Quevillon E, Silventoinen V, Pillai S, Harte N, Mulder N, Apweiler R, et al.. InterProScan: protein domains identifier. *Nucleic Acids Res*. 2005; doi: 10.1093/nar/gki442.

100. Zheng Y, Jiao C, Sun H, Rosli HG, Pombo MA, Zhang P, et al.. iTAK: A Program for Genome-wide Prediction and Classification of Plant Transcription Factors, Transcriptional Regulators, and Protein Kinases. *Mol Plant*. 2016; doi: 10.1016/j.molp.2016.09.014.

101. Calle García J, Guadagno A, Paytuvi-Gallart A, Saera-Vila A, Amoroso CG, D'Esposito D, et al.. PRGdb 4.0: an updated database dedicated to genes involved in plant disease resistance process. *Nucleic Acids Res*. Oxford University Press; 50:D1483–902022;

102. Delorenzi M, Speed T. An HMM model for coiled-coil domains and a comparison with PSSM-based predictions. *Bioinformatics*. Oxford University Press; 18:617–252002;

103. Mistry J, Chuguransky S, Williams L, Qureshi M, Salazar GA, Sonnhammer ELL, et al.. Pfam: The protein families database in 2021. *Nucleic Acids Res*. 2021; doi: 10.1093/nar/gkaa913.

104. : NCBI Conserved Domain Search. https://www.ncbi.nlm.nih.gov/Structure/cdd/wrpsb.cgi Accessed 2024 Jul 13.

105. : Multiple Sequence Alignment - CLUSTALW. https://www.genome.jp/tools-bin/clustalw Accessed 2024 Jul 13.

106. Minh BQ, Schmidt HA, Chernomor O, Schrempf D, Woodhams MD, Von Haeseler A, et al.. IQ-TREE 2: new models and efficient methods for phylogenetic inference in the genomic era. *Mol Biol Evol*. Oxford University Press; 37:1530–42020;

107. : Expasy - ProtParam. https://web.expasy.org/protparam/ Accessed 2024 Jul 13.

108. : CELLO:Subcellular Localization Predictive System. http://cello.life.nctu.edu.tw/ Accessed 2024 Jul 13.

109. : Releases · CJ-Chen/TBtools-II. GitHub. https://github.com/CJ-Chen/TBtools-II/releases Accessed 2024 Jul 13.

110. Peterson KTDPN, Kumar GSMNS. MEGA5: molecular evolutionary genetics analysis using maximum likelihood, evolutionary distance, and maximum parsimony methods. *Mol Biol Evol*. 28:2731–92011;

111. Grzybowski MW, Mural RV, Xu G, Turkus J, Yang J, Schnable JC. A common resequencing-based genetic marker data set for global maize diversity. *Plant J*. 2023; doi: 10.1111/tpj.16123.

112. Bradbury PJ, Zhang Z, Kroon DE, Casstevens TM, Ramdoss Y, Buckler ES. TASSEL: software for association mapping of complex traits in diverse samples. *Bioinformatics*. 2007; doi: 10.1093/bioinformatics/btm308.

113. Zhang Z, Ersoz E, Lai C-Q, Todhunter RJ, Tiwari HK, Gore MA, et al.. Mixed linear model approach adapted for genome-wide association studies. *Nat Genet*. 2010; doi: 10.1038/ng.546.

114. Segura V, Vilhjálmsson BJ, Platt A, Korte A, Seren Ü, Long Q, et al.. An efficient multi-locus mixed-model approach for genome-wide association studies in structured populations. *Nat Genet*. Nature Publishing Group US New York; 44:825–302012;

115. Huang M, Liu X, Zhou Y, Summers RM, Zhang Z. BLINK: a package for the next level of genome-wide association studies with both individuals and markers in the millions. *GigaScience*. 2019; doi: 10.1093/gigascience/giy154.

116. Wang J, Zhang Z. GAPIT Version 3: Boosting Power and Accuracy for Genomic Association and Prediction. *Genomics Proteomics Bioinformatics*. 2021; doi: 10.1016/j.gpb.2021.08.005.

117. Dong S-S, He W-M, Ji J-J, Zhang C, Guo Y, Yang T-L. LDBlockShow: a fast and convenient tool for visualizing linkage disequilibrium and haplotype blocks based on variant call format files. *Brief Bioinform*. Oxford University Press; 22:bbaa2272021;

118. Zimin AV, Marçais G, Puiu D, Roberts M, Salzberg SL, Yorke JA. The MaSuRCA genome assembler. *Bioinformatics*. Oxford University Press; 29:2669–772013;

119. Li W, Godzik A. Cd-hit: a fast program for clustering and comparing large sets of protein or nucleotide sequences. *Bioinformatics*. Oxford University Press; 22:1658–92006;

120. Quinlan AR, Hall IM. BEDTools: a flexible suite of utilities for comparing genomic features. *Bioinformatics*. Oxford University Press; 26:841–22010;

Figure legends

Fig. 1: Overview of the Safflower_A2 genome (a) The outermost layer of the Circos represents the twelve assembled chromosomes; (b) Repetitive elements density; (c) Gene density; (d) Distribution of simple sequence repeats within the genome; (e) Distribution of single nucleotide polymorphisms (SNPs) across the chromosomes; (f) GC content of the safflower genome.

Fig. 2: Overview of annotation of protein-coding genes for Safflower_A2 genome assembly. (a) Functional annotation of protein coding genes using RefSeq, Cluster of Orthologous Genes (COG), Gene Ontology (GO), Kyoto Encyclopaedia of Genes and Genomes (KEGG) and Enzyme Code (EC) databases (b) Frequency histogram showing the distribution of top 20 GO terms across 3 categories: Biological Process, Cellular Component and Molecular Function (c) Top 20 members of Transcription Factors (TFs), Transcription Regulators (TRs) and Protein Kinases (PKs) identified in the Safflower_A2 genome (d) Distribution of COG categories across protein-coding genes.

Fig. 3: Overview of R genes in the Safflower_A2 genome. (a) Different domains present in R genes of safflower (b) Composition of R genes based on the number of domains (c) Phylogenetic analysis of NLR genes (d) Distribution of the NLR genes on safflower chromosomes (e) Ka/Ks analysis of NLR genes (f) Intra species synteny analysis of NLR genes. (f) Intra species synteny analysis of NLR genes.

Fig. 4: Diversity analysis and Genome Wide Association study of Safflower core collection. (a) Admixture plot showing four subpopulations ADI (Red), ADII (Green), AD III (Cyan), AD IV (Purple) (b) Phylogenetic tree showing evolutionary analysis (c) Marker density plot for 320,399

SNPs used in GWAS analysis for chromosomes 1 to 12, colours indicate marker density per Mb (d) Ideogram of QTNs detected from GWAS. QTNs for traits oil content, oleic and linoleic acid, hundred seed weight, plant height, head number, primary branches and days to 50% flowering detected across two growing seasons (2011-12 and 2012-13) are shown according to their respective chromosomal (labelled 1-12) positions.

Fig. 5: Exploration of the genetic basis of oil content in the safflower: (a) QQ and Manhattan plots for oil content representing all the multi-locus models. (b) LD-Block of 7 kb depicting correlation between the SNPs for the oil content (c) Haploblocks comprising the QTN OC1, OC8, and SNPs of gene BIG GRAIN (d) Haplo-network representing network of the haplotypes (e) Correlation of the favourable haplotypes with phenotypic data for season 1 (OC_1: 2011-12) and (g) season 2 (OC_2: 2012-13).

Fig. 6: The pan-genome of safflower. (a) Schematic diagram for the construction of pangenome (b) PAV matrix showing the presence (green) and absence (pink) of pan-genes in the different accessions. (c) Classification of pan-genes based on PAV analysis (d) Modelling of the pan-genome depicting core and pan genes (e) Distribution of distinct pan-genes amongst core collection accessions

 Click here to access/download;Table;gigacience tables.docx

Table 1: Comparison of Safflower_A2 genome with earlier published genome assemblies of Safflower

Genome feature	**Safflower_A2**	**Anhui_1**	**Chuanhonghua 1**
**Assembly statistics**			
Genome size (k-mer)	1.17	1.17	1.17
Contigs	2427	368	3941
Length of primary assembly (Mb)	1154	1070	1171
N50 (Mb)	8.9	21.3	1.071
Pseudochromosomes	12	12	12
Length of final assembly (Gb)	1.09	1.05	1.174
N50 (Mb)	88.4	88.2	96.3
Longest scaffold (Mb)	111	106.7	185
Unplaced contigs	1684	240	509
Size of remaining contigs (Mb)	66.3	Not available	Not available
**Quality assessment**			
BUSCO	97.90%	90.70%	89.79%
Mapping proportion (Long reads)	99.29%	98.10%	93.36%
**Annotation**			
Repetitive elements	71.30%	60.13%	71.41%
Number of transcripts	59,995	45,331	Not available
BUSCO	91.5 %	86.20 %	71.70 %
Average exons per gene	4.024	6.54	5.92
Mean exon length (bp)	265.84	269.59	235.66
Mean CDS length (bp)	1215	1265.89	Not available

Table 2: Table representing Quantative trait nucleotides (QTNs) marking candidate genes and associated haplotypes for various important agronomical traits in safflower.

Trait	QTN	Chromosome	Position	Alleles	Candidate gene and its annotation	Numb-er of Haplotypes	Number of SNPs in Haploblock	Number of accessions exhibiting the haplotypes	Favourable haplotype(s)	Haplotype location w.r.t. gene	Distance from gene (bp)	Superior Haplotypes
**100 seed weight**	SW10	3	74251721	G,A	g35324; RNA-binding protein 2-like isoform X2	60	40	112	H01(53)	Downstream	67	H19, H21
	SW23	3	74075377	C,T	g35305; Peptidyl-prolyl cis-trans isomerase CYP57 isoform X1	50	17	118	H01(57), H02 (4) ,H03 (3),H04(3)	Downstream	10	H01, H31, H02
	SW3	3	72485086	A,G	g35324 ; Xyloglucan galactosyltransferase XLT2	48	32	115	H01(61), H02 (6)	Gene within Haplotype	0	H24, H08, H30
	SW31	5	9066250	G,A	g42789; Oleosin-B6-like	28	13	122	H01(94)	Gene within Haplotype	0	H21, H2
	SW37	7	81516758	T,A	g57921; Protein FRIGIDA-ESSENTIAL 1-like isoform	30	16	122	HA01(93)	Downstream	199	

**Days to 50% Flowering**	DTF7	3	54974331	C,T	g34050; Exocyst complex component SEC5A-like isoform X2	74	26	113	H01(39), H02 (2)	Gene within Haplotype	0	H031, H029, H30
	DTF10	7	86666550	G,T	g58272 ; E3 ubiquitin-protein ligase COP1-like	80	13	115	H01(29), H02 (3), H03 (3)	Upstream	605	H58, H53, H80
	DTF2	11	7169035	C,T	g12806 ; E3 ubiquitin-protein ligase UPL1-like	13	6	123	H01(89), H02 (7),H03 (6),H04(5),H05(4),H06(3)	Downstream	4954	H01, H05, H06
	DTF2	11	7169035	C,T	g12807; 40S ribosomal protein S8	13	6	123	H01(89), H02 (7), H03 (6),H04(5),H05(4),H06(3)	Gene within Haplotype	0	H01, H05, H06
**Number of Heads**	HN5	4	5068529	G,A	g37215; Alpha-xylosidase 1-like	40	23	115	H01(76)	Upstream	530	H01, H0024
**Oleic and Linoleic Acid**	OA-LA15	5	18156971	C,G	g43426; Cytochrome P450 71A4-like	21	26	123	H01(53), H02 (36), H03 (11), H04(6)	Upstream	14331	H01, H03

	OA-LA17	5	18157188	A,G	g43427; Cytochrome P450 71A4-like	21	24	123	H01(53), H02 (36),H03 (11),H04(6)	Upstream	26	H01, H03
	OA-LA20	5	18141916	A,C	g43426; Cytochrome P450 71A4-like	14	23	117	H01(73), H02 (25),H03 (8)	Upstream	43	H01
	OA-LA33	5	18179456	T,A	g43429 and g43430.t1; Cytochrome P450 71A2-like	18	18	122	H01(85) H02(21)	Downstream	5875	
	OA-LA35	6	84242096	T,A	g53664; Probable calcium-binding protein CML25	52	20	120	H01(69)	Upstream	2183	H01
	OA-LA5	7	66993411	G,A	g57141; Cytochrome P450 710A11-like	26	16	121	H01(70) H02(27)	Upstream	608	
**Oil Content**	OC2	2	65505220	T,C	g28700; Pentatricopeptide repeat-containing protein At4g20740	83	16	122	H01, H02(3), H03(3), H04(3)	Upstream	71	H01, H96
	OC4	5	5164369	C,T	g42440; Purple acid phosphatase 27	11	6	123	H01(82), H02 (14),H03 (9),H04(7),H05(3),H06(2)	Gene within Haplotype	0	H01, H02

	OC6	9	18518779	A,G	g53664; Probable UDP-N-acetylglucosamine--peptide N-acetylglucosaminyltransferase SPINDLY isoform X1	16	15	122	H01(107)	Gene within Haplotype	0	H06, H07,H09,H01
	OC9	9	79566941	G,A	g67871; Retrovirus-related Pol polyprotein from transposon TNT 1-94	3	2	122	H01(108), H02 (8),H03 (7)	Upstream	2428	H01, H03
	OC1	11	76393484	G,A	g16920; Protein BIG GRAIN 1-like E	71	40	116	H01(30), H02 (7),H03 (6),H04(3),H05(3),H06	Gene within Haplotype	0	
	OC8	11	76395157	C,T	g16920; Protein BIG GRAIN 1-like E	67	39	116	H001(33), H02 (7),HO3 (6),HO4(3),HO5 (3),HA006(3)	Gene within Haplotype	0	H01, H40,H58
**Number of Primary Branches**	PB4	5	5018372	A,C	g42435; Acetolactate synthase 1, chloroplastic	29	14	122	H01(94)	Upstream	3704	H01, H28
	PB9	5	3333942	T,C	g42292; F-box/LRR-repeat protein 14-like isoform X1	26	15	122	H01(96)	Upstream	688	H01, H08

	PB1	12	63076322	C,T	g20942. and g20943;<br>Putative pentatricopeptide repeat-containing protein At1g12700, mitochondrial	31	17	119	H01(86), H02(3)	Gene within Haplotype	0	
**Plant Height**	PH3	2	10029119	A,G	g25891;<br>Vignain-like	63	13	122	H01(54), H02 (6)	Downstream	1031	H01, H48
	PH4	3	15050693	G,A	g32062;<br>Trafficking protein particle complex subunit 6B (TRAPPC6B)	20	19	122	H01 (103)	Downstream	872	

Figure

Click here to access/download;Figure;Fig 1.jpg

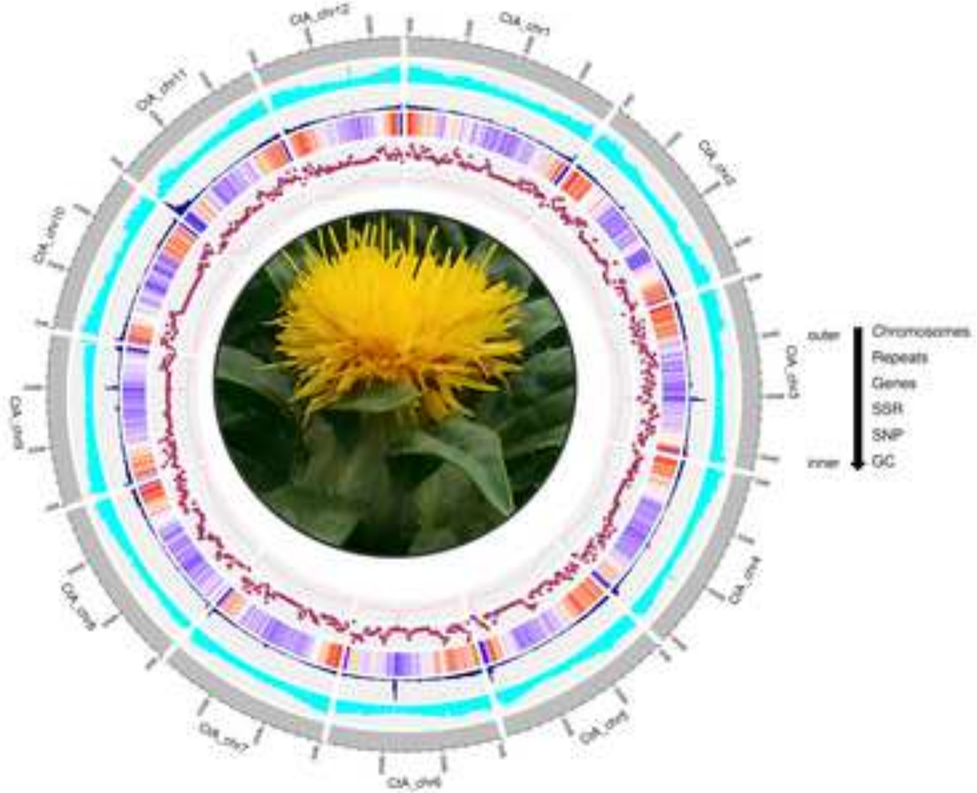

Figure

Click here to access/download;Figure;Fig 2.jpg

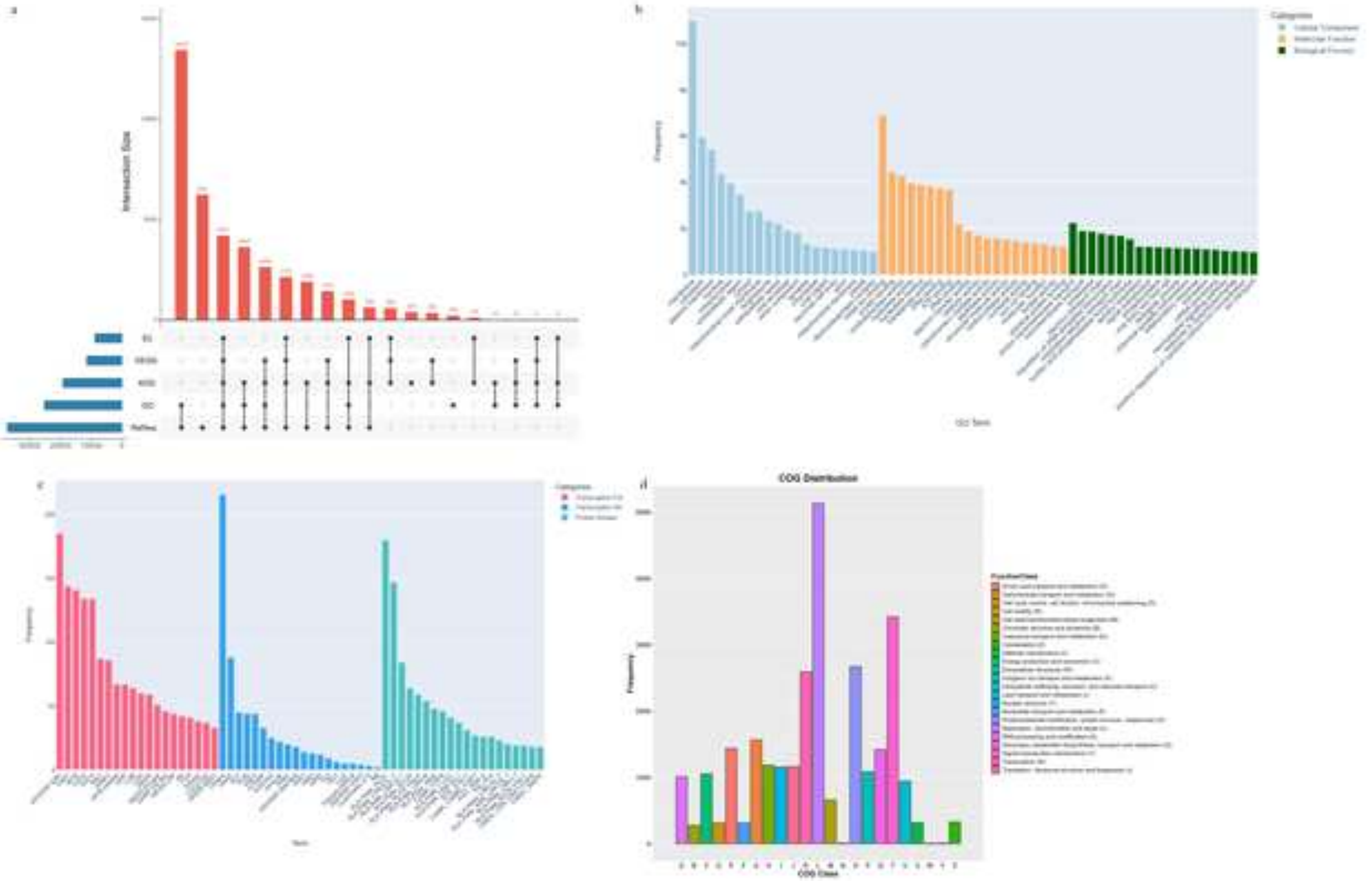

Click here to access/download;Figure;Fig 3.jpg

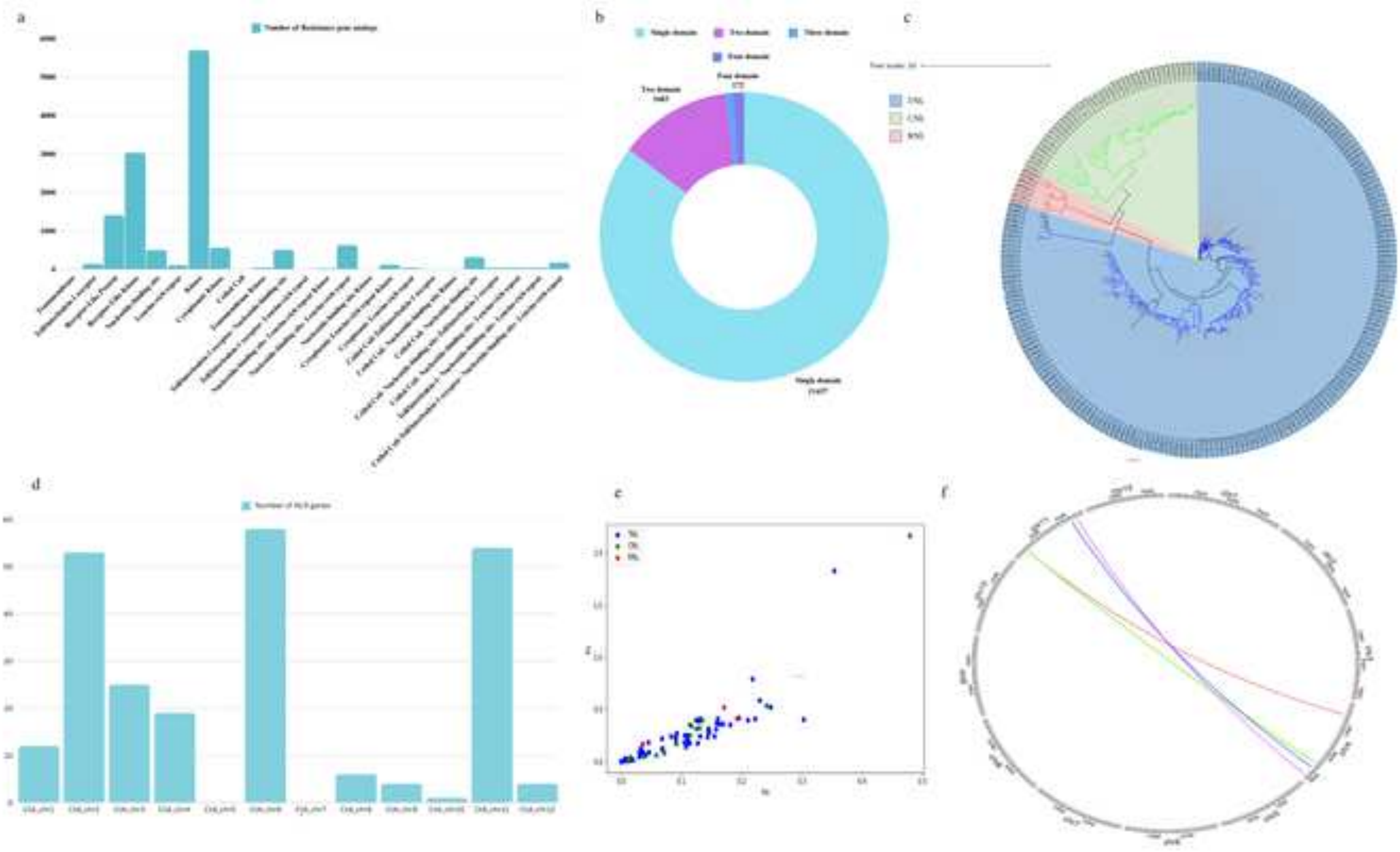

Figure

Click here to access/download;Figure;Fig 4.jpg

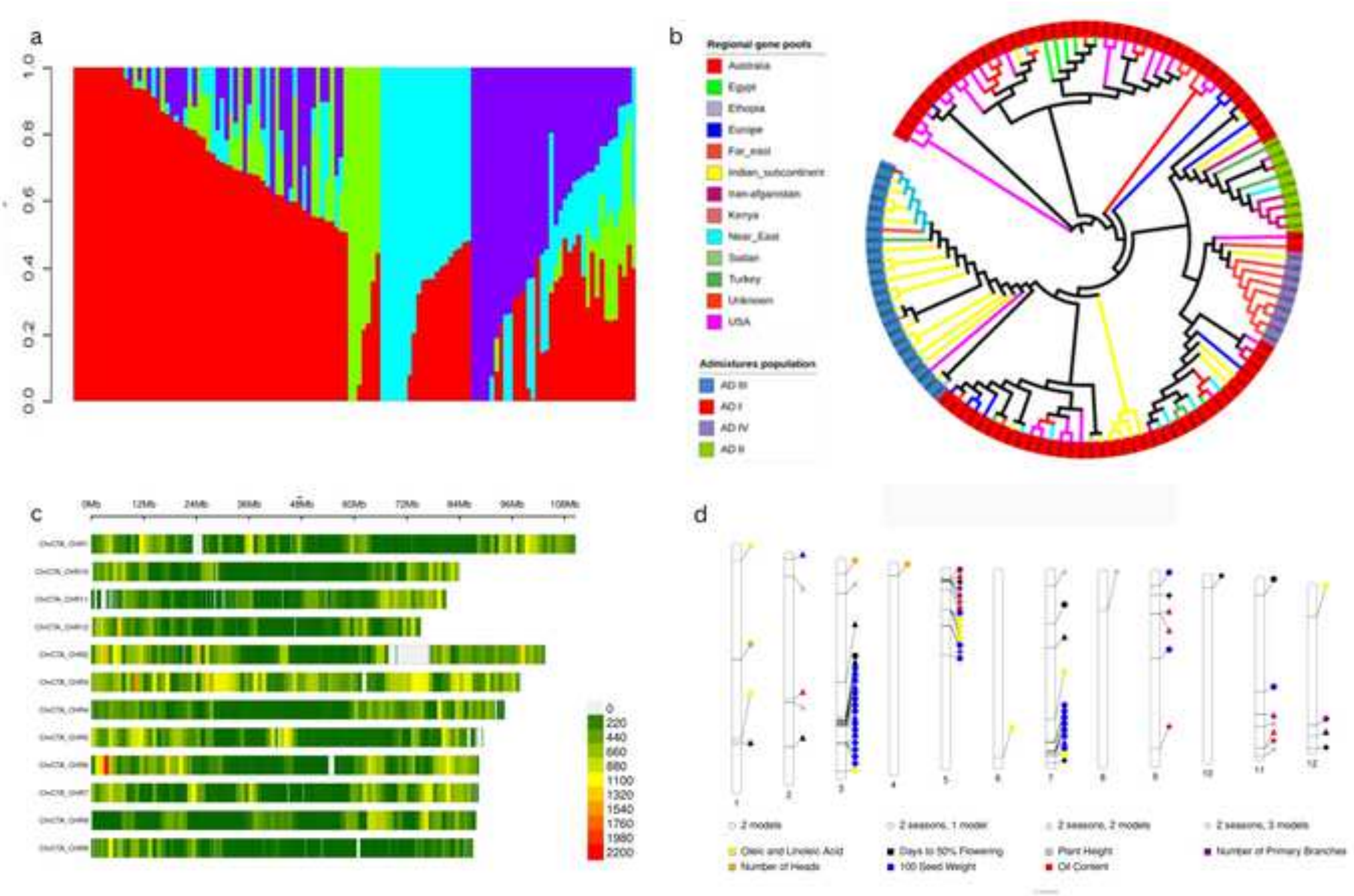

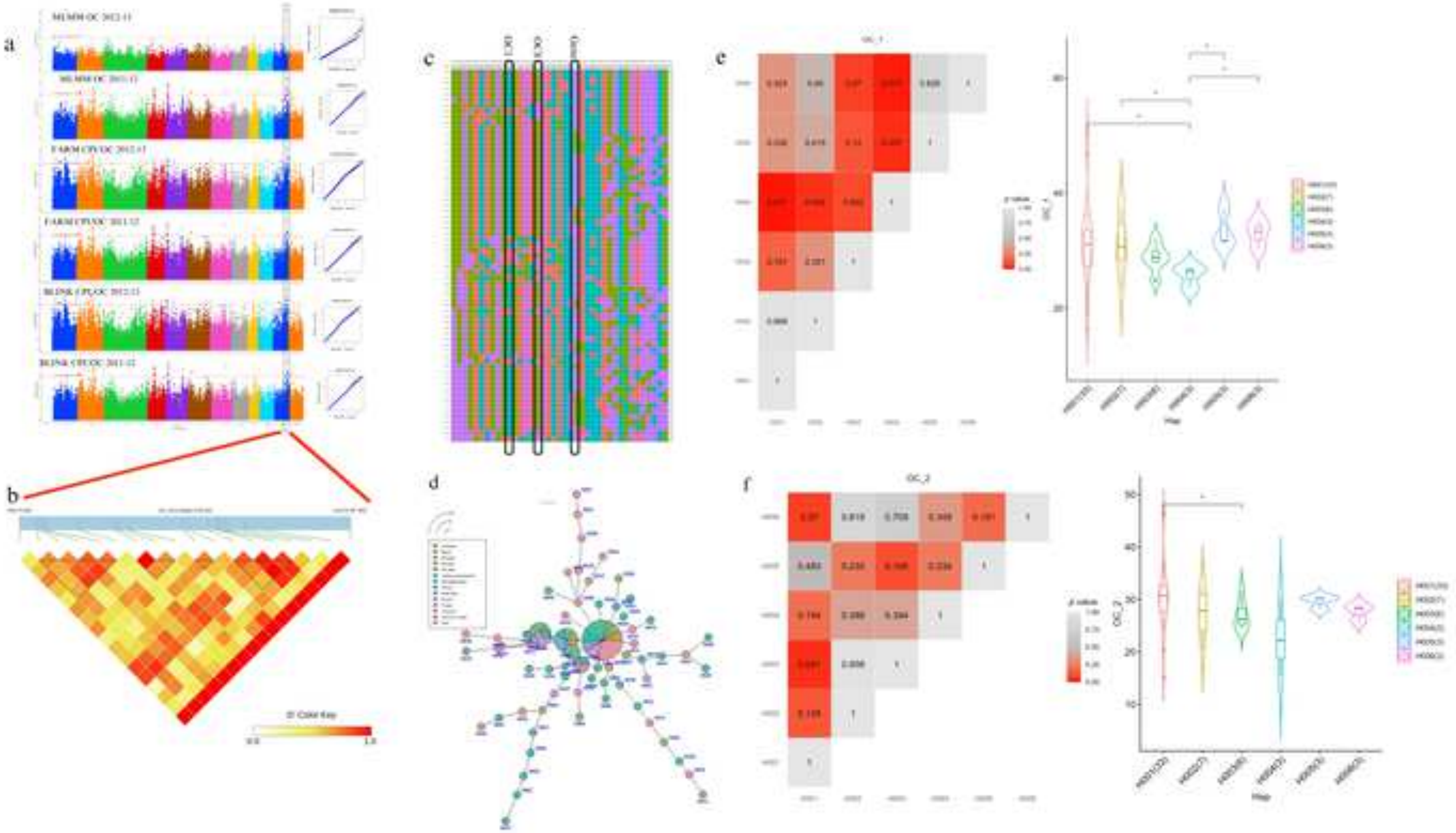
a
b
c
d
e
f

Figure Click here to access/download;Figure;Fig 6.jpg

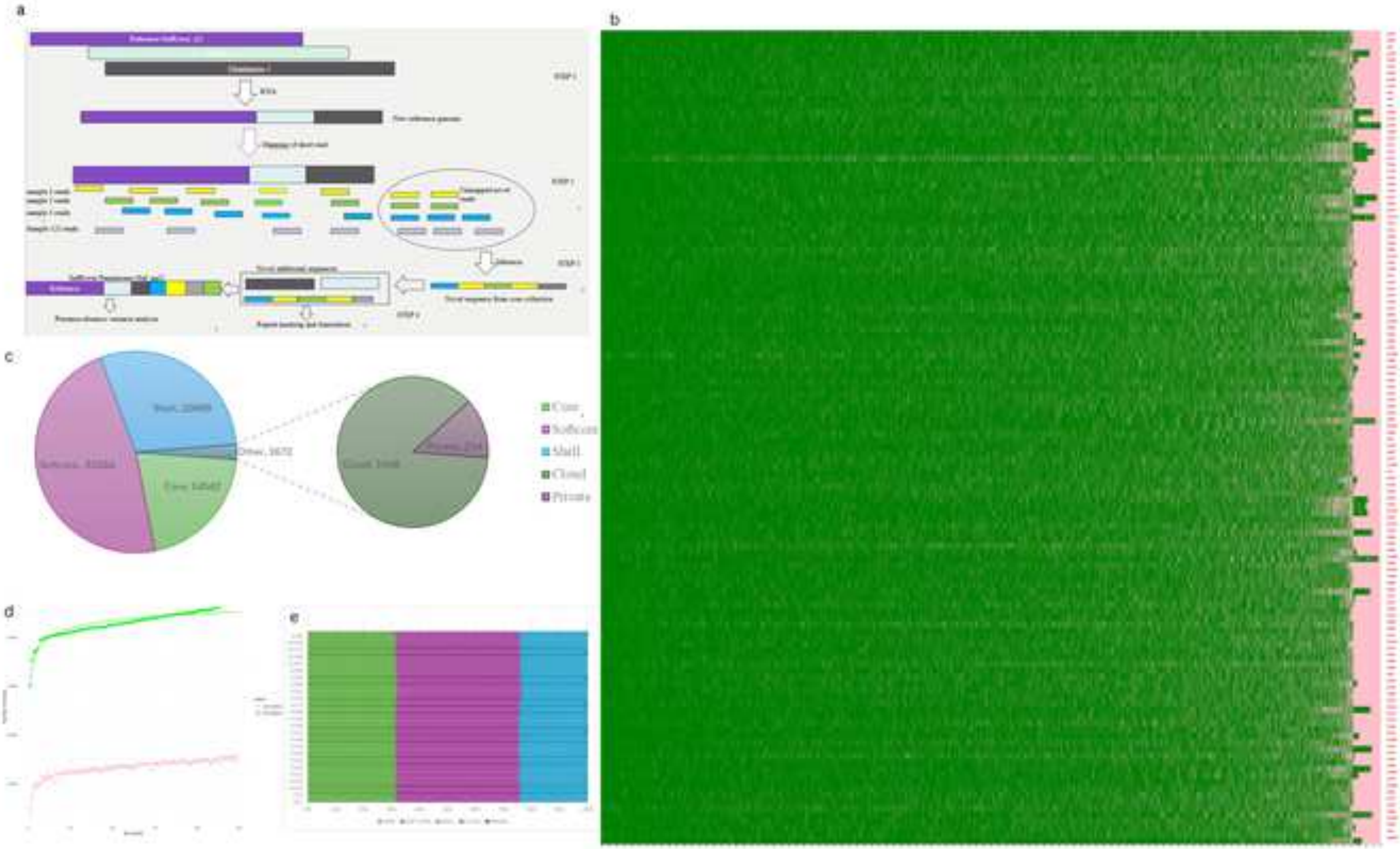

Click here to access/download
Supplementary Material
Supplementary information edited VB.docx

Dear Dr. Scott Edmunds,

Editor-in-Chief

GigaScience

We are re-submitting our manuscript entitled "**Improved reference assembly and core collection re-sequencing to facilitate exploration of important agronomical traits for the improvement of oilseed crop, *Carthamus tinctorius* L.**", to be considered for publication in your esteemed journal. The manuscript was earlier entitled as "An improved chromosome-level assembly, core collection-based association mapping, and pan-genome analysis to facilitate improvement in the oilseed crop, Safflower (*Carthamus tinctorius* L.)" (GIGA-D-24-00287). It was handled by Dr. Hongfang Zhang and the outcome of the previous review process was given as "resubmission after addressing all the concerns raised by reviewers". Based on the suggestions provided by the Editor and reviewers, we have addressed all the raised concerns and revised the manuscript to incorporate the changes. A detailed response sheet to the queries raised in the previous review is enclosed with this letter. The revised manuscript describes the following key findings:

(i) We report a significantly improved *de novo* chromosome-scale reference genome for safflower. The sequenced accession harbors several important traits of agronomic value thus the generated assembly will be crucial to the breeding community. Additionally, our manuscript describes a comprehensive repertoire of disease resistance genes and genes contributing to oil content, oil composition, and pigments, which are important economic traits of the crop and would facilitate improvement programs in the crop.

(ii) A SNP-based, high-density linkage map for the crop using a RIL population derived from parental lines contrasting in several traits of interest. To the best of our knowledge, this is the first report of a high-density linkage map in safflower.

(iii) Resequencing of safflower core collection which includes 123 accessions representing high diversity at geographical, phenotypic and molecular scales.

(iv) Core collection-based genome-wide association mapping (GWAS) for several traits of agronomic value, candidate gene and haplopheno-analysis leading to identification of several significant marker-trait associations.

(v) A pan-genome that provides critical insights into regional genome specificities and an additional 11,000 genes. The enrichment of variable genes was mainly detected in the categories viz. regulation of biological processes, response to stimulus, catalytic and binding as well as disease resistance.

(vi) Finally, we have established an accessible database of safflower "The Safflower Genome Resource" wherein all the genomic and genetic resources generated by our study have been provided for ready use by the scientific community.

Accessible links for the submitted data are given below:

1. The raw sequencing data generated for the Genome Assembly has been deposited at NCBI under the BioProject PRJNA1089929.
2. The genome assembly, functional annotation, transcript and protein sequences, Bionano Optical Maps and VCF file for the SNP data, are available at the Safflower Genome Resource (SGR: http://13.60.187.179:3002/downloads), developed by us.
3. The phenotypic data of the core collection used for GWAS is available at Figshare. The link for the reviewer is "https://figshare.com/s/c8efdcf9b799bf669685".
4. Pangenome assembly and its annotation are also available at Safflower Genome Resource (SGR: http://13.60.187.179:3002/downloads).

All authors are aware of the submission and have given their consent. No AI-assisted technology is used in drafting the Manuscript. The contents of the manuscript have not been submitted or published elsewhere.

We would greatly appreciate a positive consideration of our manuscript and the opportunity to contribute to the scientific discourse in your esteemed journal. We look forward to your feedback and guidance.

Warm regards,

Prof. Shailendra Goel and Prof. Arun Jagannath
(Joint corresponding authors)
Department of Botany
University of Delhi
India
shailendragoel@gmail.com; jagannatharun@yahoo.co.in

Reviewer response sheets:

	**Reviewer Comment**	**Authors answer**
	**Reviewer 1**	
1	The article's structure requires refinement. For example, the database section could be developed into a standalone paper rather than being integrated into the current work. Databases are not the culmination of genomic research but rather tools that leverage genomic data. The author might consider expanding this section for separate publication.<br><br>Additionally, details like genome size estimation might not warrant a separate section at this stage, suggesting a need for better balance in the depth of content.	Thank you for reviewing our manuscript and providing your useful assessment for it. Based on your suggestion, we have reduced this section significantly and have only provided a link for access to the database. We never intended to show database as a culmination of the research. Our primary goal is to share the generated data with the research community to facilitate ready adoption of the generated resources.<br><br>We have removed the separate section explaining the genome size, and this portion is now integrated into the genome assembly section.
2	The emphasis on certain basic data, such as LAI and BUSCO, seems misplaced. Given the progress in sequencing technology and the use of tools like Hifiasm, genome completeness is no longer a novel aspect. The focus should instead be on the novel insights and annotations that this genome offers compared to the previously published versions and the potential implications of these findings	The suggestion has been implemented.<br>We have given LAI and BUSCO as an indicator for the quality of the assembled genome. Following the reviewer's suggestion, additional analyses have been carried out. The revised text now focuses on biological significance and evolutionary insight into genome expansion based on the analysis of retrotransposons.
3	In the GWAS analysis, the author presents insights into population structure and identifies several associated loci. However, the lack of functional experimental validation is notable. At a minimum, a more detailed bioinformatics analysis is necessary to substantiate the findings.	As per the reviewer's suggestion, we have expanded our bioinformatics approach to include candidate gene analysis and haplotype-phenotype (haplopheno) analysis to further support the identified loci associated with traits of importance.
4	For the pangenome analysis, it would be beneficial to include the previously published genomes in the comparison before moving to a pangenome based on second-generation sequencing. The quality of the previously published genomes is likely to be more reliable than that of assemblies based on short reads.	The suggestion has been implemented.<br>In response to the reviewer's suggestion, we have modified our methodology. We have incorporated two previously published genomes (Wu et al. 2021, Chen et al. 2023) along with our reference genome, creating a new reference sequence before including the second-generation sequencing data for pan genome analysis.
	**Reviewer 2:**	

1	Line 264: The accuracy of gene annotation is very basic and important. This article mentions that a total of 65,298 protein coding genes were annotated, which may be the result of data analysis errors. It is recommended to re annotate and analyze. As far as I know, there should not be so many genes in a genome of about 1G.	Thank you for critical assessment of our manusc and your suggestions for its improvement. Conside your suggestion, we have repeated our annota analysis and filtered the genes which show a continu repeat coverage of ≥30% over their boundaries. U filtering, 59,995 transcripts were retai Collectively, ~ 80% (47,704) of the transcripts w annotated with at least one functional term from vari public databases. The gene models corresponded 39,945 unigenes (at 80% similarity). The entire anal has been performed with highly stringent filters remove potential false positives.<br>We would like to bring it to the reviewer's kind notice that protein-coding genes are not unigenes, and the confusion might have been because of the terminology, we have now tried to remove this confusion throughout the manuscript. We hope that we could address your concern.<br>Similar number genes have been reported in recent studies (Jiang et al., 2020, https://doi.org/10.1093/gigascience/giaa015; Wang et al., 2024; https://doi.org/10.1016/j.jare.2024.12.038)
2	The Supplementary Fig. S4 did not show significant interaction regions, and there may be significant errors based on such assembly.	We have implemented reviewer's suggestion. We have earlier used Pretext Viewer tool to visualize the Hi-C interactions. This has posed certain challenges as pointed out by the reviewer. To address the concern, we have now regenerated the Hi-C interaction map for visualization using JUICER pipeline (https://github.com/aidenlab/juicer). The figure in Fig. S4 has been accordingly updated and interaction regions could be easily followed.
3	Line 237: This study mentioned the identification of 32 CHS, much more than sunflowers, but this result should be incorrect. Currently, other reported genomes do not contain too many CHS. Also, these protein coding sequences should be presented in the attachment.	We apologize for the error in these findings. There was an error in updating the list after detecting the gene sequences based on C or N-terminal CHS domain leading to an inflated count. After reconciling the list, this error has been now rectified, and the number of genes has been revised to 8. We have included these sequences of the putative genes in our SGR database.
4	Two websites provided by the author (http://115.246.222.210:3002/ http://14.139.45.108:8484/) cannot be opened, so website accessibility should be provided.	The genome database has now been successfully uploaded to an Amazon Web Services (AWS) server, and it can be accessed using the updated link: http://13.60.187.179:3002/ . We have updated the link in the manuscript as well. This

		information was earlier communicated to the journal during submission process however it seems that reviewers did not receive it. We apologies for any inconvenience this may have caused.
5	The authors mentioned pan genome assembly but did not describe the sample characteristics for pan genome analysis. I do not understand what Safpg_V1 represents.	The accessions used for re-sequencing and pan genome analysis belonged to a safflower core collection reported earlier by our research group (Ambreen et al., 2018 https://doi.org/10.3389/fpls.2018.00402 ; Kumar et al., 2015 https://doi.org/10.1007/s11105-014-0828-8). These manuscripts provide detailed information about the core collection including its phenotypic and genetic variability.<br>However, to address the reviewer's concern and enable ready information to the readers, we have now included a Supplementary Table S17, which describes the geographical locations and accessions numbers of the core collection. Rest of the information can be accessed from earlier publications.<br><br>"Safpg_V1" is the name given to the safflower pangenome generated in this study.
