# Supplementary material for: Improved reference assembly and core collection resequencing to facilitate exploration of important agronomical traits for the improvement of oilseed crop, Carthamus tinctorius L
Source: Gigascience. 2025 Dec 11;15:giaf151. doi: 10.1093/gigascience/giaf151 (PMC12888819; doi:10.1093/gigascience/giaf151)
Supplement: giaf151_GIGA-D-25-00014_Revision_1 [file giaf151_giga-d-25-00014_revision_1.pdf]

GigaScience

Improved reference assembly and core collection re-sequencing to facilitate exploration of important agronomical traits for the improvement of oilseed crop, Carthamus tinctorius L.

--Manuscript Draft--

**Manuscript Number:**	GIGA-D-25-00014R1
**Full Title:**	Improved reference assembly and core collection re-sequencing to facilitate exploration of important agronomical traits for the improvement of oilseed crop, Carthamus tinctorius L.
**Article Type:**	Research
**Funding Information:**	Department of Biotechnology, Ministry of Science and Technology, India (BT/Ag/Network/Safflower/2019-20; Sub Projects 3 and 4) — Dr. Arun Jagannath

Abstract:

Background

Safflower (Carthamus tinctorius L.) is a drought-resilient oilseed crop. Besides producing edible oil rich in oleic and linoleic acid, it is also used in biofuels, cosmetics, colouring dyes, pharmaceuticals and nutraceuticals. Despite its significant economic uses, availability of genetic and genomic resources in safflower are limited.

Results

We report an improved de novo genome assembly of safflower (Safflower_A2). A chromosome-level assembly of 1.15 Gb with telomeres and centromeric repeats, was constructed using PacBio HiFi reads, optical maps, Illumina short reads, and Hi-C sequencing. Safflower_A2 shows better contiguity, completeness, and high-quality annotation than previous assemblies. The assembly was further validated with the help of a single nucleotide polymorphism (SNP)-based linkage map. A genome-wide survey identified genes for disease resistance, oil quality, oil content and pigments. Employing the de novo genome assembly as a reference, we used resequencing data of a global core-collection of 123 accessions to carry out a SNP-based genome-wide association study, which identified significant associations for several traits of agronomic value, including seed oil content. Resequencing data was also applied for a pan-genome analysis which provided critical insights into genome diversity identifying an additional ~11000 transcripts and their functional enrichment that will be useful for region-specific breeding lines.

Conclusion

Our study provides insights into the genomic architecture of safflower by leveraging an improved genome assembly and annotation. Additionally, resources including high-density linkage map, marker-trait associations, and pan-genome developed in this study provide valuable resources for use in breeding and crop improvement programs by the global research community.

**Corresponding Author:**	Shailendra Goel, Ph.D.<br>University of Delhi<br>New Delhi, Delhi INDIA
**Corresponding Author Secondary Information:**	
**Corresponding Author's Institution:**	University of Delhi
**Corresponding Author's Secondary Institution:**	
**First Author:**	Megha Sharma
**First Author Secondary Information:**	

Powered by Editorial Manager® and ProduXion Manager® from Aries Systems Corporation

Order of Authors:	Megha Sharma
	Varun Bhardwaj
	Praveen Kumar Oraon, Ph.D
	Shivani Choudary
	Heena Ambreen, Ph.D.
	Rohit Nandan Shukla
	Harsha Rayudu Jamedar, Ph.D
	Ajitha VIjjeswarapu, Ph.D
	Vandana Jaiswal, Ph.D
	Palchamy Kadirvel, Ph.D
	Arun Jagannath, Ph.D
	Shailendra Goel, Ph.D.
Order of Authors Secondary Information:	
Response to Reviewers:	We thank the reviewer for their insightful comments and acknowledge their contribution towards improving the manuscript. We have made every effort to address their concern and hope that we could improve the manuscript to their satisfaction<br><br>Detailed response to the reviewers queries has been attached with the cover letter.
Additional Information:	
Question	Response
Are you submitting this manuscript to a special series or article collection?	No
**Experimental design and statistics**<br><br>Full details of the experimental design and statistical methods used should be given in the Methods section, as detailed in our Minimum Standards Reporting Checklist. Information essential to interpreting the data presented should be made available in the figure legends.<br><br>Have you included all the information requested in your manuscript?	Yes
**Resources**<br><br>A description of all resources used, including antibodies, cell lines, animals and software tools, with enough information to allow them to be uniquely identified, should be included in the Methods section. Authors are strongly encouraged to cite Research Resource	Yes

Powered by Editorial Manager® and ProduXion Manager® from Aries Systems Corporation

Identifiers (RRIDs) for antibodies, model organisms and tools, where possible.<br><br>Have you included the information requested as detailed in our Minimum Standards Reporting Checklist?	
**Availability of data and materials**<br><br>All datasets and code on which the conclusions of the paper rely must be either included in your submission or deposited in publicly available repositories (where available and ethically appropriate), referencing such data using a unique identifier in the references and in the "Availability of Data and Materials" section of your manuscript.<br><br>Have you have met the above requirement as detailed in our Minimum Standards Reporting Checklist?	Yes
GigaScience has policies and guidelines in place for the use of generative AI-writing tools such as ChatGPT. If you have used such writing tools to assist with writing the manuscript this must be declared and cited in the text. Authors should not list AI-writing tools and other AI-assisted technologies as an author or co-author and should acknowledge that they are fully responsible for text generated or refined by AI-writing tools.<p><br><br>A summary of use (particularly in the introduction or among methods) needs to be included at the end of the paper, and the outputs should also be included as a supplementary file hosted in GigaDB or other open repositories. Please read our guidelines for more information. <p><br><br>By submitting to GigaScience, you are aware of the journal's AI-writing tools policy, and if you have declared use of such tools below, you have acknowledged this where appropriate in your manuscript and have made a summary of use and outputs available. <p>	No

<b>AI-assisted writing tools have been used in the preparation of this manuscript?	

Manuscript Click here to access/download;Manuscript;manucript_gigascience_final_revie

Improved reference assembly and core collection re-sequencing to facilitate exploration of important agronomical traits for the improvement of oilseed crop, *Carthamus tinctorius* L.

Megha Sharma[1#], Varun Bhardwaj[1#], Praveen Kumar Oraon[1], Shivani Choudhary[1], Heena Ambreen[2], Rohit Nandan Shukla[3], Harsha Rayudu Jamedar[4], Ajitha Vijjeswarapu[4], Vandana Jaiswal[5], Palchamy Kadirvel [4], Arun Jagannath[1*], Shailendra Goel[1*]

1. Department of Botany, University of Delhi, Delhi 110007, India
2. Department of Biosciences, University of Exeter, Exeter, EX4 4QD, United Kingdom
3. Bionivid Technology Pvt. Limited, Bengaluru 560064, India
4. ICAR-Indian Institute of Oilseeds Research, Hyderabad-500030, India
5. CSIR-Institute of Himalayan Bioresource Technology, Palampur, Himachal Pradesh 176061, India

*Corresponding authors: shailendragoel@gmail.com, jagannatharun@yahoo.co.in

#Co-first author- These authors contributed equally to the work.

Abstract:

Background - Safflower (*Carthamus tinctorius* L.) is a drought-resilient oilseed crop. Besides producing edible oil rich in oleic and linoleic acid, it is also used in biofuels, cosmetics, colouring dyes, pharmaceuticals and nutraceuticals. Despite its significant economic uses, availability of genetic and genomic resources in safflower are limited.

Results - We report an improved *de novo* genome assembly of safflower (Safflower_A2). A chromosome-level assembly of 1.15 Gb with telomeres and centromeric repeats, was constructed using PacBio HiFi reads, optical maps, Illumina short reads, and Hi-C sequencing. Safflower_A2 shows better contiguity, completeness, and high-quality annotation than previous assemblies. The assembly was further validated with the help of a single nucleotide polymorphism (SNP)-based

linkage map. A genome-wide survey identified genes for comprehensive exploration of disease resistance in the safflower. Employing the *de novo* genome assembly as a reference, we used resequencing data of a global core-collection of 123 accessions to carry out a SNP-based genome-wide association study, which identified significant associations for several traits, their haplotypes of agronomic value, including seed oil content. Resequencing data was also applied for a pan-genome analysis which provided critical insights into genome diversity identifying an additional ~11000 genes and their functional enrichment that will be useful for region-specific breeding lines.

Conclusion - Our study provides insights into the genomic architecture of safflower by leveraging an improved genome assembly and annotation. Additionally, resources including high-density linkage map, marker-trait associations, and pan-genome developed in this study provide valuable resources for use in breeding and crop improvement programs by the global research community.

Keywords: Safflower, Genome assembly, Core collection, Optical mapping, Resistance genes, Genome-Wide Association Study, Candidate gene analysis, Haplotypes, Pan-genome, KASP

Introduction

Safflower (*Carthamus tinctorius L. 2n = 24*), a member of the family Asteraceae, is a drought-resilient diploid oilseed crop. The crop produces edible oil with a unique profile consisting of nutritionally desirable unsaturated fatty acids [1]. The seed oil is also a rich source of phospholipids, phytosterols, phenols, and tocopherols which makes it highly valuable for diverse pharmaceutical and nutraceutical applications [2,3]. Safflower is currently cultivated across ~23 countries in a total area of ~1.23 million hectares, producing 1.10 million tonnes of seed [4]. The largest global producers of the crop are Kazakhstan, Russian Federation, United States of America, Mexico and India, accounting for more than 85% of the global seed production [4]. Currently, safflower has a market value of $232.1 million, however due to its ability to grow under drought conditions, its market is expected to increase to ~$355 million in a decade as drought conditions become more prevalent [5]. Despite its economic scope, safflower has observed a decline in acreage due to multiple factors like spiny nature of the plant, susceptibility to various biotic and abiotic stresses, and scarcity of cultivars with high yield and oil content [6].

Several studies have established the diverse genetic pool of safflower harbouring significant morphological, geographical and molecular diversity [7–10]. Further, primary and secondary diversification of safflower has also led to the development of varieties with distinct traits that emerged over time [11]. Owing to the vast variability observed in the crop, a single genome sequence would not sufficiently reflect the full repertoire of genes available in the crop. Resequencing multiple diverse genotypes encompassing the global diversity would enable generation of crucial genomic resources that would be valuable for expediting safflower breeding programmes. Currently, reference genomes are available for two safflower varieties, ‘Anhui 1’ [12] and ‘Chuanhonghua 1’ [13] which are region-specific and do not represent all desirable traits

of global relevance. Moreover, while resequencing of 220 accessions reported by [13] provides valuable insights, ~70% accessions of the sequenced panel are from the Chinese gene pool which does not adequately encapsulates the global diversity of the crop. This necessitates a comprehensive exploration of global germplasm for study and deployment of broader genomic diversity.

Here, we report an improved *de novo* chromosome-level genome assembly of an elite safflower accession (hereafter designated as "Safflower_A2") characterized by several desirable traits of high agronomic value *viz*., high oil content (~47%), high oleic acid (~80%), higher seed yield, large head diameter (~24mm) and high head number (~60 per plant) (Supplementary Fig. S1). The genome assembly was derived through integration of multiple sequencing technologies including PacBio HiFi, Bionano optical maps, Hi-C and Illumina paired-end short reads. Additional support for anchoring the generated genome assembly was provided by a SNP based high-density genetic map generated by Genotyping by Sequencing (GBS) of a Recombinant Inbred Line (RIL) mapping population (F_8) developed using A2 as one of the parents. The genome assembly generated in this study have been utilized to generate a repertoire of Resistance Gene Analogs (RGAs) for ready to implement in crop improvement studies. Further, we performed re-sequencing of 116 accessions of a core collection reported earlier by our group [14] and 7 additional accessions of agronomical importance and employed it for genome-wide association studies (GWAS), which revealed crucial loci for several agricultural traits of interest, including seed oil content. Haplotype analysis was carried out to identify associated agronomically important traits. The SNPs identified were validated using the Kompetitive Allele-Specific PCR **(**KASP) analysis. Subsequently, we have constructed a pan-genome for safflower revealing distinct functional enrichments among pan-genes. To ensure direct availability of safflower resources and datasets to the scientific community,

we present "Safflower Genome Resource", a comprehensive database housing generated genomic resources including reference genome, protein-coding genes, Simple Sequence Repeats (SSRs) and SNPs. The database will provide essential support to the global plant breeder community in advancing trait improvement efforts in safflower.

Results

Development and evaluation of Safflower Genome Assembly

The genome size of Safflower_A2 was estimated using flow cytometry and k-mer analysis. Flow cytometric analysis indicated an approximate size of 1.37 picograms (2C), which corresponds to ~1.34 Gb (Supplementary Fig. S2 a, b, c), and is in line with the previously predicted size for safflower [15,16]. Genome size estimation using k-mer (k=17) distribution of HiFi long reads (Supplementary Table S1) gave an estimate of ~1.17 Gb (Supplementary Fig. S2 d).

A combination of four different sequencing technologies, including PacBio HiFi reads, optical mapping, Hi-C and Illumina paired-end short read sequencing was used for generation of the *de novo* reference genome assembly of safflower (Safflower_A2) (Supplementary Fig. S3). A total of 38.5 Gb of HiFi reads (~30X coverage) with a mean length of 13 kb and an accuracy of >99% were generated for the construction of contig-level genome assembly (Supplementary Table S2). Firstly, 3,444,538 HiFi long reads were constituted into a contig level assembly of 1.15 Gb comprising 2,427 contigs. Thereafter, the contigs were scaffolded using long optical maps, resulting in a scaffold-level assembly of 1.09 Gb comprising 31 scaffolds. Using paired-end linked reads produced by Hi-C sequencing, the 31 scaffolds generated above were integrated into 21 super-scaffolds and an additional scaffold that corresponded to the chloroplast genome. Finally,

through careful manual curation, the scaffolds were anchored into 12 pseudochromosomes (2n=24) (Fig. 1a, Supplementary Fig. S4). The total length of the final anchored assembly was ~1.09 Gb with an N50 of 88.40 Mb and N90 of 81.10 Mb (Table 1). Our assembly further consist of 1,680 unplaced small contigs that were < 0.5 Mb in length with a total size of 68.5 Mb, making total length of assembly to 1.15Gb. The contiguity, completeness and accuracy of the genome was evaluated by mapping the short Illumina (99.29%) and long PacBio reads (95.29%) onto the generated assembly (Supplementary Table S3), complete Benchmarking Single Copy Orthologs (BUSCO) score (97.90%) (Supplementary Table S4), k-mer completeness score (97.73%), consensus quality value (QV) (68.31%) (Supplementary Table S5) and LTR assembly index (LAI) (22.49). The contiguous nature of the assembly enabled delineation of telomeres and centromeres in the safflower genome for the first time. The most frequent telomeric repeat was AACCCTG with counts ranging from 7 to 1,429. We identified telomeres at one end of nine chromosomes and at both ends of three chromosomes (Supplementary Fig. S5). We detected centromeric repeats on all the chromosomes. These were of four different lengths (342 bp, 348 bp, 349 bp, and 350 bp) with counts ranging from 5 to 1,272 in the genome. The 349 bp repeat was the most abundant and present on all chromosomes except chromosomes 3 and 7 (Supplementary Fig. S5). Further, the chloroplast (cp) genome was assembled as a single circular contig of 153,026 bp (Supplementary Fig. S6). Its annotation identified 205 protein-coding genes which were higher than the 127 cp genes reported earlier [15].

Safflower_A2 exhibits better quality as compared to the other two published genome assemblies (Table 1). Whole genome alignment of Safflower_A2 assembly with the Anhui 1 [12] genome revealed high consonance between the two genomes (Supplementary Fig. S7a). However, the one-to-one alignment suggests genome assembly of Chuanhonghua 1 [13] is fragmented in nature

(Supplementary Fig. S7b). The structural variant (SV) analysis detected large translocations in the Chuanhonghua 1 genome (Supplementary Fig. S7c). However, such translocations were not detected when aligned with Anhui 1 genome (Supplementary Fig. S7d). Deletions (DEL) were the most abundant SV type across both alignments, predominantly in the 1–10 kb range (Supplementary Fig. S7e). Tandem duplications (DUP: TANDEM) were more frequent than interspersed duplications (DUP:INT), while inversions (INV) were less common but distributed across all size categories. Although total SV counts were higher when mapped to the Chaunhongua 1 genome, the overall size-based distribution of SVs remained consistent among the genomes (Supplementary Fig. S7f, g).

Construction of a high-density genetic linkage map and assignment of chromosomes

A total of 151 Gb paired-end GBS data was generated for 121 lines of a RIL population (A2 X A1; designated “Population A”) with average coverage of ~1.15x (0.33x-1.82x) per individual. Variant calling yielded 1.49 million SNPs, which were filtered using stringent criteria (Supplementary Table S6) and a final set of 15,732 high quality SNPs were used to construct the first SNP-based high-density linkage map in cultivated safflower comprising 12 linkage groups (LG1-LG12; Supplementary Fig. S8, Supplementary Table S7). The map spanned 1,581.05 cM, with linkage groups ranging in length from 65.70 cM (LG 9) to 209.08 cM (LG 1). The average number of markers per linkage group was 1,311 and ranged from 3,587 in LG 8 to 217 in LG 10. Average marker distance was 9.28 per cM, ranging from 5.72 per cM (LG3) to 24.76 cM (LG8). This genetic linkage map was anchored to the genome assembly, which showed concordance with the genetic maps confirming the accuracy of assembled genome (Supplementary Fig. S9)

Full-length transcriptome sequencing and detection of alternative splicing events

Transcriptomic libraries were generated from eight different tissues of safflower *viz*., shoot, leaf, root, flower, bud and various seed developmental stages (5 DAP, 10 DAP, 20 DAP and 30 DAP; DAP: days after pollination) (Supplementary Table S8). Long read PacBio sequencing yielded 3,772,953 circular consensus sequences (CCS) reads, which resulted in 2,22,133 full-length high-quality transcripts. The obtained transcripts were aligned to the repeat-masked reference Safflower_A2 genome. The alignment data was used as evidence for gene prediction and detection of alternative splicing events. SUPPA2 detected seven types of Alternative Splicing (AS) events (totalling 3,826), namely: retained intron (RI), skipping exon (SE), alternative 5'/3' splice site events (A5S/A3S), alternative first/last exons (AF/AL) and mutually exclusive exons (MX). A5S were the most abundant and MX were the rarest type of splicing events accounting for 57.05% and 0.26% of total local events, respectively (Supplementary Table S9).

Annotation of the repeatome and detection of LTR for the genome expansion

We identified 787.75 Mb of repetitive elements constituting ~71.3% of the total length in the Safflower_A2 genome (Fig. 1b, Supplementary Table S10, Supplementary Fig. S10). Retrotransposons [class I Transposable Elements (TE)] were the most dominant repetitive category with its sub-class long terminal repeats (LTRs) representing the major component (43.6%) of repetitive elements comprising of 22.32% Ty3/*Gypsy* and 21.3% Ty1/*Copia* elements. Whereas non-LTR retroelements (long interspersed nuclear elements: LINEs and short interspersed nuclear elements: SINEs) constituted 0.87% of the repetitive elements. Using domain-based Annotation

of Transposable Elements (DANTE), we assigned 70% of the Ty1/*Copia* elements and 65% of the Ty3/*Gypsy* elements detected by the EDTA to distinct LTR lineages (Supplementary Table S11). Among these, Ty1/*Copia*/SIRE and Ty3/*Gypsy*/Tekay were identified as the most abundant lineages in the safflower genome, with copy numbers of 65,067 and 71,143, respectively. Our results are concordant with the other members of Asteraceae [16–19]. Based on the presence of complete functional domains of LTR TEs, 3,497 Ty1/*Copia* elements, and 4,716 Ty3/*Gypsy* elements were classified as intact (Supplementary Fig. S11a,b). Further, 2,195 Ty1/*Copia* and 2,879 Ty3/*Gypsy* elements were defined as autonomous owing to the presence of target site duplication (TSD) and primer binding sites (PBS). At the hierarchical level of lineages, Ty1/*Copia*/SIRE (1,742) and Ty3/*Gypsy*/Retand (2,193) exhibited the highest number of complete members. Phylogenetic tree of Ty3/*Gypsy* (Supplementary Fig. S11c) and Ty1/*Copia* (Supplementary Fig. S11d) grouped different subfamilies into distinct clades. However, Ty1/*Copia*/SIRE was divided into three clades, underlining the existing variation in this subfamily. An assessment of insertion time, revealed that 87.19% of the complete LTR-TEs were inserted within the past 1 million years, indicating that transposon activity might be one of the major drivers of genome expansion in safflower (Supplementary Fig. S11e). In-depth analysis highlighted Ty3/*Gypsy*/Tekay, Ty3/*Gypsy*/Retand, Ty3/*Gypsy*/Athila, Ty1/*Copia* /SIRE, Ty1/*Copia* /TAR, and Ty1/*Copia*/Angela as the dominant contributors to this recent transposon burst. Notably, the most recent transposon burst aligns with the recent whole-genome duplication (γ) event in safflower [13].

DNA transposons (class II elements) constituted around 17.63% of the total repetitive elements (Supplementary Table S10, Supplementary Fig. S10). DNA transposons were further classified into Tandem inverted repeats (TIR; 7.13%), Miniature inverted-repeat transposable element

(MITES; 7.61%) and Helitrons (2.88%) (Supplementary Table S10, Supplementary Fig. S10). Simple Sequence Repeats (SSRs) constituted 0.38% of the total repeats (Fig. 1d).

Non-coding RNA (tRNA and rRNA) genes were also surveyed in the Safflower_A2 genome assembly. We identified 4,763 rRNA genes comprising 3,619 5S type, 398 5.8S type, 377 18S type, and 370 28S type. Additionally, 1,110 tRNA genes were predicted, of which 766 coded for 20 amino acids (Supplementary Table S12).

Annotation of protein-coding genes

The safflower genome harboured a total number of 59,995 transcripts with an average of ~4.4 exons per transcript and at an average intergenic distance of ~14 kb, identified by evidence from homology and RNA-seq methods (Fig. 1c, Table 1). After clustering through cd-hit, these 59,995 transcripts corresponded to 39,945 unigene models (at 80% similarity). The derived transcript set yielded a combined BUSCO score of 91.5%, indicating completeness of the safflower gene repertoire (Supplementary Table S13). Collectively, ~80% (47,704) of the predicted protein coding transcripts were annotated with at least one functional term using publicly available databases RefSeq, Gene Ontology (GO), Enzyme Code (EC), Cluster of Orthologous Groups (KOG), Kyoto Encyclopaedia of Genes and Genomes (KEGG) and Interproscan (Fig. 2 a-d, Supplementary Table S14). In addition, we could delineate a total of 2,893 transcription factors and regulators. We also identified 1,587 protein kinases. .

Identification of Resistance Gene Analogs (RGAs)

To facilitate genetic dissection of disease resistance in safflower, we determined a total of 2,461 putative genes encoding for RGAs, that were categorized into 24 major classes based on their constituent domains (Fig. 3a, Supplementary Table S15). Among the RGAs, the most characterized and well-known gene family for disease resistance in plants is the Nucleotide-binding-site Leucine-rich repeat Receptor (NLR) gene family [20], which includes Toll/interleukin-1 receptor-nucleotide-binding site-Leucine-rich repeat (TNL), Coiled-coil-Nucleotide-binding site- Leucine-rich repeat (CNL), and resistance to the powdery mildew RPW8-NBS-LRR (RNL) genes. We identified 228 non-redundant, high-confidence NLR genes encoding for 236 NLR transcripts in the safflower genome of which 191, 38, and 7 members encode TNL, CNL, and RNL genes, respectively. The localization of the NLRs on different safflower chromosomes exhibited biased distribution with chromosomes 2, 6, and 11 encompassing high proportions of NLRs while no NLRs were found on chromosomes 5 and 7 (Fig. 3b, Supplementary Fig. S12). Physically, the NLRs within each chromosome were present in multigene clusters near the telomeric region (Supplementary Fig. S12). Among the NLR genes, 75 closely related homologous gene pairs were identified with an average Ka/Ks value of 0.49, 0.52, and 0.33, for TNL, CNL and RNL respectively (Fig. 3b). All the NLR genes showed negative selection (Ka/Ks < 1), however, two gene pairs also indicated positive selection (Ka/Ks > 1). We delineated several types of duplication events including tandem (163), proximal (50), dispersed (11), and segmental (12), which accounted for ~70%, 21%, 4.6%, and 5% of all NLR genes, respectively.

We also predicted NLR genes in genomes of *Arabidopsis thaliana* [21] and *Helianthus annuus* (sunflower) [22] identifying 284 and 202 NLR genes respectively. Phylogenetic analysis was performed incorporating the NLR genes from all the three species. We detected three distinct clades: TNL, CNL, and RNL, supported by high bootstrap values (>75%) (Fig. 3e). Among these

clades, NLR genes from safflower and sunflower clustered together, whereas *Arabidopsis thaliana* genes were placed in a divergent clade. The collinearity analysis between safflower and *Arabidopsis* identified 144 collinear genes distributed across 30 syntenic blocks (Supplementary Fig. S13a). In contrast, the analysis between safflower and sunflower revealed 66 collinear genes within 11 syntenic blocks (Supplementary Fig. S13b).

Out of the 236 NLRs, we were able to assign the function to the 214 NLRs based on its functional annotation. Annotation of these NLRs identified proteins such as *TMV-N-like*, ROQ1, RPP13, RRS1, RUN1, and DSC1. These proteins have been shown to play significant roles in conferring resistance against a wide range of pathogens, including bacterial, viral, and fungal pathogens in different plant species (Supplementary Table S16). These NLRs might provide resistance/ tolerance against the major threats such as wilt, root rots, and leaf blight. The incorporation of these resistance genes into safflower breeding programs could facilitate the development of robust, disease-resistant cultivars.

Exploring genetic basis of various agronomically important traits in safflower

A total of 2.05 Tb of resequencing data was generated for 123 accessions with an average coverage of 15.3x per accession (Supplementary Table S17). Variant calling of the safflower core collection identified ~13 million raw SNPs and ~2.3 million small indels. We obtained a final set of ~1.8 million SNPs after robust filtering (Supplementary Table S18; Supplementary Table 19).

The population structure analysis of core collection identified four major clusters (K=4; membership coefficient (qi) ≥0.5)) designated as ADI, ADII, ADIII and ADIV and comprising 62, 6, 19 and 16 accessions, respectively (Supplementary Table S20). ADI comprised

large number of accessions from different continents while ADIII delimited primarily Indian accessions (Fig. 4a). Principal component analysis (PCA) was performed and the first two principal axes, PC1 (18%) and PC2 (13%) were plotted (Supplementary Fig. S14a). All accessions from ADI clustered together in quadrants 2 and 3 of PCA. Quadrant 3 comprised accessions from USA while quadrant 2 consisted of accessions from other regional gene pools. All accessions from ADIII clustered in quadrant 4 whereas ADII and ADIV accessions were clustered in quadrant 1. Based on the phylogenetic tree, four major clusters (NJI – NJIV) were observed (Fig. 4b). Most ADI accessions clustered together in NJI. ADII accessions, along with some ADI accessions, were in NJII. NJIII comprised accessions from ADIII while NJIV had accessions from ADIV and ADI. Our population genetic structure analysis based on ADMIXTURE, PCA and phylogenetic analysis was able to infer consistent phylogenetic relationships between the accessions. F_{st} divergence was estimated between the populations. AD I and AD III showed high genetic divergence (F_{st} = 0.45) whereas AD II and AD IV showed minimum genetic divergence (F_{st} = 0.239). Linkage decay (LD) of the re-sequenced accessions indicated that the LD decreases to half (r^2 = 0.15) from its maximum at ~6 kb (Supplementary Fig. S14b). The overall LD is similar for all chromosomes ranging from 0.15 to 0.10 which is in consonance with earlier reports on sunflower [23].

Phenotypic data for all traits represented in the core collection is sourced from our earlier study [14], which has been shown to have broad spectrum of variability with a normal distribution. Pearson's correlation analysis demonstrated strong concordance (>90%) in phenotypic data across two growing seasons for traits including oil content (OC), plant height (PH), and days to 50% flowering (DTF). However, for traits like 100-seed weight (100SW), the number of primary branches (PB), and the number of heads (HN), the correlation was moderate (0.61 to 0.81). Phenotypic data for Oleic and Linoleic acid content (OA-LA) was available for only one growing

season, and therefore, seasonal concordance could not be assessed for these traits. SNPs (~1.8 million) generated were further filtered to a final set of 320,399 (filtering criteria summarized in Supplementary Table S21 and SNP distribution in Supplementary Table S22 and Fig. 4c). We identified 3,159 significant Marker Trait Associations (MTAs) collectively for all eight agronomic traits over two growing seasons at $p<0.0001$. The population structure analysis indicated 4 subpopulations in the core collection; hence the analysis was carried out keeping number of Principal Component (PCs) as 4. QQ plots were analysed to identify the best fitting models for each trait and the analysis revealed that multi-locus models were best suited for detecting significant associations. Only the MTAs which were following specific criteria were retained (outlined below) and called as Quantitative Trait Nucleotides (QTNs). For traits OC, PH, and DTF, the QTNs were consistently identified across all three multi-locus models in both growing seasons. In contrast, traits HN, PB, and 100SW showed more seasonal variability, hence QTNs were defined as those present in at least one multi-locus model and both growing seasons. Oleic acid (OA) and linoleic acid (LA) data were available for only one growing season; thus, QTNs for these traits were identified using the best-fitting model, MLMM. A total of 96 QTNs were identified for eight traits on all chromosomes (Fig. 4d, Supplementary Table S23).

LD block analysis revealed that the average size of the LD block in the QTN region is ~ 6.7 kb (Supplementary Table S24). Consequently, candidate genes were searched within 7 kb upstream and downstream of the QTN positions. A total of 32 candidate genes corresponding to 33 QTNs were identified across all eight traits based on their putative functions reported in the literature. Haplotypes were identified in the LD block for QTNs with putative functions, and their associations with the phenotypes were further analysed (Table 2, Supplementary fig. S15).

Selected QTNs were subsequently validated using KASP assays (Supplementary Table S25, Supplementary Table S26), providing additional support for the genetic associations identified.

Being an oil crop, OC is the most important trait for safflower. Three QTNs OC1, OC8 and OC13 (Fig. 5a, b) marked the gene BIG GRAIN 1-like protein, a positive regulator of auxin transport and signalling, reported to control grain size in rice by modulating cell division [24]. Interestingly, we found OC1, OC8, OC13 and BIG GRAIN within the same haploblock (Fig. 5c). OC8_H01 is the most geographically distributed whereas OC8_H04 is the least represented haplotype (Fig. 5d). OC8_H05, and OC8_H06 encode for moderate to high oil (>25%) (Fig. 5e-f, Supplementary fig. S15 a,b). KASP assay validated QTNs OC1 and OC8 along with four SNP sites (76395211,7639615,76395777,76397432) which were1.5 kb upstream to the BIG GRAIN 1-like gene. Another QTN, OC12 lies in the vicinity of a gene encoding myosin-binding protein (MYOB2). MYOB2, is known to be a lipid droplet (LD)-associated protein in Arabidopsis leaves, which may be involved in enhancing lipid transport and storage [25]. OC12_H02 is the haplotype associated with high oil (30% to 55%) (Supplementary fig. S15 c) and henceforth, validated using the KASP assay.

For trait 100SW, QTN SW23 is downstream of a CYP57 isoform, known to regulate cell division and elongation processes that influence seed size and weight in Arabidopsis [26]. Another QTN SW3 was in the regulatory region of the xyloglucan galactosyltransferase gene, involved in hemicellulose modification of primary cell walls of most dicotyledonous plants [27]. Haplotype analysis detected that SW23_H02 is associated with high seed weight, whereas SW3_H02 with low to moderate seed weight (2.5g-5g) (Supplementary fig. S15 d,e). A KASP panel of eight accessions validated the presence of QTN SW23 and the two associated SNPs with the gene. QTN SW31 lies in the upstream region of the oleosin-B6-like protein, which is associated with lipid

droplet stability and oil body formation in seeds, potentially playing a role in seed weight and energy storage [28]. Another QTN SW10 is located downstream of the gene encoding RNA-binding protein 2 (RBP) implicated in post-transcriptional regulation RBPs such as APUM24 are known regulators of seed development [29] (Supplementary fig. S15 f,,g). QTN SW39 was associated with the OFP9 transcriptional repressor protein, known to regulate grain size in rice through hormonal modulation [30]. QTN SW10 and SW39 were verified by KASP analysis.

For oleic and linoleic acid, we detected six QTNs (OA_LA15, OA_LA17, OA_LA20, OA_LA33, OA_LA45 and OA_LA44) on chromosome 5 associated with cytochrome P450 71A4-like, which is involved in fatty acid catabolism via epoxidation [31]. Haplo-pheno analysis (Supplementary fig. S15 h,i,j,k) suggests that QTNs associated with cytochrome P450 71A4-like resulted in low to moderate OA, however no correlation was detected with LA. It is possible that FAD and cytochrome P450 71A4 are competing for the same substrate (oleic acid), however, further studies are required to explore this hypothesis [32]. KASP analysis validated the presence of OA_LA17 in the panel comprising both high and low linoleic acid lines. Haplopheno analysis of QTN OA_LA20 identified a haplotype (OA_LA20_H002) which was associated with low oleic acid content (Supplementary Fig. S15 j). Two SNPs were identified which might be playing a significant role in regulating fatty acid content in safflower. Both SNPs were validated by KASP analysis.

For DTF, we identified QTN DTF2, associated with the gene encoding E3 ubiquitin-protein ligase UPL1-like, known to regulate various developmental processes, including flowering [33] . QTN DTF10 was found to be in the proximity of E3 ubiquitin-protein ligase COP1-like, which accelerates the degradation of GIGANTEA (GI) via the 26S proteasome, thereby delaying flowering under low-temperature conditions [34]. Haplotype analysis identified DTF2_H05 and

DTF2_H06, associated with a reduction in number of days to flowering whereas DTF10_H02 and DTF10_H03 were responsible for increasing DTF (Supplementary fig. S15 l, m). Validation of DTF2 and one corresponding SNP was done using KASP, lending further support to our analysis.

For pre-harvest traits such as PH, QTN PH3 (on chromosome 2) is downstream of the gene encoding a vignain-like protein, a cysteine protease, involved in resource allocation or tissue remodelling, which are critical during active growth [35]. For PH3, we detected two haplotypes PH3_H02, which led to the increased height in season 1 but not in season 2 (Supplementary fig. S15 n,o). QTN PH4 (validated using the KASP) lies in the vicinity of the gene coding for trafficking protein particle complex subunit 6B, a component of the transport protein particle (TRAPP) complex, which is involved in vesicle transport, important for plant growth, and development [36] .

For PB, QTN PB14 is located near TF Teosinte branched1/Cycloidea/Proliferating cell factor T (TCP20), which modulate plant development by influencing hormonal pathways, including brassinosteroid biosynthesis which is closely linked to branching and shoot architecture [37,38]. Another QTN, PB9 lies upstream of a gene encoding F-box/LRR-repeat protein 14, involved in auxin signalling pathway and programmed cell death. Haplotype analysis revealed that PB14_H02 shows an association with low number of primary branches (Supplementary fig. S15p). QTN PB14 and PB9 were validated using the KASP analysis.

For HN, QTN HN5 lies upstream of the gene coding for alpha-xylosidase 1, critical for maintaining cell wall integrity and enhanced cell wall loosening in the elongating flower stem, however only one major haplotype was detected [39].

Pan-genome assembly, annotation, and Presence Absence Variation (PAV)analysis

Using the three chromosomal-level safflower assemblies (this study, [12,13]) and accessions from core collection (Fig 6a), we assembled a pan-genome of safflower of 1.26 Gb (henceforth called Safpg_v1) including an additional 99.9 Mb, which represents an increase of 8.9% over the reference genome. This additional sequence comprised 63,814 contigs with a length range from 1 kb to 47.85 kb. We predicted 11,479 transcripts, which increased the total number of predicted transcripts of the safflower genome to 71470 (size> 150 bp). Functional annotation of the newly added transcripts against the Refseq database, assigned functions to ~5383 transcripts. The enrichment of variable genes was mainly detected in the categories *viz*. regulation of biological process, response to stimulus, catalytic and binding as well as disease resistance. GO enrichment analysis of annotated novel transcripts revealed significant enrichment in biological processes related to stress response (Fig 6b). We detected 136 and 103 enriched transcripts for biotic and abiotic stresses, respectively. Furthermore, domains were identified through InterProScan exhibiting domains related to protein kinases, zinc-finger, reverse transcriptase, disease resistance gene, HEAT repeat domains and Leucine rich repeats. KEGG pathway analysis identified key pathways including linoleic acid metabolism, galactose metabolism, and ABC transporter systems These enrichment results indicated that these additional genes were largely involved in metabolic processes, helping the plant in combating biotic and abiotic stresses [40].

The breeding history and genetic changes of the crop can be revealed in the Presence-Absence Variation (PAV) among different accessions. We identified 14,542 cores, 33,184 soft cores, 20,699 shell, 1,452 cloud, and 214 private genes (Fig 6c) through the PAV matrix ((Fig 6d). All the core and soft core were assigned as conserved (47,726) whereas shell, cloud and private genes

were assigned as variable (22371) genes. Modelling of the pan-genome showed that the number of core genes remain constant. However, the number of pan-genes continues to increase with the addition of the new genome, suggesting that saturation has not yet been achieved (Supplementary Fig. S16a). We detected larger gene length and more exons in the core genes as compared to variable genes, showing the conserved nature and long evolutionary history of core genes, and comparatively a new origin of the variable genes. The maximum number of the genes were contributed by NC132, an accession from the USA. The largest number of the variable genes was harboured by CC62, a Russian accession. The maximum number of shell genes was present in accession CC25 from India. Interestingly, an Indian accession, CC38 tends to be the most diverse accession of the core collection with 1,326 cloud genes and 203 private genes. CC108 (USA), CC72 (Spain), CC51 (Iran) and NC132 (USA) are also diverse accessions based on cloud genes (Supplementary table S27). The number of core and soft-core genes is similar among the regional pools. The highest number of variable genes was seen in accessions of the Indian subcontinent and USA depicting their high genetic diversity (Supplementary Fig. S16b). Out of 103 new abiotic resistance genes, 55 were found across all 13 regional gene pools, and 43 were present in at least 11 of them. For biotic stress, 56 out of 135 enriched transcripts were seen in all 13gene pools, while 65 were present in at least 10 genepools (Supplementary table S27 and S28). These pan-genomic genes could be used in for regional breeding programs

Discussion

In the present study, we generated an improved safflower genome assembly, which was validated with the help of a GBS-based linkage map. This reference assembly was used for calling SNPs

from core collection resequencing data. GWAS, candidate gene analysis and haplopheno analysis was conducted leading to identification of genomic regions associated with important safflower agronomic traits. The resequencing data was also used to identify a pan-genome for the crop.

Improved quality of the Safflower_A2 genome assembly

We report an improved, contiguous, and richly annotated genome sequence of a safflower accession (Safflower_A2) with high oil and nutritionally desirable high oleic acid content. The two earlier reported chromosomal-level genome assemblies were derived from Chinese safflower accessions, Anhui [12] and Chuanhonghua 1 [13], which were rich in the linoleic acid. K-mer analysis (K=17) of our genome demonstrated a genome size of 1.17 Gb with low heterozygosity and high repeat content, which is in consonance with earlier studies [12,13]. The estimated genome size through flow cytometry was 1.34 Gb, which was 8.7% higher than k-mer distribution analysis. A lower estimation by k-mer analysis could be attributed to the high amount of repeat sequences in the safflower genome [41]. The genome assembly of Safflower_A2 is 1.15 Gb which is better than the Anhui 1 genome assembly (1.07 Gb) and similar to the Chuanhonghua 1 (1.17Gb). As mentioned earlier, Safflower_A2 and Anhui 1 genome revealed one-to-one alignment (Supplementary fig. S7a). However, Safflower_A2 chromosome lengths were significantly longer than those of Anhui 1 (Supplementary Fig. S17a, S17b). Safflower_A2 genome also showed higher completeness with a significantly higher BUSCO score (97.9%) as compared to Anhui 1 (90%) and Chuanhonghua 1 (89.25%) genomes. A higher rate of mapping back of raw long reads of Safflower_A2 genome (99.29%) as compared to Anhui 1 (98%) and Chuanhonghua 1 (93%) genomes further supports superior quality of the safflower_A2 genome (Table 1). Although the

primary genome assembly of Chuanhonghua 1 was reportedly higher at 1.17 Gb, our analysis shows the fragmented nature of this genome (explained by indices described below), hence, comparison of chromosomal lengths could not be accurately conducted as we could not correlate chromosomes between the two genomes (Supplementary fig. S7b). During our structural variant analysis, we detected the presence of large translocations in the Chuanhonghua 1 genome (particularly on chromosome 1), which further suggests possible mis-assembly or unresolved scaffolding errors during genome construction (Supplementary fig. S7c, d). Although, such large-scale structural rearrangements could be due to true biological divergence, their absence in the Anhui 1 genome indicates that the translocations are likely assembly artefacts**.** We have validated our genome assembly using a high-density linkage map constructed using GBS data from a RIL population. A high concordance between the genome assembly and the linkage map confirms the accuracy and completeness of our genome assembly. As compared to earlier studies, we detected higher number of LTRs in our genome, which is supported by a high LAI score (22.49) that is comparable to that of the Anhui 1 genome (23.08) and significantly higher than that of Chuanhonghua 1 genome (14.65; Table 1). Further, we detected telomeric repeats at both ends of three chromosomes and one end of nine chromosomes which indicates near completeness of the Safflower_A2 genome assembly. We also detected centromeric repeats of four different lengths (342 bp, 348 bp, 349 bp and 350 bp) on all the chromosomes representing resolution of the repetitive regions of the genome. Centromeric and telomeric regions are being reported for the first time in the Safflower_A2 genome. We detected 59,995 protein coding transcripts corresponding to 39,945 Unigene models (at 80% similarity) after clustering. A higher number of unigenes can be attributed to improved quality of the safflower_A2 genome and use of BRAKER3 pipeline in the current study which outperforms MAKER2 (used in earlier studies) in prediction of exons,

genes and transcripts [42]. Use of a comprehensive Iso-Seq dataset as evidence for annotation, improved the BUSCO score of predicted protein sequences (Table 1). Furthermore, our results are in consonance with the recent reports on other plant species including *Eriobotrya japonica* [43] and *Lonicera caerulea* [39].

Identification of genes associated with disease resistance

The generation of a repertoire of R genes and their analysis is important to facilitate breeding for resistance to biotic stresses. A comprehensive analysis of R genes in the safflower genome is lacking. We identified 228 non-redundant genes, coding for 236 NLRs transcript in the safflower genome, which would expedite the cloning of resistance genes and enhance our understanding of their associated mechanisms [45]. The protein coded by an NLR gene consists of the NBS domain, which hydrolyses energy, and the LRR domain for pathogen recognition [46]. The TIR domain is involved in the downstream signalling involving Enhanced Disease Susceptibility 1 (EDS1) protein, providing immunity against biotic and hemi-biotic pathogens, exhibiting both local and systematic immunity. In contrast, non-TNL domains (CNL and RNL) are involved in NPR1-mediated immunity, contributing to broad-spectrum systemic immunity in plants [47]. In safflower, the ratio of TNL and non-TNL is ~4.25, suggesting the higher contribution of TNL in the disease resistance of safflower. The number of TNL-RGAs is higher than non-TNL RGAs in safflower, which is in contrast with sunflower, wherein non-TNL genes were higher (~0.85) [48]. However, our observations are in consonance with those of Arabidopsis (~4.26) as observed in the earlier studies also [13]. To understand the evolutionary history, we conducted a phylogenetic analysis by incorporating NLR genes from the model plant *Arabidopsis thaliana* and sunflower

(*Helianthus annuus*). Phylogenetic analysis classified NLR genes into three distinct clades: TNL, CNL, and RNL indicating ancient divergence and functional specialization within the NLR gene families (Fig. 3d). Among these clades, NLRs from safflower and sunflower clustered together, representing a lineage-specific expansion within the Asteraceae family. In contrast, *Arabidopsis thaliana* genes were placed in a divergent clade, highlighting both conserved and divergent evolutionary patterns across species. Additionally, large branch lengths in the TNL and CNL clades across all species suggest dynamic evolution, likely driven by co-evolution with pathogens. In contrast, the shorter branch lengths of the RNL genes suggest their role as helper components downstream to the immune signalling pathway [49]. Furthermore, TNL and CNL genes show early divergence, while RNL genes represent more recent divergence. The collinearity analysis revealed a higher number of colinear blocks between safflower and *Arabidopsis* as compared to safflower and sunflower, which could be attributed to the greater number of annotated R genes in the *Arabidopsis thaliana* genome compared to the sunflower genome. Additionally, the TNL to non-TNL ratio in both safflower and *Arabidopsis thaliana* is similar (approximately 4.2), while in sunflower it is around 0.85. The variation in the TNL to non-TNL ratio between safflower and sunflower genome can be explained by a whole-genome duplication event that occurred after the divergence of the two species [13]. This lower ratio of TNLs in sunflower may contribute to the reduced number of colinear blocks observed.

The functional annotation of R genes in safflower has unveiled a repertoire of proteins integral to the plant's defence mechanisms against a spectrum of pathogens (Supplementary Table S16). Notably, proteins such as TMV resistance protein-N-like, ROQ1, RPP13, RRS1, RUN1, and DSC1 have been identified to confer resistance against viral, bacterial and fungal pathogens including *Xanthomonas*, *Pseudomonas*, *Ralstonia*, *Puccinia*, and *Verticillium* species respectively

[50–58]. For instance, ROQ1 recognizes bacterial effectors like XopQ and HopQ1, activating defence responses that are crucial for resistance, as shown in crops like tomato and *Nicotiana benthamiana* [50–52]. Similarly, RPP13 has been shown to contribute to basal resistance in wheat by interacting with pathogen effectors, thereby restricting the development of diseases such as powdery mildew [53,59]. The At4g11170 gene encodes a Resistance Methylated Protein (RMP) that has been associated with resistance to *Fusarium oxysporum* [60], a significant pathogen in safflower cultivation. In addition to these, genes like NRG1.1 [61], RPP8 [62–64], At4g11170 [60,65], and RGA3 [49,66–69], have been implicated in defence against pathogens such as *Alternaria*, *Fusarium*, *Phytophthora*, *Potyvirus*, and *Golovinomyces* respectively. Incorporating these R genes into safflower breeding strategies through marker-assisted selection can expedite the development of resistant cultivars. Furthermore, pyramiding multiple R genes conferring resistance to different pathogens, can enhance the durability and breadth of disease resistance. This approach mitigates the risk of resistance breakdown due to pathogen evolution and provides a sustainable solution for disease management in safflower.

Exploring the genetic basis of various agronomically important traits in safflower

To decipher the full repertoire of genes available in a crop, the availability of sequence data from diverse accessions is important. However, due to the significantly large diversity harboured by safflower, resequencing of a core collection is more practical, cost-effective and timesaving. Thus, we re-sequenced a core collection comprising 123 accessions developed earlier by our group [14]. This core collection was developed through maximization strategy from a germplasm collection of 531 accessions representing the global genetic, morphological and geographical diversity

available for safflower. In the earlier study by [13], a collection of 220 accessions was re-sequenced. However, this collection mainly consisted of accessions of Chinese origin (149) with a limited representation of global genetic diversity. Through ADMIXTURE analysis, we predicted four hypothetical sub-populations and identified nineteen admixtures in our population. Our earlier study [70] based on SSR data indicated two clusters (highest peak at K=2 and a smaller peak at K=4) and 16 admixtures. Use of a larger SNP dataset in the current study increased the resolution. We further inferred genetic relationships among accessions using distance-based methods, NJ and PCA, and observed its concordance with Bayesian based ADMIXTURE methods. All accessions from ADI clustered together in quadrants 2 and 3 of PCA. Quadrant 3 comprised accessions from USA while quadrant 2 consisted of accessions from other regional gene pools. All accessions from ADIII clustered in quadrant 4 whereas ADII and ADIV accessions were clustered in quadrant 1. Most ADI accessions clustered together in NJI. ADII accessions, along with some ADI accessions, were in NJII. NJIII comprised accessions from ADIII while NJIV had accessions from ADIV and ADI. We observed lack of geographical structuring among accessions of the core collection which could be attributed to maximization strategy used for core collection development, emphasising allelic diversity with minimum redundancy [14]. Further, low F_{st} and kinship value between the sub-populations represents the low differentiation and low genetic similarity between the sub-populations. Thus, low molecular relatedness and weak population structure among the core collection accessions reduce the likelihood of false marker-trait associations [49,50], affirming its appropriateness for association mapping.

GWAS is a powerful tool in crop genetics, providing comprehensive insights into the genetic basis of complex traits, accelerating breeding programs by eliminating the need of developing biparental populations [71]. In the current study, five different models were tested and three multi-locus

models (MLMM, FarmCPU and BLINK) were utilized to detect significant MTAs. The multi-locus models demonstrate robustness as they consider multiple loci, hence reducing the false positive. In our data, multi-locus models fared better than single-locus models as indicated by QQ plots. MLMM is effective in identifying large effect loci, while FarmCPU can detect small effect loci [72,73]. Although FarmCPU has reduced rate of false positives than MLMM, it is still prone to errors. In comparison, BLINK includes LD information in the analysis [74], hence performs better for environmentally sensitive traits as we observed in HN, PB and 100SW. Since all three models have their strength and weaknesses, we analysed QQ plots to ensure that the model used is not overfitting and an appropriate model has been used (explained in result section). Further, we ensured robustness of markers by comparing the analysis of two seasons data and retaining markers which were identified in both seasons. We report a total of 96 QTNs, wherein 22 QTNs were identified for OC, 7 for PH, 16 for OA and LA, 21 for SW, 4 for HN, 14 for PB and 12 for DTF (Supplementary table S23). In a previous study [13], QTNs have been reported for oil content, flower colour, ball (head) number, branch height, bract spine, first branch number, plant height, and stem diameter. While QTNs reported in the study show strong correlations with traits, EMMA (Efficient Mixed-Model Association) model for detection of MTAs is computationally less robust compared to MLMM, FarmCPU and BLINK used in the current study, especially for large datasets. Thus, by leveraging a multi-model GWAS approach, phenotypic data from 2 consecutive growing seasons, and a globally diverse germplasm collection, our study provides more robust QTNs. The QTN-marked regions were subjected to candidate gene analysis, identifying genes invaluable for breeding programs that could lead to developing varieties with improved oil content and cultivars optimized for specific growing environments. We identified several candidate genes associated with key agronomic traits, including OC, PH, DTF, OA, LA,

100SW, PB, and HN as detailed in Table 2. These candidate genes could also be used for genome editing approaches, although further validation is required. Similar approaches have been employed in apple [75], rice [76] and soyabean [77]. To increase the confidence in the identified candidate genes, we performed haplotype analysis. A similar approach has been employed for the detection of haplotypes associated with various agronomically important traits [78,79]. We detected robust haplotypes exhibiting significant variation in the core collection for OC, SW, DTF, PB, OA, and LA. However, only one or two major haplotypes were identified for PH, and HN, highlighting their complexity due to strong interactions with environmental conditions. We observed many SNPs (ranging from 11-40) within each haplotype. The number of haplotypes ranged from 11 to 83, with OC, DTF, SW, OA, and LA having more than 50 haplotypes, indicating presence of diverse SNP combinations present in germplasm. Interestingly, we found that putative candidate genes were in proximity (within 2 Kb) of the haplotypes, suggesting that these SNP groups may play crucial roles in gene function. In this study, we identified favourable haplotypes, which are prevalent in large populations and encode a wide range of trait values and likely selected through evolution [80,81]. However, we also detected superior rare haplotypes, contributing to exceptional agronomical trait values. For superior OC, we identified three accessions—CC106, CC101, and CC090—characterized by high oil content coupled with superior alleles for OA or LA. However, these accessions had favourable haplotypes for other traits. The information generated can be used to create varieties with multiple superior haplotypes using approaches like pyramid breeding [82].

In crop improvement programs, functional markers (FMs) are instrumental in enabling precise selection for desirable traits. The development of high-throughput and cost-effective genotyping platforms, such as KASP , has significantly enhanced the application of marker-assisted selection

by allowing rapid screening of large breeding populations. In this study, we developed KASP assays targeting 20 SNP loci (Supplementary Table S25, S26) associated with seven key agronomic traits. Based on phenotypic trait values, panels were constructed, and marker functionality was assessed across diverse genetic backgrounds. Among the 20 SNPs developed and validated in the present study, 10 were QTNs, including traits such as OC (OC1, OC8, OC12), OA and LA (OA_LA17), 100SW (SW_23), DTF (DTF2), PH(PH_4), and PB (PB14, PB18). Furthermore, 10 SNPs were selected based on their proximity to candidate genes involved in trait regulation, including those associated with oil content (4), fatty acid content (2), seed weight (3), and flowering time (1). Our study developed a KASP assay for the SNPs closer to genes (Supplementary Table 26, Supplementary Table 26) known to play an important role in various agronomic traits. These KASP markers provide a rapid and reliable tool for marker-assisted selection, enabling efficient integration of favourable alleles into elite cultivars. The validation of representative QTNs and their associated SNPs with candidate genes reinforces the reliability and robustness of our bioinformatic dataset. Present integrative bioinformatic analyses identified key candidate genes, laying the groundwork for future experimental studies. Future research should aim to integrate transcriptomic data for expression-based correlation, functional characterization through approaches such as genome editing, gene knockout, overexpression, or allelic function analysis. Nonetheless, the information generated in current analysis is invaluable for safflower breeding programs.

Pan-genome analysis reveals distinct functional enrichments among pan-genes

A single reference genome cannot encompass the entire genetic variability present in a species like *C. tinctorius*, which has gone through extensive diversification [83]. Pan-genomes developed from

diverse individuals act as an important resource to capture the available genetic variability and mining of alleles. In recent years, pan-genomes have been constructed for various plant species [83–86]. We constructed the pan-genome of safflower using unmapped reads of 123 accessions of our core collection through iterative mapping and assembly approach. The newly assembled sequence includes those regions that were absent in the reference genome and thus, acts as an extended repertoire of available genes for the crop. Gene Ontology (GO) enrichment analysis of annotated novel transcripts revealed significant enrichment in biological processes related to stress response. Abiotic stress-related gene (103 transcripts) included peroxidases, DELLA proteins, DNA repair proteins, E3 ubiquitin-protein ligases, heat shock proteins, and TIFY transcription factors, indicating their potential role in enhancing safflower's tolerance to environmental stresses. In contrast, transcripts associated with biotic stress were found to encode a range of resistance proteins, including RML1A-like, RGA-3, RPP-13, RGA-4, R1A-10, R1B-14, RML1B-like, thaumatin-like proteins, BTB/POZ domain proteins, ankyrin repeat-containing proteins, and TMV-resistance genes. Identification of thaumatin-like proteins [87–89] is crucial as it has been linked to resistance against Fusarium wilt, which is a major disease for safflower (Supplementary Table S16). In terms of molecular function, the novel transcripts were predominantly enriched in binding activities, especially ion binding, small molecule binding, and nucleic acid binding. Cellular component analysis indicated their localization to membrane-associated structures such as the plasma membrane, nuclear lumen, cell junctions, and the U2-type spliceosome complex. Domain analysis further revealed features such as protein kinases, LRR domains, P-loop NTPase, zinc-finger motifs, and HEAT repeat domains, suggesting roles in signal transduction and stress response. Enriched domains like GIP-1 (G-protein interacting protein), heavy metal-associated (HMA) motifs, transient receptor potential (TRP) channels, and Lipoxygenase PLAT domains

highlight potential involvement in detoxification, ion transport, and lipid metabolism under stress [35].

We performed PAV analysis using the map-to-pan approach and identified core genes that are common across multiple genomes and define the species, as well as variable genes found in only a few genomes, which contribute to the unique characteristics of each genome. We further analysed core and variable genes among regional gene pools. The regional gene pools of safflower, *i.e.*, USA, India, Far-east and Europe comprise accessions (Supplementary Table S27,S28) with large numbers of variable genes and thus, exhibit high genetic diversity that might have arisen during diversification of safflower. In our study, the Indian accession, CC38, was found to be the most diverse accession of the core collection consisting of a large number of cloud and private genes. PAV analysis of stress-related transcripts showed peroxidases and heat-shock proteins common across accessions and gene pools. However, genes like TIFY-8 and WD repeat-containing protein76 which were present in only 30 and 83 accessions, respectively. Among all the accessions, distribution of stress related genes showed 96 genes in CC30 (Indian subcontinent), 94 genes in CC47 (Iran-Afghanistan), and 91 genes in CC62 (Europe), suggesting that these gene pools are most diverse (Supplementary Table 28). The USA accession CC86, with only 64 genes indicated that this gene pool is the least diverse. For biotic stress, TMV resistance protein N-like, L-type lectin-domain containing receptor kinase S, and DSC were found in most accessions. The Indian subcontinent accession (CC56) showed the highest number of biotic resistance genes, while the USA accession carried the fewest. Overall, the inclusion of novel gene content from the safflower pan-genome highlights substantial genetic diversity, which likely contributed to the species' adaptive capacity and provides resources for regional breeding programs.

Methods

Plant material

For generating genome assembly, a safflower accession from USA (PI:560169; USDA, USA; named as "Safflower_A2") attributed with substantially high seed oil content (~47%) and naturally enriched with high oleic acid (~87%) was selected. Safflower core collection reported earlier by our group [14] comprising 116 globally distributed accessions and 7 additional accessions (Supplementary table 17) with agronomically important traits were subjected to Illumina-based resequencing (~15x coverage) using Novaseq sequencer.

Genome sequencing and assembly

HiFi long read sequencing was performed on PacBio Sequel II platform following manufacturer's instructions (PacBio, California). A Bionano Saphyr chip (Bionano genomics, California, USA) was used for generating optical mapping data. The Proximo Hi-C (Plant) Kit Protocol (Phase genomics, USA) was used to construct Hi-C library (Phase genomics, USA). The Hi-C libraries were sequenced on Illumina Novaseq 6000 (Illumina, USA).

Genome size was estimated through two approaches: flow cytometry and k-mer frequency distribution. For flow cytometry, CyStain PI Absolute P kit (Sysmex, Germany) was used for sample preparation following manufacturer's instructions. A minimum of 5,000 stained nuclei per sample were evaluated on CyFlow Cube 8 flow cytometer (Sysmex, Germany) using tomato 'Stupicke´ polnı´ rane´' as a reference [90]. K-mer-based genome size estimation was performed using clean HiFi reads from PacBio SMRT sequencing applying Kmerfreq [91] and GCE v1.02 [92] .

Long PacBio HiFi reads were assembled into contigs using Hifiasm v.0.16 [93]. Due to homozygous nature of safflower genome, purging was disabled (-l0) and other parameters were applied at default settings. Using optical maps and contig-level assembly, hybrid scaffolding was performed using Bionano Solve v3.6 with default parameters. Scaffold-level assembly was polished using Illumina paired-end short reads through NextPolishv1.4.1 [94]. For construction of pseudochromosomes, hybrid scaffolds were linked using paired-end Hi-C reads with SALSA [95] at default settings. Additional two rounds of polishing were done using Pilon [96]. Finally, Hi-C raw reads were aligned to pseudochromosomes through BWA2 [97] to generate Hi-C heatmap using PretextMap [98] and Juicer [99] for visualisation, manual correction and generation of final chromosomal-level genome assembly. Additionally, we also assembled Chloroplast genome using Illumina short reads by Navoplasty [100] where NC_030783.1 [73] was used as a reference.

The genome assembly was validated for its integrity and completeness. Sequencing reads from Illumina and PacBio HiFi were mapped back to the assembly using bwa-mem2 v2.2 and minimap2 v2.24 [101] respectively. For estimation of base-level accuracy, Merqury v1.3 [102] was applied. BUSCO v5.6.0 [103] analysis was implemented to assess completeness of the genome. LTR_retreiver v2.9.8 [104] was used for estimation of LAI score. Telomeres were detected using Telomere identification toolkit v0.2.41 (TIDK) [105]. Centromeric repeats were detected using TRASH [106].

Present genome assembly was aligned with earlier published safflower genomes [12,13] using D-genies [107]. We further identified structural variants (deletion, insertion, translocations). Firstly, high-quality PacBio HiFi reads from the Safflower_A2 genotype were aligned to Anhui 1 and Chuanhongua1 genome assemblies using minimap2 v2.24 [101] with parameters (-ax map-hifi). The resulting BAM files were sorted and indexed using samtools v1.15.1[108]. SVIM v2.0.0 [109]

was then run in default mode to call structural variants from each alignment. Only high-confidence variants greater than 1 Kb were used for downstream analysis. SyntenyplotR [110] and KaryoplotteR [111] was used for the visualisation of the genome alignment maps.

Construction of a high-density genetic linkage map and chromosomal assignment

A RIL population comprising 121 individuals (designated as "population A"; F_8) was developed by crossing parents A1 (PI:537110) and A2 (PI:560169). The GBS library was prepared following [112] using a combination of *Mse*I and *Hae*II enzymes. Libraries were sequenced on Illumina NovaSeq 6000 platform. Filtered reads were aligned to Safflower_A2 genome and SNPs were called (Supplementary Fig. 3b). SNPs were filtered using criteria summarized in Supplementary Table S6. A high-density linkage map was constructed via JoinMap v4.1 using the Kosambi mapping function at LOD 20, followed by marker order correction and calculation of genetic distances using R/ASMap [113] at LOD 4. The linkage map was utilized for evaluating and anchoring of chromosomes using ALLMAPS [114].

Full-length transcriptome sequencing and detection of alternate splicing events

Iso-Seq sequencing was performed on RNA from eight samples [shoots, seedling-roots, leaves, flowers and buds, and seed developmental stages (at 5 DAPS, 10 DAP, 20 DAP and 30 DAP)]. Size-selected SMRTbell libraries were sequenced on PacBio Sequel II platform. Raw data was processed via SMRTlink v9.0 to generate circular consensus sequences (CCS) using default parameters. IsoSeq v3 pipeline [115] was used to obtain full-length transcripts which were collapsed into transcript clusters using pbcluster. pbmm2 [116] was used to map FL-transcripts to

repeat-masked Safflower_A2 genome using parameters --preset ISOSEQ –sort. Splicing patterns of FL transcripts were analysed using SUPPA2 [117,118].

Annotation of repeatome, gene prediction and functional annotation of protein-coding genes

Chromosomal-level genome assembly of the safflower_A2 was subjected to an Extensive De novo TE Annotator tool (EDTA v2.0.0) [119] (using parameters –anno 1, --sensitive 1) for the identification of safflower-specific *de novo* TE libraries. The in-built tools within the EDTAv2 pipelines including Repeat modeller, LTR_FINDER, LTRharvest, LTR_retreiver, HelitronScanner, MITE hunters, and TIR-Learner were used for the annotation of the transposable elements in the safflower genomes using Repbase (v20181026) libraries [119]. MISA v1.0 was used for the identification of the SSRs in our genome. Identified repeats were masked using RepeatMasker v4.1.7 [120]. The classification of LTR retrotransposons (LTR-TEs) was carried out using the Domain-based Annotation of Transposable Elements (DANTE, v0.2.5) pipeline [121]. This tool extracts information from the Viridiplantae data in the Rexdb database. Additionally, we employed DANTE_LTR [121] to identify and classify LTR-TEs as complete/autonomous i.e. consisting of complete machinery for transposition. An LTR was classified as autonomous if it contained all necessary domains, including reverse transcriptase (RT), capsid-related domain (GAG), RNase H (RH), protease (PROT), integrase (INT). Whereas, it was called complete if it contained target site duplications (TSD), and primer-binding site (PBS) along with the necessary domains. For phylogenetic analysis, amino acid sequences of the identified complete Copia and Gypsy elements were extracted to generate a multiple sequence alignment using MAFFT [122]. A phylogenetic tree was subsequently constructed with iqtree2 v2.3.0 The insertion times of the complete LTRs were estimated using LTR_retriever v2.9.8[123].

The identification of noncoding RNAs, including ribosomal RNA (rRNA) and transfer RNA (tRNA), was performed in the safflower genome assembly. Predictions of rRNA genes were conducted using barrnap v0.9, applying specific parameters for eukaryotic genomes (--kingdom euk) [124]. For tRNA gene predictions, we utilized tRNAscan-SE v2.0 [125].

Masked genome was used for gene prediction using BRAKER3 v3.0.4[42]. Mapping data generated by pbmm2 (as described above) was used as a training set for *ab initio* gene finders, AUGUSTUS [117] and GeneMark [126] for gene prediction. Transcripts <150 bases and protein sequences with <50 amino acids were removed. Subsequently, transcripts were filtered to remove those which showed a continuous repeat coverage of ≥30 %. To identify the number of unigenes, CD-HIT v4.8.1 [127] was employed using parameters -c 0.8 -n 5 -M 16000. BUSCO analysis was performed to assess completeness of filtered gene sets. Gene models were subjected to functional annotation using public nucleotide and protein databases in OmicsBox v3.1.2 [128]. Homology searches were conducted against the NCBI-RefSeq database using BLASTp with a threshold e-value of 1×10^{-3} against GO, EggNOG mapper v5 and KEGG expression database [129]. The motifs and domains-based functional annotation and identification of conserved domains and families in protein-coding genes were implemented using all public databases in InterProScan v5.6 [130]. Transcription factors/regulators and Protein Kinases were identified using iTAK v1.6 [131] with default parameters.

Identification of resistance gene analogs (RGAs)

RGAs were identified using Disease Resistance Analysis and Gene Orthology (DRAGO2) pipeline [132]. DRAGO2 classifies RGAs into Coiled-coil (CC), Receptor-like kinases (RLKs), Receptor-

like proteins (RLPs), Nucleotide binding site-leucine rich repeats (NBS-LRR) and others. Based on domain structures, NBS-LRR were further classified into CNL and TNL. HMMER software was used to classify NBS-LRR genes using the NB-ARC profile (PF00931) for the NBS domain [133]. NBS-LRR sequences were retrieved from NCBI, and BLASTp was performed. Common candidate genes identified from three analyses (HMMER, DRAGO2 and BLASTp) were retained for downstream analysis. PFAM [134] and Conserved Domains Database (CDD) [135] were used for functional domain annotation. Chromosome-wide distribution of NLRs was investigated using GFF files. To analyse NLRs under selection pressure, non-synonymous substitution to synonymous substitution (Ka/Ks) ratio was calculated using KaKs calculator 2.0 [131]. For inferring the evolutionary/phylogenetic history, NLR genes were also identified from the genome of *Helianthus annuus* (OXS) [22] and *Arabidopsis thaliana* [21]. Protein sequences were aligned using MAFFT [122] (--localpair --maxiterate 16 --reorder). Phylogenetic tree was constructed using IQ-TREE v2.0.6 [136] with maximum likelihood (ML) using 1000 ultra bootstrap replicates. The visualization of tree was done using Interactive Tree of Life (iTOL) v5. Colinear genes and syntenic blocks among the safflower and sunflower genomes as well as safflower and Arabidopsis genome were identified using MCScanX (-s 3 -b 2 -w 2 -e 10e-3 -k 40) [77] .

Exploring genetic basis of the safflower for the various agronomically important traits

Genomic DNA was sequenced on Illumina NovaSeq 6000 platform (Illumina, USA) to generate 150 bp paired end reads. Reads were cleaned and mapped to Safflower_A2 genome using BWA-mem v0.7.17 [83]. GATK v4.4.0 [84] was used for variant calling with parameters: --minimum-mapping-quality 20, --min-base-quality-score 20, and hard filtering was applied based on GATK

best practices recommendations [137] followed by filtration using VCFtools v0.1.15 (Supplementary Table S18).

Fast Tree v2.1.10 [86] with GTR model was used for construction of the phylogenetic tree using filtered SNPs and visualized using Interactive Tree of Life (iTOL) v5 [87]. Genetic structure of the core collection was assessed using ADMIXTURE v1.3.0 [88]. Number of clusters (K) were inferred based on lowest CV error. PCA was performed using PLINK v1.90b4.6 [89] with default parameters. The first two eigenvectors showing maximum variability were plotted using R. Pairwise F_{ST} between subpopulations inferred from ADMIXTURE (K=4) were calculated using VCFtools [90]. PopLD decay [91] with default settings was used for estimation of LD in safflower.

Phenotypic data described earlier [14] for eight traits including PH, HN, PB, DTF, OC, 100SW, OA and LA content from two independent growing seasons (2011-2012 and 2012-2013) were used. SNPs generated in the present study were filtered using TASSEL v5 [138]. GWAS analysis was conducted for data of two seasons independently using two single locus models [General Linear Model (GLM) and Mixed Linear Model (MLM)]; [139] and three multi-locus models [Multi-Locus Mixed Model (MLMM) [72], Fixed and random model Circulating Probability Unification (FarmCPU; [28]), and Bayesian-information and Linkage-disequilibrium Iteratively Nested Keyway (BLINK; [74]). These models were implemented using GAPIT v3 [140] in R programming software assigning PCAvalue of 4 (PCA.total=4) based on admixture analysis. GAPITv3 uses Benjamini–Hochberg method which is well known to control the False Discovery Rate (FDR). MTAs were considered significant at $p<0.0001$.

QTNs identified for eight traits were used for candidate gene analysis (CGA). LD block analysis was conducted using LDBlockShow [141] to define the region for CGA. Candidate genes were

subsequently searched within a 7 kb region upstream and downstream of the QTNs. To verify the association of candidate genes with traits, putative candidates were screened based on their annotated functions. Genes associated with metabolic pathways, stress responses, and traits such as oil biosynthesis, plant architecture, and flowering time were selected for Haplo-pheno analysis. The GenhapR [80] package was utilized to detect haplotypes within the LD regions of candidate genes. Association analysis was conducted for haplotypes represented in three or more individuals in the core collection, while other haplotypes were classified as rare. Haplotypes with high average values and present in a large number of accessions were designated as favourable haplotypes. The haplotypes found in accessions exhibiting the highest trait values were identified as superior haplotypes.

Candidate genes with significant functional associations to the traits were further analysed by the searching the 2 kb upstream and downstream to identify the SNPs in the vicinity of the genes. Following manual curation, 20 SNP sites associated with agronomic traits were selected for validation using the KASP assay on an appropriate panel representing high and low trait values. A total 46 accession were used in different panels (Supplementary Table S26). High-quality genomic DNA was extracted from these accessions using the HiPurA Plant Genomic DNA Miniprep Purification Kit (Cat. No. MB507). The Primers were designed using Web-based Allele-Specific Primer design tool (WASP) [142] and the assay was designed using web-based tool PrimerDigital [143] PCR reactions were performed as per the user manual (LGC Genomics, United Kingdom). The plate was read with FRET-capable plate reader (Victor X3, PerkinElmer) with the relevant filter sets for fluorescence detection. Fluorescence data was analyzed using KlusterCaller™ software (Version 3.4.1.36; LGC Genomics, UK), and genotypes were assigned based on the clustering of allele-specific fluorescence signals.

Pan-genome assembly, annotation, and PAV analysis

A pan-genome was constructed through an iterative mapping and assembly approach using sequencing data of core collection (Illumina short reads, 123 samples) and available chromosomal-level assemblies of safflower [12,13]. We constructed pan-genome assembly in four major steps detailed below (Figure 6a). In step 1, the chromosomal-level assemblies of safflower (Anhui1 and Chaunhangua1) were iteratively mapped to the Safflower_A2 reference sequence using minimap2 and identified novel segments were integrated into the reference genome. In step 2, filtered Illumina sequencing reads from the core collection were mapped to the reference genome using BWA-mem v0.7.17. In step 3, unmapped and discordant reads were extracted using SAMtools view v1.20 (-f4, -f8 and -f12) and assembled *de novo* using MaSurca v3.2.3 [144] with default settings (SOAP_ASSEMBLY=0, close gap=1). Contaminated reads from non-plants in the resultant contigs were identified using BLASTn (e-value=1×10^{-5}) against the NCBI-NR and RefSeq databases and discarded from the further analyses. To eliminate any other potential contamination, contigs were screened using NCBI-FCS[145] , and contigs containing non-plant sequences were removed. In step 4, all the novel sequences (Safflower_A2) were further assembled resulting in novel additional sequences. Repeat regions were identified using EDTA v2.0.0 [119] with the parameters --anno 1 and --sensitive 1. The genome was then masked using RepeatMasker v4.1.7 [120], which utilizes the Repbase (v20181026) library. The masked genome was subsequently used for gene prediction with BRAKER v3.0.4. For homology-based gene prediction protein sequences of *Arabidopsis thaliana*, *Helianthus annus*, *Lactuca sativa*, *Cyanara cardunculus* and *Carthamus tinctorius* were downloaded from Refseq and Swiss prot databases and were used as a hint. Predicted genes were clustered using CD-HIT v4.8.1[146] and redundancy was removed. Genes intersecting with repeat regions (>30%) were removed using BEDtools

intersect v2.21.0 [147]. The genes retained from the above steps were aligned to the Safflower_A2 genome, followed by the removal of genes showing high similarity (perc_identity =0.8 and query_cov =0.8). The remaining genes were considered pan-genes and used for downstream analyses. Proteins encoded by the pan-genes were subjected to functional annotation using OmicsBox v3.1.2 [128]. Functional enrichment was performed using Database for Annotation, Visualization, and Integrated Discovery (DAVID) [148]. Contigs from the above assembly were concatenated with the Safflower_A2 genome to construct a pan-genome (named Safpg_v1). PAV analysis was performed by aligning raw reads of genomes to genic sequences of Safpg_v1 using bowtie2 (--no-mixed, --local). Genes were considered present if 80% of the gene is covered by the reads with a minimum depth of 3, else marked as absent. Based on the presence of a gene in the accessions, it was assigned a category as core (≥97%), softcore (90-96%), shell (15-89%), cloud (<15%), or private (only in one accession). To check whether a pan-genome is saturated or not, core genome size and pan-genome size were fitted using the nls function in R.

Data availability

The raw sequencing data and genome assembly generated during this study has been deposited at NCBI under the BioProject PRJNA1089929. Genome assembly, functional annotation, protein, transcript sequence files pan-genome assembly and its annotation and PAV matrix, are available at the Safflower Genome Resource (SGR) (http://51.21.157.20:3002/). Bionano optical mapping data is available at SGR (http://51.21.157.20:3002/downloads/).

Declarations

Ethics approval: Not applicable

Consent for publication: Not applicable

Competing interests: The authors declare no competing interests.

Funding

This work was supported by Department of Biotechnology, Government of India grants to AJ and SG (BT/Ag/Network/Safflower/2019-20; Sub Projects 3 and 4).

Author contributions

MS and VB carried out all field and laboratory experiments; SG, AJ, MS and VB contributed to writing the manuscript; SG and AJ conceptualized, supervised the overall study and secured funding; MS, VB, PKO, HA, SG and RNS planned and performed bioinformatic analysis; VB, MS, AJ, SG and VJ planned and performed mapping studies. SC, HR, AV, AKP performed the experimental analysis of KASP validations assays.

Acknowledgements

MS, VB, PKO acknowledge junior and senior research fellowships provided by the Council of Scientific and Industrial Research, Ministry of Science and Technology, Government of India. Whereas SC acknowledge her junior research fellowships provided by University Grant Commission (UGC), Government of India.

References

1. Fernandez-Martinez J, Del Rio M, De Haro A. Survey of safflower (Carthamus tinctorius L.) germplasm for variants in fatty acid composition and other seed characters. *Euphytica*. Springer; 69:115–221993

2. Khalid N, Khan RS, Hussain MI, Farooq M, Ahmad A, Ahmed I. A comprehensive characterisation of safflower oil for its potential applications as a bioactive food ingredient - A review. *Trends Food Sci Technol*. 2017; doi: 10.1016/j.tifs.2017.06.009.

3. Sharma M, Bhardwaj V, Goswami P, Kalra A, Palchamy K, Jagannath A, et al.. Increasing Nutraceutical and Pharmaceutical Applications of Safflower: Genetic and Genomic Approaches. In: Kole C, editor. *Compend Crop Genome Des Nutraceuticals*. Singapore: Springer Nature Singapore

4. FAOSTAT. https://www.fao.org/faostat/en/#data/QCL (2024). Accessed 2024 Jan 13.

5. Safflower Oil Market. https://www.futuremarketinsights.com/reports/safflower-oil-market Accessed 2023 May 26.

6. Emongor V. Safflower (Carthamus tinctorius L.) the underutilized and neglected crop: a review. 2010

7. Ashri A. Evaluation of the germ plasm collection of safflower, Carthamus tinctorius LV Distribution and regional divergence for morphological characters. *Euphytica*. Springer; 24:651–91975

8. Yang Y-X, Wu W, Zheng Y-L, Chen L, Liu R-J, Huang C-Y. Genetic diversity and relationships among safflower (Carthamus tinctorius L.) analyzed by inter-simple sequence repeats (ISSRs). *Genet Resour Crop Evol*. Springer; 54:1043–512007

9. Ambreen H, Kumar S, Variath MT, Joshi G, Bali S, Agarwal M, et al.. Development of genomic microsatellite markers in Carthamus tinctorius L.(safflower) using next generation sequencing and assessment of their cross-species transferability and utility for diversity analysis. *PloS One*. Public Library of Science San Francisco, CA USA; 10:e01354432015

10. Kumar S, Ambreen H, Murali TV, Bali S, Agarwal M, Kumar A, et al.. Assessment of genetic diversity and population structure in a global reference collection of 531 accessions of Carthamus tinctorius L.(Safflower) using AFLP markers. *Plant Mol Biol Report*. Springer; 33:1299–3132015

11. Chapman MA, Burke JM. DNA sequence diversity and the origin of cultivated safflower (Carthamus tinctorius L.; Asteraceae). *BMC Plant Biol*. 2007; doi: 10.1186/1471-2229-7-60.

12. Wu Z, Liu H, Zhan W, Yu Z, Qin E, Liu S, et al.. The chromosome-scale reference genome of safflower (Carthamus tinctorius) provides insights into linoleic acid and flavonoid biosynthesis. *Plant Biotechnol J*. Association of Applied Biologists; :1–18 2021;

13. Chen J, Guo S, Hu X, Wang R, Jia D, Li Q, et al.. Whole-genome and genome-wide association studies improve key agricultural traits of safflower for industrial and medicinal use. *Hortic Res*. Oxford University Press; 10:uhad1972023;

14. Kumar S, Ambreen H, Variath MT, Rao AR, Agarwal M, Kumar A, et al.. Utilization of molecular, phenotypic, and geographical diversity to develop compact composite core collection in the oilseed crop, safflower (Carthamus tinctorius L.) through maximization strategy. *Front Plant Sci*. Frontiers Media SA; 7:15542016;

15. Lu C, Shen Q, Yang J, Wang B, Song C. The complete chloroplast genome sequence of Safflower (*Carthamus tinctorius* L.). *Mitochondrial DNA Part A*. 2016; doi: 10.3109/19401736.2015.1018217.

16. Ventimiglia M, Castellacci M, Usai G, Vangelisti A, Simoni S, Natali L, et al.. Discovering the Repeatome of Five Species Belonging to the Asteraceae Family: A Computational Study. *Plants*. MDPI; 12:14052023

17. Kirov I, Omarov M, Merkulov P, Dudnikov M, Gvaramiya S, Kolganova E, et al.. Genomic and transcriptomic survey provides new insight into the organization and transposition activity of highly expressed LTR retrotransposons of sunflower (Helianthus annuus l.). *Int J Mol Sci*. MDPI; 21:93312020

18. Masand M, Sharma S, Kumari S, Pal P, Majeed A, Singh G, et al.. High-quality haplotype-resolved chromosome assembly provides evolutionary insights and targeted steviol glycosides (SGs) biosynthesis in *Stevia rebaudiana* Bertoni. *Plant Biotechnol J*. 2024; doi: 10.1111/pbi.14446.

19. Mascagni F, Barghini E, Giordani T, Rieseberg LH, Cavallini A, Natali L. Repetitive DNA and plant domestication: variation in copy number and proximity to genes of LTR-retrotransposons among wild and cultivated sunflower (Helianthus annuus) genotypes. *Genome Biol Evol*. Oxford University Press; 7:3368–822015

20. Kourelis J, Van Der Hoorn RA. Defended to the nines: 25 years of resistance gene cloning identifies nine mechanisms for R protein function. *Plant Cell*. American Society of Plant Biologists; 30:285–992018

21. Hou X, Wang D, Cheng Z, Wang Y, Jiao Y. A near-complete assembly of an Arabidopsis thaliana genome. *Mol Plant*. Elsevier; 15:1247–502022

22. Yi L, Bao H, Wu Y, Mu Y, Du C, Peng J, et al.. Chromosome-level genome assemblies of sunflower oilseed and confectionery cultivars. *Sci Data*. Nature Publishing Group UK London; 12:242025

23. Filippi CV, Merino GA, Montecchia JF, Aguirre NC, Rivarola M, Naamati G, et al.. Genetic diversity, population structure and linkage disequilibrium assessment among international sunflower breeding collections. *Genes*. MDPI; 11:2832020

24. Liu H, Zhou H, Wu Y, Li X, Zhao J, Zuo T, et al.. The impact of genetic relationship and linkage disequilibrium on genomic selection. *PloS One*. Public Library of Science San Francisco, CA USA; 10:e01323792015

25. Omata Y, Sato R, Mishiro-Sato E, Kano K, Ueda H, Hara-Nishimura I, et al.. Lipid droplets in Arabidopsis thaliana leaves contain myosin-binding proteins and enzymes associated with furan-containing fatty acid biosynthesis. *Front Plant Sci*. Frontiers Media SA; 15:13314792024

26. Jameson PE, Song J. Cytokinin: a key driver of seed yield. *J Exp Bot*. Oxford University Press; 67:593–6062016

27. Madson M, Dunand C, Li X, Verma R, Vanzin GF, Caplan J, et al.. The MUR3 gene of Arabidopsis encodes a xyloglucan galactosyltransferase that is evolutionarily related to animal exostosins. *Plant Cell*. American Society of Plant Biologists; 15:1662–702003

28. Huang AH. Plant lipid droplets and their associated proteins: potential for rapid advances. *Plant Physiol*. American Society of Plant Biologists; 176:1894–9182018

29. Lou L, Ding L, Wang T, Xiang Y. Emerging roles of RNA-binding proteins in seed development and performance. *Int J Mol Sci*. MDPI; 21:68222020

30. Lu C, Ren X, Zhou Y, Jia S, Bai H, Zhao D, et al.. OsOFP9 regulates diverse key traits of rice by integrating multiple plant hormones. *Plant J*. 2025; doi: 10.1111/tpj.70044.

31. Sauveplane V, Kandel S, Kastner P, Ehlting J, Compagnon V, Werck-Reichhart D, et al.. *Arabidopsis thaliana* CYP77A4 is the first cytochrome P450 able to catalyze the epoxidation of free fatty acids in plants. *FEBS J*. 2009; doi: 10.1111/j.1742-4658.2008.06819.x.

32. Xiang F, Liu W, Liu X, Song Y, Zhang Y, Zhu X, et al.. Direct balancing of lipid mobilization and reactive oxygen species production by the epoxidation of fatty acid catalyzed by a cytochrome P450 protein during seed germination. *New Phytol*. 2023; doi: 10.1111/nph.18669.

33. Shu K, Yang W. E3 ubiquitin ligases: ubiquitous actors in plant development and abiotic stress responses. *Plant Cell Physiol*. Oxford University Press; 58:1461–762017

34. Jang G, Kim J, Yu J-K, Kim H-J, Kim Y, Kim D-W, et al.. Cost-effective unmanned aerial vehicle (UAV) platform for field plant breeding application. *Remote Sens*. MDPI; 12:9982020

35. Li S-W, Shi R-F, Leng Y, Zhou Y. Transcriptomic analysis reveals the gene expression profile that specifically responds to IBA during adventitious rooting in mung bean seedlings. *BMC Genomics*. 2016; doi: 10.1186/s12864-016-2372-4.

36. Rosquete MR, Worden N, Ren G, Sinclair RM, Pfleger S, Salemi M, et al.. AtTRAPPC11/ROG2: a role for TRAPPs in maintenance of the plant trans-Golgi network/early endosome organization and function. *Plant Cell*. American Society of Plant Biologists; 31:1879–982019

37. Li S. The *Arabidopsis thaliana* TCP transcription factors: A broadening horizon beyond development. *Plant Signal Behav*. 2015; doi: 10.1080/15592324.2015.1044192.

38. Li W, Li D-D, Han L-H, Tao M, Hu Q-Q, Wu W-Y, et al.. Genome-wide identification and characterization of TCP transcription factor genes in upland cotton (Gossypium hirsutum). *Sci Rep*. Nature Publishing Group UK London; 7:101182017

39. Shigeyama T, Watanabe A, Tokuchi K, Toh S, Sakurai N, Shibuya N, et al.. α-Xylosidase plays essential roles in xyloglucan remodelling, maintenance of cell wall integrity, and seed germination in Arabidopsis thaliana. *J Exp Bot*. Oxford University Press UK; 67:5615–292016

40. Bayer PE, Golicz AA, Scheben A, Batley J, Edwards D. Plant pan-genomes are the new reference. *Nat Plants*. Nature Publishing Group UK London; 6:914–202020

41. Hare, Emily E., and J. Spencer Johnston. "Genome size determination using flow cytometry of propidium iodide-stained nuclei." *Molecular methods for evolutionary genetics* (2011): 3-12.

42. Gabriel L, Brůna T, Hoff KJ, Ebel M, Lomsadze A, Borodovsky M, et al.. BRAKER3: Fully automated genome annotation using RNA-Seq and protein evidence with GeneMark-ETP, AUGUSTUS and TSEBRA. *BioRxiv Prepr Serv Biol*. 2023; doi: 10.1101/2023.06.10.544449.

43. Jiang S, An H, Xu F, Zhang X. Chromosome-level genome assembly and annotation of the loquat (Eriobotrya japonica) genome. *GigaScience*. Oxford University Press; 9:giaa0152020

44. Wang J, Wang X, Ma Y, Gao R, Wang Y, An Z, et al.. Lonicera caerulea genome reveals molecular mechanisms of freezing tolerance and anthocyanin biosynthesis. *J Adv Res*. Elsevier; 2024

45. Xue J-Y, Takken FL, Nepal MP, Maekawa T, Shao Z-Q. Evolution and functional mechanisms of plant disease resistance. *Front Genet*. Frontiers; 11:5932402020

46. Monosi B, Wisser RJ, Pennill L, Hulbert SH. Full-genome analysis of resistance gene homologues in rice. *Theor Appl Genet*. Springer; 109:1434–472004

47. Bai L, Zhou P, Li D, Ju X. Changes in the gastrointestinal microbiota of children with acute lymphoblastic leukaemia and its association with antibiotics in the short term. *J Med Microbiol*. Microbiology Society; 66:1297–3072017

48. Neupane S, Andersen EJ, Neupane A, Nepal MP. Genome-wide identification of NBS-encoding resistance genes in sunflower (Helianthus annuus L.). *Genes*. MDPI; 9:3842018

49. Saile SC, El Kasmi F. Small family, big impact: RNL helper NLRs and their importance in plant innate immunity. *PLoS Pathog*. Public Library of Science San Francisco, CA USA; 19:e10113152023

50. Athanasopoulos V, Barker A, Yu D, Tan AH, Srivastava M, Contreras N, et al.. The ROQUIN family of proteins localizes to stress granules via the ROQ domain and binds target mRNAs. *FEBS J*. 2010; doi: 10.1111/j.1742-4658.2010.07628.x.

51. Schultink A, Qi T, Lee A, Steinbrenner AD, Staskawicz B. Roq1 mediates recognition of the Xanthomonas and Pseudomonas effector proteins XopQ and HopQ1. *Plant J*. 2017; doi: 10.1111/tpj.13715.

52. Thomas NC, Hendrich CG, Gill US, Allen C, Hutton SF, Schultink A. The immune receptor Roq1 confers resistance to the bacterial pathogens Xanthomonas, Pseudomonas syringae, and Ralstonia in tomato. *Front Plant Sci*. Frontiers Media SA; 11:4632020

53. Yuan B, Li C, Wang Q, Yao Q, Guo X, Zhang Y, et al.. Identification and functional characterization of the RPP13 gene family in potato (Solanum tuberosum L.) for disease resistance. *Front Plant Sci*. Frontiers Media SA; 15:15150602025

54. Calonnec A, Deliere L, Cartolaro P, Delmotte F, Forget D, Wiedemann-Merdinoglu S, et al.. Evaluation of grapevine resistance to downy and powdery mildew in a population segregating for run1 and rpv1 resistance genes. 2008

55. Gore MA, Hayes AJ, Jeong SC, Yue YG, Buss GR, Maroof MS. Mapping tightly linked genes controlling potyvirus infection at the *Rsv1* and *Rpv1* region in soybean. *Genome*. 2002; doi: 10.1139/g02-009.

56. GoreM.A. 2000 *High-resolution mapping of the region around the soybean virus resistance genes, and Master's thesis, Virginia Polytechnic Inst*. and State Univ., Blacksburg

57. Poque S, Pagny G, Ouibrahim L, Chague A, Eyquard J-P, Caballero M, et al.. Allelic variation at the rpv1 locus controls partial resistance to Plum pox virus infection in Arabidopsis thaliana. *BMC Plant Biol*. 2015; doi: 10.1186/s12870-015-0559-5.

58. Williams SJ, Yin L, Foley G, Casey LW, Outram MA, Ericsson DJ, et al.. Structure and function of the TIR domain from the grape NLR protein RPV1. *Front Plant Sci*. Frontiers Media SA; 7:18502016

59. Zhang X, Wang G, Qu X, Wang M, Guo H, Zhang L, et al.. A truncated CC-NB-ARC gene TaRPP13L1-3D positively regulates powdery mildew resistance in wheat via the RanGAP-WPP complex-mediated nucleocytoplasmic shuttle. *Planta*. Springer; 255:602022

60. Hajabdollahi N, Saberi Riseh R, Khodaygan P, Moradi M, Moslemkhani K. Differentially expressed genes in resistant and susceptible *Pistacia vera* L. Cultivars in response to *Pseudomonas fluorescens* and *Phytophthora parsiana*. *Biocontrol Sci Technol*. 2021; doi: 10.1080/09583157.2020.1867706.

61. Qi T, Seong K, Thomazella DPT, Kim JR, Pham J, Seo E, et al.. NRG1 functions downstream of EDS1 to regulate TIR-NLR-mediated plant immunity in *Nicotiana benthamiana*. *Proc Natl Acad Sci*. 2018; doi: 10.1073/pnas.1814856115.

62. Michel V, Julio E, Candresse T, Cotucheau J, Decorps C, Volpatti R, et al.. *NtTPN1* : a *RPP8* -like *R* gene required for *Potato virus Y* -induced veinal necrosis in tobacco. *Plant J*. 2018; doi: 10.1111/tpj.13980.

63. Jia RZ, Ming R, Zhu YJ. Genome-wide analysis of Nucleotide-Binding Site (NBS) disease Resistance (R) Genes in Sacred Lotus (Nelumbo nucifera Gaertn.) reveals their transition role during early evolution of land plants. *Trop Plant Biol*. Springer; 6:98–1162013

64. Yong CSY, Atheeqah-Hamzah N. Transcriptome-wide Identification of Nine Tandem Repeat Protein Families in Roselle (Hibiscus sabdariffa L.). *Trop Life Sci Res*. 35:1212024

65. Zhu Q-H, Stephen S, Kazan K, Jin G, Fan L, Taylor J, et al.. Characterization of the defense transcriptome responsive to Fusarium oxysporum-infection in Arabidopsis using RNA-seq. *Gene*. Elsevier; 512:259–662013

66. Kanazin V, Marek LF, Shoemaker RC. Resistance gene analogs are conserved and clustered in soybean. *Proc Natl Acad Sci*. 1996; doi: 10.1073/pnas.93.21.11746.

67. Sutanto A, Sukma D, Hermanto C. Isolation and characterization of resistance gene analogue (RGA) from Fusarium resistant banana cultivars. *Emir J Food Agric*. Pensoft Publishers; 26:5082014

68. Thakur S, Syed SE, Pandey S, Sharma S, Saharan MS, Bashyal BM. In silico analysis and expression profiling for Resistance Gene Analogues (RGAs) and defence-related genes in early germinating conditions of rice against bakanae disease caused by Fusarium fujikuroi. *Indian J Biochem Biophys IJBB*. 61:714–302024

69. Xu X, Hayashi N, Wang C-T, Fukuoka S, Kawasaki S, Takatsuji H, et al.. Rice blast resistance gene Pikahei-1 (t), a member of a resistance gene cluster on chromosome 4, encodes a nucleotide-binding site and leucine-rich repeat protein. *Mol Breed*. Springer; 34:691–7002014

70. Ambreen H, Kumar S, Kumar A, Agarwal M, Jagannath A, Goel S. Association mapping for important agronomic traits in safflower (Carthamus tinctorius L.) core collection using microsatellite markers. *Front Plant Sci*. Frontiers Media SA; 9:4022018

71. Khan SU, Saeed S, Khan MHU, Fan C, Ahmar S, Arriagada O, et al.. Advances and challenges for QTL analysis and GWAS in the plant-breeding of high-yielding: a focus on rapeseed. *Biomolecules*. MDPI; 11:15162021

72. Segura V, Vilhjálmsson BJ, Platt A, Korte A, Seren Ü, Long Q, et al.. An efficient multi-locus mixed-model approach for genome-wide association studies in structured populations. *Nat Genet*. Nature Publishing Group US New York; 44:825–302012

73. Liu G, Zhao Y, Gowda M, Longin CFH, Reif JC, Mette MF. Predicting hybrid performances for quality traits through genomic-assisted approaches in Central European wheat. *PLoS One*. Public Library of Science San Francisco, CA USA; 11:e01586352016

74. Huang M, Liu X, Zhou Y, Summers RM, Zhang Z. BLINK: a package for the next level of genome-wide association studies with both individuals and markers in the millions. *GigaScience*. 2019; doi: 10.1093/gigascience/giy154.

75. Dujak C, Coleto-Alcudia V, Aranzana MJ. Genomic analysis of fruit size and shape traits in apple: unveiling candidate genes through GWAS analysis. *Hortic Res*. Oxford University Press; 11:uhad2702024

76. Bukomarhe CB, Kimwemwe PK, Githiri SM, Mamati EG, Kimani W, Mutai C, et al.. Association mapping of candidate genes associated with iron and zinc content in rice (Oryza sativa L.) grains. *Genes*. MDPI; 14:18152023

77. Jia Q, Hu S, Li X, Wei L, Wang Q, Zhang W, et al.. Identification of candidate genes and development of KASP markers for soybean shade-tolerance using GWAS. *Front Plant Sci*. Frontiers Media SA; 15:14795362024

78. Bhat JA, Karikari B, Adeboye KA, Ganie SA, Barmukh R, Hu D, et al.. Identification of superior haplotypes in a diverse natural population for breeding desirable plant height in soybean. *Theor Appl Genet*. 2022; doi: 10.1007/s00122-022-04120-0.

79. Wang X, Pang Y, Zhang J, Wu Z, Chen K, Ali J, et al.. Genome-wide and gene-based association mapping for rice eating and cooking characteristics and protein content. *Sci Rep*. Nature Publishing Group UK London; 7:172032017

80. Zhang R, Jia G, Diao X. geneHapR: an R package for gene haplotypic statistics and visualization. *BMC Bioinformatics*. 2023; doi: 10.1186/s12859-023-05318-9.

81. Bhat JA, Yu D, Bohra A, Ganie SA, Varshney RK. Features and applications of haplotypes in crop breeding. *Commun Biol*. Nature Publishing Group UK London; 4:12662021

82. Dormatey R, Sun C, Ali K, Coulter JA, Bi Z, Bai J. Gene pyramiding for sustainable crop improvement against biotic and abiotic stresses. *Agronomy*. MDPI; 10:12552020

83. Golicz AA, Bayer PE, Barker GC, Edger PP, Kim H, Martinez PA, et al.. The pangenome of an agronomically important crop plant Brassica oleracea. *Nat Commun*. Nature Publishing Group UK London; 7:133902016

84. Montenegro JD, Golicz AA, Bayer PE, Hurgobin B, Lee H, Chan CK, et al.. The pangenome of hexaploid bread wheat. *Plant J*. 2017; doi: 10.1111/tpj.13515.

85. Qin P, Lu H, Du H, Wang H, Chen W, Chen Z, et al.. Pan-genome analysis of 33 genetically diverse rice accessions reveals hidden genomic variations. *Cell*. Elsevier; 184:3542–582021

86. Bayer PE, Petereit J, Durant É, Monat C, Rouard M, Hu H, et al.. Wheat Panache: A pangenome graph database representing presence–absence variation across sixteen bread wheat genomes. *Plant Genome*. 2022; doi: 10.1002/tpg2.20221.

87. Anisimova OK, Kochieva EZ, Shchennikova AV, Filyushin MA. Thaumatin-like protein (TLP) genes in garlic (Allium sativum L.): Genome-wide identification, characterization, and expression in response to Fusarium proliferatum infection. *Plants*. MDPI; 11:7482022

88. De Jesús-Pires C, Ferreira-Neto JRC, Pacifico Bezerra-Neto J, Kido EA, De Oliveira Silva RL, Pandolfi V, et al.. Plant Thaumatin-like Proteins: Function, Evolution and Biotechnological Applications. *Curr Protein Pept Sci*. 2020; doi: 10.2174/1389203720666190318164905.

89. Mahdavi F, Sariah M, Maziah M. Expression of rice thaumatin-like protein gene in transgenic banana plants enhances resistance to Fusarium wilt. *Appl Biochem Biotechnol*. Springer; 166:1008–192012

90. Doležel J, Greilhuber J, Suda J. Estimation of nuclear DNA content in plants using flow cytometry. *Nat Protoc*. Nature Publishing Group; 2007; doi: 10.1038/nprot.2007.310.

91. Wang H, Liu B, Zhang Y, Jiang F, Ren Y, Yin L, et al.. Estimation of genome size using k-mer frequencies from corrected long reads. *ArXiv Prepr ArXiv200311817*. 2020

92. fanwei. fanagislab/GCE. https://github.com/fanagislab/GCE

93. Cheng H, Concepcion GT, Feng X, Zhang H, Li H. Haplotype-resolved de novo assembly using phased assembly graphs with hifiasm. *Nat Methods*. Nature Publishing Group US New York; 18:170–52021

94. Hu J, Fan J, Sun Z, Liu S. NextPolish: a fast and efficient genome polishing tool for long-read assembly. *Bioinformatics*. Oxford University Press; 36:2253–52020

95. Ghurye J, Pop M, Koren S, Bickhart D, Chin C-S. Scaffolding of long read assemblies using long range contact information. *BMC Genomics*. 2017; doi: 10.1186/s12864-017-3879-z.

96. Walker BJ, Abeel T, Shea T, Priest M, Abouelliel A, Sakthikumar S, et al.. Pilon: an integrated tool for comprehensive microbial variant detection and genome assembly improvement. *PloS One*. Public Library of Science San Francisco, USA; 9:e1129632014

97. Vasimuddin M, Misra S, Li H, Aluru S. Efficient architecture-aware acceleration of BWA-MEM for multicore systems. *2019 IEEE Int Parallel Distrib Process Symp IPDPS*. IEEE; p. 314–24.

98. sanger-tol/ Tree of Life programme https://itol.embl.de/

99. Durand NC, Shamim MS, Machol I, Rao SS, Huntley MH, Lander ES, et al.. Juicer provides a one-click system for analyzing loop-resolution Hi-C experiments. *Cell Syst*. Elsevier; 3:95–82016

100. Dierckxsens N, Mardulyn P, Smits G. NOVOPlasty: de novo assembly of organelle genomes from whole genome data. *Nucleic Acids Res*. Oxford University Press; 45:e18–e182017

101. Li H. Minimap2: pairwise alignment for nucleotide sequences. *Bioinformatics*. Oxford University Press; 34:3094–1002018

102. Rhie A, Walenz BP, Koren S, Phillippy AM. Merqury: reference-free quality, completeness, and phasing assessment for genome assemblies. *Genome Biol*. 2020; doi: 10.1186/s13059-020-02134-9.

103. Simão FA, Waterhouse RM, Ioannidis P, Kriventseva EV, Zdobnov EM. BUSCO: assessing genome assembly and annotation completeness with single-copy orthologs. *Bioinformatics*. Oxford University Press; 31:3210–22015

104. Ou S, Chen J, Jiang N. Assessing genome assembly quality using the LTR Assembly Index (LAI). *Nucleic Acids Res*. Oxford University Press; 46:e126–e1262018

105. tolkit/telomeric-identifier tolkit https://github.com/tolkit/telomeric-identifier

106. Wlodzimierz P, Hong M, Henderson IR. TRASH: tandem repeat annotation and structural hierarchy. *Bioinformatics*. Oxford University Press; 39:btad3082023

107. Cabanettes, Floréal, and Christophe Klopp.. D-GENIES: dot plot large genomes in an interactive, efficient and simple way. *PeerJ*. 62018

108. Li H, Handsaker B, Wysoker A, Fennell T, Ruan J, Homer N, et al.. The Sequence Alignment/Map format and SAMtools. *Bioinformatics*. 2009; doi: 10.1093/bioinformatics/btp352.

109. Heller D, Vingron M. SVIM: structural variant identification using mapped long reads. *Bioinformatics*. Oxford University Press; 35:2907–152019

110. Quigley S, Damas J, Larkin DM, Farré M. syntenyPlotteR: a user-friendly R package to visualize genome synteny, ideal for both experienced and novice bioinformaticians. *Bioinforma Adv*. Oxford University Press; 3:vbad1612023

111. Gel B, Serra E. karyoploteR: an R/Bioconductor package to plot customizable genomes displaying arbitrary data. *Bioinformatics*. Oxford University Press; 33:3088–902017

112. Elshire RJ, Glaubitz JC, Sun Q, Poland JA, Kawamoto K, Buckler ES, et al.. A robust, simple genotyping-by-sequencing (GBS) approach for high diversity species. *PloS One*. Public Library of Science San Francisco, USA; 6:e193792011

113. Taylor J, Butler D. R Package ASMap: Efficient Genetic Linkage Map Construction and Diagnosis. *J Stat Softw*. 2017; doi: 10.18637/jss.v079.i06.

114. Tang H, Zhang X, Miao C, Zhang J, Ming R, Schnable JC, et al.. ALLMAPS: robust scaffold ordering based on multiple maps. *Genome Biol*. 2015; doi: 10.1186/s13059-014-0573-1.

115. : Iso-Seq Home. Iso-Seq Docs. https://isoseq.how/ Accessed 2024 Jul 13.

116. . PacificBiosciences/pbmm2. PacBio; https://github.com/PacificBiosciences/pbmm2

117. Stanke M, Keller O, Gunduz I, Hayes A, Waack S, Morgenstern B. AUGUSTUS: ab initio prediction of alternative transcripts. *Nucleic Acids Res*. 2006; doi: 10.1093/nar/gkl200.

118. Trincado JL, Entizne JC, Hysenaj G, Singh B, Skalic M, Elliott DJ, et al.. SUPPA2: fast, accurate, and uncertainty-aware differential splicing analysis across multiple conditions. *Genome Biol*. 2018; doi: 10.1186/s13059-018-1417-1.

119. Ou S, Su W, Liao Y, Chougule K, Agda JR, Hellinga AJ, et al.. Benchmarking transposable element annotation methods for creation of a streamlined, comprehensive pipeline. *Genome Biol*. BioMed Central; 20:1–182019

120. Chen N. Using RepeatMasker to Identify Repetitive Elements in Genomic Sequences. *Curr Protoc Bioinforma*. 2004; doi: 10.1002/0471250953.bi0410s05.

121. Novák P, Hoštáková N, Neumann P, Macas J. DANTE and DANTE_LTR: Lineage-centric annotation pipelines for long terminal repeat retrotransposons in plant genomes. *bioRxiv*. Cold Spring Harbor Laboratory; :2024–04 2024

122. Katoh K, Standley DM. MAFFT multiple sequence alignment software version 7: improvements in performance and usability. *Mol Biol Evol*. Society for Molecular Biology and Evolution; 30:772–802013

123. LTR_Retriever. https://github.com/oushujun/LTR_retriever

124. Barrnap. https://github.com/tseemann/barrnap

125. Chan PP, Lin BY, Mak AJ, Lowe TM. tRNAscan-SE 2.0: improved detection and functional classification of transfer RNA genes. *Nucleic Acids Res*. Oxford University Press; 49:9077–962021;

126. Besemer J, Borodovsky M. GeneMark: web software for gene finding in prokaryotes, eukaryotes and viruses. *Nucleic Acids Res*. 2005; doi: 10.1093/nar/gki487.

127. Fu L, Niu B, Zhu Z, Wu S, Li W. CD-HIT: accelerated for clustering the next-generation sequencing data. *Bioinformatics*. Oxford University Press; 28:3150–22012

128.Bioinformatics Software OmicsBox | Biobam. https://www.biobam.com/omicsbox/ Accessed 2024 Jul 13.

129. KEGG: Kyoto Encyclopedia of Genes and Genomes. https://www.genome.jp/kegg/ Accessed 2024 Jul 13.

130. Quevillon E, Silventoinen V, Pillai S, Harte N, Mulder N, Apweiler R, et al.. InterProScan: protein domains identifier. *Nucleic Acids Res*. 2005; doi: 10.1093/nar/gki442.

131. Zheng Y, Jiao C, Sun H, Rosli HG, Pombo MA, Zhang P, et al.. iTAK: A Program for Genome-wide Prediction and Classification of Plant Transcription Factors, Transcriptional Regulators, and Protein Kinases. *Mol Plant*. 2016; doi: 10.1016/j.molp.2016.09.014.

132. Calle García J, Guadagno A, Paytuvi-Gallart A, Saera-Vila A, Amoroso CG, D'Esposito D, et al.. PRGdb 4.0: an updated database dedicated to genes involved in plant disease resistance process. *Nucleic Acids Res*. Oxford University Press; 50:D1483–902022

133. Delorenzi M, Speed T. An HMM model for coiled-coil domains and a comparison with PSSM-based predictions. *Bioinformatics*. Oxford University Press; 18:617–252002

134. Mistry J, Chuguransky S, Williams L, Qureshi M, Salazar GA, Sonnhammer ELL, et al.. Pfam: The protein families database in 2021. *Nucleic Acids Res*. 2021; doi: 10.1093/nar/gkaa913.

135. NCBI Conserved Domain Search. https://www.ncbi.nlm.nih.gov/Structure/cdd/wrpsb.cgi Accessed 2024 Jul 13.

136. Minh BQ, Schmidt HA, Chernomor O, Schrempf D, Woodhams MD, Von Haeseler A, et al.. IQ-TREE 2: new models and efficient methods for phylogenetic inference in the genomic era. *Mol Biol Evol*. Oxford University Press; 37:1530–42020

137. Grzybowski MW, Mural RV, Xu G, Turkus J, Yang J, Schnable JC. A common resequencing-based genetic marker data set for global maize diversity. *Plant J*. 2023; doi: 10.1111/tpj.16123.

138. Bradbury PJ, Zhang Z, Kroon DE, Casstevens TM, Ramdoss Y, Buckler ES. TASSEL: software for association mapping of complex traits in diverse samples. *Bioinformatics*. 2007; doi: 10.1093/bioinformatics/btm308.

139. Zhang Z, Ersoz E, Lai C-Q, Todhunter RJ, Tiwari HK, Gore MA, et al.. Mixed linear model approach adapted for genome-wide association studies. *Nat Genet*. 2010; doi: 10.1038/ng.546.

140. Wang J, Zhang Z. GAPIT Version 3: Boosting Power and Accuracy for Genomic Association and Prediction. *Genomics Proteomics Bioinformatics*. 2021; doi: 10.1016/j.gpb.2021.08.005.

141. Dong S-S, He W-M, Ji J-J, Zhang C, Guo Y, Yang T-L. LDBlockShow: a fast and convenient tool for visualizing linkage disequilibrium and haplotype blocks based on variant call format files. *Brief Bioinform*. Oxford University Press; 22:bbaa2272021

142. Wangkumhang P, Chaichoompu K, Ngamphiw C, Ruangrit U, Chanprasert J, Assawamakin A, et al.. WASP: a Web-based Allele-Specific PCR assay designing tool for detecting SNPs and mutations. *BMC Genomics*. 2007; doi: 10.1186/1471-2164-8-275.

143. Kalendar R, Shustov AV, Akhmetollayev I, Kairov U. Designing allele-specific competitive-extension PCR-based assays for high-throughput genotyping and gene characterization. *Front Mol Biosci*. Frontiers Media SA; 2022 9:7739562022

144. Zimin AV, Marçais G, Puiu D, Roberts M, Salzberg SL, Yorke JA. The MaSuRCA genome assembler. *Bioinformatics*. Oxford University Press, 2013; 29:2669–772013

145. Astashyn A, Tvedte ES, Sweeney D, Sapojnikov V, Bouk N, Joukov V, et al. Rapid and sensitive detection of genome contamination at scale with FCS-GX. *Genome Biol*. 2024; doi: 10.1186/s13059-024-03198-7.

146. Li W, Godzik A. Cd-hit: a fast program for clustering and comparing large sets of protein or nucleotide sequences. *Bioinformatics*. Oxford University Press; 22:1658–92006

147. Quinlan AR, Hall IM. BEDTools: a flexible suite of utilities for comparing genomic features. *Bioinformatics*. Oxford University Press; 26:841–22010

148. Sherman BT, Hao M, Qiu J, Jiao X, Baseler MW, Lane HC, et al.. DAVID: a web server for functional enrichment analysis and functional annotation of gene lists (2021 update). *Nucleic Acids Res*. Oxford University Press; 50:W216–212022

Figure legends

Fig. 1: Overview of the Safflower_A2 genome (a) The outermost layer of the circos represents the twelve assembled chromosomes; (b) Repetitive elements density; (c) Gene density; (d) Distribution of simple sequence repeats within the genome; (e) Distribution of single nucleotide polymorphisms (SNPs) across the chromosomes; (f) GC content of the safflower genome.

Fig. 2: Overview of annotation of protein-coding genes for Safflower_A2 genome assembly. (a) Functional annotation of protein coding genes using RefSeq, Cluster of Orthologous Genes (KOG), Gene Ontology (GO), Kyoto Encyclopaedia of Genes and Genomes (KEGG) and Enzyme Code (EC) databases (b) Frequency histogram showing the distribution of top 20 GO terms across 3 categories: Biological Process, Cellular Component and Molecular Function (c) Top 20 members of Transcription Factors (TFs), Transcription Regulators (TRs) and Protein Kinases (PKs) identified in the Safflower_A2 genome (d) Distribution of KOG categories across protein-coding genes.

Fig. 3: Overview of R genes in the Safflower_A2 genome. (a) Different domains present in R genes of safflower (b) Distribution of the NLR genes on safflower chromosomes (e) (c) Ka/Ks analysis of NLR genes (d) Phylogenetic analysis of NLR genes of safflower, sunflower and *Arabidopsis thaliana.*

Fig. 4: Diversity analysis and Genome Wide Association study of Safflower core collection. (a) Admixture plot showing four subpopulations ADI (Red), ADII (Green), AD III (Cyan), AD IV (Purple) (b) Phylogenetic tree showing evolutionary analysis (c) Marker density plot for 320,399 SNPs used in GWAS analysis for chromosomes 1 to 12, colours indicate marker density per Mb

(d) Ideogram of QTNs detected from GWAS. QTNs for traits oil content, oleic and linoleic acid, hundred seed weight, plant height, head number, primary branches and days to 50% flowering detected across two growing seasons (2011-12 and 2012-13) are shown according to their respective chromosomal (labelled 1-12) positions.

Fig. 5: Exploration of the genetic basis of oil content in the safflower: (a) QQ and Manhattan plots for oil content representing all the multi-locus models. (b) LD-Block of 7 kb depicting correlation between the SNPs for the oil content (c) Haploblocks comprising the QTN OC1 (CtA_chr11_76393484), OC8 (CtA_chr11_76395157), OC13 (CtA_chr11_76395310) and SNPs of gene BIG GRAIN (represented by *) (d) Haplo-network representing network of the haplotypes (e) Correlation of the favourable haplotypes with phenotypic data for season 1 (OC_1: 2011-12) and (f) season 2 (OC_2: 2012-13).

Fig. 6: The pan-genome of safflower. (a) Schematic diagram for the construction of pan-genome. (b) Functional enrichment of the novel genes of the safflower pan-genome (c) Classification of pan-genes based on PAV analysis (d) PAV matrix showing the presence (green) and absence (pink) of pan-genes in the different accessions

 Click here to access/download;Table;gigacience tables.docx

Table 1: Comparison of Safflower_A2 genome with earlier published genome assemblies of Safflower

Genome feature	**Safflower_A2**	**Anhui_1**	**Chuanhonghua 1**
**Assembly statistics**			
k-mer based genome size estimation (in Gb)	1.17	1.17	1.17
Contigs	2427	368	3941
Length of primary assembly (Mb)	1154	1070	1171
N50 (Mb)	8.9	21.3	1.071
Pseudochromosomes	12	12	12
Length of final assembly (Gb)	1.09	1.05	1.174
N50 (Mb)	88.4	88.2	96.3
Longest scaffold (Mb)	111	106.7	185
Unplaced contigs	1684	240	509
Size of remaining contigs (Mb)	66.3	Not available	Not available
**Quality assessment**			
Complete BUSCO	97.90%	90.70%	89.79%
Mapping proportion (Long reads)	99.29%	98.10%	93.36%
**Annotation**			
Repetitive elements	71.30%	60.13%	71.41%
Number of transcripts	59,995	45,331	Not available
Complete BUSCO	91.5 %	86.20 %	71.70 %
Average exons per gene	4.024	6.54	5.92
Mean exon length (bp)	265.84	269.59	235.66
Mean CDS length (bp)	1215	1265.89	Not available

Table 2: Table representing Quantative trait nucleotides (QTNs) marking candidate genes and associated haplotypes for various important agronomical traits in safflower.

Trait	QTN	Chromosome	Position	Alleles	Candidate gene and its annotation	Numb-er of Haplotypes	Number of SNPs in Haploblock	Number of accessions exhibiting the haplotypes	Favourable haplotype(s)	Haplotype location w.r.t. gene	Distance from gene (bp)	Superior Haplotypes
**100 seed weight**	SW10	3	74251721	G,A	g35324; RNA-binding protein 2-like isoform X2	60	40	112	H001(53)	Downstream	67	H019, H021
	SW23	3	74075377	C,T	g35305; Peptidyl-prolyl cis-trans isomerase CYP57 isoform X1	50	17	118	H001(57), H002 (4) ,H003 (3),H004(3)	Downstream	10	H001, H031, H002
	SW3	3	72485086	A,G	g35324 ; Xyloglucan galactosyltransferase XLT2	48	32	115	H001(61), H002 (6)	Gene within Haplotype	0	H024, H008, H030
	SW31	5	9066250	G,A	g42789; Oleosin-B6-like	28	13	122	H001(94)	Gene within Haplotype	0	H021, H002
	SW37	7	81516758	T,A	g57921; Protein FRIGIDA-ESSENTIAL 1-like isoform	30	16	122	H001(93)	Downstream	199	

**Days to 50% Flowering**	DTF7	3	54974331	C,T	g34050; Exocyst complex component SEC5A-like isoform X2	74	26	113	H001(39), H002 (2)	Gene within Haplotype	0	H031, H029, H030
	DTF10	7	86666550	G,T	g58272 ; E3 ubiquitin-protein ligase COP1-like	80	13	115	H001(29), H002 (3), H003 (3)	Upstream	605	H058, H053, H080
	DTF2	11	7169035	C,T	g12806 ; E3 ubiquitin-protein ligase UPL1-like	13	6	123	H001(89), H002 (7),H003 (6),H004(5),H005(4),H006(3)	Downstream	4954	H001, H005, H006
	DTF2	11	7169035	C,T	g12807; 40S ribosomal protein S8	13	6	123	H001(89), H002 (7), H003 (6), H004(5), H005(4), H006(3)	Gene within Haplotype	0	H001, H005, H006
**Number of Heads**	HN5	4	5068529	G,A	g37215; Alpha-xylosidase 1-like	40	23	115	H001 (76)	Upstream	530	H01, H024
**Oleic and Linoleic Acid**	OA-LA15	5	18156971	C,G	g43426; Cytochrome P450 71A4-like	21	26	123	H001 (53), H002 (36), H003 (11), H004(6)	Upstream	14331	H001, H003

	OA-LA17	5	18157188	A,G	g43427; Cytochrome P450 71A4-like	21	24	123	H001(53), H002 (36), H003 (11), H004(6)	Upstream	26	H001, H003
	OA-LA20	5	18141916	A,C	g43426; Cytochrome P450 71A4-like	14	23	117	H001(73), H002 (25), H003 (8)	Upstream	43	H001
	OA-LA33	5	18179456	T,A	g43429 and g43430.t1; Cytochrome P450 71A2-like	18	18	122	H001(85) H002(21)	Downstream	5875	
	OA-LA35	6	84242096	T,A	g53664; Probable calcium-binding protein CML25	52	20	120	H001(69)	Upstream	2183	H001
	OA-LA5	7	66993411	G,A	g57141; Cytochrome P450 710A11-like	26	16	121	H001(70) H002(27)	Upstream	608	
**Oil Content**	OC2	2	65505220	T,C	g28700; Pentatricopeptide repeat-containing protein At4g20740	83	16	122	H001, H002(3), H003(3), H004(3)	Upstream	71	H001, H096
	OC4	5	5164369	C,T	g42440; Purple acid phosphatase 27	11	6	123	H001(82), H002 (14),H003 (9), H004 (7), H005(3), H006(2)	Gene within Haplotype	0	H001, H002

	OC6	9	18518779	A,G	g53664; Probable UDP-N-acetylglucosamine--peptide N-acetylglucosaminyltransferase SPINDLY isoform X1	16	15	122	H001(107)	Gene within Haplotype	0	H006, H007,H009,H001
	OC9	9	79566941	G,A	g67871; Retrovirus-related Pol polyprotein from transposon TNT 1-94	3	2	122	H01(108), H02 (8), H03 (7)	Upstream	2428	H001, H003
	OC1	11	76393484	G,A	g16920; Protein BIG GRAIN 1-like E	71	40	116	H001(30), H002 (7),H003 (6),H004(3), H005(3), H006	Gene within Haplotype	0	
	OC8	11	76395157	C,T	g16920; Protein BIG GRAIN 1-like E	67	39	116	H001(33), H02 (7), H003 (6), H004(3), H005(3), H006(3)	Gene within Haplotype	0	H001, H040,H058
**Number of Primary Branches**	PB4	5	5018372	A,C	g42435; Acetolactate synthase 1, chloroplastic	29	14	122	H001(94)	Upstream	3704	H001, H028
	PB9	5	3333942	T,C	g42292; F-box/LRR-repeat protein 14-like isoform X1	26	15	122	H001(96)	Upstream	688	H001, H008

	PB1	12	63076322	C,T	g20942. and g20943;<br>Putative pentatricopeptide repeat-containing protein At1g12700, mitochondrial	31	17	119	H001(86), H002(3)	Gene within Haplotype	0	
**Plant Height**	PH3	2	10029119	A,G	g25891;<br>Vignain-like	63	13	122	H001(54), H002 (6)	Downstream	1031	H001, H048
	PH4	3	15050693	G,A	g32062;<br>Trafficking protein particle complex subunit 6B (TRAPPC6B)	20	19	122	H001 (103)	Downstream	872	

Click here to access/download;Figure;Fig 1.jpg

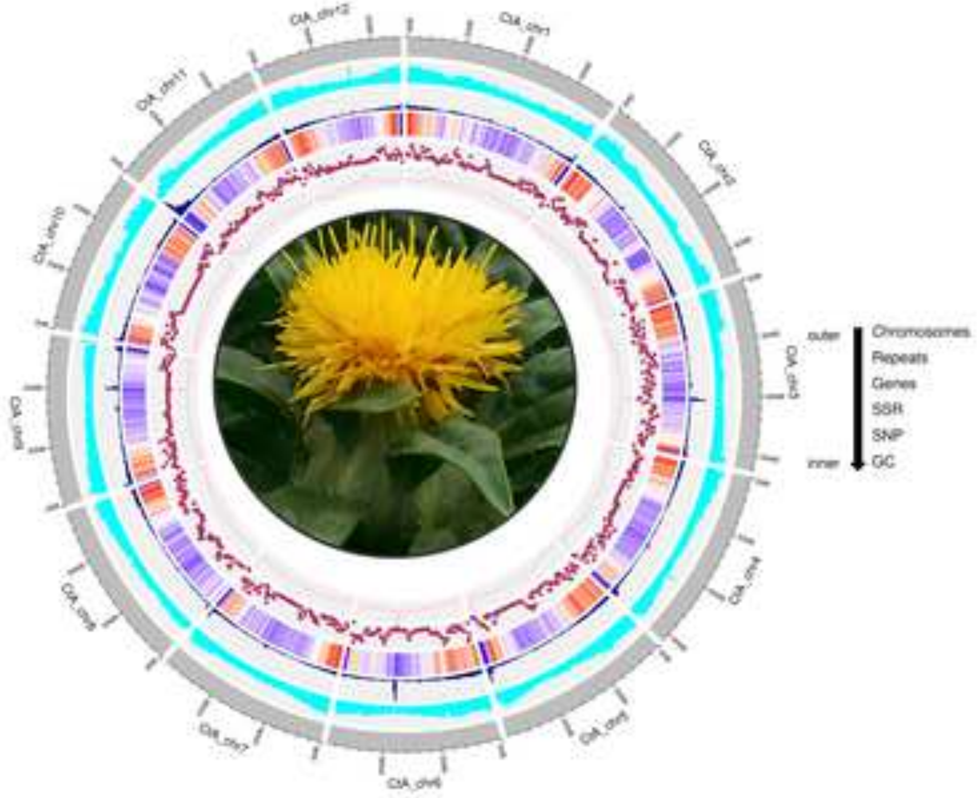

Click here to access/download;Figure;Fig 2.jpg

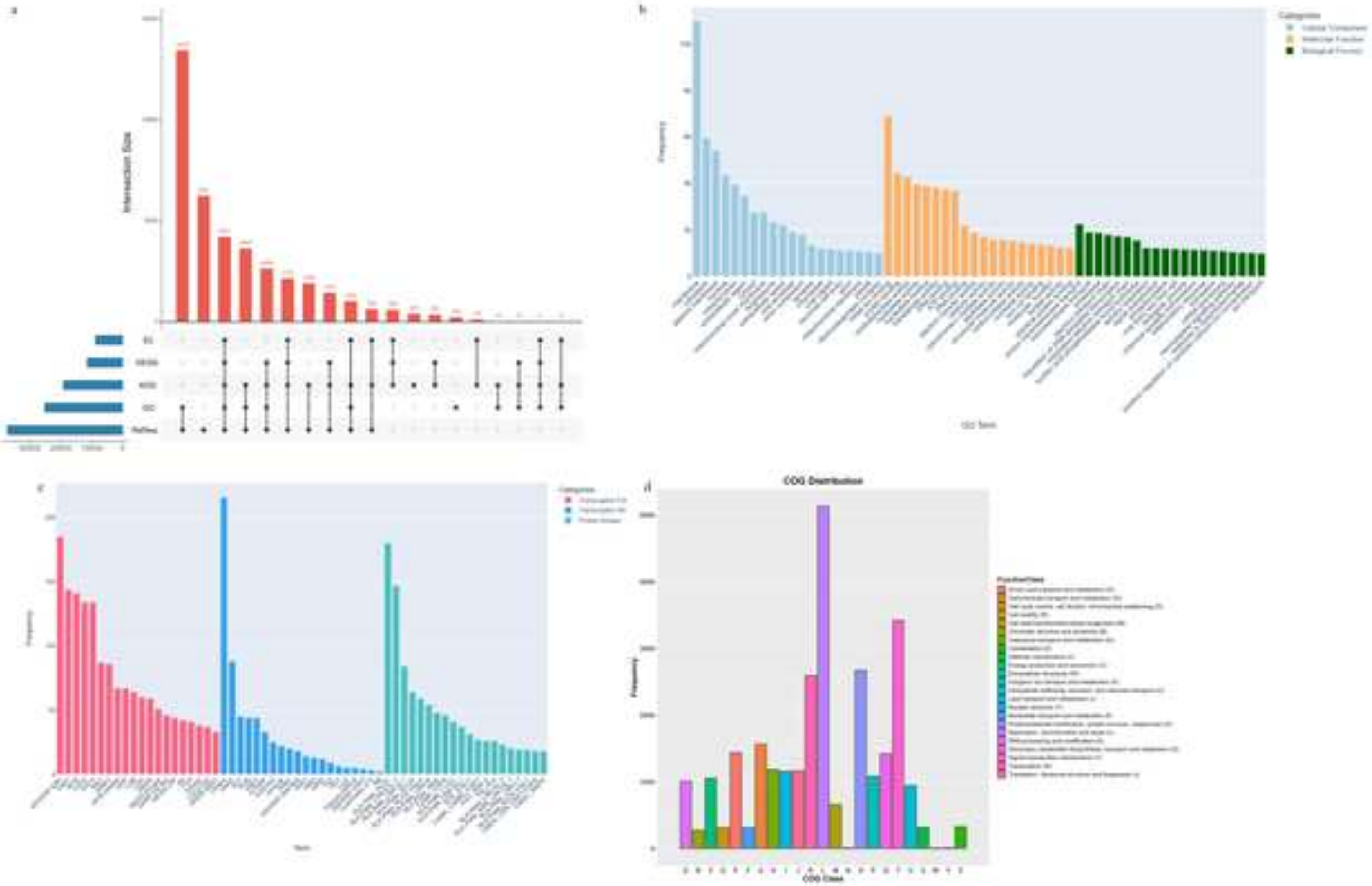

Figure
Click here to access/download;Figure;Fig 3 (1).jpg

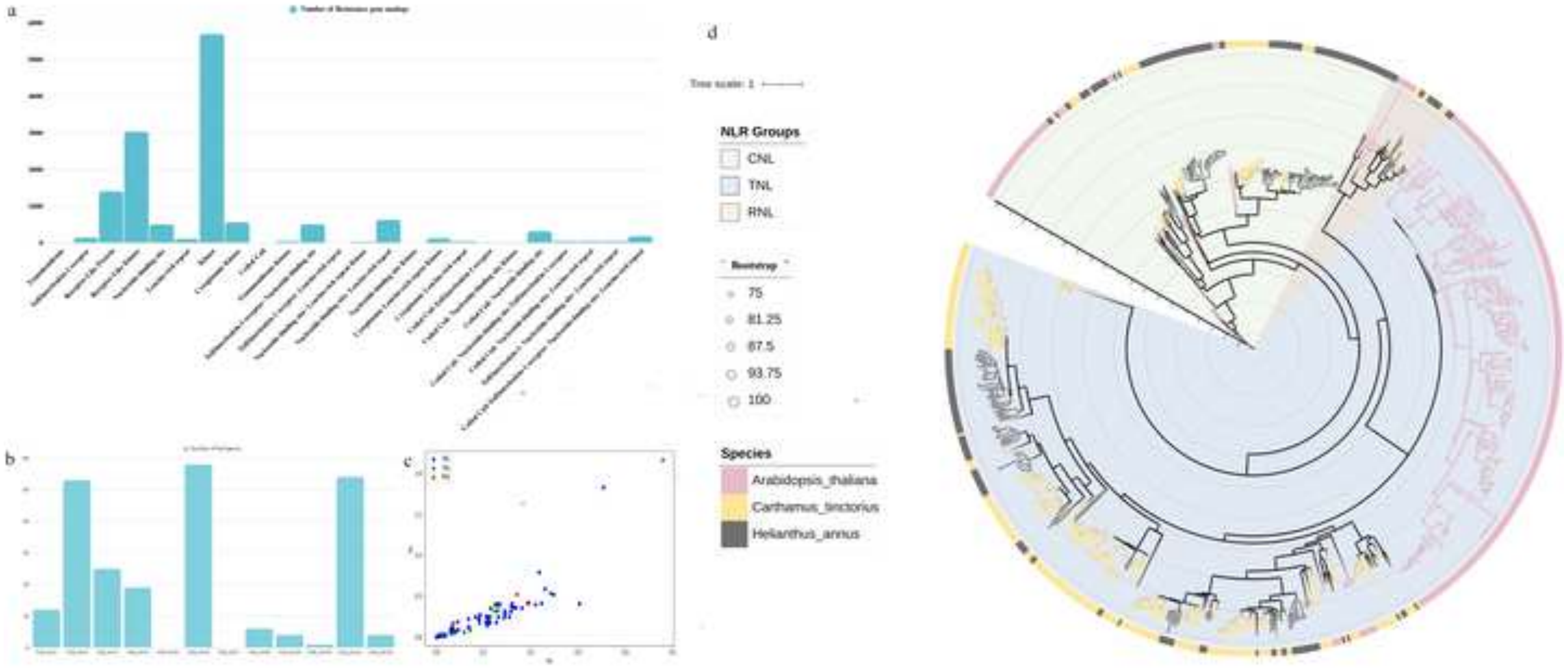

Figure

Click here to access/download;Figure;Figure 5 (3).jpg

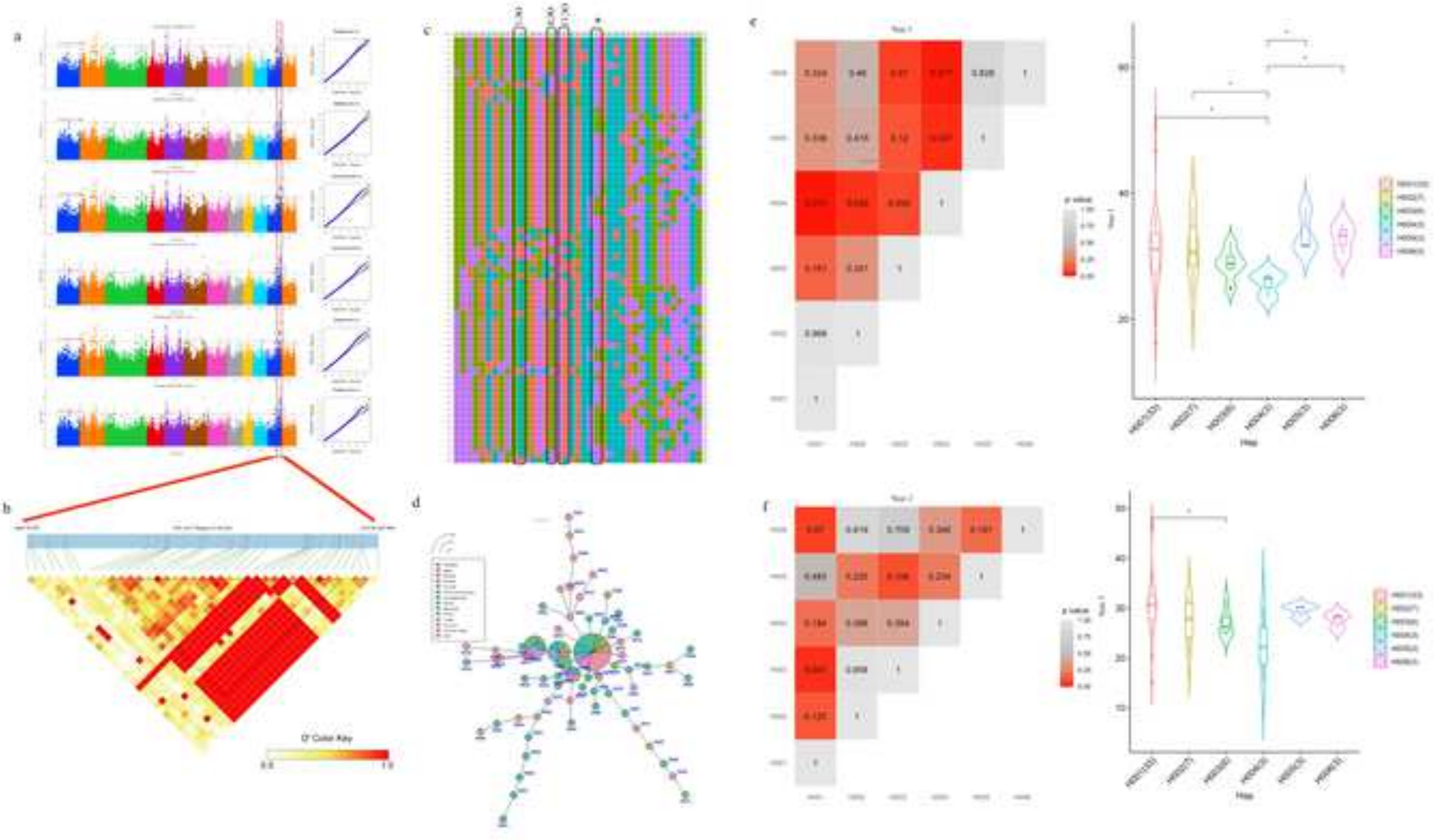

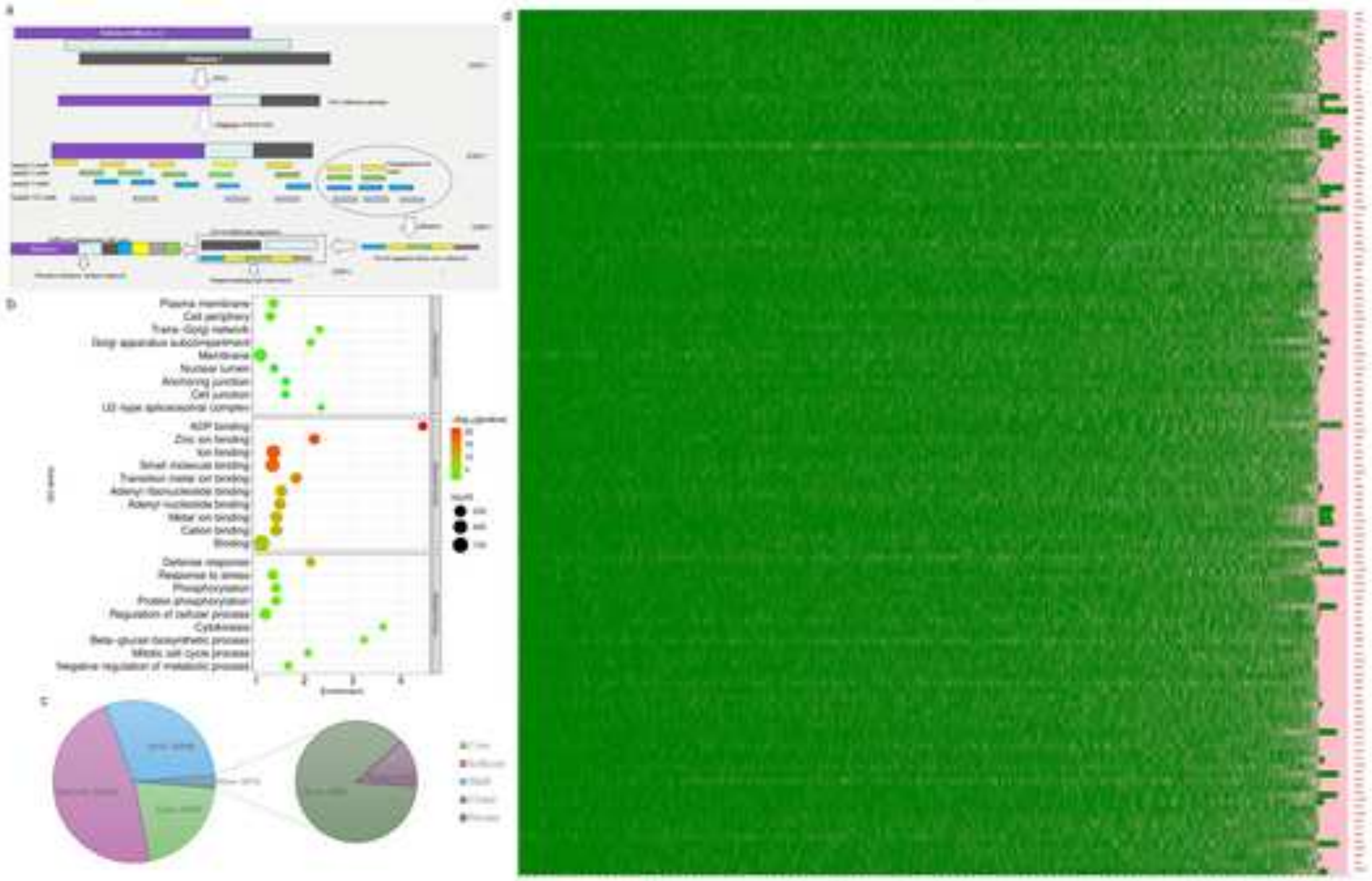
Plasma membrane
Cell periphery
Trans-Golgi network
Golgi apparatus subcompartment
Membrane
Nuclear lumen
Anchoring junction
Cell junction
U2-type spliceosomal complex
ADP binding
Zinc ion binding
Ion binding
Small molecule binding
Transition metal ion binding
Adenyl ribonucleotide binding
Adenyl nucleotide binding
Metal ion binding
Cation binding
Binding
Defense response
Response to stress
Phosphorylation
Protein phosphorylation
Regulation of cellular process
Cytokinesis
Beta-glucan biosynthetic process
Mitotic cell cycle process
Negative regulation of metabolic process

Figure

Click here to access/download;Figure;Fig 4 (2).jpg

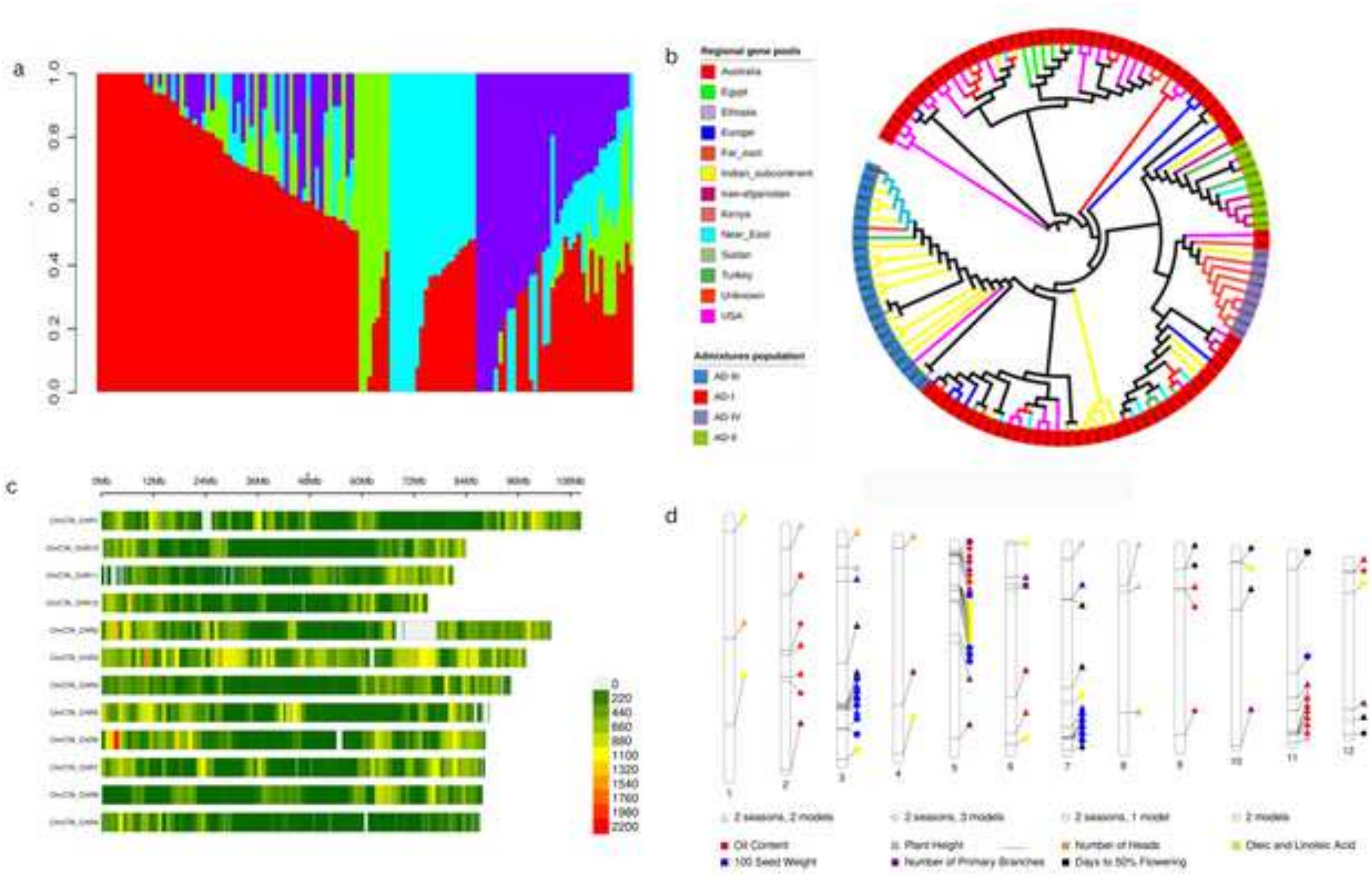

Click here to access/download
Supplementary Material
final Supplementary information_du.docx

 Click here to access/download;Personal Cover;cover letter.docx

Dear Dr. Scott Edmunds,

Editor-in-Chief

GigaScience

We are submitting our manuscript entitled "**Improved reference assembly and core collection re-sequencing to facilitate exploration of important agronomical traits for the improvement of oilseed crop, *Carthamus tinctorius* L.**", to be considered for publication in your esteemed journal. This is the second revision of the manuscript, and we have carried out an extensive revision based on the input from reviewers. A detailed response to the reviewer's query has been provided. In this process, several new analyses have been included in the manuscript. We acknowledge a significant improvement in the manuscript and our understanding of the subject as a result of the exhaustive reviewing process.

In this round of reviewing, Reviewer-2 has suggested multiple revisions and a few wet lab validations. His suggestions have been helpful in improving the manuscript. We have carried out additional extensive analysis, including structural variant analysis, cross-species analysis, and functional exploration of resistance genes, gene enrichment analysis of pangenome, and revised various portions of the manuscript. He has raised queries related to the validation of identified QTNs and associated SNPs, which were addressed by carrying out extensive KASP analysis. In total, 20 SNPs have been validated, and these results are now part of this manuscript.

However, the reviewer also suggested experimental verifications of identified candidate genes using gene knockout, overexpression, or allelic function analysis, which are time-consuming experiments and, hence, are beyond the scope of this manuscript. This manuscript is a discovery study, and validation of identified genes is a long, drawn-out process, which is part of our future projects and will take years to complete. In the first review process, earlier reviewers have also accepted that such elaborate validation experiments are not possible and have asked for a detailed bioinformatic analysis, which prompted us to carry out haplo-pheno analysis. We request an understanding of our reasonable limitations while determining the fate of this manuscript.

Accessible links for the submitted data are given below:

1. The raw sequencing data generated for the Genome Assembly has been deposited at NCBI under the BioProject PRJNA1089929 (https://dataview.ncbi.nlm.nih.gov/object/PRJNA1089929?reviewer=fpkm54q3j61l3bv8vtpcssecj7).
2. The genome assembly, functional annotation, transcript and protein sequences, Bionano Optical Maps and VCF file for the SNP data, are available at the Safflower Genome Resource (SGR: http://51.21.157.20:3002/downloads), developed by us.
3. Pangenome assembly and its annotation are also available at Safflower Genome Resource (SGR: http://51.21.157.20:3002/downloads).

All authors are aware of the submission and have given their consent. No AI-assisted technology is used in drafting the Manuscript. The contents of the manuscript have not been submitted or published elsewhere.

We would greatly appreciate a positive consideration of our manuscript and the opportunity to contribute to the scientific discourse in your esteemed journal. We look forward to your feedback and guidance.

Warm regards,

Prof. Shailendra Goel and Prof. Arun Jagannath
(Joint corresponding authors)
Department of Botany
University of Delhi
India
shailendragoel@gmail.com; jagannatharun@yahoo.co.in

Detailed response to the reviewer's query:

	Queries	Answers
	**Reviewer #1:**	
	Compared to the first version, this version has made significant improvements. Firstly, we can believe that the genome quality of this version should be relatively good, and the number of genes annotated this time is reasonable.	We thank the reviewer for his efforts and time spent on improving this manuscript. We acknowledge that his suggestion has not only improved the manuscript but also have enhanced our understanding of the subject.
1	However, **I am still concerned that the content of this article is too rich, making it difficult for readers to grasp the key points of the article, and should be appropriately reduced. My suggestion is that the authors should focus on in-depth analysis of population genetics of genomes and agronomic traits, such as the section on gene family identification, which can be omitted.** More seriously, the author should strengthen their study of relevant theories such as molecular biology or genetics. The description of some concepts is clearly incorrect, which will mislead the author's understanding of genomics. Two more serious issues are as follows: Firstly, the author mentioned in multiple places that compared to the previous version of the safflower genome, the author has identified a larger number of gene family members (such as PAL, FAD2). I think the author should be cautious when drawing conclusions, whether the author is identifying more gene transcripts or more genes? Because the author mistakenly identified AT2G5890.1 and AT2G5890.2 as two paralogs in lines 246 and 247, which is a clear molecular biology concept. They are only two different transcripts of one gene. Furthermore, FAD and FAD2 are not part of the same gene family. Therefore, the number of genes mentioned in the article may be different transcripts. Suggest that the author annotate genes at the chromosome level and generate gff3 files, which can be referenced from Arabidopsis annotation files, to clearly identify the number of genes and transcripts. In addition, the understanding of some biological processes is also incorrect. Line 502, "PAL catalyses the first step of the flavonoid biosynthetic pathway." Actually, Phenylalanine ammonia-lyase (PAL, EC 4.3.1.24) catalyses the deamination of phenylalanine to cinnamate and ammonia, the first step of the phenylpropanoid pathway, rather than flavonoid biosynthetic pathway.	We agree that the manuscript is becoming more heterogeneous, and especially because we have been asked to add more analyses. In light of these changes, we do agree with your recommendation to focus more on the in-depth study of population genetics and agronomic traits. We have revised the manuscript to emphasize these aspects more strongly. Accordingly, we have removed the section on gene family identification, as it was peripheral to the central objectives of our study.<br>This adjustment will also provide us with additional space to accommodate the changes suggested by Reviewer 2.
2	In lines 217 to 220, the author's understanding of Ka/Ks is incorrect. A ratio less than 1 represents purifying selection, while the opposite represents positive selection. But the author's description is exactly the opposite.	Thank you for pointing this out. Inadvertently, it was written the other way around; we have corrected it.

3	Also, the availability and reproducibility of genomic data are crucial, as mentioned by the author in the Safflower Genome Resource (SGR) database （http://13.60.187.179:3002/）And NCBI BioProject PRJNA1089929 cannot be accessed. The author should open access permissions or make the original data public to ensure that the data can be duplicated.	Because of the restriction on data traffic by the University of Delhi, where we work, we have set up a server on Amazon Web Services. While updating it with some information asked by the data management team, we had to restart it, which changed the URL. The previous URL (http://13.60.187.179:3002/) is not active, and a new URL (http://51.21.157.20:3002) was provided to the Gigascience Database management team, who assured that it will be provided to the reviewers. The new link has been provided in the manuscript. We have now made the server static instead of dynamic to avoid such a change in the address. Additionally, the NCBI BioProject **PRJNA1089929** is currently under controlled access as per NCBI policy and will be made fully public immediately upon the acceptance of the manuscript. In the meantime, to facilitate the review process, the data can be checked via the reviewer link. This reviewer link does not provide access to the data and is only useful to check whether the said data is uploaded on the NCBI server.<br>Data view link for the reviewer: **https://dataview.ncbi.nlm.nih.gov/object/PRJNA1089929?reviewer=fpkm54q3j61l3bv8vtpcssecj7**
	**Reviewer #2:**	
	This article presents an improved genome assembly of safflower (Carthamus tinctorius L.) (Safflower_A2) and resequenced the global core germplasm using this assembly, conducting GWAS and pan-genome analyses. The authors assert that this assembly is superior to previous versions regarding genome continuity, completeness, and annotation quality. They also employed these resources to identify genes related to significant agronomic traits such as disease resistance, oil quality, oil content, and pigment synthesis. Additionally, a high-density genetic linkage map was constructed, and pan-genome analysis revealed genomic diversity.	We thank the reviewer for his insightful comments and acknowledge his contribution towards improving the manuscript. We have made every effort to address his concern and hope that we could improve the manuscript to his satisfaction
	**Queries regarding comparison with earlier genomes**	
1	The article states that the genome size is 1.15 Gb, the BUSCO completeness is 97.9%, and the long-read mapping rate is 99.29%. However, in comparing it to the previous two genomes (Anhui 1 and Chuanhonghua 1), although the N50 value is higher, the specific methods and results of **the repetitive sequence** processing are not detailed. The collinearity analysis with the published safflower genomes (Anhui 1 and Chuanhonghua 1) provides only partial results. A complete genome alignment map (such as SynMap) and statistics on structural variations (such as inversions and translocations) must be included.	Thank you for your insightful comments and suggestions. In response, we have provided a more detailed description of **the repetitive sequence** annotation in the methods section, and we have made some required changes in the results section and supplementary Table S10, to enhance the readability.<br>We have expanded the comparison with the previously published genomes (Anhui 1 and Chuanhonghua 1), as suggested. We incorporated the detection of structural variations, including translocations, deletions, inversions, and duplications (Supplementary fig S7). Notably, we observed a large translocation event in Chuanhonghua 1, which likely reflects a mis-assembly in that genome. We have made appropriate changes in Results (Lines 125--137) and Discussion (Lines 424-451).
	**Queries regarding NLR genes**	

2	The phylogenetic tree of NLR genes mentioned in the article illustrates the branch patterns of TNL and CNL, but no comparison with other species (such as sunflower and Arabidopsis) is provided, and there is a lack of collinearity analysis. Cross-species comparisons should be included to support evolutionary conclusions.	We have expanded our analysis to include cross-species comparisons of NLR genes. Specifically, we have incorporated both phylogenetic and collinearity analyses involving NLRs from *Helianthus annuus* (sunflower) and *Arabidopsis thaliana*. The updated phylogenetic tree includes representative TNL and CNL genes from these species, providing clearer insights into the evolutionary relationships and lineage-specific expansions. This analysis is now presented in Figure 3e, results and discussion (Lines 484-495).<br><br>Additionally, a collinearity analysis was performed using MCScanX to identify conserved syntenic regions of NLR genes between safflower and the two species. The results are shown in Supplementary Figure S13 and are described in the revised results (236-244) and discussion section (Line 495-503).
3	This study identified 236 NLR genes but did not discuss their potential targets in safflower disease resistance breeding. Combining the functional analogies of known disease resistance genes (such as Xa21 in rice and Sr35 in wheat) is suggested to propose research directions for candidate genes.	We have now included a discussion of potential target NLR genes in safflower with reference to their possible roles in disease resistance breeding. This comparative functional analysis and the corresponding candidate genes are now detailed in Supplementary Table S16, and the relevant discussion has been added to the revised manuscript (Lines: 245-252; 504-522).
4	The evolutionary tree in Figure 3 does not indicate the branch support rate (such as bootstrap values), which should be added to enhance credibility.	We have revised the evolutionary tree to improve its clarity and interpretability. Specifically, we reconstructed the phylogenetic tree using updated alignments for the three species and have now included bootstrap values to indicate branch support and increase the reliability of the inferred relationships. These changes are reflected in the updated **Figure 3e** and have been described accordingly in the revised figure legend and Results section.
**Queries regarding Pan-genome**		
5	The article notes that the pangenome contains 11,479 new transcripts but does not deeply analyze the roles of these genes in adaptation or domestication. It is suggested to link functional enrichment results (such as stress response-related genes) to explore their guiding value for region-specific breeding.	We have performed the functional enrichment analysis of the 11,479 new transcripts and identified 238 candidate genes showing significant enrichment in biological processes related to stress response, metabolic pathways, and traits relevant to adaptation. These results are now detailed in Figure 6b, Supplementary Table S28 and S29, and the corresponding discussion has been expanded in the manuscript to highlight the potential of these genes for guiding region-specific breeding in safflower (lines 386-414; 628-667). Since our study does not incorporate the wild genome, the exploration of the domestication related genes was not possible. Although, we agree with the importance of such an analysis and ensure to include this suggestion in our future work.
6	Pan-genome construction: It is necessary to provide the parameters of MaSuRCA assembly and the specific criteria for filtering contaminant sequences (such as the E-value threshold of BLASTn).	Appropriate changes have been made to the methods section of the manuscript (Lines 858-859).

Queries regarding GWAS and candidate gene		
7	GWAS analysis: The article used multiple models (MLMM, FarmCPU, BLINK) to screen QTNs but did not mention the multiple testing correction method (such as Bonferroni or FDR). It is essential to clarify the rationale for determining the significance threshold (p < 0.0001).	We have used a significant threshold of p < 0.0001 based on various GWAS studies carried out in different crops (Lee et al., 2023; Gyawali et al., 2019; Rabieyan et al., 2022). Additionally, only QTNs identified in two growing seasons have been retained for downstream analysis, thereby improving the reliability of these associations. Appropriate changes have been made in the methods section to clearly indicate this information (Line 821-822.)
8	QTN validation: Have the 81 identified QTNs been validated in independent populations or functional experiments? Discussing the potential limitations of unverified QTNs or proposing subsequent validation plans is suggested.	We would like to thank the reviewer for this important comment. The current manuscript is a continuous piece of work; we are reporting where we are now, and many of the things suggested by the reviewer are already part of our future plans. Following further data curation and parameter optimization, the number of detected QTNs has increased from 81 to 96 and has been updated in the manuscript. We have validated various QTNs and SNPs using KASP assay. We have experimentally validated 10 of these QTNs using KASP assays across a diverse panel of safflower lines. These validated markers showed consistent associations with the traits of interest, supporting their potential utility in breeding applications. We have also analysed 10 SNPs closer to various genes of interest identified by our analysis (mentioned in an answer below). We have now included these results in the manuscript to address the reviewers' concerns.
9	The ADMIXTURE analysis shows a weak population structure, but whether PCA or kinship matrices were included as covariates in the GWAS is unclear. It is necessary to clarify whether the model accounted for population stratification.	In this study, we utilized the GAPITv3 pipeline (Wang & Zhang, 2021), which performs both principal component analysis (PCA) and kinship matrix calculation (using VanRaden method) internally. The number of principal components to include was specified in the command using the parameter PCA.total = 4, which was decided based on our population structure analysis (Line 820).
11	The text does not clearly distinguish the differences between the "multi-locus models" of FarmCPU and BLINK. The core assumptions and applicable scenarios of each model should be elaborated on.	We have now included a paragraph in the revised manuscript to clarify the core assumptions and implications of different models in our discussion section. (Lines554-564).

12	The article identifies multiple candidate genes associated with agronomic traits (such as BIG GRAIN 1-like, PPR proteins, CYP57, etc.), but lacks experimental verification (such as gene knockout, overexpression, or allelic function analysis). Relying solely on bioinformatics predictions and support from existing literature is inadequate to confirm the actual functions of these genes, especially in safflower, a non-model plant, where gene function conservation may vary.	As we have mentioned above that like any other research program, this is an ongoing program in our lab. Many of the things suggested are part of our future plans. As a part of our validation efforts, 20 SNPs have already been validated through KASP assays and these results are now part of this manuscript. Other suggested experimental verifications (gene knockout, overexpression, or allelic function analysis) are time-consuming experiments and are beyond the scope of this manuscript. Earlier reviewer has also accepted that such elaborate experiments are not possible and has asked for a detailed bioinformatic analysis, which prompted us to carry out haplopheno analysis. We request the reviewer to understand our limitations. We have done a lot of work, including KASP analysis and other detailed bioinformatic assays for the manuscript, and request for an understanding while determining the fate of this manuscript.
13	The screening of candidate genes relies on a fixed threshold of "7 kb upstream and downstream", without considering gene function annotations or expression data (such as RNA-seq), resulting in a high risk of false positives.	The choice of 7 kb is based on the estimated LD decay analysis. The identified genes were chosen after considering the gene function annotation only. Kindly refer to the Methods section (Lines 823-828).<br>In addition, we conducted haplotype–phenotype association analyses to investigate the relationship between allele variation and trait expression. KASP marker analysis was also performed to validate the genetic regions surrounding selected QTNs.<br>While we recognize that gene expression data (e.g., from RNA-seq) can provide additional supporting evidences, those analyses are currently in progress and will be incorporated in future studies aimed at functional validation.
**Minor**		
1	Some references lack a DOI (e.g., References 4 and 5), and the format should be standardized according to the journal's requirements.	References 4 5 etc are web-based resources and, as such, do not have DOIs assigned. As per the journal’s guidelines, all web links and URLs have been included as numbered references in the reference list. Each web reference now contains the full site title, the URL, formatted according to the journal’s requirements.<br><br>We have reviewed and standardized the entire reference list accordingly in the revised manuscript.
2	The readability of the figures and tables is poor. The labels in the Circos plot of Figure 1 are too small. Enlarging the chromosome numbers and adding a color legend are recommended. The clarity of Figure 6b is inadequate.	We would like to clarify that high-quality figures in SVG format—including improved versions of Figures—have already been submitted to the GigaDB server and communicated to the GigaScience editorial team in an earlier stage of the submission process. The readability issues noted may be specific to the compressed PNG images embedded in the manuscript PDF. The SVG versions provide full clarity and scalability. We suggest that the reviewer access the SVG versions of the figures.

		GigaScience database team ensures the quality of all images.
3	In Table 1, the unit for "Genome size (k-mer)" should be Gb instead of being unitless; "BUSCO 97.90%" should be fully classified (e.g., "97.9% complete BUSCOs").	Suggested changes have been made to the manuscript.
4	Some software mentioned in the methods (such as BRAKER3 and EDTA) do not indicate the version number, which should be included to ensure reproducibility.	Suggested changes have been made to the manuscript.
