# Supplementary material for: Improved reference assembly and core collection resequencing to facilitate exploration of important agronomical traits for the improvement of oilseed crop, Carthamus tinctorius L
Source: Gigascience. 2025 Dec 11;15:giaf151. doi: 10.1093/gigascience/giaf151 (PMC12888819; doi:10.1093/gigascience/giaf151)
Supplement: giaf151_Reviewer_1_Report_Original_Submission — Zhihua Wu -- 2/21/2025 [file giaf151_reviewer_1_report_original_submission.pdf]

**Reviewer Report**

**Title: Improved reference assembly and core collection re-sequencing to facilitate exploration of important agronomical traits for the improvement of oilseed crop, Carthamus tinctorius L.**

**Version: Original Submission** **Date:** 2/21/2025

**Reviewer name: Zhihua Wu**

**Reviewer Comments to Author:**

Compared to the first version, this version has made significant improvements. Firstly, we can believe that the genome quality of this version should be relatively good, and the number of genes annotated this time is reasonable. However, I am still concerned that the content of this article is too rich, making it difficult for readers to grasp the key points of the article, and should be appropriately reduced. My suggestion is that the authors should focus on in-depth analysis of population genetics of genomes and agronomic traits, such as the section on gene family identification, which can be omitted. More seriously, the author should strengthen their study of relevant theories such as molecular biology or genetics. The description of some concepts is clearly incorrect, which will mislead the author's understanding of genomics. Two more serious issues are as follows:

Firstly, the author mentioned in multiple places that compared to the previous version of the safflower genome, the author has identified a larger number of gene family members (such as PAL, FAD2). I think the author should be cautious when drawing conclusions, whether the author is identifying more gene transcripts or more genes? Because the author mistakenly identified AT2G5890.1 and AT2G5890.2 as two paralogs in lines 246 and 247, which is a clear molecular biology concept. They are only two different transcripts of one gene. Furthermore, FAD and FAD2 are not part of the same gene family. Therefore, the number of genes mentioned in the article may be different transcripts. Suggest that the author annotate genes at the chromosome level and generate gff3 files, which can be referenced from Arabidopsis annotation files, to clearly identify the number of genes and transcripts. Secondly, in lines 217 to 220, the author's understanding of Ka/Ks is incorrect. A ratio less than 1 represents purifying selection, while the opposite represents positive selection. But the author's description is exactly the opposite.

In addition, the understanding of some biological processes is also incorrect. Line 502, "PAL catalyses the first step of the flavonoid biosynthetic pathway." Actually, Phenylalanine ammonia-lyase (PAL, EC 4.3.1.24) catalyzes the deamination of phenylalanine to cinnamate and ammonia, the first step of the phenylpropanoid pathway, rather than flavonoid biosynthetic pathway.

Also, the availability and reproducibility of genomic data are crucial, as mentioned by the author in the Safflower Genome Resource (SGR) databaseï¼ˆ http://13.60.187.179:3002/ ï¼‰And NCBI BioProject PRJNA1089929 cannot be accessed. The author should open access permissions or make the original data public to ensure that the data can be duplicated.

**Methods**

Are the methods appropriate to the aims of the study, are they well described, and are necessary controls included? Choose an item.

**Conclusions**

Are the conclusions adequately supported by the data shown? Choose an item.

**Reporting Standards**

Does the manuscript adhere to the journal's guidelines on minimum standards of reporting? Choose an item.

Choose an item.

**Statistics**

Are you able to assess all statistics in the manuscript, including the appropriateness of statistical tests used? Choose an item.

**Quality of Written English**

Please indicate the quality of language in the manuscript: Choose an item.

**Declaration of Competing Interests**

Please complete a declaration of competing interests, considering the following questions:

- Have you in the past five years received reimbursements, fees, funding, or salary from an organisation that may in any way gain or lose financially from the publication of this manuscript, either now or in the future?
- Do you hold any stocks or shares in an organisation that may in any way gain or lose financially from the publication of this manuscript, either now or in the future?
- Do you hold or are you currently applying for any patents relating to the content of the manuscript?
- Have you received reimbursements, fees, funding, or salary from an organization that holds or has applied for patents relating to the content of the manuscript?
- Do you have any other financial competing interests?
- Do you have any non-financial competing interests in relation to this paper?

If you can answer no to all of the above, write 'I declare that I have no competing interests' below. If your reply is yes to any, please give details below.

I declare that I have no competing interests

I agree to the open peer review policy of the journal. I understand that my name will be included on my report to the authors and, if the manuscript is accepted for publication, my named report including any attachments I upload will be posted on the website along with the authors' responses. I agree for my report to be made available under an Open Access Creative Commons CC-BY license (http://creativecommons.org/licenses/by/4.0/). I understand that any comments which I do not wish to be included in my named report can be included as confidential comments to the editors, which will not be published.

Choose an item.

To further support our reviewers, we have joined with Publons, where you can gain additional credit to further highlight your hard work (see: https://publons.com/journal/530/gigascience). On publication of this paper, your review will be automatically added to Publons, you can then choose whether or not to claim your Publons credit. I understand this statement.

Yes Choose an item.
