# Supplementary material for: Improved reference assembly and core collection resequencing to facilitate exploration of important agronomical traits for the improvement of oilseed crop, Carthamus tinctorius L
Source: Gigascience. 2025 Dec 11;15:giaf151. doi: 10.1093/gigascience/giaf151 (PMC12888819; doi:10.1093/gigascience/giaf151)
Supplement: giaf151_Reviewer_1_Report_Revision_1 — Zhihua Wu -- 7/12/2025 [file giaf151_reviewer_1_report_revision_1.pdf]

Reviewer Report

Title: Improved reference assembly and core collection re-sequencing to facilitate exploration of important agronomical traits for the improvement of oilseed crop, Carthamus tinctorius L.

Version: Revision 1 **Date:** 7/12/2025

Reviewer name: Zhihua Wu

Reviewer Comments to Author:

Compared to the previous version, this version has made improvements, but there are still some points worth paying attention to, especially the scientific presentation and description of data.

1. The article does not have any experimental verification about associated genes, and it would be meaningful to verify their potential function of genes, such as qRT-PCR.
2. Line123~124: Its annotation identified 205 protein-coding genes which were higher than the 127 cp genes reported earlier [15]. To my knowledge, most chloroplasts have around 80 protein coding genes. The result or description of this sentence is incorrect and may mislead readers, indicating that the author is unclear about the characteristics of the chloroplast genome.
3. The article conducted KASP validation on the markers associated with agronomic traits, but only the primers were seen and the results of KASP analysis were not presented.
4. In the methodology, the author should supplement the methods for identifying MTAs or QTNs.
5. The author's literature references are not comprehensive enough. In recent years, there have been multiple literature reports on the application and genomic research progress of safflower, such as, "Current advances of Carthamus tinctorius L.: a review of its application and molecular regulation of flavonoid biosynthesis" And 'Research Progress of Genomics Applications in Secondary Metabolites of Medicinal Plants: A Case Study in Safffower'.

In short, the author needs to provide a very objective description and summary of the data and results, otherwise it will mislead readers.

Methods

Are the methods appropriate to the aims of the study, are they well described, and are necessary controls included? Choose an item.

Conclusions

Are the conclusions adequately supported by the data shown? Choose an item.

Reporting Standards

Does the manuscript adhere to the journal’s guidelines on minimum standards of reporting? Choose an item.

Choose an item.

Statistics

Are you able to assess all statistics in the manuscript, including the appropriateness of statistical tests used? Choose an item.

Quality of Written English

Please indicate the quality of language in the manuscript: Choose an item.

Declaration of Competing Interests

Please complete a declaration of competing interests, considering the following questions:

- Have you in the past five years received reimbursements, fees, funding, or salary from an organisation that may in any way gain or lose financially from the publication of this manuscript, either now or in the future?
- Do you hold any stocks or shares in an organisation that may in any way gain or lose financially from the publication of this manuscript, either now or in the future?
- Do you hold or are you currently applying for any patents relating to the content of the manuscript?
- Have you received reimbursements, fees, funding, or salary from an organization that holds or has applied for patents relating to the content of the manuscript?
- Do you have any other financial competing interests?
- Do you have any non-financial competing interests in relation to this paper?

If you can answer no to all of the above, write 'I declare that I have no competing interests' below. If your reply is yes to any, please give details below.

I declare that I have no competing interests

I agree to the open peer review policy of the journal. I understand that my name will be included on my report to the authors and, if the manuscript is accepted for publication, my named report including any attachments I upload will be posted on the website along with the authors' responses. I agree for my report to be made available under an Open Access Creative Commons CC-BY license (http://creativecommons.org/licenses/by/4.0/). I understand that any comments which I do not wish to be included in my named report can be included as confidential comments to the editors, which will not be published.

Choose an item.

To further support our reviewers, we have joined with Publons, where you can gain additional credit to further highlight your hard work (see: https://publons.com/journal/530/gigascience). On publication of this paper, your review will be automatically added to Publons, you can then choose whether or not to claim your Publons credit. I understand this statement.

Yes Choose an item.
