# Supplementary material for: Improved reference assembly and core collection resequencing to facilitate exploration of important agronomical traits for the improvement of oilseed crop, Carthamus tinctorius L
Source: Gigascience. 2025 Dec 11;15:giaf151. doi: 10.1093/gigascience/giaf151 (PMC12888819; doi:10.1093/gigascience/giaf151)
Supplement: giaf151_Reviewer_2_Report_Original_Submission — Cheng Dai -- 3/9/2025 [file giaf151_reviewer_2_report_original_submission.pdf]

Reviewer Report

Title: Improved reference assembly and core collection re-sequencing to facilitate exploration of important agronomical traits for the improvement of oilseed crop, Carthamus tinctorius L.

Version: Original Submission **Date:** 3/9/2025

Reviewer name: Cheng Dai

Reviewer Comments to Author:

This article presents an improved genome assembly of safflower (Carthamus tinctorius L.) (Safflower_A2) and resequenced the global core germplasm using this assembly, conducting GWAS and pangenome analyses. The authors assert that this assembly is superior to previous versions regarding genome continuity, completeness, and annotation quality. They also employed these resources to identify genes related to significant agronomic traits such as disease resistance, oil quality, oil content, and pigment synthesis. Additionally, a high-density genetic linkage map was constructed, and pangenome analysis revealed genomic diversity.

Major commentsï¼š

1. The article states that the genome size is 1.15 Gb, the BUSCO completeness is 97.9%, and the long-read mapping rate is 99.29%. However, in comparing it to the previous two genomes (Anhui_1 and Chuanhonghua 1), although the N50 value is higher, the specific methods and results of the repetitive sequence processing are not detailed. The collinearity analysis with the published safflower genomes (Anhui 1 and Chuanhonghua 1) provides only partial results. A complete genome alignment map (such as SynMap) and statistics on structural variations (such as inversions and translocations) must be included.
2. The phylogenetic tree of NLR genes mentioned in the article illustrates the branch patterns of TNL and CNL, but no comparison with other species (such as sunflower and Arabidopsis) is provided, and there is a lack of collinearity analysis. Cross-species comparisons should be included to support evolutionary conclusions.
3. The article identifies multiple candidate genes associated with agronomic traits (such as BIG GRAIN 1-like, PPR proteins, CYP57, etc.), but lacks experimental verification (such as gene knockout, overexpression, or allelic function analysis). Relying solely on bioinformatics predictions and support from existing literature is inadequate to confirm the actual functions of these genes, especially in safflower, a non-model plant, where gene function conservation may vary.
4. This study identified 236 NLR genes but did not discuss their potential targets in safflower disease resistance breeding. Combining the functional analogies of known disease resistance genes (such as Xa21 in rice and Sr35 in wheat) is suggested to propose research directions for candidate genes.
5. The article notes that the pangenome contains 11,479 new transcripts but does not deeply analyze the roles of these genes in adaptation or domestication. It is suggested to link functional enrichment results (such as stress response-related genes) to explore their guiding value for region-specific breeding.
6. QTN validation: Have the 81 identified QTNs been validated in independent populations or functional

experiments? Discussing the potential limitations of unverified QTNs or proposing subsequent validation plans is suggested.

7. The ADMIXTURE analysis shows a weak population structure, but whether PCA or kinship matrices were included as covariates in the GWAS is unclear. It is necessary to clarify whether the model accounted for population stratification.

8. GWAS analysis: The article used multiple models (MLMM, FarmCPU, BLINK) to screen QTNs but did not mention the multiple testing correction method (such as Bonferroni or FDR). It is essential to clarify the rationale for determining the significance threshold ($p < 0.0001$).

9. Pangenome construction: It is necessary to provide the parameters of MaSuRCA assembly and the specific criteria for filtering contaminant sequences (such as the E-value threshold of BLASTn).

Minorï¼š

1. Some references lack a DOI (e.g., References 4 and 5), and the format should be standardized according to the journal's requirements.

2. The readability of the figures and tables is poor. The labels in the Circos plot of Figure 1 are too small. Enlarging the chromosome numbers and adding a color legend are recommended. The clarity of Figure 6b is inadequate.

3. The text does not clearly distinguish the differences between the "multi-locus models" of FarmCPU and BLINK. The core assumptions and applicable scenarios of each model should be elaborated on.

4. The evolutionary tree in Figure 3 does not indicate the branch support rate (such as bootstrap values), which should be added to enhance credibility.

5. In Table 1, the unit for "Genome size (k-mer)" should be Gb instead of being unitless; "BUSCO 97.90%" should be fully classified (e.g., "97.9% complete BUSCOs").

6. Some software mentioned in the methods (such as BRAKER3 and EDTA) do not indicate the version number, which should be included to ensure reproducibility.

7. The screening of candidate genes relies on a fixed threshold of "7 kb upstream and downstream", without considering gene function annotations or expression data (such as RNA-seq), resulting in a high risk of false positives.

Methods

Are the methods appropriate to the aims of the study, are they well described, and are necessary controls included? Choose an item.

Conclusions

Are the conclusions adequately supported by the data shown? Choose an item.

Reporting Standards

Does the manuscript adhere to the journal’s guidelines on [minimum standards of reporting?]() Choose an item.

Choose an item.

Statistics

Are you able to assess all statistics in the manuscript, including the appropriateness of statistical tests used? Choose an item.

Quality of Written English

Please indicate the quality of language in the manuscript: Choose an item.

Declaration of Competing Interests

Please complete a declaration of competing interests, considering the following questions:

- Have you in the past five years received reimbursements, fees, funding, or salary from an organisation that may in any way gain or lose financially from the publication of this manuscript, either now or in the future?
- Do you hold any stocks or shares in an organisation that may in any way gain or lose financially from the publication of this manuscript, either now or in the future?
- Do you hold or are you currently applying for any patents relating to the content of the manuscript?
- Have you received reimbursements, fees, funding, or salary from an organization that holds or has applied for patents relating to the content of the manuscript?
- Do you have any other financial competing interests?
- Do you have any non-financial competing interests in relation to this paper?

If you can answer no to all of the above, write 'I declare that I have no competing interests' below. If your reply is yes to any, please give details below.

I declare that I have no competing interests.

I agree to the open peer review policy of the journal. I understand that my name will be included on my report to the authors and, if the manuscript is accepted for publication, my named report including any attachments I upload will be posted on the website along with the authors' responses. I agree for my report to be made available under an Open Access Creative Commons CC-BY license (http://creativecommons.org/licenses/by/4.0/). I understand that any comments which I do not wish to be included in my named report can be included as confidential comments to the editors, which will not be published.

Choose an item.

To further support our reviewers, we have joined with Publons, where you can gain additional credit to further highlight your hard work (see: https://publons.com/journal/530/gigascience). On publication of this paper, your review will be automatically added to Publons, you can then choose whether or not to claim your Publons credit. I understand this statement.

Yes Choose an item.
